# Primary liver cancer burden and its association with health development in the Western Pacific, 1990–2021

**DOI:** 10.3389/fonc.2025.1627330

**Published:** 2025-07-31

**Authors:** Teng-Chao Zhou, Dan Qin, Peng Yan, Bin Lan, Qing Wang, Jie Tan

**Affiliations:** ^1^ Vascular Disease Research Center of Hunan Provincial Geriatric Institute (SLYS04), Changsha, China; ^2^ Department of Interventional Vascular Surgery, Hunan Provincial People’s Hospital (The First Affiliated Hospital of Hunan Normal University), Changsha, China; ^3^ Department of Neurosurgery, Hunan Provincial People’s Hospital (The First Affiliated Hospital of Hunan Normal University), Changsha, China

**Keywords:** regional cancer burden, liver cancer disparities, chronic viral hepatitis, alcohol consumption, metabolic liver disease, health development

## Abstract

**Background:**

Liver cancer (LC) is a major public health challenge in the Western Pacific Region (WPR), characterized by etiological diversity and increasing burden from both infectious and noninfectious causes. This study aimed to assess the burden of primary LC and its association with health system development indicators across the WPR from 1990 to 2021.

**Methods:**

We conducted a secondary analysis using data from the Global Burden of Disease (GBD) Study 2021, covering 31 countries and territories in the WPR. LC burden was evaluated in terms of incidence, prevalence, mortality, and disability-adjusted life years (DALYs), stratified by sex, age, location, and five etiologies: hepatitis B virus (HBV), hepatitis C virus (HCV), alcohol use, nonalcoholic steatohepatitis (NASH), and other causes. Associations with Human Resources for Health (HRH), Universal Health Coverage (UHC) index, and Sociodemographic Index (SDI) were examined using Spearman correlation and generalized linear models.

**Results:**

Between 1990 and 2021, age-standardized DALYs rates for LC in the WPR rose from 281.78 to 334.74 per 100,000 population (EAPC: 0.56%). Total incidence nearly doubled, with sharp increases in Mongolia, Australia, and China. HBV remained the leading cause, while burdens from alcohol use and NASH grew markedly. LC burden showed moderate to strong positive correlations with HRH, UHC, and SDI, especially for HCV- and NASH-related cases; HBV-related burden showed weak associations.

**Conclusions:**

Despite progress in viral hepatitis control, the LC burden continues to rise in the WPR. Integrated strategies targeting viral, metabolic, and alcohol-related causes are urgently needed.

## Background

Primary liver cancer (LC) is a major global health challenge with diverse etiologies, influenced by shifting demographic, behavioral, and virological factors (1, 2). While chronic hepatitis B virus (HBV) and hepatitis C virus (HCV) infections remain dominant causes of LC worldwide, the contribution of nonviral factors, particularly alcohol-related liver disease (ARLD) and NASH, has increased significantly in both high- and low-income regions (3). Moreover, there is a class of LC cases that arise from several other causes, including autoimmune disorders, genetic conditions, and environmental toxins (4).

Despite progress in prevention and treatment, the global burden of LC remains substantial. A study by Tan et al. reported 280,889 new LC cases and 242,689 LC-related deaths in 2021, reflecting respective increases of 20.8% and 17.8% compared to 2010 (5). Although global age-standardized incidence rates (ASIR: 10.16 per 100,000) and mortality rates (ASMR: 8.75 per 100,000) have shown modest annual declines of -1.05% and -1.40%, the absolute burden remains high due to population growth and aging.

The Western Pacific Region (WPR) is disproportionately affected, accounting for over 60% of global LC deaths despite comprising only about a quarter of the world’s population (6). Countries such as China, Vietnam, and Mongolia have some of the highest ASIR and ASMR globally. For instance, Mongolia reported an ASIR exceeding 80 per 100,000 in 2020, ten times higher than the global average (6). While HBV and HCV continue to burden certain populations, shifts in diet and behavior are fueling rising rates of obesity, diabetes, and metabolic syndrome, which are key drivers of liver cancer due to NASH (7, 8). Alcohol use, both occasional and chronic, remains common and further exacerbates the severity of the disease (9).

In parallel, the WPR is characterized by marked heterogeneity in healthcare infrastructure, access to early detection and treatment, and implementation of public health interventions. This variability presents a unique opportunity to explore how liver cancer burden correlates with health system development indicators such as the Socio-demographic Index (SDI), Universal Health Coverage (UHC) index, and healthcare workforce density (HRH). Previous studies using GBD data have highlighted complex and sometimes paradoxical relationships between healthcare development and liver cancer burden. For example, Cao et al. found that HBV-related LC burden remained substantial in higher-SDI countries, where improved diagnostics may increase apparent incidence despite decreasing true disease prevalence (10). Another analysis by the same group observed that in countries with SDI ≥ 0.7, the burden of LC, particularly from nonviral causes, was rising despite improvements in healthcare system capacity (6). Moreover, Danpanichkul et al. emphasized the impact of rural–urban disparities and healthcare workforce shortages on screening and treatment access in China (11). Similarly, Omata et al. noted that countries with more robust cancer registration systems report higher incidence, likely due to superior detection rather than increased biological risk (12).

Given this complex interplay of epidemiology, healthcare capacity, and socioeconomic transformation, a focused analysis of the Western Pacific Region is both timely and necessary. By examining liver cancer burden across five key etiologies—HBV (LCHB), HCV (LCHC), alcohol (LCAL), NASH (LCNA), and other causes (LCOT)—and correlating these patterns with health system indicators. This study aims to provide evidence for regional prioritization and policy interventions tailored to evolving liver cancer challenges in WPR.

## Methods

### Study overview

This study is a secondary analysis using publicly available data from the GBD 2021 study, which provides standardized estimates of disease burden at global, regional, and national levels based on the best available epidemiological evidence. Data were accessed via the GBD Results Tool (https://vizhub.healthdata.org/gbd-results/).

We analyzed LC data from the GBD database, excluding hepatoblastoma. “LC (excluding hepatoblastoma)” reflects the burden attributable to five etiologies: HBV, HCV, alcohol use, NASH, and other causes. Metrics included incidence, prevalence, mortality, and DALYs, reported in absolute numbers and age-standardized rates (ASRs) per 100,000 population. ASRs were calculated using the direct method based on the GBD standard population to ensure comparability across different age structures.

Our study included 31 countries and territories in the World Health Organization (WHO) WPR, categorized by the proportion of the population aged 65 years and over. Seven are aging countries (>13% aged ≥65), nine are in transition (7–13%), and 15 are young (≤7%). This classification allowed for analysis of demographic aging and its impact on liver cancer burden across diverse settings in the WPR.

### Data sources and definition of LC

The GBD 2021 study compiled data from 79,865 previously identified sources and 19,055 new sources via systematic literature review, as listed in the GBD 2021 Sources Tool (https://ghdx.healthdata.org/gbd-2021/sources) (13). This study assessed trends in the burden of LC and its five major causes (LCHB, LCHC, LCAL, LCNA, and LCOT), as well as the relationship between LC burden and health development indicators.

### Burden estimation and indicators

The burden metrics including incidence, prevalence, mortality, and DALYs in the GBD 2021 were extracted (14). Mortality estimates were derived using the Cause of Death Ensemble model (CODEm), which employs a Bayesian geospatial regression framework that combines multiple predictive models, accounts for spatial and temporal correlations, and evaluates performance through out-of-sample validation to generate robust mortality estimates across locations with varying data availability. All estimates included 95% uncertainty intervals (UIs). Uncertainty propagation was managed through Monte Carlo simulations, drawing from posterior distributions of model parameters to account for data variability.

### Health development indicators

To explore the relationship between LC burden and health system development, three indicators were assessed. Human Resources for Health (HRH) density—healthcare workers per 10,000 population—was derived from the GBD 2019 dataset, covering 16 worker categories and estimated via spatiotemporal Gaussian process regression from 1990 to 2019 (15).

The Universal Health Coverage (UHC) Service Coverage Index, aligned with Sustainable Development Goals (SDG) 3.8.1, reflects access to essential health services on a 0–100 scale. Data for 2021 and historical trends were obtained from the WHO Global Health Observatory. Some WPR countries (e.g., Guam, Northern Mariana Islands, Tokelau) lacked UHC data due to surveillance limitations.

The SDI, extracted from GBD 2021, assesses development based on total fertility rate (TFR) under age 25, mean educational attainment (MEA) in adults ≥15, and income per capita. Each factor was normalized and combined using a geometric mean to yield an SDI value ranging from 0 (lowest development) to 1 (highest).

### Data analysis

We ranked 31 countries in the Western Pacific Region (WPR) by age-standardized incidence, prevalence, mortality, and DALYs for liver cancer (LC) in 1990, 2019, and 2021. Spearman’s rank correlation (r_s_) was used to assess associations between LC burden (ASRs) and HRH, UHC Index, and SDI, given its robustness to non-linear relationships. Generalized Linear Models (GLMs) with log-link functions were applied to ASR data. Trends were visualized via scatter plots using locally weighted regression smoothing (LOESS) with the “geom_smooth” function in R (v4.2.2). Estimated Annual Percentage Changes (EAPCs) and 95% confidence intervals (CIs) were calculated from log-linear models using: 100% × (exp[β] − 1), where β is the regression coefficient. Trends were considered statistically significant if *P* < 0.05 or if 95% CIs excluded zero. All analyses and visualizations were conducted using R software version 4.2.2.

## Results

### Trends in population ageing

**Figure 1:**
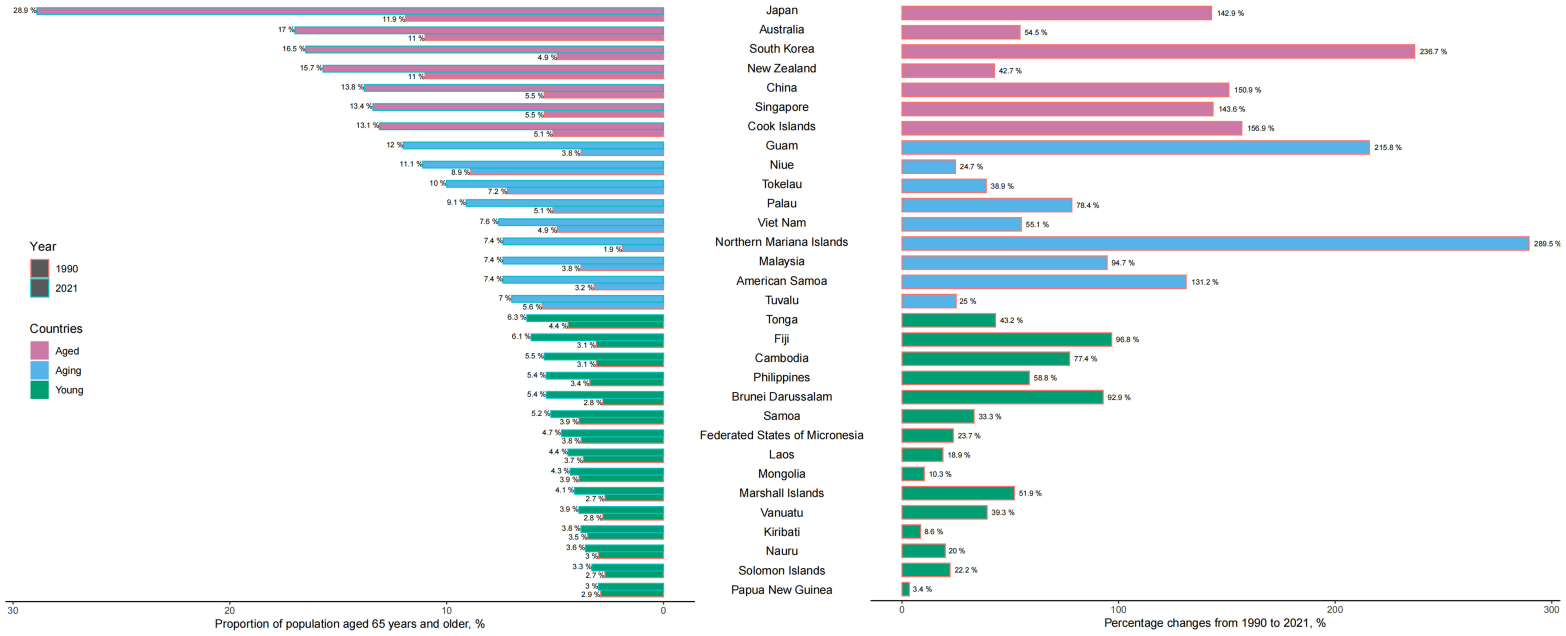
Population aged 65 years and older in the Western Pacific countries in 1990–2021. According to the share of the over-65 population, the Western Pacific countries are grouped into three categories: young (less than 7% aged 65 or over), ageing (7–13%), and aged (more than 13%) countries. The proportion of the over-65 population in 1990 and 2021 (left), and percentage changes in the proportion of the over-65 population from 1990 to 2021 (right).

In 2021, approximately 13.8% (265 million) of the Western Pacific population was aged ≥65 years. Countries with the highest proportions included Japan (28.9%), Australia (17.0%), South Korea (16.5%), and China (13.8%). The pace of ageing increased notably, with proportions of older adults more than doubling in the Cook Islands, China, Singapore, Japan, and American Samoa, and tripling in the Northern Mariana Islands, South Korea, and Guam from 1990 to 2021 (**Figure 1**).

### Trends in total LC

The age-standardized DALYs rate (ASDR) increased from 281.78 per 100,000 in 1990 to 339.73 per 100,000 in 2019, then slightly declined to 334.74 per 100,000 in 2021 (**Table 1**). The estimated annual percentage change (EAPC) from 1990 to 2021 was 0.56%, indicating a sustained rise in disease burden over time. Males consistently exhibited higher DALYs rates than females, with the highest male-specific burden observed in Mongolia, South Korea, and Guam (**Supplementary Table S1**, **Supplementary Figure S1A**). The total number of DALYs rose from 4.34 million (95% UI: 3.89–4.80 million) in 1990 to 6.44 million (95% UI: 5.50–7.38 million) in 2021—a 48.37% increase (95% CI: 14.77%–89.80%) (**Supplementary Table S2**). Mongolia recorded the highest ASDR, increasing from 878.61 to 1518.64 per 100,000 (EAPC: 1.78%). South Korea and Japan showed declining trends (EAPCs: -0.60% and -0.23%, respectively), while Australia increased significantly from 54.16 to 197.23 per 100,000 (EAPC: 4.26%). Upward trends were also seen in the Cook Islands, China, and Palau (**Supplementary Figure S2A**). In rankings, Mongolia consistently held the highest burden, while Northern Mariana Islands rose from 20th in 1990 to 9th in 2021. Australia moved from 30th to 12th over the same period (**Supplementary Figure S3**).

**Table 1:** Age-standardized liver cancer DALYs rates (per 100,000) in the Western Pacific region by member state, in 1990, 2019, and 2021.

Location	Age-standardized DALYs rate, per 100, 000 (95% UI)						EAPC, % (95%CI)	
	1990	Rank	2019	Rank	2021	Rank	1990-2019	1990-2021
Western Pacific region	281.78 (252.40, 311.16)		339.73 (290.18, 389.28)		334.74 (285.92, 383.56)		0.65 (0.48, 0.78)	0.56 (0.4, 0.68)
Aged countries								
South Korea	762.24 (593.69, 930.22)	2	574.4 (483.45, 665.82)	2	632.97 (521.52, 746.14)	2	-0.97 (-1.15, -0.71)	-0.6 (-0.71, -0.42)
Cook Islands	203.58 (159.94, 246.91)	7	380.34 (302.24, 458.21)	4	401.85 (312.97, 488.62)	4	2.18 (2.15, 2.22)	2.22 (2.19, 2.23)
Japan	420.66 (398.71, 442.41)	3	377.94 (348.15, 407.61)	5	392.07 (358.37, 424.62)	5	-0.37 (-0.47, -0.28)	-0.23 (-0.34, -0.13)
China	268.4 (232.32, 305.14)	5	353.6 (291.38, 415.46)	7	341.92 (281.32, 403.13)	7	0.96 (0.78, 1.07)	0.78 (0.62, 0.9)
Australia	54.16 (46.67, 61.54)	30	192.38 (164.96, 219.97)	12	197.23 (168.95, 224.48)	12	4.47 (4.45, 4.49)	4.26 (4.24, 4.26)
Singapore	176.77 (155.93, 197.86)	8	177.01 (150.44, 203.19)	14	191.58 (162.55, 219.69)	13	0 (-0.12, 0.09)	0.26 (0.13, 0.34)
New Zealand	61.29 (56.39, 66.17)	27	143.59 (131.9, 155.24)	16	146.9 (134.95, 158.9)	16	2.98 (2.97, 2.98)	2.86 (2.85, 2.87)
Aging countries								
Viet Nam	249.44 (190.85, 309.19)	6	360.68 (268.28, 452.14)	6	377.78 (277, 477.81)	6	1.28 (1.18, 1.32)	1.35 (1.21, 1.41)
Palau	160.61 (106.27, 215.89)	11	310.08 (235, 384.46)	8	329.65 (244.81, 414.58)	8	2.29 (2.01, 2.77)	2.35 (2.13, 2.73)
Northern Mariana Islands	81.57 (60.16, 103)	20	194.41 (152.74, 236.54)	10	213.97 (172.79, 255.66)	9	3.04 (2.91, 3.27)	3.16 (2.98, 3.46)
Guam	59.07 (49.9, 68.23)	25	178.59 (142.64, 214.13)	9	210.75 (175.5, 245.09)	11	3.88 (3.68, 4.19)	3.79 (3.62, 4.03)
American Samoa	66.49 (51.54, 81.5)	28	200.62 (169.62, 232.44)	13	187.53 (148.95, 226.31)	14	3.89 (3.69, 4.02)	3.8 (3.59, 3.94)
Niue	112.05 (80.87, 143.28)	16	174.91 (122.55, 228.57)	15	179.71 (125.36, 234.86)	15	1.55 (1.44, 1.62)	1.54 (1.42, 1.61)
Malaysia	68.96 (55.72, 82.2)	24	141.28 (113.99, 168.33)	17	143.13 (113.96, 171.81)	17	2.5 (2.5, 2.5)	2.38 (2.33, 2.41)
Tokelau	88.98 (55.37, 122.37)	19	131.39 (88.41, 174.06)	21	142.47 (97.17, 187.89)	18	1.35 (1.22, 1.63)	1.53 (1.39, 1.83)
Tuvalu	101.92 (71.92, 131.08)	17	121.08 (87.44, 155.16)	25	121.87 (87.49, 156.39)	26	0.6 (0.58, 0.68)	0.58 (0.57, 0.63)
Young countries								
Mongolia	878.61 (658.45, 1099.38)	1	1463.9 (1163.36, 1767.97)	1	1518.64 (1190.51, 1842.84)	1	1.78 (1.65, 1.98)	1.78 (1.68, 1.93)
Tonga	376.25 (279.96, 473.87)	4	464.48 (345.25, 584.17)	3	471.62 (346.37, 595.98)	3	0.73 (0.73, 0.72)	0.73 (0.69, 0.74)
Brunei Darussalam	171.3 (130.93, 212.01)	9	193.54 (153.03, 233.86)	11	212.05 (166.44, 258.27)	10	0.42 (0.34, 0.54)	0.69 (0.64, 0.78)
Cambodia	122.36 (72.03, 173.75)	14	136.96 (78.96, 193.41)	19	142.4 (87.05, 199.52)	19	0.39 (0.32, 0.37)	0.49 (0.45, 0.61)
Vanuatu	66.33 (37.4, 94.72)	26	139.17 (125.28, 153.12)	18	141.26 (122.57, 160.46)	20	2.59 (1.67, 4.26)	2.47 (1.71, 3.9)
Laos	167.3 (123.15, 209.41)	10	132.12 (100.36, 164.11)	20	136.28 (101.5, 171.11)	21	-0.81 (-0.84, -0.7)	-0.66 (-0.65, -0.62)
Kiribati	120.47 (93.14, 147.49)	15	129.31 (95.51, 163.52)	22	131.23 (97.83, 164.83)	22	0.24 (0.09, 0.36)	0.28 (0.16, 0.36)
Federated States of Micronesia	80.79 (55.68, 105.27)	21	122.53 (76.33, 167.09)	24	127.6 (82.01, 172.82)	23	1.45 (1.09, 1.61)	1.49 (1.26, 1.61)
Nauru	129.22 (98.8, 160.13)	12	124.06 (82.97, 164.1)	23	126.53 (82.7, 170.12)	24	-0.14 (-0.6, 0.08)	-0.07 (-0.57, 0.2)
Fiji	71.94 (53.21, 91)	23	118.26 (90.53, 146.32)	26	122.73 (92.54, 153.77)	25	1.73 (1.65, 1.85)	1.74 (1.71, 1.8)
Samoa	66.49 (51.54, 81.5)	22	93.82 (72.84, 115.08)	27	95.22 (71.87, 117.87)	27	0.58 (0.54, 0.6)	0.59 (0.46, 0.64)
Solomon Islands	94.83 (33.57, 156.06)	18	92.55 (59.87, 125.57)	28	93.73 (60.97, 126.38)	28	-0.08 (-0.75, 2.02)	-0.04 (-0.68, 1.94)
Philippines	124.1 (100.85, 147.38)	13	83.53 (48.45, 118.37)	29	87.08 (50.07, 123.98)	29	-1.36 (-2.5, -0.75)	-1.14 (-2.23, -0.56)
Marshall Islands	41.63 (25.36, 57.84)	31	77.31 (51.75, 102.91)	30	79.11 (52.16, 105.96)	30	2.16 (2.01, 2.49)	2.09 (1.97, 2.35)
Papua New Guinea	160.61 (106.27, 215.89)	29	42.81 (12.62, 72.22)	31	43.8 (12.51, 75.36)	31	-1.08 (-1.49, -1.02)	-0.94 (-1.42, -0.82)

DALYs, Disability-adjusted life years; UI, Uncertainty interval; EAPC, Estimated annual percentage change; CI, Confidence interval.

The ASIR also rose, from 9.1 per 100,000 in 1990 to 14.55 in 2021 (EAPC: 1.53%). Males had significantly higher incidence rates across all years (**Supplementary Figure S1B**). The number of new LC cases increased from 140,251 in 1990 to 280,124 in 2021, marking a 99.73% increase (95% CI: 58.59%–149.72%) (**Supplementary Table S2**). Among high-burden countries, Mongolia’s ASIR increased from 28.35 to 51.01 (EAPC: 1.91%). Steady increases occurred in South Korea (EAPC: 1.12%) and Japan (EAPC: 1.45%). Australia saw a sharp rise, from 2.11 to 9.72 (EAPC: 5.05%), while China and Vietnam also recorded notable increases (**Supplementary Figure S2B**; **Supplementary Tables S3**, **S4**). In rankings, Mongolia remained first throughout the study period. Australia moved from 26th (1990) to 10th (2021), reflecting a 350.91% increase from 1990 to 2019. Northern Mariana Islands and Guam also experienced rapid increases (**Supplementary Figure S4**).

**Figure 2:**
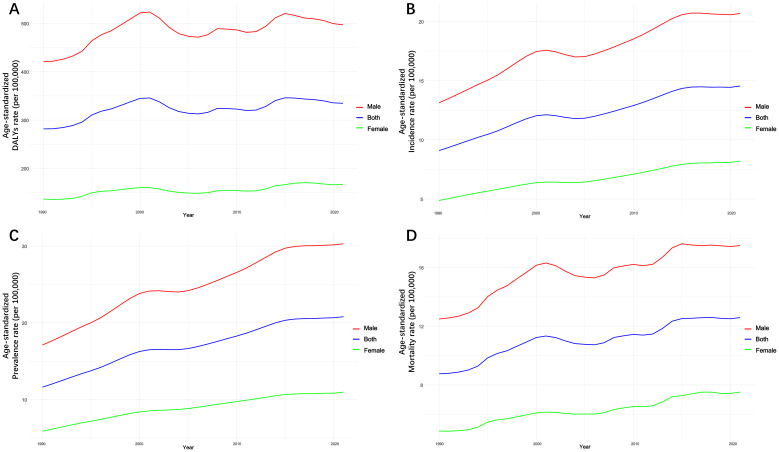
Trends in age-standardized DALYs, incidence, prevalence, and mortality rate of total LC by sex from 1990 to 2021. **(A)** Age-standardized DALYs rate. **(B)** Age-standardized incidence rate. **(C)** Age-standardized prevalence rate. **(D)** Age-standardized mortality rate.

The age-standardized prevalence rate (ASPR) rose from 11.62 per 100,000 in 1990 to 20.79 in 2021 (EAPC: 1.89%), indicating an expanding population of individuals living with LC (**Supplementary Tables S5**, **S6**; **Figure 2C**). The number of prevalent cases increased from 179,116 in 1990 to 400,357 in 2021—a 123.52% rise (95% CI: 78.18%–178.38%) (**Supplementary Table S2**). Prevalence was higher in males (**Supplementary Figure S1C**). South Korea (30.59 to 67.55) and Japan (31.23 to 56.71) had the highest ASPRs. Mongolia rose from 30.76 to 55.25 (EAPC: 1.91%). Australia’s ASPR grew dramatically, from 2.46 to 14.07 (EAPC: 5.79%, 95% CI: 5.75%–5.81%) (**Supplementary Figure S2C**). South Korea surpassed Japan in 2019 to lead prevalence in 2019 and 2021. Australia moved from 26th in 1990 to 10th by 2019–2021, with a 472.06% increase from 1990 to 2019 (**Supplementary Figure S5**).

The ASMR rose from 8.76 per 100,000 in 1990 to 12.59 in 2021 (EAPC: 1.18%, 95% CI: 1.06%–1.27%) (**Supplementary Tables S7**, **S8**; **Figure 2D**). Mortality remained significantly higher in males (**Supplementary Figure S1D**). LC caused 242,358 deaths in 2021 compared to 134,989 in 1990—an increase of 79.54% (95% CI: 43.05%–123.78%) (**Supplementary Table S1**). Mongolia had the highest ASMR, from 29.54 to 53.13 (EAPC: 1.91%, 95% CI: 1.80%–2.08%). Australia recorded the steepest increase, from 2.11 to 8.82 (EAPC: 4.61%, 95% CI: 3.63%–5.57%) (**Supplementary Figure S1D**). Guam and Northern Mariana Islands also had substantial increases (**Supplementary Figure S2D**). Mongolia remained the top-ranked country for mortality. Australia climbed from 26th in 1990 to 10th in 2019 and 2021, marking a 302.88% mortality increase from 1990 to 2019 (**Supplementary Figure S6**).

### Trends in five specific LC

**Figure 3:**
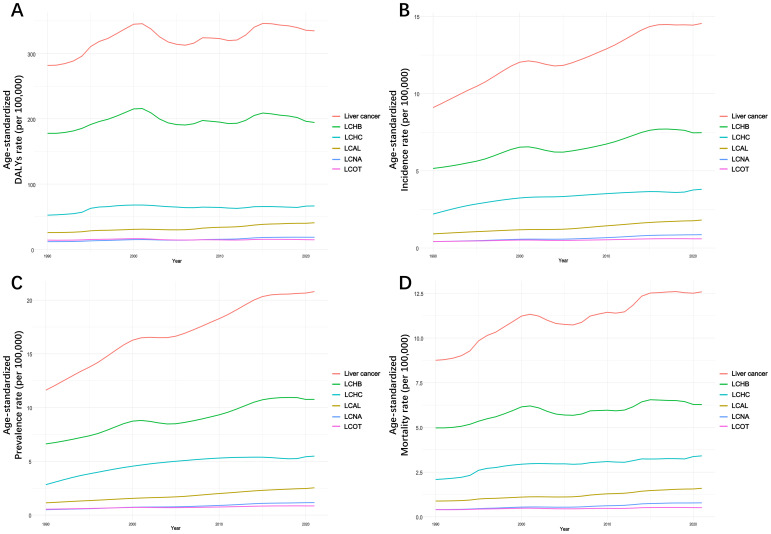
Age-standardized DALYs **(A)**, incidence **(B)**, prevalence **(C)**, and mortality **(D)** rates of total and 5 specific LC in the WPR by years, from 1990 to 2021.

**Figure 4:**
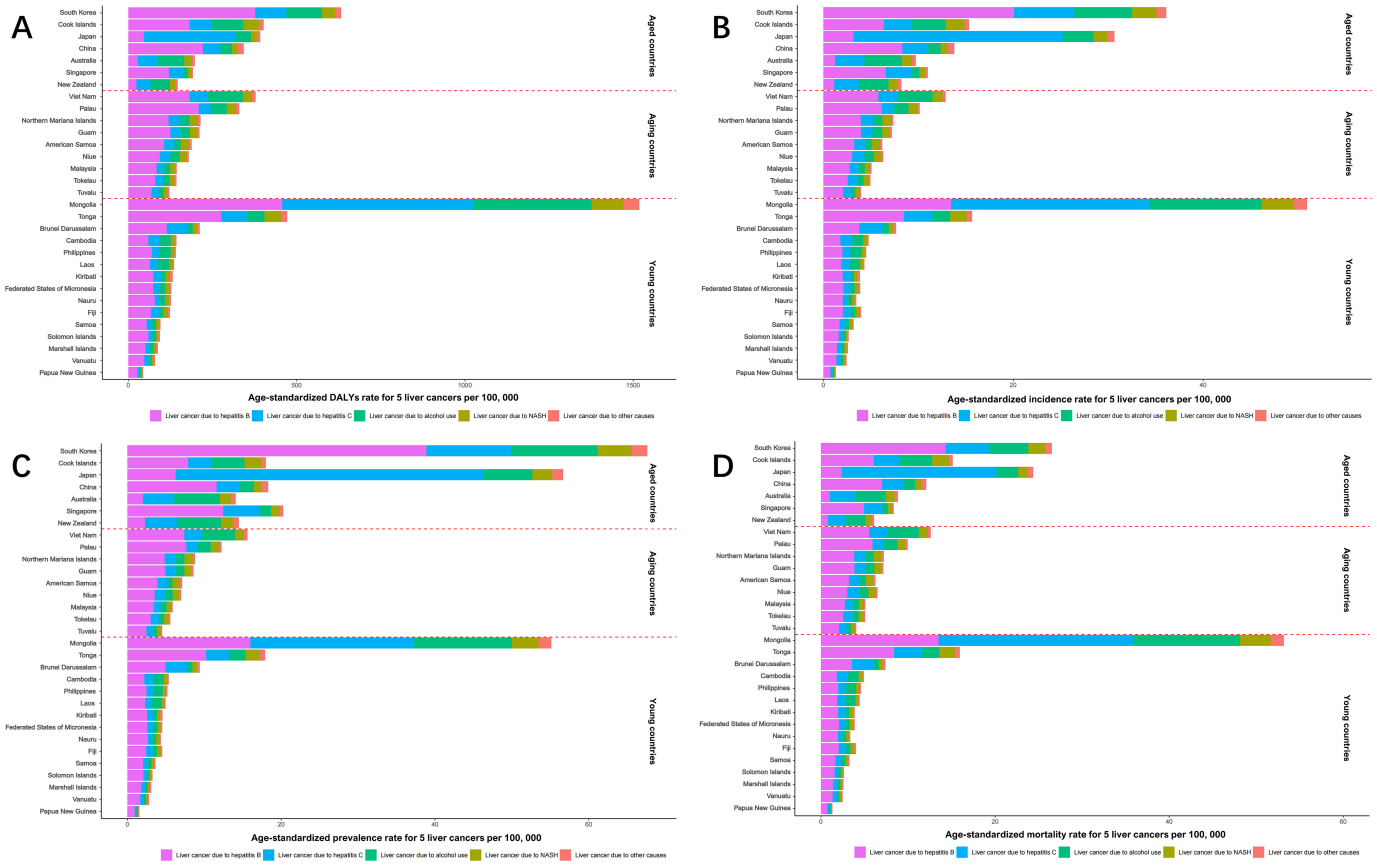
Age-standardized rate of DALYs **(A)**, incidence **(B)**, prevalence **(C)**, and mortality **(D)** for 5 types of LC in the WPR by member state, in 2021. DALYs, disability-adjusted life years.

The disease burden from the five major causes of LC showed varied trends across the WPR from 1990 to 2021. While total LC burden fluctuated, each specific etiology demonstrated a general upward trend over time (**Figures 3**, **4**; **Supplementary Figure S7**; **Supplementary Table S2**). LCHB remained the leading contributor to total LC burden, though its growth has slowed down slightly in recent years. The ASDR of LCHB increased slightly from 177.79 per 100,000 in 1990 to 194.37 per 100,000 in 2021 (EAPC: 0.29%, 95% CI: 0.07%–0.56%). Similarly, the ASIR rose from 5.16 per 100,000 in 1990 to 7.47 per 100,000 in 2021 (EAPC: 1.20%, 95% CI: 1.00%–1.44%). The burden of LCHB peaked in the early 2000s, followed by a modest decline and stabilization. However, incidence, prevalence, and mortality continued to rise, particularly among males, who consistently bore a higher burden than females (**Table 2**, **Supplementary Figure S8**).

**Table 2:** Age-standardized rates (per 100,000) of 5 specific liver cancer in the Western Pacific region, in 1990, 2019, and 2021.

Liver cancer cause	1990 (95% UI)	2019 (95% UI)	2021 (95% UI)	EAPC in 1990-2019, % (95% CI)	EAPC in 1990-2021, % (95% CI)
DALYs					
LCHB	177.79 (151.15, 206.44)	201.89 (160.56, 254.61)	194.37 (154.61, 245.83)	0.44 (0.21, 0.73)	0.29 (0.07, 0.56)
LCHC	52.39 (45.78, 60.05)	64.23 (53.46, 75.13)	66.48 (55.14, 78.94)	0.71 (0.54, 0.78)	0.77 (0.6, 0.89)
LCAL	25.69 (20.43, 32.2)	39.93 (31.72, 51.14)	40.71 (30.82, 53.63)	1.53 (1.53, 1.61)	1.5 (1.34, 1.66)
LCNA	11.8 (9.75, 14.13)	18.57 (14.55, 23)	18.53 (14.39, 22.85)	1.58 (1.39, 1.69)	1.47 (1.26, 1.56)
LCOT	14.11 (11.5, 17.27)	15.11 (11.76, 19.29)	14.65 (11.4, 18.82)	0.24 (0.08, 0.38)	0.12 (-0.03, 0.28)
Incidence					
LCHB	5.16 (4.39, 6.01)	7.63 (6.07, 9.62)	7.47 (5.98, 9.37)	1.36 (1.12, 1.64)	1.2 (1, 1.44)
LCHC	2.2 (1.94, 2.48)	3.62 (3.07, 4.14)	3.8 (3.19, 4.4)	1.73 (1.6, 1.78)	1.78 (1.62, 1.87)
LCAL	0.92 (0.74, 1.13)	1.76 (1.4, 2.22)	1.81 (1.4, 2.34)	2.26 (2.22, 2.36)	2.21 (2.08, 2.38)
LCNA	0.41 (0.34, 0.5)	0.85 (0.67, 1.05)	0.86 (0.68, 1.07)	2.55 (2.37, 2.59)	2.42 (2.26, 2.48)
LCOT	0.42 (0.34, 0.51)	0.6 (0.47, 0.76)	0.6 (0.46, 0.75)	1.24 (1.12, 1.39)	1.16 (0.98, 1.25)
Prevalence					
LCHB	6.62 (5.65, 7.69)	10.93 (8.78, 13.7)	10.77 (8.64, 13.45)	1.74 (1.53, 2.01)	1.58 (1.38, 1.82)
LCHC	2.83 (2.53, 3.15)	5.25 (4.5, 5.92)	5.49 (4.66, 6.3)	2.15 (2.01, 2.2)	2.16 (1.99, 2.26)
LCAL	1.14 (0.92, 1.4)	2.45 (1.97, 3.07)	2.54 (1.96, 3.27)	2.67 (2.66, 2.74)	2.62 (2.47, 2.77)
LCNA	0.5 (0.41, 0.59)	1.14 (0.9, 1.39)	1.16 (0.91, 1.43)	2.88 (2.75, 3)	2.75 (2.61, 2.9)
LCOT	0.54 (0.44, 0.66)	0.86 (0.67, 1.08)	0.85 (0.67, 1.07)	1.62 (1.46, 1.71)	1.47 (1.37, 1.57)
Mortality					
LCHB	4.97 (4.25, 5.78)	6.45 (5.12, 8.09)	6.28 (5.03, 7.87)	0.9 (0.64, 1.17)	0.76 (0.55, 1)
LCHC	2.09 (1.83, 2.38)	3.24 (2.71, 3.73)	3.41 (2.83, 3.95)	1.52 (1.36, 1.56)	1.59 (1.42, 1.65)
LCAL	0.88 (0.71, 1.09)	1.55 (1.24, 1.97)	1.6 (1.24, 2.05)	1.97 (1.94, 2.06)	1.95 (1.81, 2.06)
LCNA	0.41 (0.33, 0.49)	0.78 (0.61, 0.96)	0.78 (0.62, 0.97)	2.24 (2.14, 2.35)	2.1 (2.06, 2.23)
LCOT	0.4 (0.33, 0.49)	0.52 (0.41, 0.66)	0.51 (0.4, 0.64)	0.91 (0.75, 1.03)	0.79 (0.62, 0.87)

UI, Uncertainty interval; EAPC, Estimated annual percentage change; CI, Confidence interval; DALYs, Disability-adjusted life years; LCHB, Liver cancer by HBV; LCHC, Liver cancer by HCV; LCAL, Liver cancer by alcoholic use; LCNA; Liver cancer by nonalcoholic steatohepatitis (NASH); LCOT, Liver cancer by other causes.

LCHC and LCAL demonstrated steady increases, contributing significantly to the overall regional LC burden. For LCHC, the ASDR rose from 52.39 in 1990 to 66.48 in 2021 (EAPC: 0.77%, 95% CI: 0.60%–0.89%), while the ASIR climbed from 2.2 to 3.8 per 100,000 (EAPC: 1.78%, 95% CI: 1.62%–1.87%). The ASDR of LCHC peaked around 2000 before gradually declining and stabilizing in recent years. Incidence and prevalence have continued to rise, with high mortality rates persisting. Males again showed higher burden metrics than females (**Supplementary Figure S9**). LCAL showed a marked increase, with ASDR rising from 25.69 to 40.71 per 100,000 (EAPC: 1.50%, 95% CI: 1.34%–1.66%) and ASIR from 0.92 to 1.81 per 100,000 (EAPC: 2.21%, 95% CI: 2.08%–2.38%). LCAL metrics followed a continuous upward trajectory, accelerating more noticeably after 2000. The sex disparity was particularly prominent, suggesting that alcohol-related LC remains predominantly male-associated (**Supplementary Figure S10**).

LCNA and LCOT displayed more modest burdens but steady upward trends. For LCNA, the ASDR increased from 11.8 to 18.53 per 100,000 (EAPC: 1.47%, 95% CI: 1.26%–1.56%), and the ASIR doubled from 0.41 to 0.86 (EAPC: 2.42%, 95% CI: 2.26%–2.48%). Burden metrics for LCNA rose steadily, with a sharper acceleration noted after 2010. Sex differences were relatively less marked than in other etiologies (**Supplementary Figure S11**). LCOT showed the slowest growth among the five causes. Its ASDR rose slightly from 14.11 to 14.65 (EAPC: 0.12%, 95% CI: -0.03%–0.28%), while ASIR grew from 0.42 to 0.60 per 100,000 (EAPC: 1.16%, 95% CI: 0.98%–1.25%). ASPR and ASMR continued to rise, particularly in males (**Supplementary Figure S12**).

In age-specific trends (**Supplementary Figure S13**), LCHB showed a sharp ASDR rise beginning in middle age, peaking in the elderly, and remaining the highest contributor across all age groups. LCHC presented a delayed but substantial increase in older adults. LCAL and LCNA both increased progressively with age, also peaking in older populations. LCOT remained comparatively low across all age brackets, with only slight increases with advancing age.

**Supplementary Table S9** outlines the ASDRs for all five etiologies across 31 countries in 1990 and 2021. Mongolia consistently held the highest LC burden, particularly due to LCHB, which continued to increase (EAPC: 1.04%). Countries such as Australia, the Cook Islands, and Micronesia experienced rapid rises in LC burden despite access to antiviral treatments. Notably, Australia recorded the fastest ASDR increase for LCNA (EAPC: 5.23%) with similar growth in both men and women, likely driven by rising obesity and metabolic disease prevalence. Comprehensive burden estimates for ASIR, ASPR, and ASMR by cause are provided in **Supplementary Tables S10**-**S12**.

### Distribution of HRH, SDI, and UHC service coverage index

**Supplementary Table S13** presents the distribution of HRH density, SDI, and UHC service coverage index across 31 countries and territories in the WPR, revealing notable improvements in health workforce capacity, socio-economic development, and healthcare accessibility over the past three decades, albeit with marked inter-country variations.

From 1990 to 2019, all countries showed increases in HRH density. Australia (483.13 per 10,000), New Zealand (471.55), and Japan (386.81) recorded the highest densities in 2019. China showed the most significant growth, from 29.4 to 140.23 (EAPC: 5.53%). Conversely, Papua New Guinea (24.24) and Solomon Islands (42.03) had the lowest densities. Although countries such as Laos, Cambodia, and the Solomon Islands improved, their HRH densities remain low, signaling ongoing healthcare workforce challenges.

SDI showed moderate to high growth across most countries. High-SDI nations like Japan and Australia experienced modest increases (~10–15%) between 1990 and 2021. The largest SDI gains were seen in Laos (+88.46%), Cambodia (+62.07%), and China (+56.52%), reflecting strong socio-economic progress. However, Papua New Guinea (0.42), Solomon Islands (0.43), and Vanuatu (0.47) had the lowest SDIs in 2021, indicating persistent barriers to education, income, and fertility-related development.

The UHC service coverage index also improved significantly. South Korea and Singapore (89), Australia (87), and Japan (83) achieved the highest scores in 2021, reflecting robust healthcare systems. The largest improvements from 2000 to 2021 were observed in Cambodia (+141.67%), Laos (+108%), and Vietnam (+83.78%). Nevertheless, disparities remain. Papua New Guinea recorded the lowest UHC score (30), indicating critical healthcare access limitations. The Cook Islands experienced a slight decline (-2.13%), suggesting recent setbacks in service coverage.

### Associations between LC and Health Development Indicators

**Figure 5:**
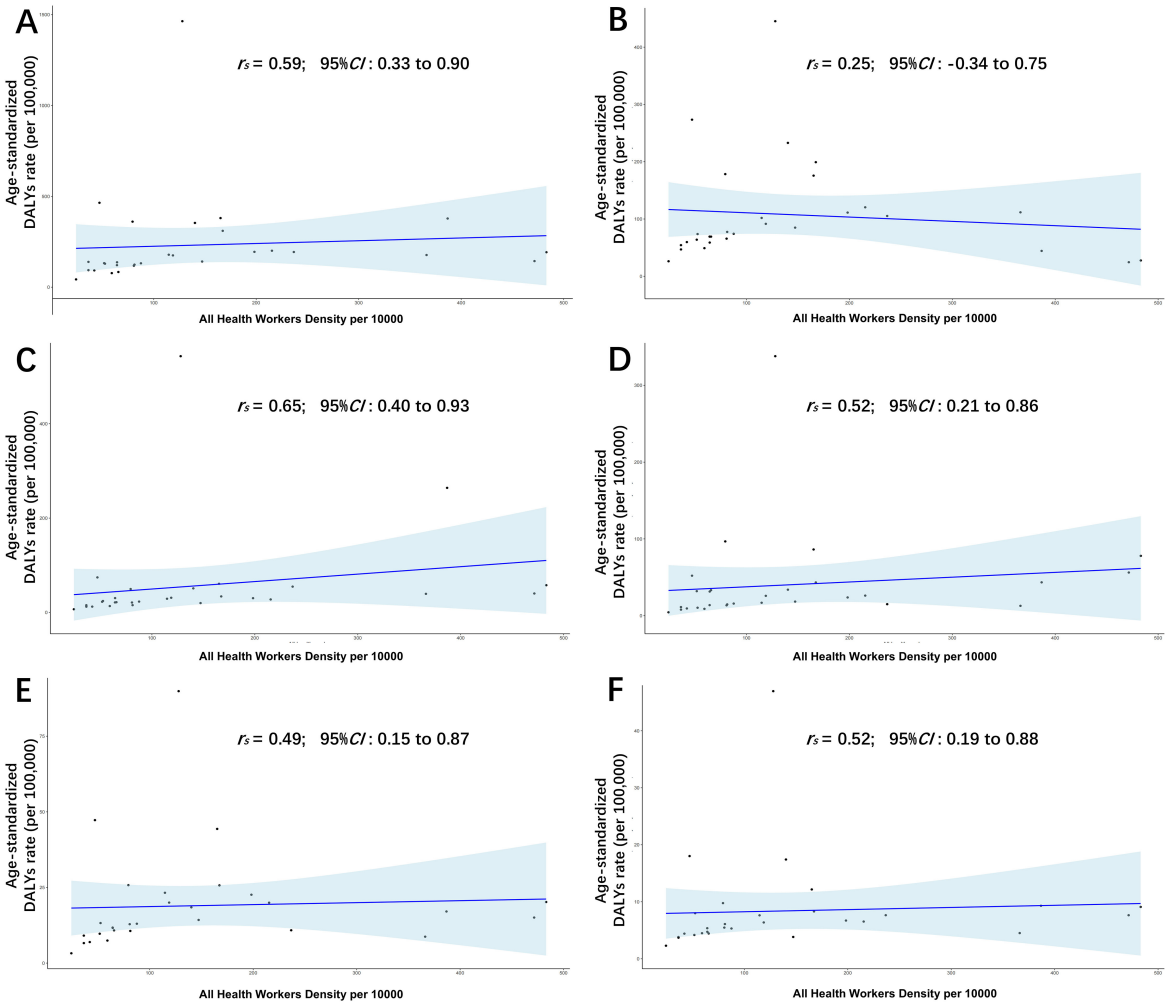
Associations of age-standardized DALYs rate of total **(A)**, LCHB **(B)**, LCHC **(C)**, LCAL **(D)**, LCNA **(E)**, LCOT **(F)** with human resource for health density in the WPR, in 2019.

**Figure 5** and **Supplementary Figures S14**-**S16** demonstrate the associations between LC burden (ASDR, ASIR, ASPR, and ASMR) and HRH density in the WPR (2019). A moderate positive correlation was observed between HRH density and total LC ASDR (r_s_ = 0.59, 95% CI: 0.33–0.90). Among the five etiologies, LCHC showed the strongest correlation (r_s_ = 0.65, 95% CI: 0.40–0.93), while LCAL, LCNA, and LCOT also showed moderate positive correlations. LCHB showed a weak, non-significant association with HRH density. Similar moderate or strong correlations were seen across ASIR, ASPR, and ASMR for total LC, with LCHB again displaying the weakest association.

**Supplementary Figures S17**-**S20** reveal positive associations between SDI and total LC burden in 2021. Correlations were moderate to strong for ASDR (r_s_ = 0.64), ASIR (r_s_ = 0.69), and ASMR (r_s_ = 0.66), and strongest for ASPR (r_s_ = 0.74). All five LC causes showed positive correlations with SDI, with LCHC again strongest and LCHB weakest. These results suggest LC burden rises with socio-economic development, particularly for non-HBV-related etiologies.

**Supplementary Figures S21**-**S24** illustrate correlations between the LC burden and the UHC Service Coverage Index in 2021. Moderate positive associations were found for ASDR (r_s_ = 0.48), ASIR (r_s_ = 0.53), ASPR (r_s_ = 0.59), and ASMR (r_s_ = 0.50). LCHB showed weak, non-significant correlations across most metrics. Notably, ASPR of LCHB and ASMR of LCNA had only weak positive associations, suggesting limited impact of UHC expansion on HBV- and NASH-related LC.

## Discussion

LC remains a significant public health challenge in the WPR, with an overall increasing trend in age-standardized DALYs, incidence, prevalence, and mortality. The ASDR increased from 281.78 to 334.74 per 100,000 between 1990 and 2021(EAPC: 0.56%). ASIR and ASPR nearly doubled over the study period, notably in Australia, Mongolia, and China, indicating a growing patient population. Mortality cases increased by 79.54% (from 134,989 deaths in 1990 to 242,358 deaths in 2021), reflecting the ongoing challenges in LC prevention and early detection. Males consistently showed a higher burden than females, particularly in Mongolia, South Korea, and China (16).

These findings align with prior GBD studies and national registries, confirming that LC remains a leading cause of cancer-related mortality in the WPR due to high-risk exposures and inadequate early detection (17). WHO reported LC as the sixth most common but second deadliest cancer in the region in 2022, largely due to late diagnoses and limited treatment access (18). A GBD study by Liu et al. predicted global LC incidence would decline post-2019, consistent with our data showing plateaued ASIR, ASPR, and ASMR since 2015, alongside a decreasing ASDR (19). The stabilization of the LC burden may result from improved control of hepatitis B and C, advances in early detection and treatment, lifestyle modifications, and variations in diagnostic practices (2, 20).

Population aging plays a crucial role in shaping the epidemiology of liver cancer, particularly in high- and middle-income countries, where the proportion of the population aged ≥65 years is increasing. Our study found that countries classified as having a high aging level tended to report a disproportionately higher liver cancer burden across most etiologies. This trend is consistent with the biological latency of LC, with risk accumulating over time due to progression of chronic liver disease (whether viral hepatitis or metabolic injury) (21). In an aging society, even if the age-standardized incidence rate remains stable or decreases, the increase in the elderly population will increase the absolute number of liver cancers. Notably, the burden of NASH and alcohol consumption is greatest in countries with a high aging population, reflecting shifts in lifestyle and longer exposure windows (22). These findings highlight the need for age-appropriate screening strategies, particularly in settings where healthcare systems must prepare for the volume and complexity of liver cancer cases in older adults.

Disease burden trends varied by the five etiologies of LC. While LCHB and LCHC burdens slightly increased (EAPCs: 0.29% and 0.77%), their growth has slowed. ASIR of LCHB is stabilizing or decreasing in several countries. HBV and HCV remain dominant causes of LC, though vaccination and antivirals are reducing their impact (4, 23).

The burden of LCAL increased substantially from 1990 to 2021, with the ASDR rising at EAPC of 1.50%, while ASIR and ASMR increased even more rapidly (EAPCs of 2.21% and 1.95%, respectively). A global study found that the WPR had the highest LCAL ASIR and ASDR among young people worldwide, underscoring the whole world and region’s urgent alcohol-related health crisis—especially in high-burden countries like Australia, Vietnam, the Cook Islands, Japan, Mongolia, New Zealand, and South Korea (24). The burden of LCNA has increased dramatically, especially in high-income countries such as Australia and Japan, which is associated with the rising prevalence of obesity and metabolic syndrome (25). This trend calls for national strategies promoting physical activity and metabolic health, especially in high-SDI countries (26). In contrast, LCOT showed the slowest growth, suggesting a lesser contribution of nontraditional etiologies relative to HBV, HCV, alcohol, and NASH (27). Overall, these trends align with previous projections: HBV-related LC is declining due to vaccination, while alcohol- and NASH-related cases are increasing, and HCV-related burden fluctuates with antiviral advancements (19, 28).

Our study identified moderate to strong positive associations between LC burden and health system development indicators—HRH density, SDI, and UHC service coverage. HRH reflects the capacity of the healthcare workforce, a core determinant of diagnostic, treatment, and follow-up services. SDI, a composite measure of income per capita, educational attainment, and fertility rate, captures the broader socioeconomic context that shapes health behaviors and policy investments. The UHC index reflects the extent to which essential health services are available and accessible across populations. Collectively, these indicators offer a holistic view of health development by encompassing structural, social, and service delivery dimensions, allowing us to examine how these interact with liver cancer burden at the population level. HRH density was moderately correlated with total LC burden (r_s_ = 0.59), especially HCV-related LC (r_s_ = 0.65), suggesting enhanced detection and treatment where the healthcare workforce is denser. However, HBV-related LC showed only weak correlation with HRH. SDI showed strong positive associations, particularly with LC prevalence (r_s_ = 0.74) and incidence (r_s_ = 0.69), indicating higher burdens in high-SDI countries such as Japan and Australia. UHC service coverage showed moderate correlation (r_s_ = 0.48–0.59) with overall LC burden, but again weaker for HBV-related LC.

Although the increasing burden of liver cancer may seem counterintuitive amid advancements in healthcare systems, this trend is consistent with prior studies demonstrating higher morbidity and mortality rates in countries with higher SDI and Universal Health Coverage Index (UHCI) (6). It is generally expected that more developed health systems and stronger socioeconomic conditions lead to reduced burden from infectious disease-related cancers, such as HBV- and HCV-associated liver cancer. However, several factors may explain the positive correlations we observed between liver cancer burden and health development indicators (HRH density, SDI, and UHC).

First, life expectancy in countries in the WPR has increased significantly, from 72.0 years in 2000 to 77.4 years in 2021, contributing to an expanding elderly population that is intrinsically at higher risk for liver cancer (29, 30). Our findings underscore a consistent age-related gradient in disease burden across all five liver cancer etiologies, highlighting the substantial impact of population aging on the regional epidemiology of liver cancer. This demographic shift is likely the most influential factor underlying the observed positive correlation between liver cancer burden and indicators of health system development. Second, countries or regions with more developed health systems often exhibit improved diagnostic capacity and reporting accuracy for liver cancer, which may contribute to higher recorded incidence and mortality rates (31, 32). Conversely, the widespread availability of hepatitis B vaccination and anti-virus therapy may mitigate this detection bias, leading to a weaker correlation between the burden of LCHB and health system development indicators (33). Lastly, socioeconomic advancement is frequently associated with lifestyle-related risk factors, such as physical inactivity, unhealthy dietary patterns, and increased alcohol consumption, which contribute to metabolic disorders including obesity and NASH. These conditions substantially elevate the risk of LC, particularly in high-SDI countries, thereby reinforcing the increasing LC burden attributed to metabolic and alcoholic etiologies (34). Therefore, the higher burden of LC in countries with stronger health systems reflects a combination of aging populations, improved detection, and a shift from infectious to non-communicable etiologies. Rather than contradicting prior knowledge, these findings point to an evolving epidemiological landscape in the WPR and reinforce the need for integrated strategies that address both traditional and emerging causes of liver cancer (29, 33).

Based on these findings, several policy recommendations are warranted. First, strengthening HBV and HCV Elimination Strategies is essential to curb the increase of the LC burden. Although the burden of LCHB is stabilizing, continued efforts are needed to increase birth-dose HBV vaccination coverage and expand access to antiviral treatment. HCV treatment should be prioritized, especially in high-burden countries such as Mongolia and China, by strengthening access to screening and direct-acting antiviral (DAA) therapy. Given the rapid growth of LCAL and LCNA, targeted interventions, such as alcohol control policies and metabolic disease prevention, are needed to curb this increasing trend (35).

This study has several limitations. First, GBD estimates are based on modeled data from diverse sources, potentially introducing uncertainties. Second, variations in data quality and reporting accuracy across countries may affect the study results. Third, our analysis did not assess LC treatment outcomes or cost-effectiveness, which are critical for guiding healthcare policy. To address these gaps, more comprehensive studies are necessary to increase the understanding of the epidemiology of LC. These include, but are not limited to, the impact of emerging therapies and targeted interventions on LC survival; longitudinal studies evaluating the causal relationship between health system factors and LC burden; and economic evaluations of HBV, HCV, and NASH prevention programs.

## Conclusion

LC remains a growing public health burden in the WPR. While HBV remains the dominant etiology, there is a clear epidemiological shift toward alcohol-related and metabolic causes such as NASH, particularly in high-SDI countries. Our results showed moderate to strong positive correlations between LC burden and health development indicators (HRH density, SDI, and UHC service coverage), which may be associated with increased life expectancy and improved detection rates. Furthermore, there was a significant cumulative age-related effect for all five LC etiologies. Urgent and targeted prevention strategies are essential, including HBV/HCV elimination, alcohol control policies, and national programs to address obesity and metabolic disease. Strengthening screening, ensuring equitable access to care, and investing in the health workforce will be critical to mitigating the rising burden and improving long-term outcomes across the region.

## Data Availability

The raw data supporting the conclusions of this article will be made available by the authors, without undue reservation.
