# Supplementary material for: Primary liver cancer burden and its association with health development in the Western Pacific, 1990–2021
Source: Front Oncol. 2025 Jul 31;15:1627330. doi: 10.3389/fonc.2025.1627330 (PMC12350082; doi:10.3389/fonc.2025.1627330)
Supplement: Supplementary file 1 [file DataSheet1.docx]

Supplementary materials for: Primary liver cancer burden and its association with health development in the Western Pacific, 1990–2021

**Supplementary figures**

Figure S1. Age-standardized DALYs (A), incidence (B), prevalence (C), and mortality (D) rate of LC in the WPR by member state and sex, in 1990 and 2021…………04

Figure S2. Age-standardized DALYs (A), incidence (B), prevalence (C) and mortality (D) rate of LC in the WPR by member state, from 1990 to 2021……………..05

Figure S3. Rank of age-standardized DALYs rate of LC in the WPR, in 1990–2019–2021………………………………………………………………………………..06

Figure S4. Rank of age-standardized incidence rate of LC in the WPR, in 1990–2019–2021……………………………………………………………………………...07

Figure S5. Rank of age-standardized prevalence rate of LC in the WPR, in 1990–2019–2021…………………………………………………………………………….08

Figure S6. Rank of age-standardized mortality rate of LC in the WPR, in 1990–2019–2021……………………………………………………………………………09

Figure S7. Cases of DALYs (A), incidence (B), prevalence (C) and mortality (D) rate of 5 specific LC in the WPR by years, from 1990 to 2021………………………...10

Figure S8. Trends in age-standardized DALYs, incidence, prevalence, and mortality rate of LCHB by sex from 1990 to 2021. (A) Age-standardized DALYs rate. (B) Age-standardized incidence rate. (C) Age-standardized prevalence rate. (D) Age-standardized mortality rate……………………………………………………………11

Figure S9. Trends in age-standardized DALYs, incidence, prevalence, and mortality rate of LCHC by sex from 1990 to 2021. (A) Age-standardized DALYs rate. (B) Age-standardized incidence rate. (C) Age-standardized prevalence rate. (D) Age-standardized mortality rate…………………………………………………………...12

Figure S10. Trends in age-standardized DALYs, incidence, prevalence, and mortality rate of LCAL by sex from 1990 to 2021. (A) Age-standardized DALYs rate. (B) Age-standardized incidence rate. (C) Age-standardized prevalence rate. (D) Age-standardized mortality rate…………………………………………………………...13

Figure S11. Trends in age-standardized DALYs, incidence, prevalence, and mortality rate of LCNA by sex from 1990 to 2021. (A) Age-standardized DALYs rate. (B) Age-standardized incidence rate. (C) Age-standardized prevalence rate. (D) Age-standardized mortality rate…………………………………………………………...14

Figure S12. Trends in age-standardized DALYs, incidence, prevalence, and mortality rate of LCOT by sex from 1990 to 2021. (A) Age-standardized DALYs rate. (B) Age-standardized incidence rate. (C) Age-standardized prevalence rate. (D) Age-standardized mortality rate…………………………………………………………...15

Figure S13. Age-standardized DALYs (A), incidence (B), prevalence (C) and mortality (D) rate of 5 specific LC in the WPR by age in 2021…………………………...16

Figure S14. Associations of age-standardized incidence rate of total (A), LCHB (B), LCHC (C), LCAL (D), LCNA (E), LCOT (F) with human resource for health density in the WPR, in 2019……………………………………………………………………………………………………………………………………………….17

Figure S15. Associations of age-standardized prevalence rate of total (A), LCHB (B), LCHC (C), LCAL (D), LCNA (E), LCOT (F) with human resource for health density in the WPR, in 2019……………………………………………………………………………………………………………………………………………….18

Figure S16. Associations of age-standardized mortality rate of total (A), LCHB (B), LCHC (C), LCAL (D), LCNA (E), LCOT (F) with human resource for health density in the WPR, in 2019……………………………………………………………………………………………………………………………………………….19

Figure S17. Associations of age-standardized DALYs rate of total (A), LCHB (B), LCHC (C), LCAL (D), LCNA (E), LCOT (F) with socio-demographic index in the WPR, in 2021………………………………………………………………………………………………………………………………………………………….…..20

Figure S18. Associations of age-standardized incidence rate of total (A), LCHB (B), LCHC (C), LCAL (D), LCNA (E), LCOT (F) with socio-demographic index in the WPR, in 2021…………………………………………………………………………………………………………………………………………………………..21

Figure S19. Associations of age-standardized prevalence rate of total (A), LCHB (B), LCHC (C), LCAL (D), LCNA (E), LCOT (F) with socio-demographic index in the WPR, in 2021…………………………………………………………………………………………………………………………………………………………..22

Figure S20. Associations of age-standardized DALYs rate of total (A), LCHB (B), LCHC (C), LCAL (D), LCNA (E), LCOT (F) with UHC service coverage index in the WPR, in 2021…………………………………………………………………………………………………………………………………………………………..23

Figure S21. Associations of age-standardized mortality rate of total (A), LCHB (B), LCHC (C), LCAL (D), LCNA (E), LCOT (F) with socio-demographic index in the WPR, in 2021……………………………………………………………………………………………………………………………………………………………...24

Figure S22. Associations of age-standardized incidence rate of total (A), LCHB (B), LCHC (C), LCAL (D), LCNA (E), LCOT (F) with UHC service coverage index in the WPR, in 2021……………………………………………………………………………………………………………………………………………………….25

Figure S23. Associations of age-standardized prevalence rate of total (A), LCHB (B), LCHC (C), LCAL (D), LCNA (E), LCOT (F) with UHC service coverage index in the WPR, in 2021……………………………………………………………………………………………………………………………………………………….26

Figure S24. Associations of age-standardized mortality rate of total (A), LCHB (B), LCHC (C), LCAL (D), LCNA (E), LCOT (F) with UHC service coverage index in the WPR, in 2021…………………………………………………………………………………………………………………………………………………………..27

**Supplementary tables**

Table S1. Age-standardized liver cancer DALYs rates (per 100,000) in the Western Pacific region by member state and sex, in 1990, 2019, and 2021…………………28

Table S2. Cases of 5 specific liver cancer in the Western Pacific region, in 1990, 2019, and 2021…………………………………………………………………………31

Table S3. Age-standardized liver cancer incidence rates (per 100,000) in the Western Pacific region by member state, in 1990, 2019, and 2021…………………………34

Table S4. Age-standardized liver cancer incidence rates (per 100,000) in the Western Pacific region by member state and sex, in 1990, 2019, and 2021……………….36

Table S5. Age-standardized liver cancer prevalence rates (per 100,000) in the Western Pacific region by member state, in 1990, 2019, and 2021………………………39

Table S6. Age-standardized liver cancer prevalence rates (per 100,000) in the Western Pacific region by member state and sex, in 1990, 2019, and 2021………………41

Table S7. Age-standardized liver cancer mortality rates (per 100,000) in the Western Pacific region by member state, in 1990, 2019, and 2021…………………………44

Table S8. Age-standardized liver cancer mortality rates (per 100,000) in the Western Pacific region by member state and sex, in 1990, 2019, and 2021………………...46

Table S9. Age-standardized DALYs rates (per 100,000) for specific liver cancer in the Western Pacific region by member state and sex, in 1990 to 2021………………49

Table S10. Age-standardized incidence rates (per 100,000) for specific liver cancer in the Western Pacific region by member state and sex, in 1990 to 2021……………64

Table S11. Age-standardized prevalence rates (per 100,000) for specific liver cancer in the Western Pacific region by member state and sex, in 1990 to 2021…………..79

Table S12. Age-standardized mortality rates (per 100,000) for specific liver cancer in the Western Pacific region by member state and sex, in 1990 to 2021………….94

Table S13. Trends in human resources for health density (1990–2019), socio-demographic index (1990-2021) and universal health coverage (1990-2021) in the Western Pacific region by member state…………………………………………………………………………………………………………………………………………...109

Figure S1. Age-standardized DALYs (A), incidence (B), prevalence (C), and mortality (D) rate of LC in the WPR by member state and sex, in 1990 and 2021


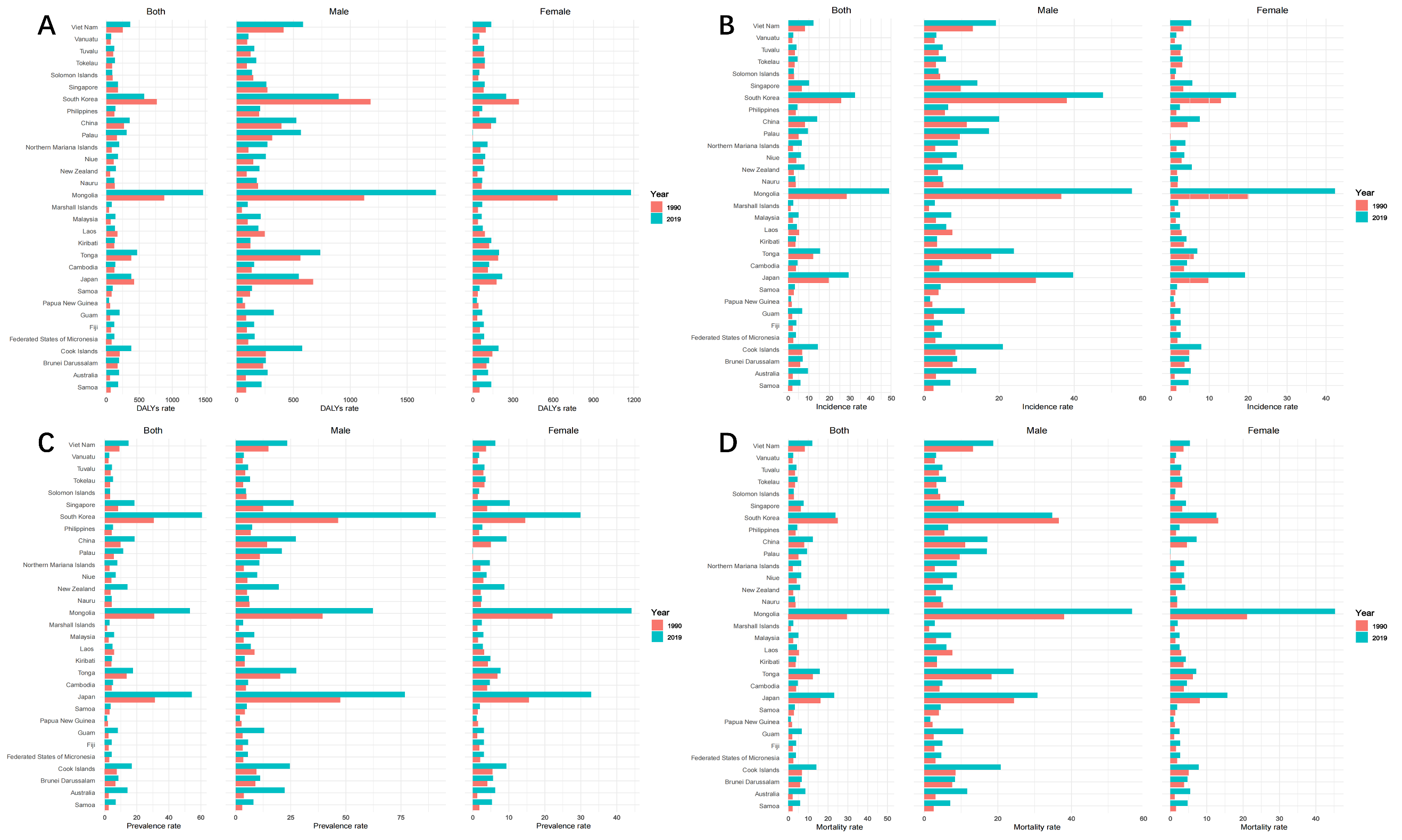


DALYs = disability-adjusted life years; LC = liver cancer; WPR = western pacific region

Figure S2. Age-standardized DALYs (A), incidence (B), prevalence (C) and mortality (D) rate of LC in the WPR by member state, from 1990 to 2021


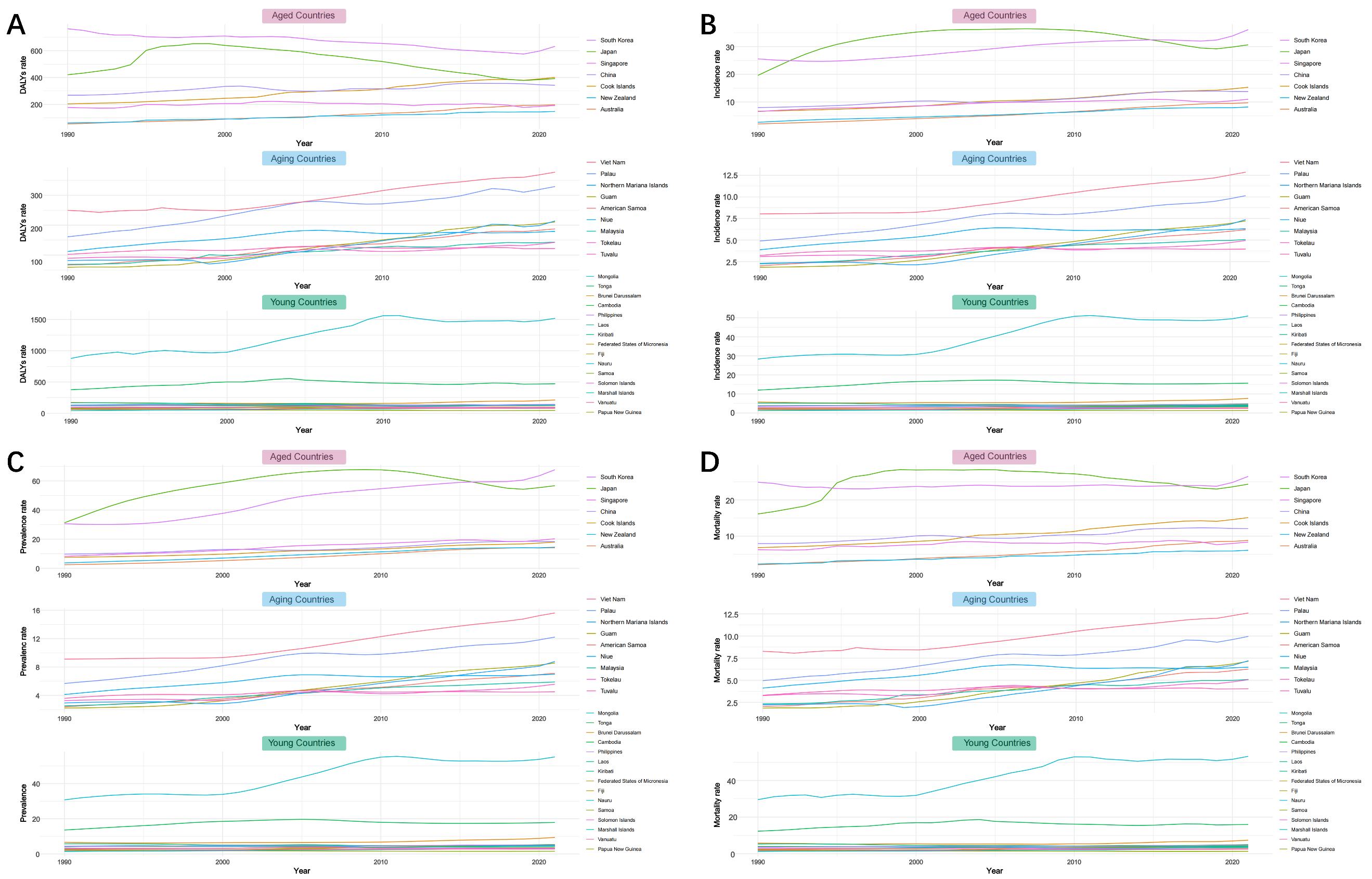


DALYs = disability-adjusted life years; LC = liver cancer; WPR = western pacific region

Figure S3. Rank of age-standardized DALYs rate of LC in the WPR, in 1990–2019–2021


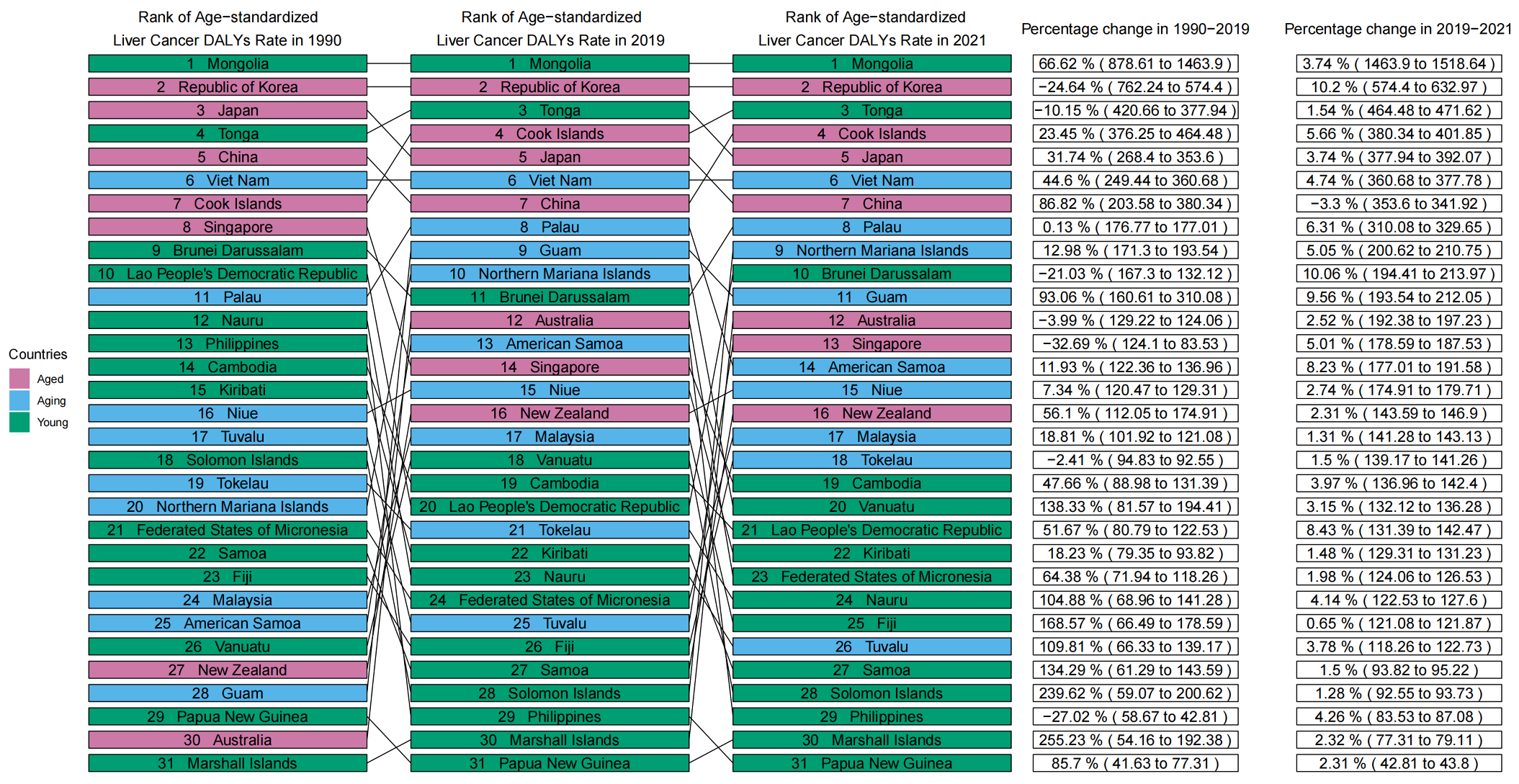


DALYs = disability-adjusted life years; LC = liver cancer; WPR = western pacific region

Figure S4. Rank of age-standardized incidence rate of LC in the WPR, in 1990–2019–2021


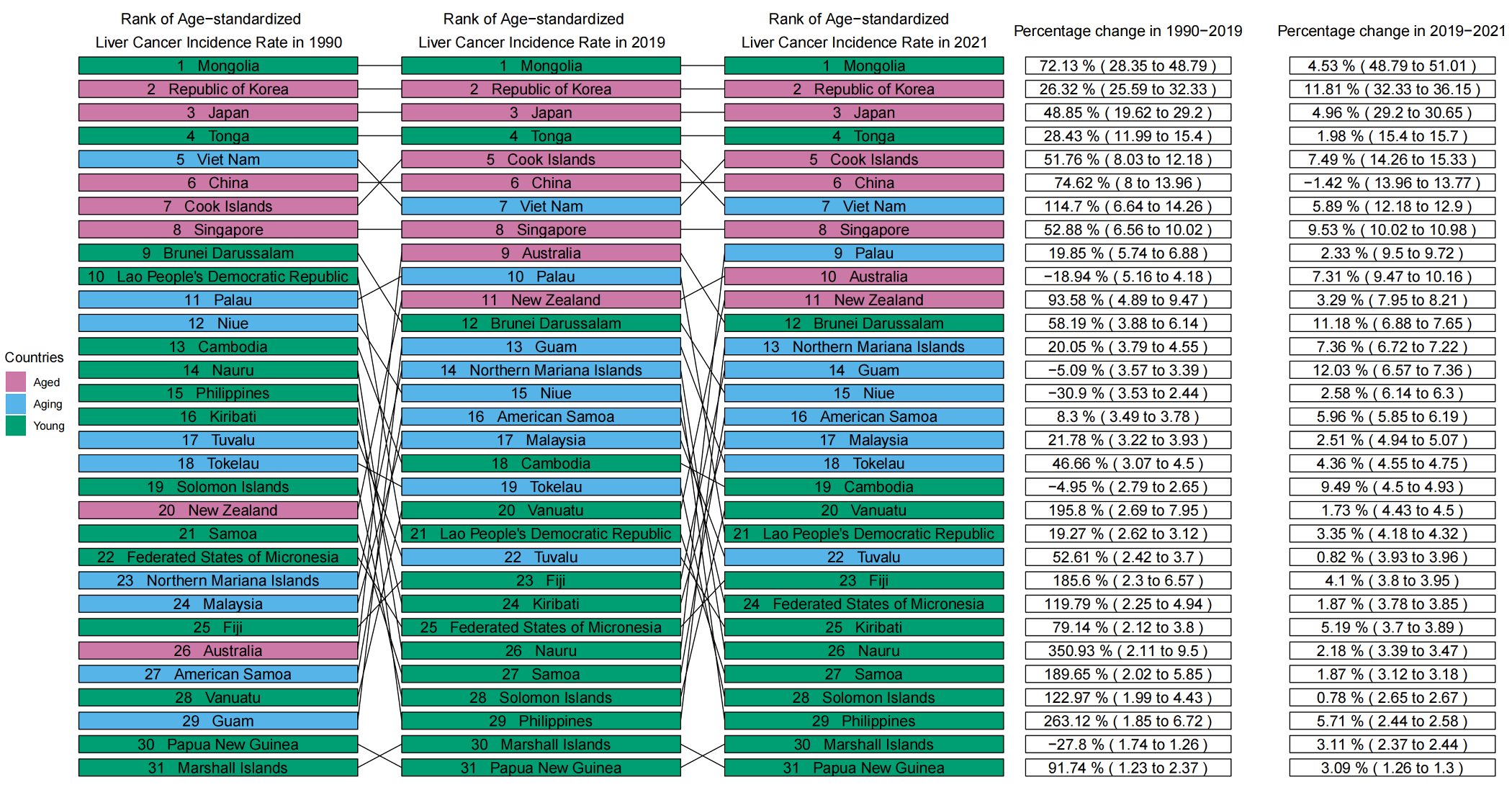


LC = liver cancer; WPR = western pacific region

Figure S5. Rank of age-standardized prevalence rate of LC in the WPR, in 1990–2019–2021


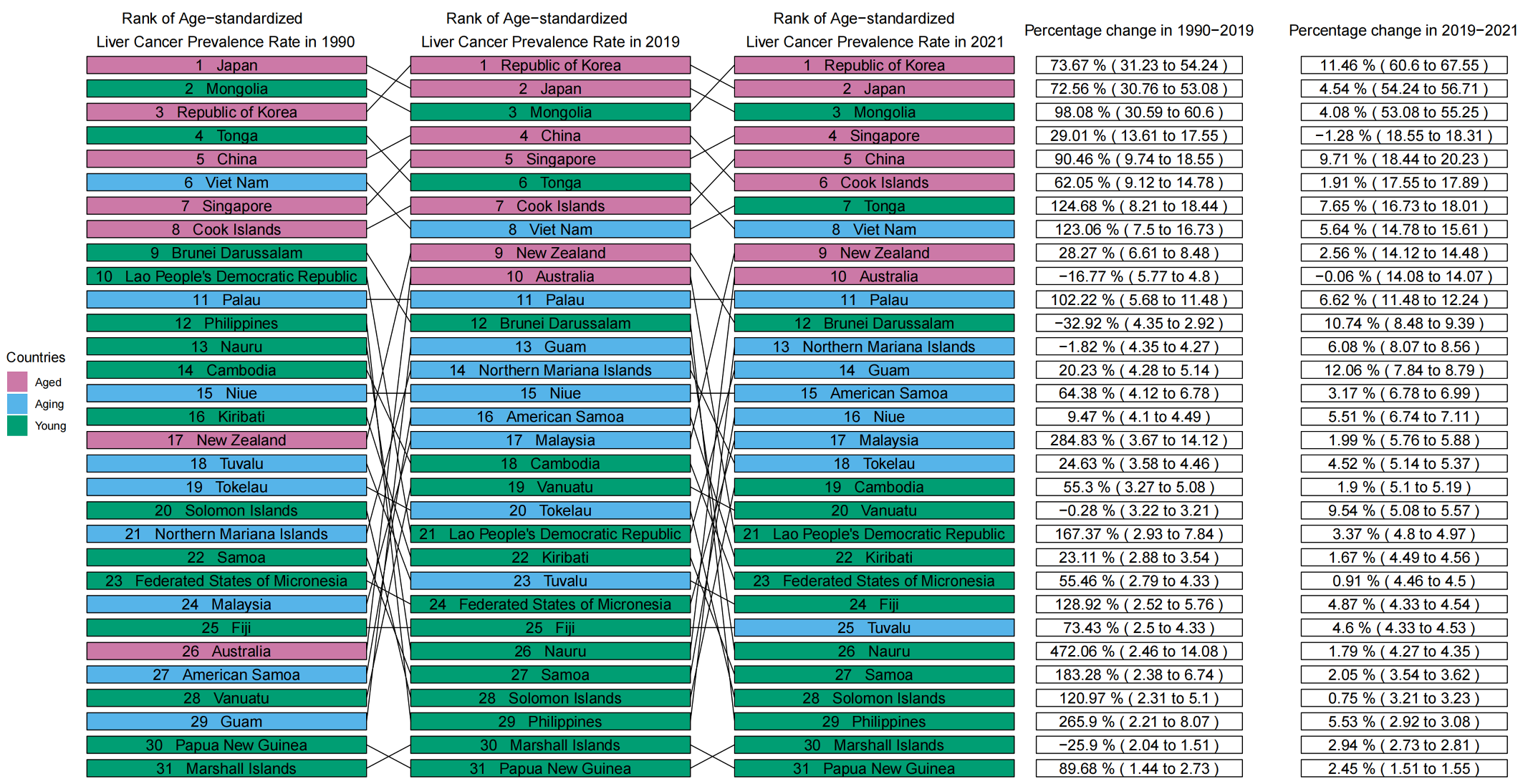


LC = liver cancer; WPR = western pacific region

Figure S6. Rank of age-standardized mortality rate of LC in the WPR, in 1990–2019–2021


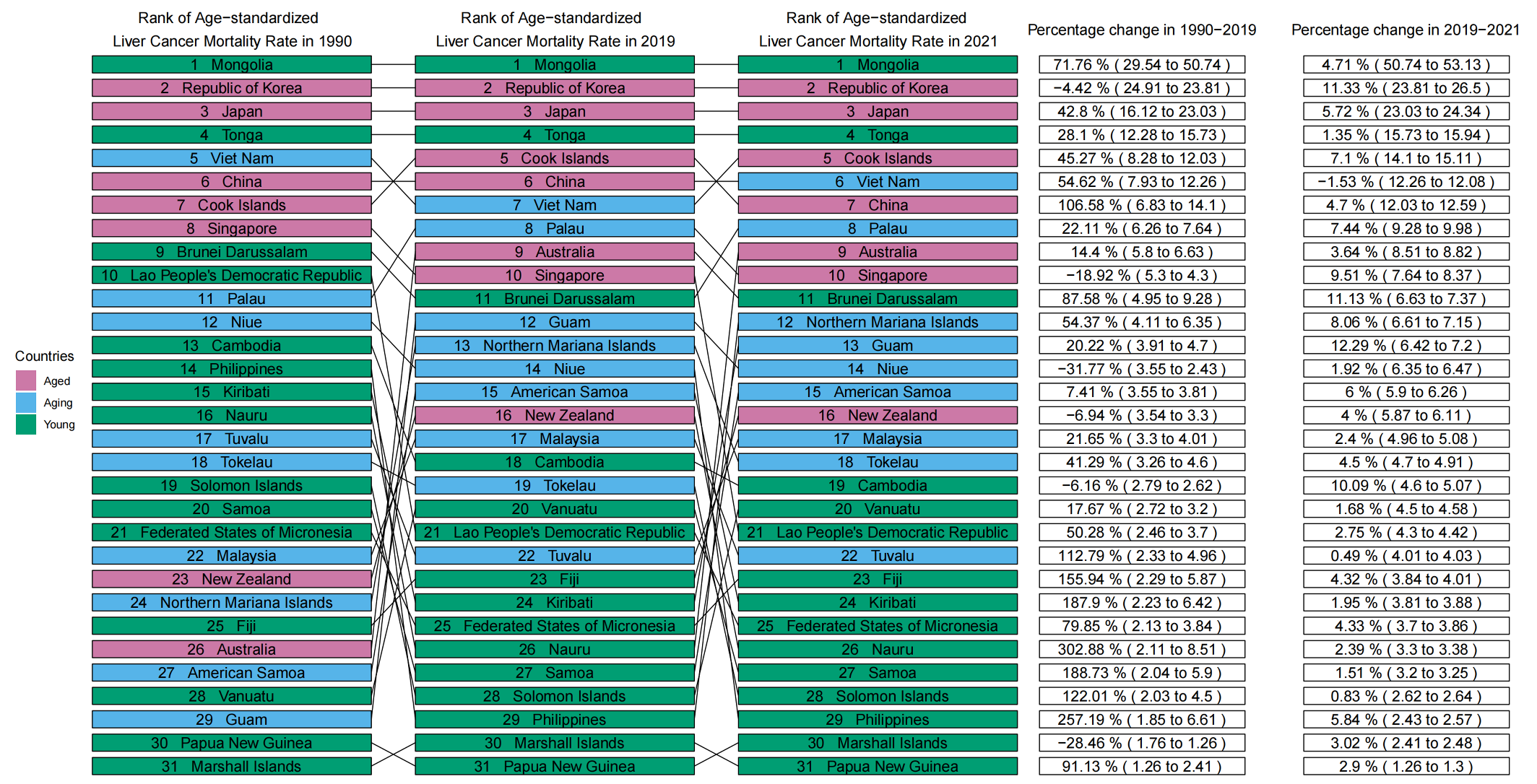


LC = liver cancer; WPR = western pacific region

Figure S7. Cases of DALYs (A), incidence (B), prevalence (C) and mortality (D) rate of 5 specific LC in the WPR by years, from 1990 to 2021


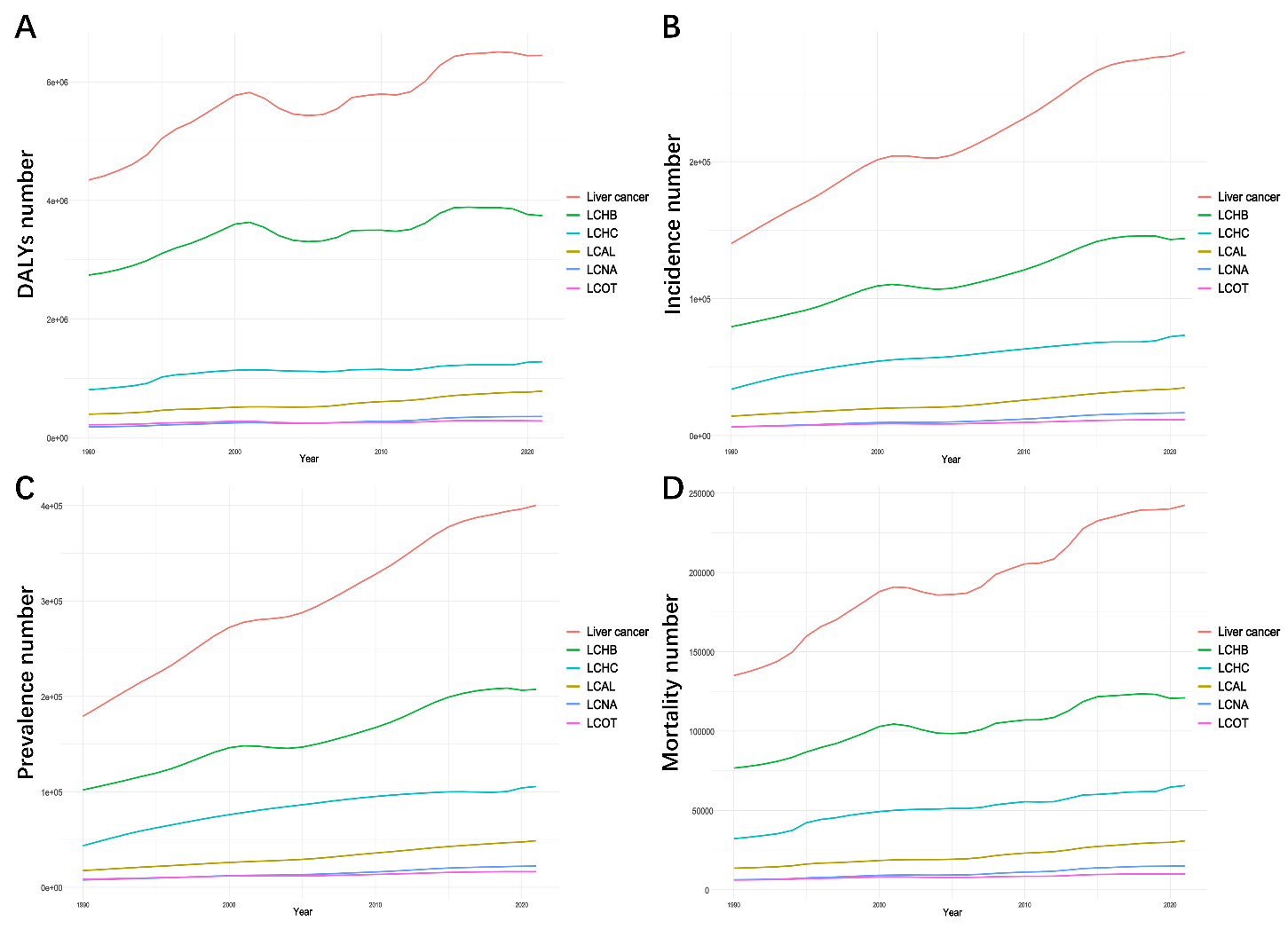


DALYs = disability-adjusted life years; LC = liver cancer; WPR = western pacific region; LCHB = liver cancer due to hepatitis B; LCHC = liver cancer due to hepatitis C; LCAL = liver cancer due to alcohol; LCNA = liver cancer due to nonalcoholic steatohepatitis (NASH); LCOT = liver cancer due to other cause

Figure S8. Trends in age-standardized DALYs, incidence, prevalence, and mortality rate of LCHB by sex from 1990 to 2021. (A) Age-standardized DALYs rate. (B) Age-standardized incidence rate. (C) Age-standardized prevalence rate. (D) Age-standardized mortality rate


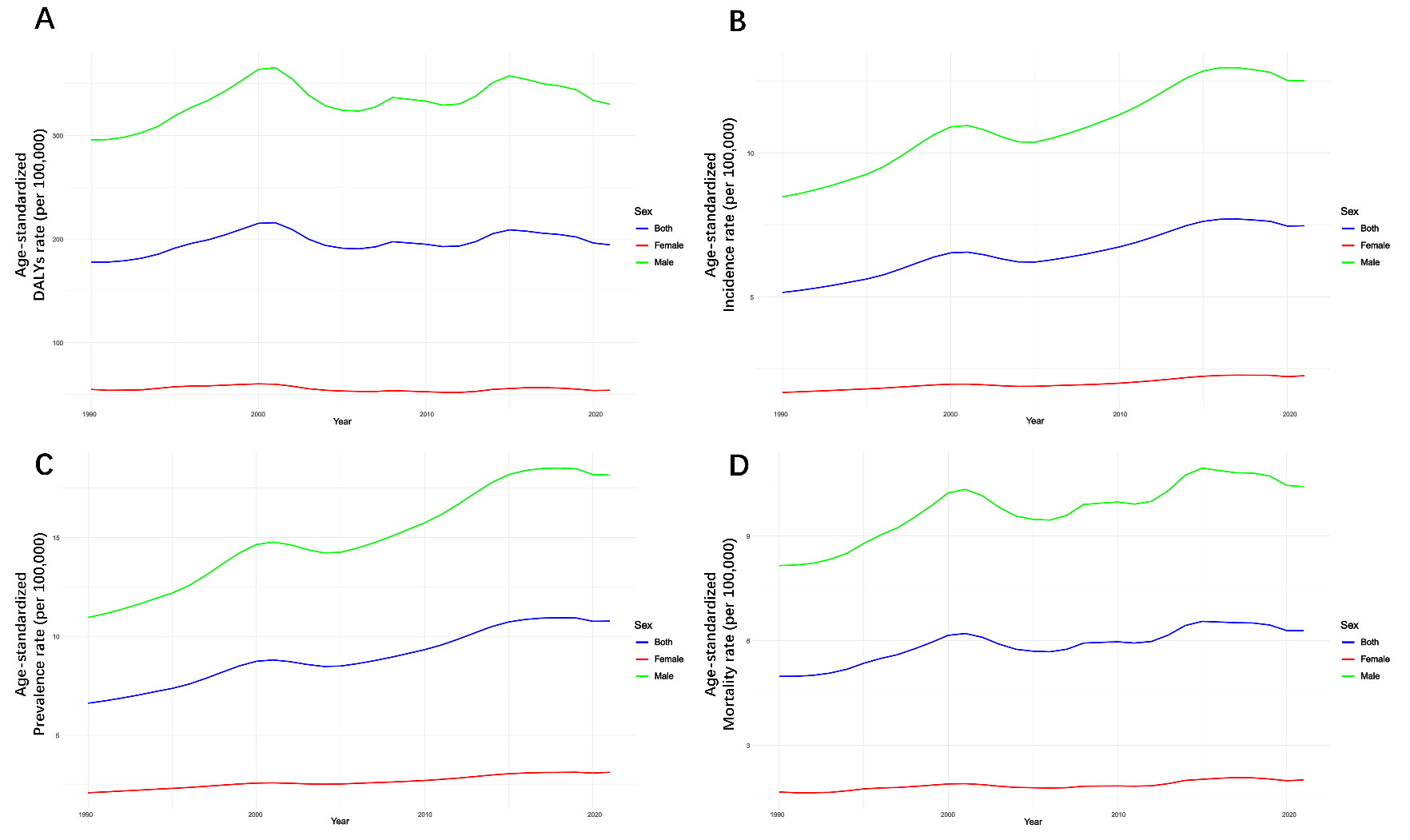


DALYs = disability-adjusted life years; LC = liver cancer; WPR = western pacific region; LCHB = liver cancer due to hepatitis B

Figure S9. Trends in age-standardized DALYs, incidence, prevalence, and mortality rate of LCHC by sex from 1990 to 2021. (A) Age-standardized DALYs rate. (B) Age-standardized incidence rate. (C) Age-standardized prevalence rate. (D) Age-standardized mortality rate


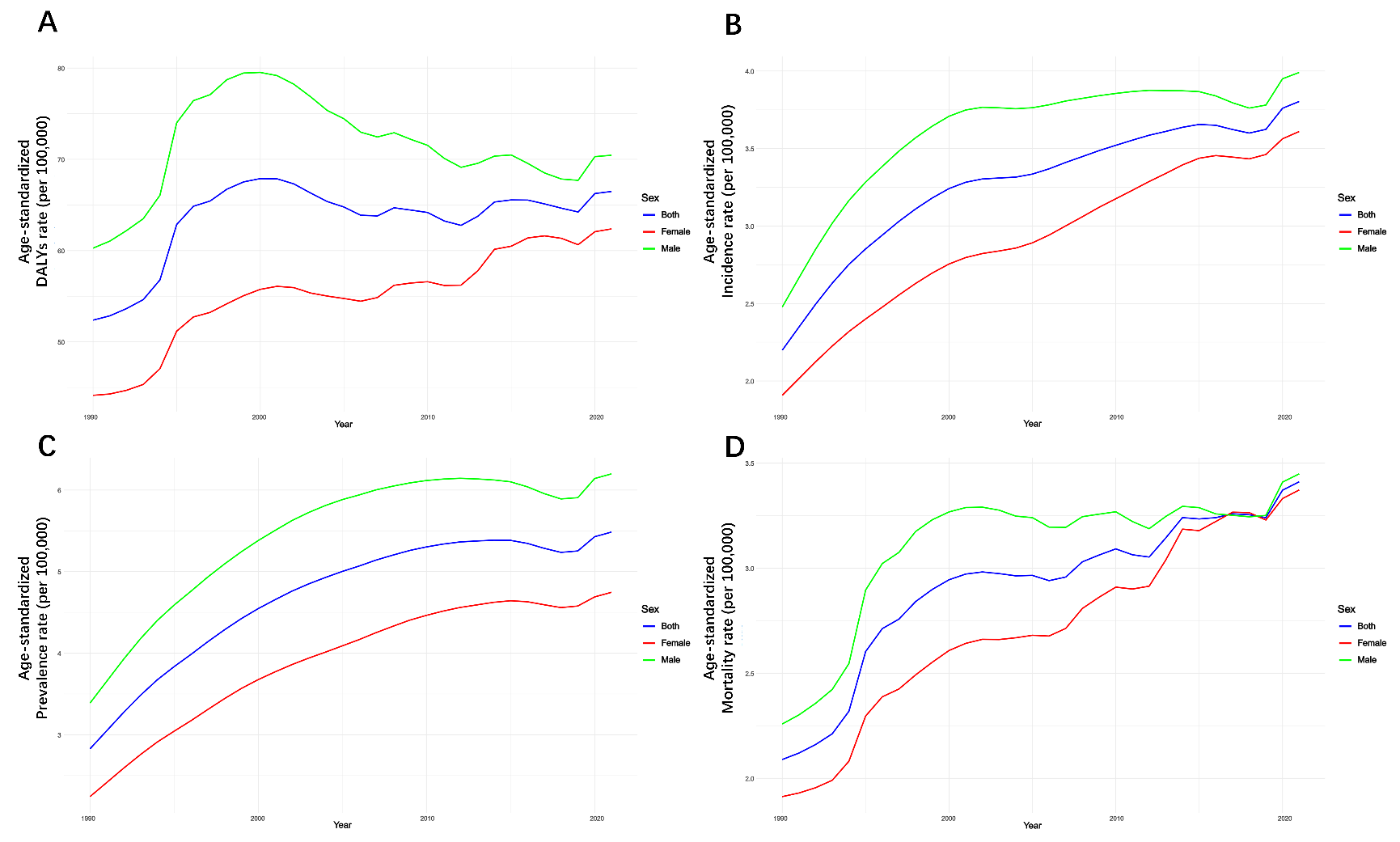


DALYs = disability-adjusted life years; LC = liver cancer; WPR = western pacific region; LCHC = liver cancer due to hepatitis C

Figure S10. Trends in age-standardized DALYs, incidence, prevalence, and mortality rate of LCAL by sex from 1990 to 2021. (A) Age-standardized DALYs rate. (B) Age-standardized incidence rate. (C) Age-standardized prevalence rate. (D) Age-standardized mortality rate


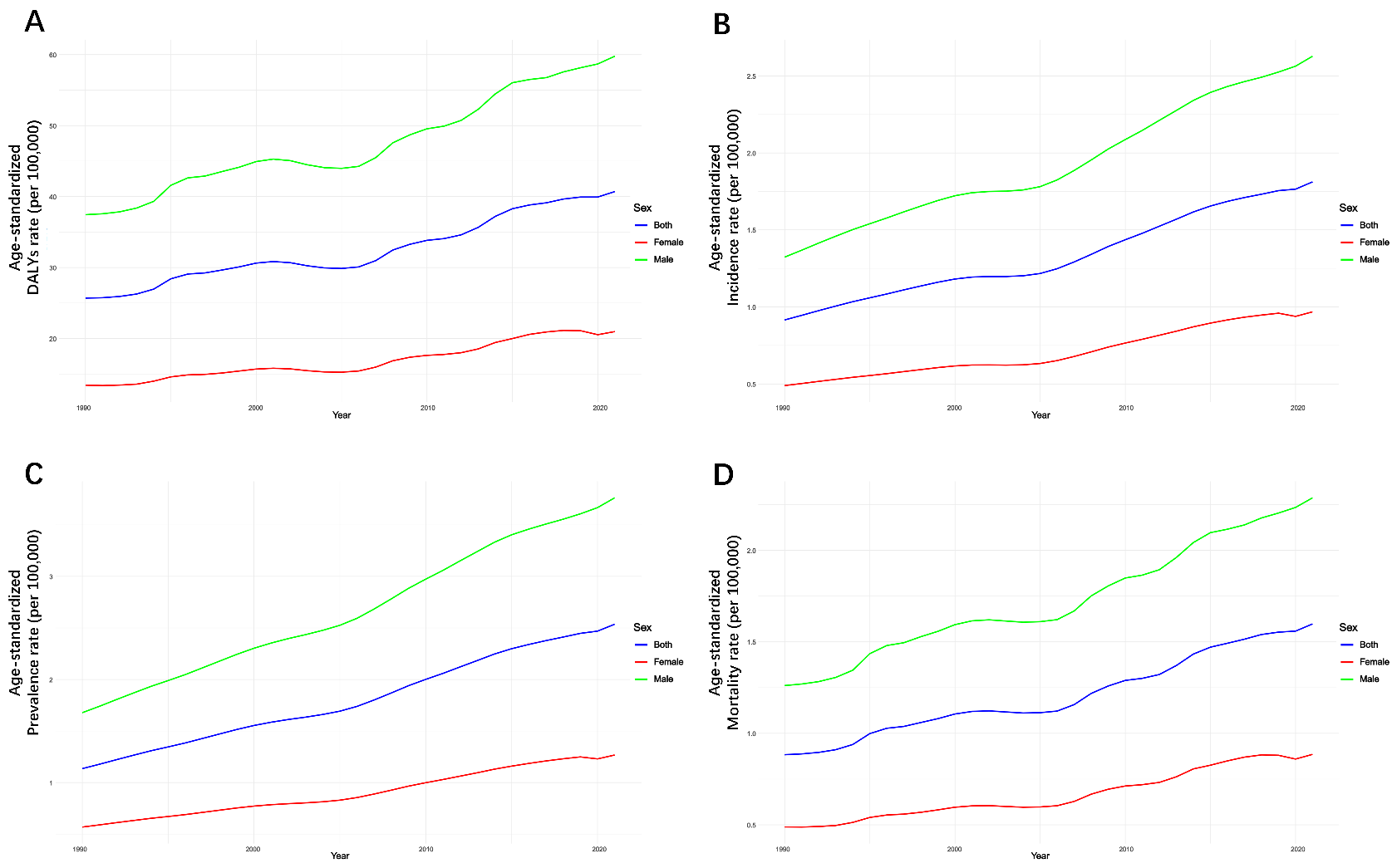


DALYs = disability-adjusted life years; LC = liver cancer; WPR = western pacific region; LCAL = liver cancer due to alcohol

Figure S11. Trends in age-standardized DALYs, incidence, prevalence, and mortality rate of LCNA by sex from 1990 to 2021. (A) Age-standardized DALYs rate. (B) Age-standardized incidence rate. (C) Age-standardized prevalence rate. (D) Age-standardized mortality rate


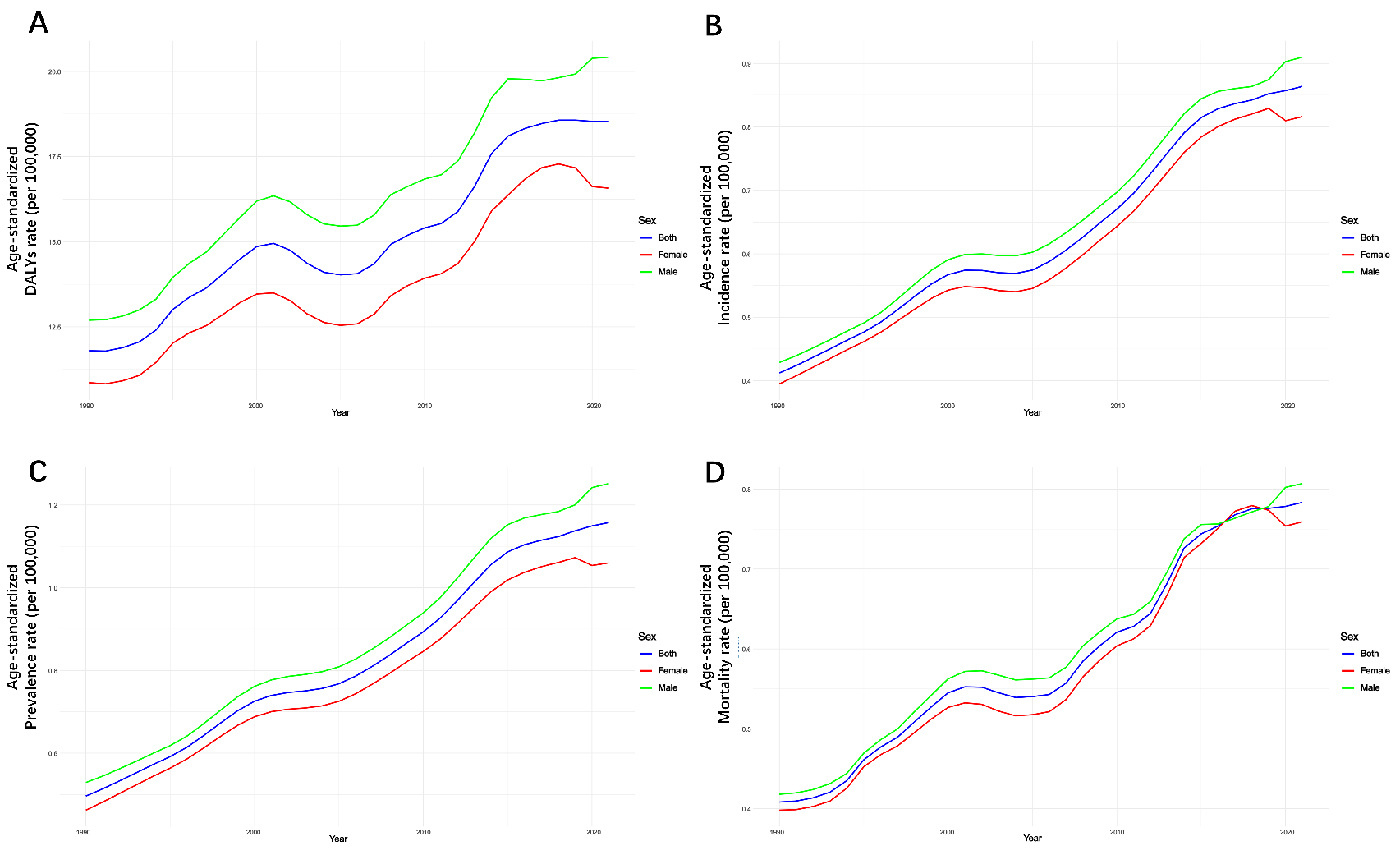


DALYs = disability-adjusted life years; LC = liver cancer; WPR = western pacific region; LCNA = liver cancer due to nonalcoholic steatohepatitis (NASH)

Figure S12. Trends in age-standardized DALYs, incidence, prevalence, and mortality rate of LCOT by sex from 1990 to 2021. (A) Age-standardized DALYs rate. (B) Age-standardized incidence rate. (C) Age-standardized prevalence rate. (D) Age-standardized mortality rate


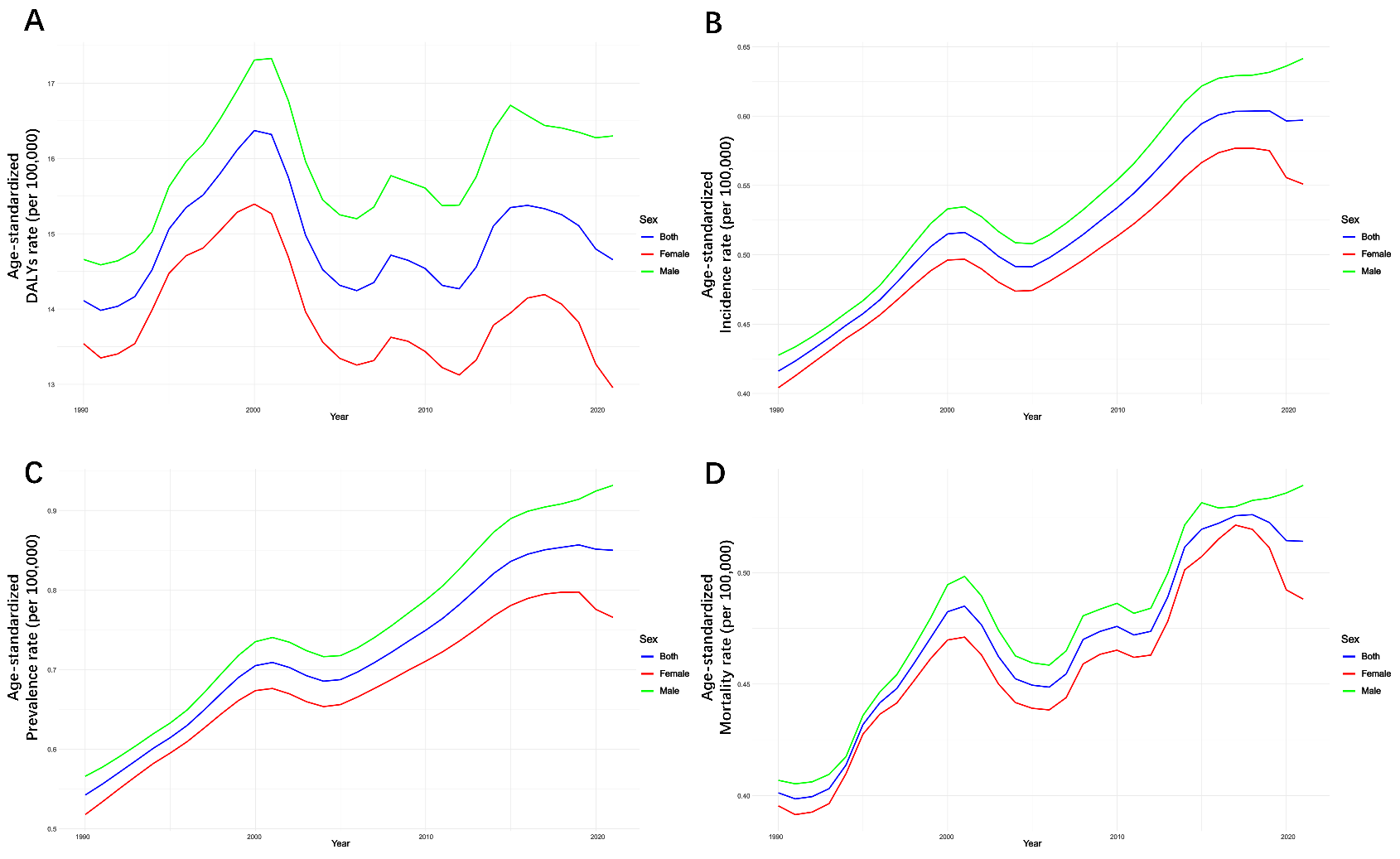


DALYs = disability-adjusted life years; LC = liver cancer; WPR = western pacific region; LCOT = liver cancer due to other cause

Figure S13. Age-standardized DALYs (A), incidence (B), prevalence (C) and mortality (D) rate of 5 specific LC in the WPR by age in 2021


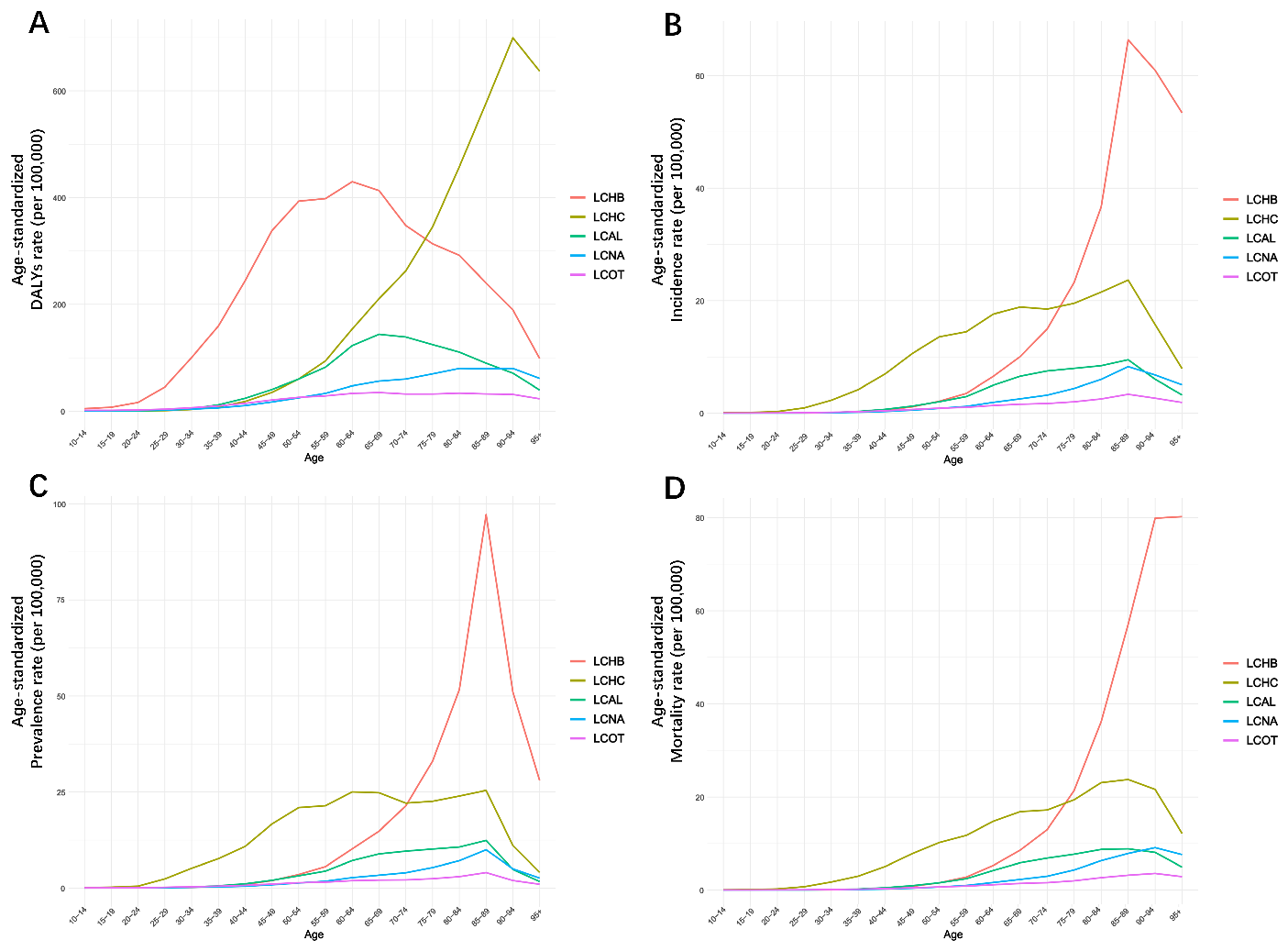


DALYs = disability-adjusted life years; LC = liver cancer; WPR = western pacific region; LCHB = liver cancer due to hepatitis B; LCHC = liver cancer due to hepatitis C; LCAL = liver cancer due to alcohol; LCNA = liver cancer due to nonalcoholic steatohepatitis (NASH); LCOT = liver cancer due to other cause

Figure S14. Associations of age-standardized incidence rate of total (A), LCHB (B), LCHC (C), LCAL (D), LCNA (E), LCOT (F) with human resource for health density in the WPR, in 2019


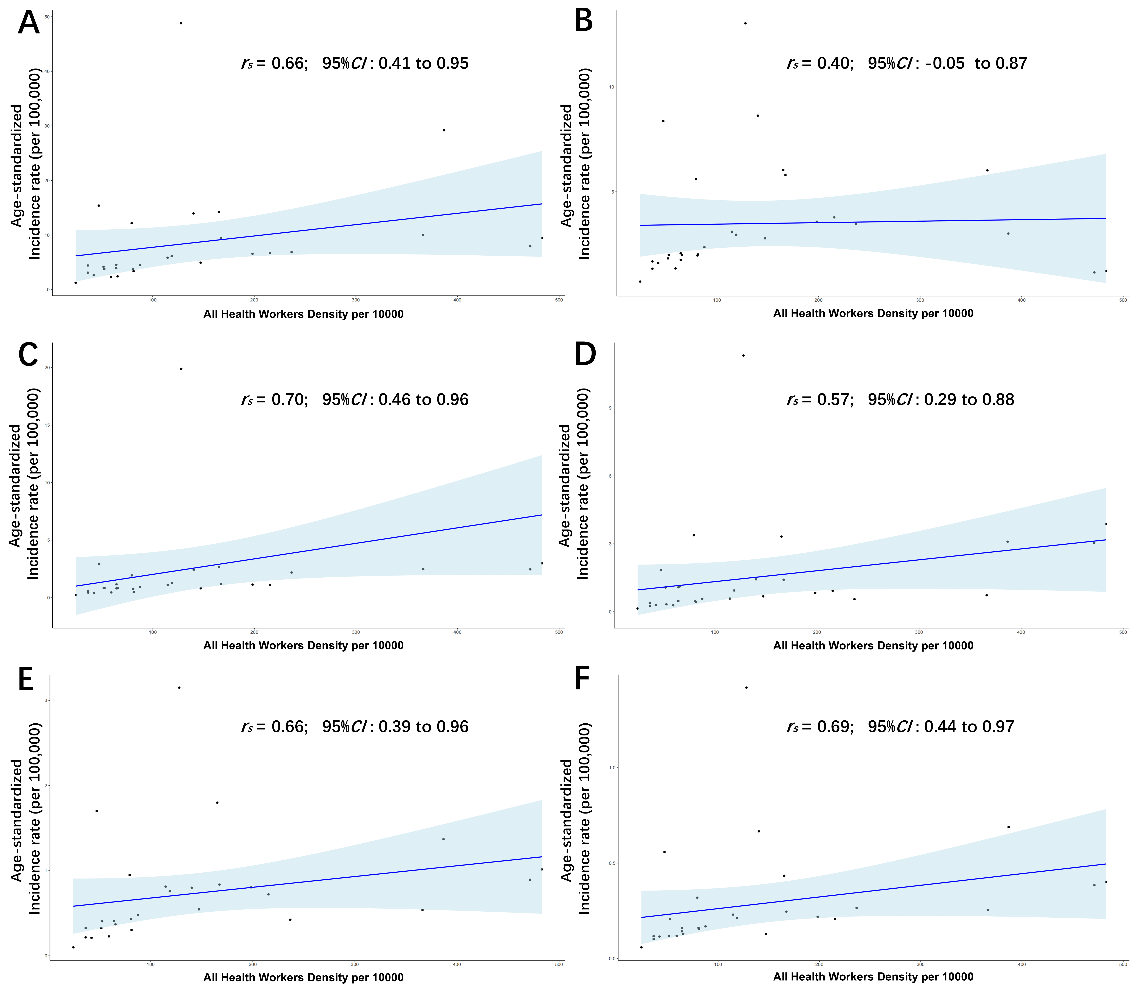


LCHB = liver cancer due to hepatitis B; LCHC = liver cancer due to hepatitis C; LCAL = liver cancer due to alcohol; LCNA = liver cancer due to nonalcoholic steatohepatitis (NASH); LCOT = liver cancer due to other cause; WPR = western pacific region

Figure S15. Associations of age-standardized prevalence rate of total (A), LCHB (B), LCHC (C), LCAL (D), LCNA (E), LCOT (F) with human resource for health density in the WPR, in 2019


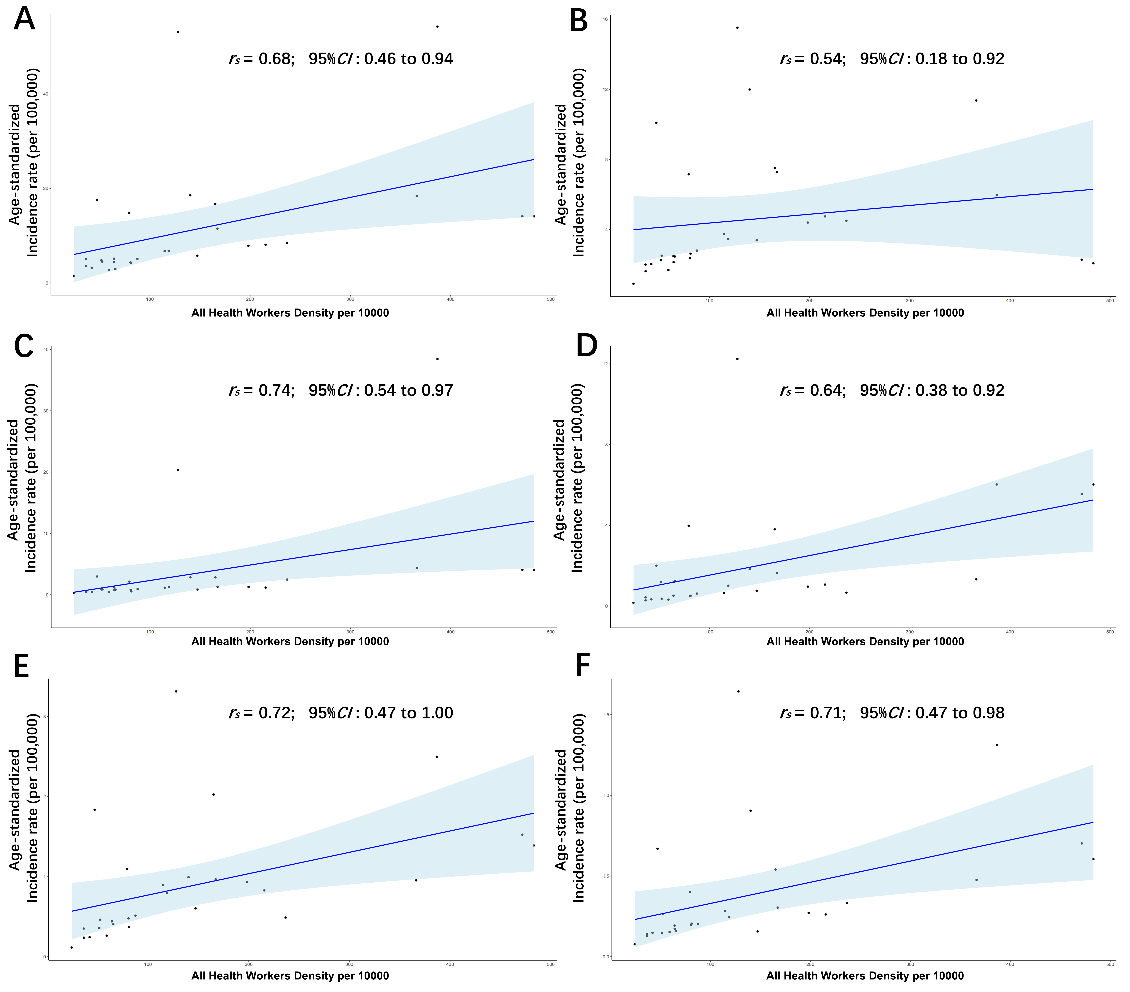


LCHB = liver cancer due to hepatitis B; LCHC = liver cancer due to hepatitis C; LCAL = liver cancer due to alcohol; LCNA = liver cancer due to nonalcoholic steatohepatitis (NASH); LCOT = liver cancer due to other cause; WPR = western pacific region

Figure S16. Associations of age-standardized mortality rate of total (A), LCHB (B), LCHC (C), LCAL (D), LCNA (E), LCOT (F) with human resource for health density in the WPR, in 2019


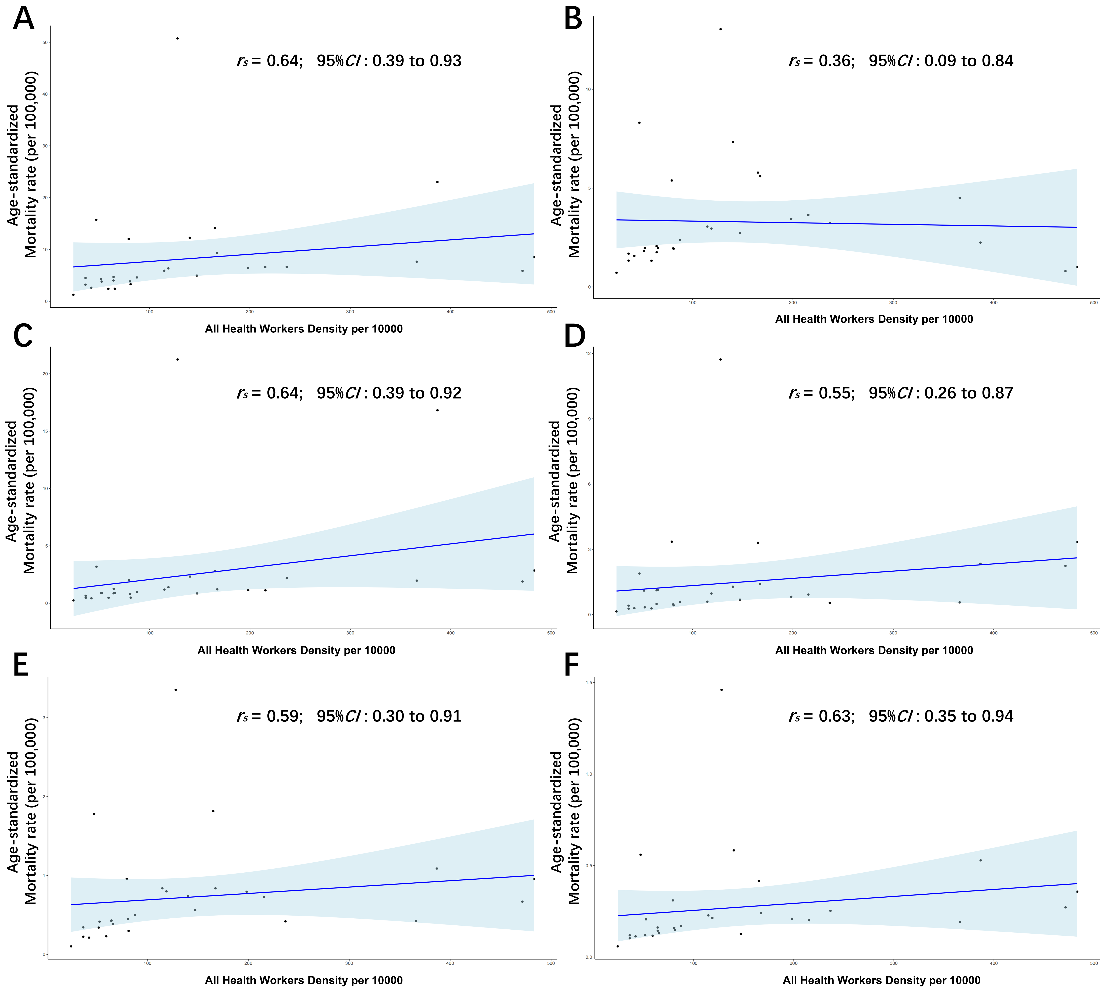


LCHB = liver cancer due to hepatitis B; LCHC = liver cancer due to hepatitis C; LCAL = liver cancer due to alcohol; LCNA = liver cancer due to nonalcoholic steatohepatitis (NASH); LCOT = liver cancer due to other cause; WPR = western pacific region

Figure S17. Associations of age-standardized DALYs rate of total (A), LCHB (B), LCHC (C), LCAL (D), LCNA (E), LCOT (F) with socio-demographic index in the WPR, in 2021


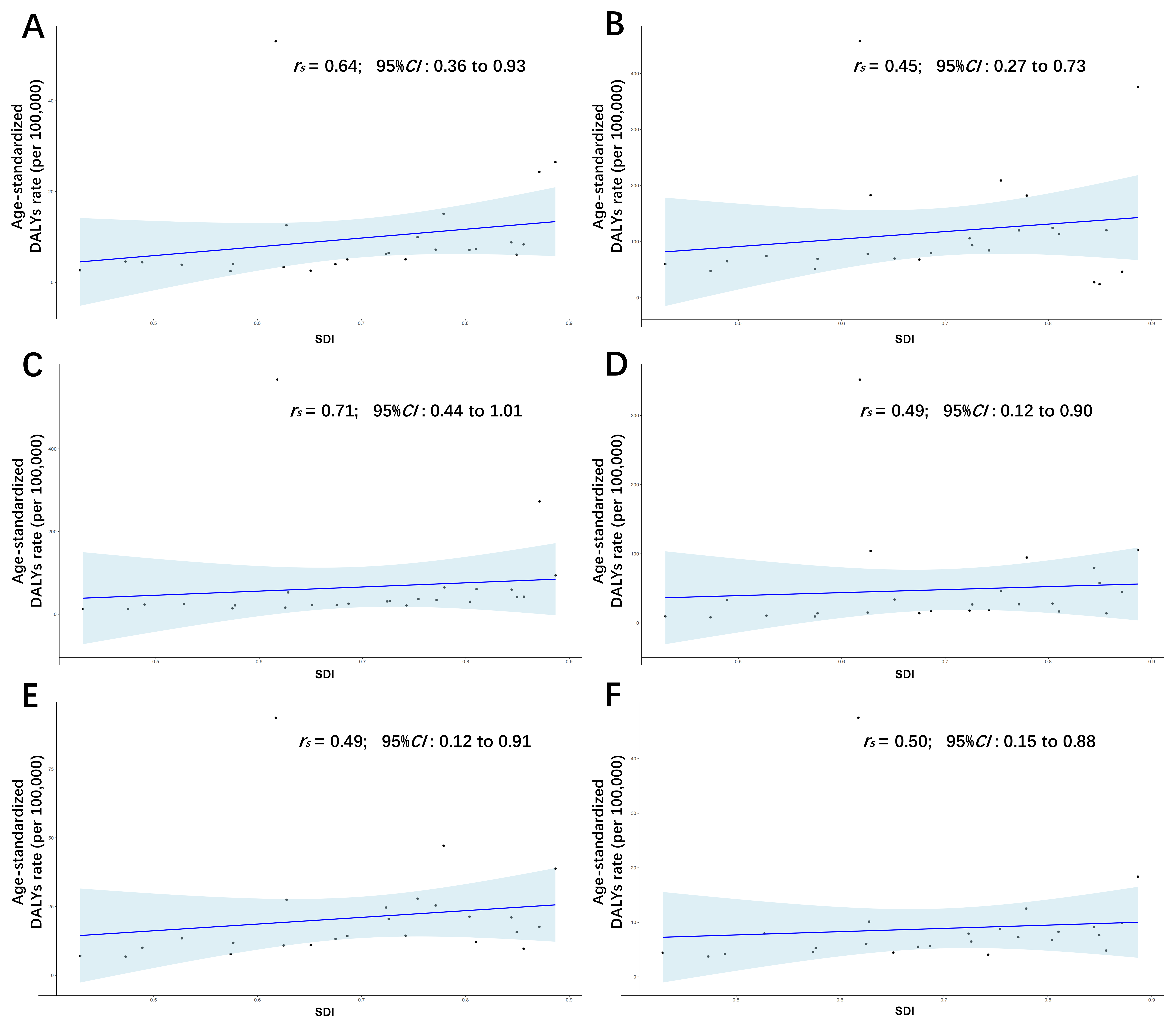


DALYs = disability-adjusted life years; LCHB = liver cancer due to hepatitis B; LCHC = liver cancer due to hepatitis C; LCAL = liver cancer due to alcohol; LCNA = liver cancer due to nonalcoholic steatohepatitis (NASH); LCOT = liver cancer due to other cause; UHC = universal health coverage; WPR = western pacific region

Figure S18. Associations of age-standardized incidence rate of total (A), LCHB (B), LCHC (C), LCAL (D), LCNA (E), LCOT (F) with socio-demographic index in the WPR, in 2021


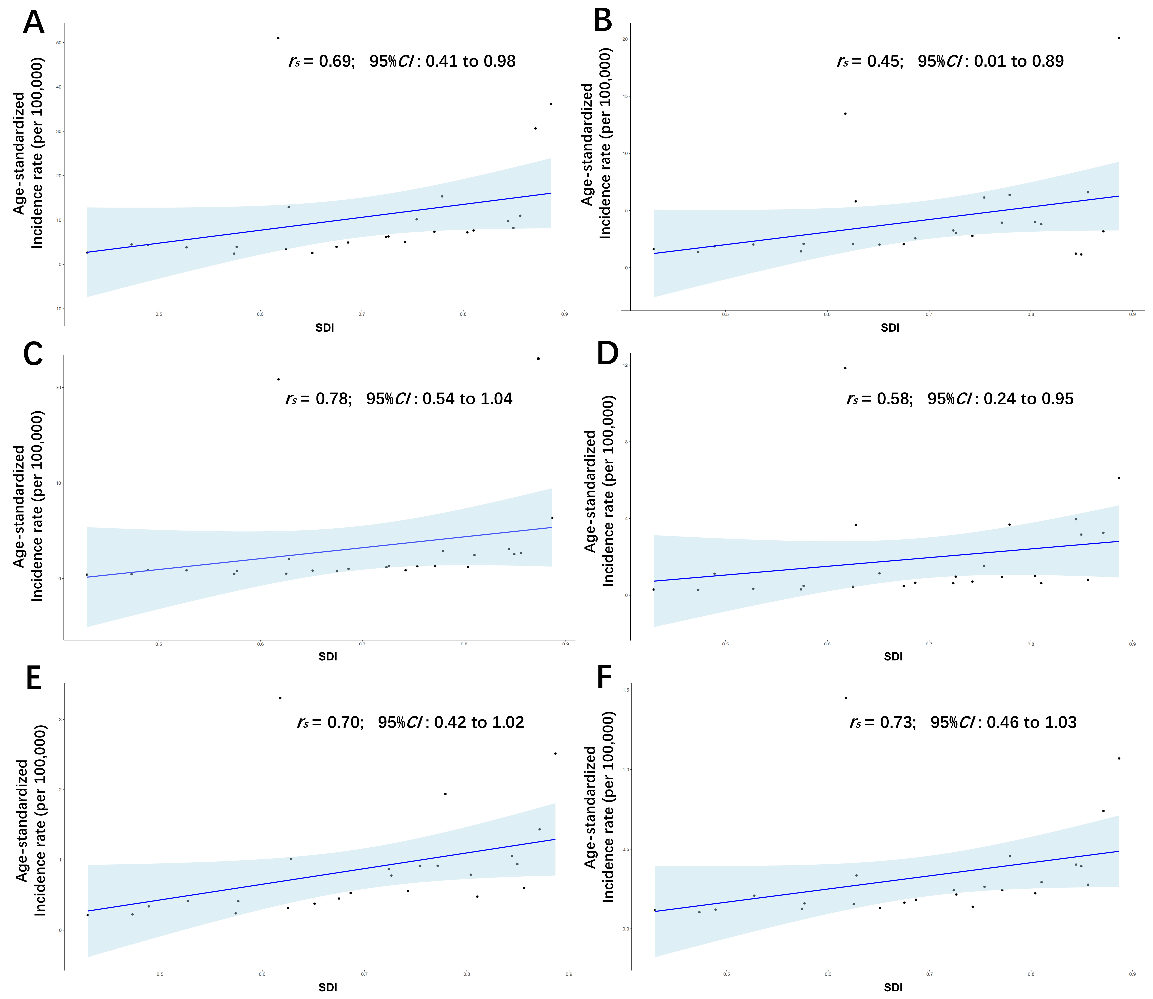


LCHB = liver cancer due to hepatitis B; LCHC = liver cancer due to hepatitis C; LCAL = liver cancer due to alcohol; LCNA = liver cancer due to nonalcoholic steatohepatitis (NASH); LCOT = liver cancer due to other cause; WPR = western pacific region; SDI = sociodemographic Index

Figure S19. Associations of age-standardized prevalence rate of total (A), LCHB (B), LCHC (C), LCAL (D), LCNA (E), LCOT (F) with socio-demographic index in the WPR, in 2021


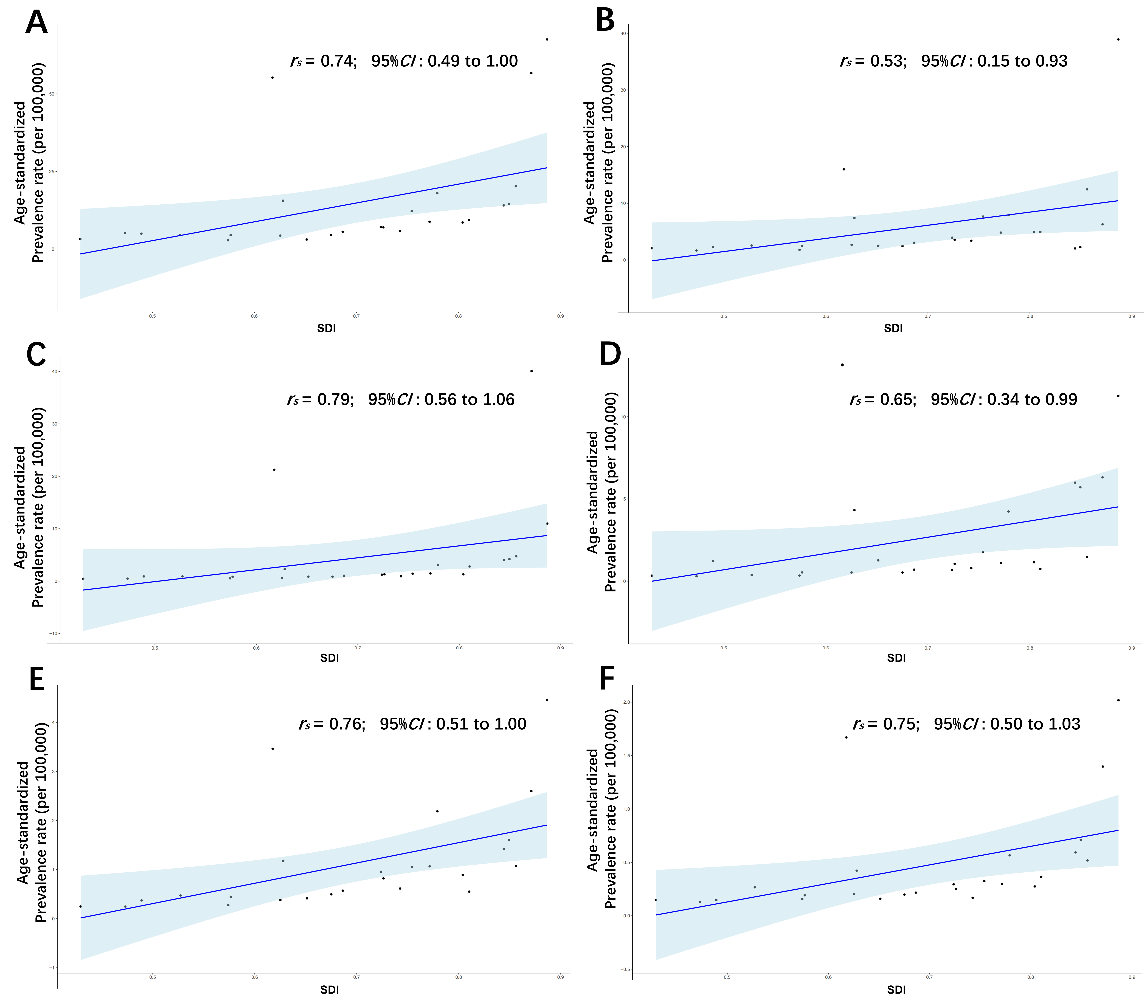


LCHB = liver cancer due to hepatitis B; LCHC = liver cancer due to hepatitis C; LCAL = liver cancer due to alcohol; LCNA = liver cancer due to nonalcoholic steatohepatitis (NASH); LCOT = liver cancer due to other cause; WPR = western pacific region; SDI = sociodemographic Index

Figure S20. Associations of age-standardized DALYs rate of total (A), LCHB (B), LCHC (C), LCAL (D), LCNA (E), LCOT (F) with UHC service coverage index in the WPR, in 2021


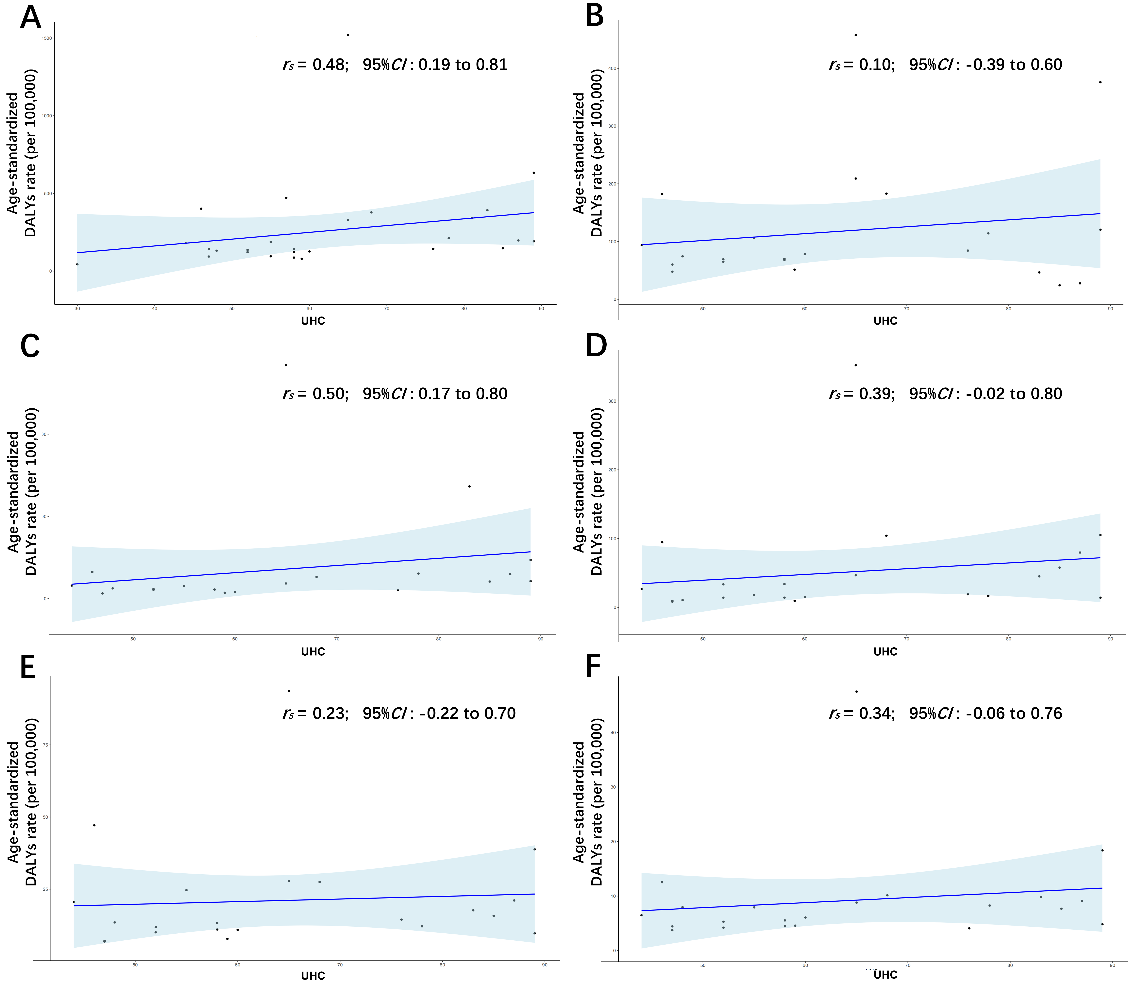


DALYs = disability-adjusted life years; LCHB = liver cancer due to hepatitis B; LCHC = liver cancer due to hepatitis C; LCAL = liver cancer due to alcohol; LCNA = liver cancer due to nonalcoholic steatohepatitis (NASH); LCOT = liver cancer due to other cause; UHC = universal health coverage; WPR = western pacific region

Figure S21. Associations of age-standardized mortality rate of total (A), LCHB (B), LCHC (C), LCAL (D), LCNA (E), LCOT (F) with socio-demographic index in the WPR, in 2021


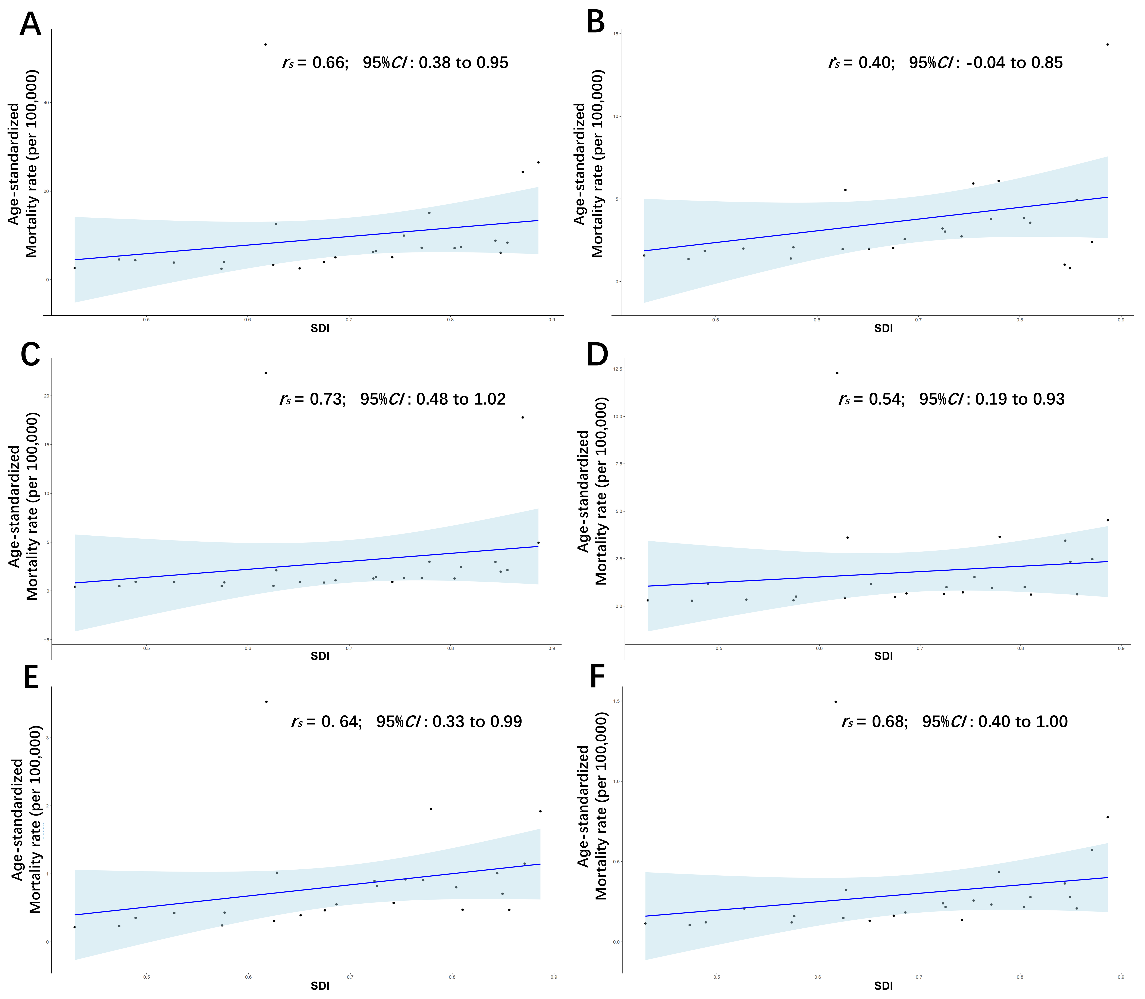


LCHB = liver cancer due to hepatitis B; LCHC = liver cancer due to hepatitis C; LCAL = liver cancer due to alcohol; LCNA = liver cancer due to nonalcoholic steatohepatitis (NASH); LCOT = liver cancer due to other cause; WPR = western pacific region; SDI = sociodemographic Index

Figure S22. Associations of age-standardized incidence rate of total (A), LCHB (B), LCHC (C), LCAL (D), LCNA (E), LCOT (F) with UHC service coverage index in the WPR, in 2021


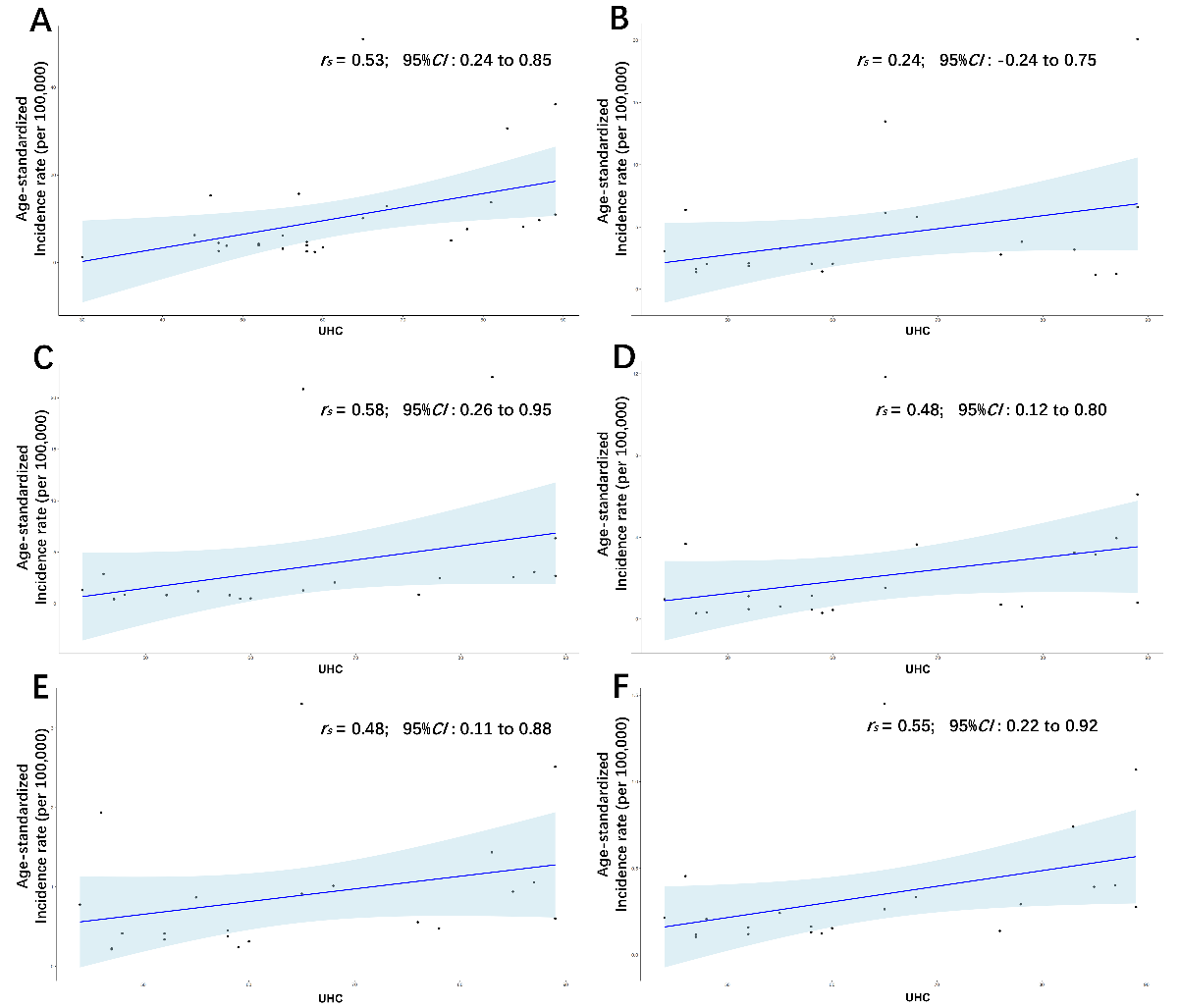


LCHB = liver cancer due to hepatitis B; LCHC = liver cancer due to hepatitis C; LCAL = liver cancer due to alcohol; LCNA = liver cancer due to nonalcoholic steatohepatitis (NASH); LCOT = liver cancer due to other cause; UHC = universal health coverage; WPR = western pacific region

Figure S23. Associations of age-standardized prevalence rate of total (A), LCHB (B), LCHC (C), LCAL (D), LCNA (E), LCOT (F) with UHC service coverage index in the WPR, in 2021


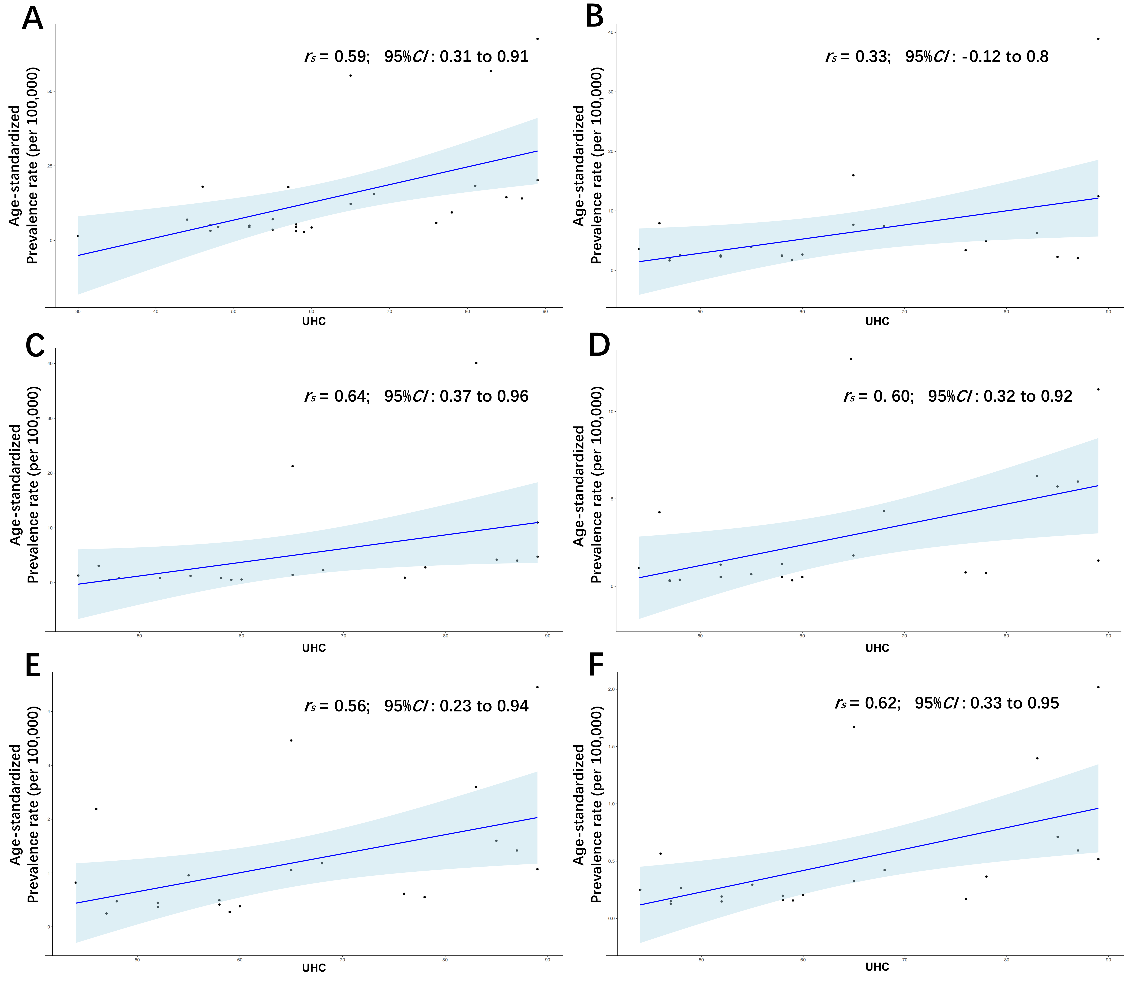


LCHB = liver cancer due to hepatitis B; LCHC = liver cancer due to hepatitis C; LCAL = liver cancer due to alcohol; LCNA = liver cancer due to nonalcoholic steatohepatitis (NASH); LCOT = liver cancer due to other cause; UHC = universal health coverage; WPR = western pacific region

Figure S24. Associations of age-standardized mortality rate of total (A), LCHB (B), LCHC (C), LCAL (D), LCNA (E), LCOT (F) with UHC service coverage index in the WPR, in 2021


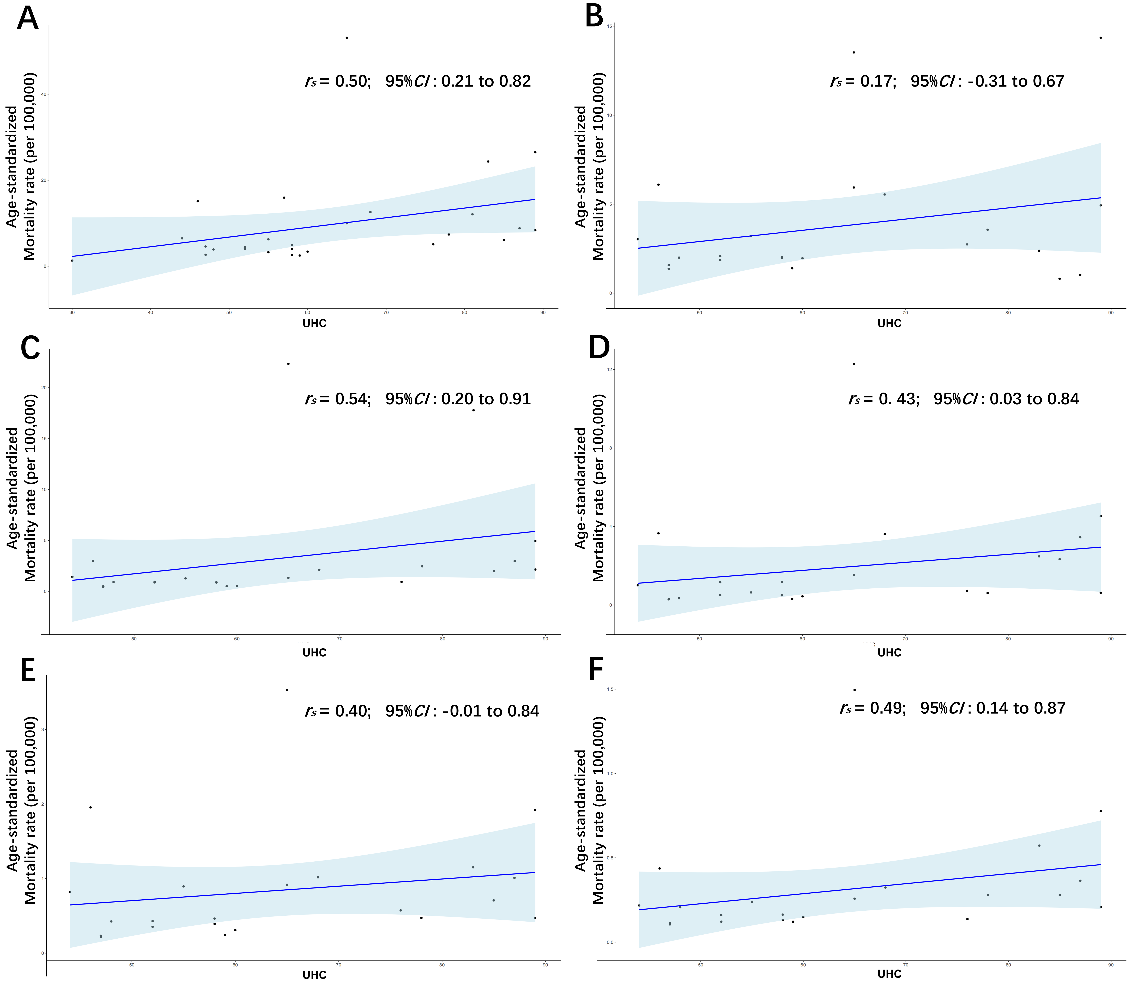


LCHB = liver cancer due to hepatitis B; LCHC = liver cancer due to hepatitis C; LCAL = liver cancer due to alcohol; LCNA = liver cancer due to nonalcoholic steatohepatitis (NASH); LCOT = liver cancer due to other cause; UHC = universal health coverage; WPR = western pacific region

Table S1. Age-standardized liver cancer DALYs rates (per 100,000) in the Western Pacific region by member state and sex, in 1990, 2019, and 2021.

| **Location** |  | **Age-standardized DALYs rate, per 100, 000 (95% UI)** | | | | | |  | **EAPC, % (95%CI)** | |
| --- | --- | --- | --- | --- | --- | --- | --- | --- | --- | --- |
|  |  | **1990** | **Rank** | **2019** | **Rank** | **2021** | **Rank** |  | **1990-2019** | **1990-2021** |
| **Male** |  |  |  |  |  |  |  |  |  |  |
| American Samoa |  | 82.25 (60.42, 104.09) | 28 | 218 (166.14, 269.87) | 15 | 228.43 (172.34, 284.51) | 15 |  | 3.42 (1.63, 5.3) | 3.35(1.64, 5.13) |
| Australia |  | 80.39 (68.33, 92.45) | 29 | 272.7 (231.15, 314.25) | 10 | 280.04 (236.66, 323.41) | 13 |  | 4.3 (3.21, 5.4) | 4.11(3.08, 5.14) |
| Brunei Darussalam |  | 233.76 (174.49, 293.03) | 11 | 257.31 (199.55, 315.06) | 13 | 281.02 (216.49, 345.55) | 12 |  | 0.33 (-1.32, 2.06) | 0.6(-0.97, 2.23) |
| Cambodia |  | 133.19 (54.42, 211.96) | 16 | 152.82 (61.38, 244.27) | 25 | 159.24 (63.53, 254.96) | 24 |  | 0.48 (-4.18, 5.31) | 0.58(-3.81, 5.11) |
| China |  | 393.4 (327.19, 459.61) | 6 | 525.41 (406.74, 644.08) | 8 | 506.96 (390.7, 623.23) | 8 |  | 1 (-0.42, 2.36) | 0.82(-0.52, 2.1) |
| Cook Islands |  | 256.83 (191.89, 321.77) | 9 | 577.81 (444.85, 710.76) | 5 | 614.14 (466.24, 762.05) | 4 |  | 2.84 (1.12, 4.62) | 2.85(1.2, 4.55) |
| Federated States of Micronesia |  | 101.01 (59.18, 142.84) | 21 | 158.54 (83.3, 233.77) | 22 | 166.05 (85.86, 246.23) | 22 |  | 1.57 (-1.84, 4.85) | 1.62(-1.63, 4.71) |
| Fiji |  | 91.01 (63.69, 118.33) | 24 | 154.37 (109.86, 198.87) | 24 | 160.24 (111.91, 208.57) | 23 |  | 1.84 (-0.26, 4) | 1.84(-0.18, 3.9) |
| Guam |  | 82.93 (69.62, 96.24) | 27 | 326.73 (273.1, 380.35) | 9 | 342.78 (282.29, 403.26) | 9 |  | 4.84 (3.66, 6.03) | 4.68(3.53, 5.83) |
| Japan |  | 672.17 (635.36, 708.98) | 3 | 546.22 (513.87, 578.56) | 7 | 564.21 (527.77, 600.65) | 7 |  | -0.71 (-1.1, -0.32) | -0.56(-0.95, -0.18) |
| Kiribati |  | 119.47 (88.26, 150.68) | 18 | 120.32 (75, 165.64) | 28 | 122.08 (77.16, 167.01) | 28 |  | 0.02 (-2.38, 2.19) | 0.07(-2.14, 2.08) |
| Laos |  | 246.67 (174.64, 318.7) | 10 | 189.72 (134.67, 244.78) | 19 | 195.2 (139.1, 251.3) | 19 |  | -0.9 (-2.93, 1.17) | -0.75(-2.64, 1.18) |
| Malaysia |  | 97.73 (76.28, 119.19) | 22 | 211.42 (166.68, 256.16) | 16 | 214.03 (166.49, 261.57) | 16 |  | 2.7 (1.16, 4.27) | 2.56(1.08, 4.06) |
| Marshall Islands |  | 44.75 (21.93, 67.56) | 31 | 96.31 (41.52, 151.1) | 30 | 101.62 (41.79, 161.44) | 30 |  | 2.68 (-1.66, 6.88) | 2.68(-1.54, 6.65) |
| Mongolia |  | 1122.68 (827.31, 1418.05) | 2 | 1753.95 (1347.95, 2159.96) | 1 | 1813.89 (1387.59, 2240.19) | 1 |  | 1.55 (-0.17, 3.36) | 1.56(-0.07, 3.27) |
| Nauru |  | 187.26 (135.75, 238.76) | 13 | 177.46 (107.36, 247.57) | 20 | 180.1 (105.16, 255.04) | 21 |  | -0.19 (-2.72, 2.09) | -0.13(-2.61, 2.06) |
| New Zealand |  | 87.94 (79.66, 96.23) | 26 | 200.73 (181.2, 220.26) | 18 | 205.37 (185.22, 225.53) | 18 |  | 2.89 (2.21, 3.57) | 2.77(2.13, 3.41) |
| Niue |  | 147.25 (100.9, 193.6) | 14 | 256.3 (162.91, 349.68) | 14 | 264.28 (167.39, 361.16) | 14 |  | 1.93 (-0.59, 4.38) | 1.9(-0.47, 4.2) |
| Northern Mariana Islands |  | 104.5 (71.65, 137.35) | 20 | 271.12 (206.25, 335.99) | 11 | 297.3 (233.68, 360.92) | 10 |  | 3.34 (1.41, 5.47) | 3.43(1.73, 5.35) |
| Palau |  | 312.01 (207.17, 416.86) | 7 | 566.07 (430.16, 701.99) | 6 | 597.52 (442.85, 752.18) | 6 |  | 2.08 (0.11, 4.3) | 2.12(0.2, 4.25) |
| Papua New Guinea |  | 74.21 (14.49, 133.94) | 30 | 53.79 (4.25, 103.33) | 31 | 55.28 (2.83, 107.72) | 31 |  | -1.1 (-11.22, 7.01) | -0.95(-11.7, 6.69) |
| Philippines |  | 197.68 (160.09, 235.27) | 12 | 207.07 (183.32, 230.82) | 17 | 210.19 (174.83, 245.55) | 17 |  | 0.16 (-0.86, 1.27) | 0.2(-0.95, 1.39) |
| Samoa |  | 117.31 (88.33, 146.29) | 19 | 134.71 (100.32, 169.11) | 26 | 137.64 (100.66, 174.62) | 26 |  | 0.48 (-1.29, 2.26) | 0.52(-1.2, 2.22) |
| Singapore |  | 271.14 (238.7, 303.58) | 8 | 261.33 (221.47, 301.2) | 12 | 283.38 (240.13, 326.64) | 11 |  | -0.13 (-1.08, 0.81) | 0.14(-0.75, 1.02) |
| Solomon Islands |  | 145.52 (43.54, 247.5) | 15 | 134.34 (78.3, 190.38) | 27 | 136.05 (80.51, 191.6) | 27 |  | -0.28 (-3.89, 5.22) | -0.22(-3.56, 4.9) |
| South Korea |  | 1177.96 (877, 1478.93) | 1 | 897.86 (748.96, 1046.77) | 2 | 986.03 (798.1, 1173.95) | 2 |  | -0.93 (-2.32, 0.61) | -0.57(-1.97, 0.95) |
| Tokelau |  | 90.11 (46.8, 133.42) | 25 | 170.86 (106.46, 235.26) | 21 | 184.89 (115.81, 253.98) | 20 |  | 2.23 (-0.78, 5.73) | 2.35(-0.46, 5.61) |
| Tonga |  | 560.41 (409.25, 711.56) | 4 | 736.45 (527.35, 945.56) | 3 | 748.23 (524.57, 971.89) | 3 |  | 0.95 (-1.03, 2.93) | 0.94(-0.98, 2.83) |
| Tuvalu |  | 123.22 (79.35, 167.09) | 17 | 155.05 (98.98, 211.11) | 23 | 156.49 (100.64, 212.33) | 25 |  | 0.8 (-1.79, 3.43) | 0.77(-1.62, 3.23) |
| Vanuatu |  | 92.26 (48.24, 136.28) | 23 | 104.75 (64, 145.49) | 29 | 107.93 (64.72, 151.15) | 29 |  | 0.44 (-2.57, 3.88) | 0.51(-2.37, 3.75) |
| Viet Nam |  | 412.07 (307.78, 516.36) | 5 | 584.82 (418.64, 751) | 4 | 611.69 (426.72, 796.66) | 5 |  | 1.21 (-0.72, 3.12) | 1.28(-0.61, 3.12) |
| Western Pacific Region |  | 420.57 (365.63, 475.51) |  | 505.95 (414.12, 597.78) |  | 496.91 (407.07, 586.76) |  |  | 0.64 (-0.48, 1.71) | 0.54(-0.5, 1.54) |
| **Female** |  |  |  |  |  |  |  |  |  |  |
| American Samoa |  | 49.89 (37.81, 61.96) | 20 | 137.41 (108.07, 166.75) | 8 | 144.59 (111.85, 177.34) | 7 |  | 3.56 (1.94, 5.25) | 3.49(1.92, 5.11) |
| Australia |  | 28.2 (24.16, 32.24) | 30 | 114.09 (97.01, 131.16) | 12 | 115.8 (97.97, 133.62) | 13 |  | 4.94 (3.87, 6.01) | 4.66(3.65, 5.67) |
| Brunei Darussalam |  | 101.21 (75.72, 126.71) | 9 | 122.57 (93.16, 151.98) | 10 | 134.93 (99.45, 170.41) | 10 |  | 0.66 (-1.06, 2.43) | 0.93(-0.78, 2.65) |
| Cambodia |  | 112.07 (62.38, 161.76) | 8 | 121.88 (79.97, 163.79) | 11 | 126.25 (83.34, 169.16) | 11 |  | 0.29 (-2.4, 3.38) | 0.39(-2.12, 3.27) |
| China |  | 134.97 (118.28, 151.67) | 6 | 173.59 (149.83, 197.36) | 6 | 169.85 (144.47, 195.22) | 6 |  | 0.87 (-0.04, 1.78) | 0.74(-0.16, 1.63) |
| Cook Islands |  | 145.33 (115.05, 175.6) | 5 | 190.54 (153.42, 227.67) | 5 | 200.54 (158.77, 242.32) | 4 |  | 0.94 (-0.46, 2.38) | 1.04(-0.32, 2.43) |
| Federated States of Micronesia |  | 52.24 (35.78, 68.7) | 19 | 81.57 (64.37, 98.76) | 20 | 84.52 (65.75, 103.28) | 19 |  | 1.55 (-0.22, 3.56) | 1.56(-0.14, 3.48) |
| Fiji |  | 31.78 (26.59, 36.96) | 29 | 70.16 (58.65, 81.67) | 22 | 75.54 (61.86, 89.23) | 22 |  | 2.77 (1.6, 3.95) | 2.83(1.68, 3.98) |
| Guam |  | 177.56 (167.72, 187.41) | 4 | 218.39 (188.83, 247.95) | 3 | 228.28 (196.67, 259.9) | 3 |  | 0.72 (0.03, 1.36) | 0.81(0.16, 1.42) |
| Japan |  | 121.85 (95.24, 148.46) | 7 | 138.36 (104.67, 172.04) | 7 | 140.2 (105.46, 174.94) | 9 |  | 0.44 (-1.2, 2.06) | 0.45(-1.1, 1.98) |
| Kiribati |  | 90.62 (69.2, 112.04) | 11 | 74.11 (57.24, 90.98) | 21 | 76.11 (58.07, 94.14) | 21 |  | -0.69 (-2.29, 0.95) | -0.56(-2.1, 1) |
| Laos |  | 39.58 (32.32, 46.85) | 24 | 65.88 (53.86, 77.9) | 26 | 67.05 (54.22, 79.88) | 26 |  | 1.77 (0.48, 3.08) | 1.71(0.47, 2.96) |
| Malaysia |  | 38.15 (24.21, 52.09) | 26 | 69.81 (49.63, 89.99) | 24 | 72.35 (50.88, 93.82) | 23 |  | 2.11 (-0.17, 4.63) | 2.09(-0.08, 4.47) |
| Marshall Islands |  | 59.59 (44.68, 74.5) | 17 | 85.15 (58.91, 111.39) | 18 | 87.88 (60.02, 115.73) | 18 |  | 1.24 (-0.81, 3.2) | 1.26(-0.69, 3.12) |
| Mongolia |  | 632.04 (466.43, 797.66) | 1 | 1179.61 (951.94, 1407.29) | 1 | 1230.04 (975.18, 1484.89) | 1 |  | 2.17 (0.61, 3.88) | 2.17(0.65, 3.81) |
| Nauru |  | 67.17 (51.32, 83.01) | 16 | 70.09 (48.48, 91.7) | 23 | 71.28 (49.78, 92.77) | 24 |  | 0.15 (-1.84, 2.02) | 0.19(-1.64, 1.93) |
| New Zealand |  | 35.23 (32.46, 38) | 28 | 87.11 (79.97, 94.25) | 17 | 88.69 (81.17, 96.21) | 17 |  | 3.17 (2.6, 3.74) | 3.02(2.48, 3.57) |
| Niue |  | 76.26 (54.76, 97.75) | 15 | 92.45 (67.42, 117.48) | 14 | 93.16 (69.15, 117.16) | 16 |  | 0.67 (-1.27, 2.67) | 0.65(-1.11, 2.48) |
| Northern Mariana Islands |  | 56.58 (43.12, 70.04) | 18 | 110.23 (85.21, 135.25) | 13 | 122.13 (96.26, 148.01) | 12 |  | 2.33 (0.68, 4.02) | 2.51(1.03, 4.06) |
| Palau |  | 0.29 (0.2, 0.38) | 31 | 0.44 (0.34, 0.53) | 31 | 0.46 (0.36, 0.57) | 31 |  | 1.45 (-0.38, 3.42) | 1.5(-0.17, 3.44) |
| Papua New Guinea |  | 41.98 (19.03, 64.93) | 22 | 31.06 (17.06, 45.06) | 30 | 31.54 (16.84, 46.23) | 30 |  | -1.03 (-4.5, 3.02) | -0.92(-4.26, 2.9) |
| Philippines |  | 49.48 (37.2, 61.76) | 21 | 69.57 (62.9, 76.24) | 25 | 70.59 (60.86, 80.32) | 25 |  | 1.18 (0.06, 2.51) | 1.15(-0.05, 2.51) |
| Samoa |  | 37.62 (29.17, 46.08) | 27 | 50.21 (37.39, 63.03) | 27 | 50.4 (37, 63.79) | 27 |  | 1 (-0.72, 2.69) | 0.95(-0.71, 2.56) |
| Singapore |  | 81.16 (68.49, 93.82) | 14 | 89.09 (73.85, 104.34) | 16 | 96.39 (79.6, 113.17) | 15 |  | 0.32 (-0.82, 1.46) | 0.56(-0.53, 1.63) |
| Solomon Islands |  | 40.45 (18.42, 62.49) | 23 | 49.36 (33.83, 64.88) | 28 | 49.57 (33.42, 65.71) | 29 |  | 0.69 (-2.09, 4.44) | 0.66(-2, 4.19) |
| South Korea |  | 345.06 (273.63, 416.48) | 2 | 248.91 (205.32, 292.51) | 2 | 276.03 (223.25, 328.82) | 2 |  | -1.12 (-2.41, 0.23) | -0.72(-1.99, 0.59) |
| Tokelau |  | 88.31 (55.15, 121.46) | 12 | 90.35 (63.37, 117.33) | 15 | 98.65 (69.75, 127.54) | 14 |  | 0.08 (-2.22, 2.64) | 0.36(-1.77, 2.74) |
| Tonga |  | 189.18 (132.12, 246.23) | 3 | 194.38 (150.73, 238.02) | 4 | 196.17 (151.18, 241.16) | 5 |  | 0.09 (-1.68, 2.05) | 0.12(-1.56, 1.96) |
| Tuvalu |  | 82.71 (55.73, 109.68) | 13 | 84.76 (62.76, 106.76) | 19 | 84.2 (62.06, 106.35) | 20 |  | 0.08 (-1.91, 2.27) | 0.06(-1.82, 2.11) |
| Vanuatu |  | 39.31 (20.65, 57.96) | 25 | 49.08 (35.11, 63.06) | 29 | 49.91 (35.86, 63.96) | 28 |  | 0.77 (-1.71, 3.92) | 0.77(-1.54, 3.71) |
| Viet Nam |  | 96.31 (75.63, 116.99) | 10 | 137.31 (104.93, 169.69) | 9 | 144.13 (109.99, 178.27) | 8 |  | 1.23 (-0.37, 2.83) | 1.31(-0.2, 2.8) |
| Western Pacific Region |  | 136.82 (122.51, 151.13) |  | 167.89 (148.26, 187.53) |  | 166.96 (146.65, 187.26) |  |  | 0.71 (-0.07, 1.48) | 0.64(-0.1, 1.38) |

Abbreviation: DALYs, Disability-adjusted life years; UI, Uncertainty interval; EAPC, Estimated annual percentage change; CI, Confidence interval

Table S2. Cases of 5 specific liver cancer in the Western Pacific region, in 1990, 2019, and 2021

| **Liver cancer cause** | **1990 (95% UI)** | **2019 (95% UI)** | **2021 (95% UI)** | **Percentage change in 1990-2019, % (95% CI)** | **Percentage change in 1990-2021, % (95% CI)** |
| --- | --- | --- | --- | --- | --- |
|  | **DALYs** | | | | |
| **Total** | 4343651.05  (3890794.31, 4796507.79) | 6423768.10  (5487102.65, 7360433.56) | 6444759.28  (5504842.21, 7384676.36) | 47.89 (14.40, 89.18) | 48.37 (14.77, 89.80) |
| **LCHB** | 2740623.64  (2329963.87, 3182311.19) | 3856477.14  (3067069.76, 4863506.58) | 3742140.33  (2976713.25, 4732866.78) | 40.72 (-3.62, 108.74) | 36.54 (-6.46, 103.13) |
| **LCHC** | 807544.22  (705658.49, 925684.4) | 1226956.83  (1021119.1, 1435198.57) | 1280012.73  (1061641.13, 1519791.7) | 51.94 (10.31, 103.38) | 58.51 (14.69115.37) |
| **LCAL** | 396087.73  (314992.92, 496431.87) | 762823.56  (605972.17, 976863.73) | 783727.69  (593414.18, 1032579.69) | 92.59 (22.07, 210.12) | 97.87 (19.54, 227.81) |
| **LCNA** | 181879.72  (150293.75, 217866.86) | 354737.12  (277931.85, 439415.09) | 356728.55  (277122.72, 439994.73) | 95.04 (27.57, 192.37) | 96.13 (27.2, 192.76) |
| **LCOT** | 217515.74  (177235.54, 266248.7) | 288573.6  (224675.59, 368397.03) | 282149.97  (219515.74, 362364.02) | 32.67 (-15.61, 107.86) | 29.71 (-17.55, 104.45) |
|  | **Incidence** | | | | |
| **Total** | 140251.73  (126632.51, 153870.95) | 276213.85  (239682.1, 312745.6) | 280124.39  (244025.95, 316222.82) | 96.94 (55.77, 146.97) | 99.73 (58.59, 149.72) |
| **LCHB** | 79481.2  (67674.76, 92651.32) | 145655.35  (115995.5, 183764.81) | 143872.34  (115070.13, 180317.24) | 83.26 (25.2, 171.54) | 81.01 (24.2166.45) |
| **LCHC** | 33889.82  (29937.99, 38218.9) | 69218.55  (58730.21, 79136.58) | 73235.75  (61459.99, 84779.66) | 104.25 (53.67, 164.33) | 116.1 (60.81183.18) |
| **LCAL** | 14111.04  (11356.74, 17402.93) | 33527.53  (26710.86, 42359.34) | 34891.27  (27014.47, 45009.86) | 137.6 (53.48, 272.99) | 147.26 (55.23296.33) |
| **LCNA** | 6355.64  (5176.77, 7644.23) | 16278.31  (12818.21, 20127.67) | 16630.67  (13036.15, 20562.57) | 156.12 (67.68, 288.81) | 161.67 (70.54297.21) |
| **LCOT** | 6414.03  (5217.83, 7841.89) | 11534.1  (9027.54, 14583.85) | 11494.35  (8892.52, 14452.63) | 79.83 (15.12, 179.5) | 79.21 (13.4176.99) |
|  | **Prevalence** | | | | |
| **Total** | 179116.05  (162103.32, 196128.78) | 393944  (343569.5, 444318.5) | 400357.88  (349454.85, 451260.92) | 119.94 (75.18, 174.10) | 123.52 (78.18, 178.38) |
| **LCHB** | 102031.41  (87041.45, 118558.72) | 208741.71  (167681.7, 261622.16) | 207276.99  (166339.77, 258941.58) | 104.59 (41.43, 200.57) | 103.15 (40.3197.49) |
| **LCHC** | 43576.04  (39043.22, 48586.14) | 100354.65  (85980.76, 113106.8) | 105602.08  (89805.47, 121273.96) | 130.3 (76.97, 189.7) | 142.34 (84.84210.61) |
| **LCAL** | 17501.9  (14236.15, 21631.81) | 46753.23  (37598.02, 58666.21) | 48828.85  (37816.57, 63031.29) | 167.13 (73.81, 312.09) | 178.99 (74.82342.76) |
| **LCNA** | 7649.71  (6335.17, 9087.2) | 21730.68  (17273.74, 26567.78) | 22286.33  (17462.7, 27624.1) | 184.07 (90.09, 319.37) | 191.34 (92.17336.04) |
| **LCOT** | 8356.98  (6858.96, 10184.07) | 16363.72  (12847.47, 20559.86) | 16363.63  (12812.95, 20563.87) | 95.81 (26.15, 199.75) | 95.81 (25.81199.81) |
|  | **Mortality** | | | | |
| **Total** | 134989.1  (122017.56, 147960.64) | 239474.2  (208444.46, 270503.94) | 242358.14  (211663.27, 273053.02) | 77.40 (40.88, 121.69) | 79.54 (43.05, 123.78) |
| **LCHB** | 76688.16  (65509.43, 89094.22) | 123116.58  (97857.63, 154507.2) | 120940.27  (96883.2, 151536.4) | 60.54 (9.84, 135.85) | 57.7 (8.74131.32) |
| **LCHC** | 32206.64  (28280.7, 36615.11) | 61889.81  (51859.56, 71265.51) | 65670.42  (54567.07, 76091.08) | 92.16 (41.63, 151.99) | 103.9 (49.03169.06) |
| **LCAL** | 13614.56  (10876.09, 16790) | 29664.27  (23605.22, 37671.49) | 30767.08  (23785.55, 39559.11) | 117.89 (40.59, 246.37) | 125.99 (41.66263.73) |
| **LCNA** | 6295.06  (5143.35, 7594.05) | 14822.97  (11653.63, 18355.52) | 15083.1  (11863.99, 18752.31) | 135.47 (53.46, 256.88) | 139.6 (56.23264.59) |
| **LCOT** | 6184.68  (5030.86, 7552.46) | 9980.58  (7787.07, 12566.22) | 9897.27  (7660.14, 12370.93) | 61.38 (3.11, 149.78) | 60.03 (1.43145.9) |

Abbreviation: UI, Uncertainty interval, CI, Confidence interval; DALYs, Disability-adjusted life years; LCHB, Liver cancer by HBV; LCHC, Liver cancer by HCV; LCAL, Liver cancer by alcoholic use; LCNA; Liver cancer by nonalcoholic steatohepatitis (NASH); LCOT, Liver cancer by other causes

Table S3. Age-standardized liver cancer incidence rates (per 100,000) in the Western Pacific region by member state, in 1990, 2019, and 2021.

| **Location** |  | **Age-standardized incidence rate, per 100, 000 (95% UI)** | | | | | |  | **EAPC, % (95%CI)** | |
| --- | --- | --- | --- | --- | --- | --- | --- | --- | --- | --- |
|  |  | **1990** | **Rank** | **2019** | **Rank** | **2021** | **Rank** |  | **1990-2019** | **1990-2021** |
| Western Pacific region |  | 9.1 (8.21, 9.98) |  | 14.46 (12.55, 16.37) |  | 14.55 (12.67, 16.42) |  |  | 1.61 (1.47, 1.72) | 1.53 (1.41, 1.62) |
| **Aged countries** |  |  |  |  |  |  |  |  |  |  |
| South Korea |  | 25.59 (20.48, 30.85) | 2 | 32.33 (27.38, 37.39) | 2 | 36.15 (30.09, 42.21) | 2 |  | 0.81 (0.67, 1.01) | 1.12 (1.02, 1.25) |
| Cook Islands |  | 6.64 (5.23, 8.05) | 7 | 14.26 (11.42, 17.03) | 5 | 15.33 (12.16, 18.51) | 5 |  | 2.67 (2.62, 2.73) | 2.74 (2.72, 2.76) |
| Japan |  | 19.62 (18.59, 20.64) | 3 | 29.2 (26.53, 31.91) | 3 | 30.65 (27.76, 33.51) | 3 |  | 1.38 (1.23, 1.51) | 1.45 (1.3, 1.58) |
| China |  | 8 (6.96, 9.03) | 6 | 13.96 (11.63, 16.3) | 6 | 13.77 (11.48, 16.07) | 6 |  | 1.94 (1.79, 2.06) | 1.77 (1.63, 1.88) |
| Australia |  | 2.11 (1.81, 2.41) | 26 | 9.5 (8.14, 10.9) | 9 | 9.72 (8.26, 11.17) | 10 |  | 5.33 (5.32, 5.34) | 5.05 (5.02, 5.07) |
| Singapore |  | 6.56 (5.71, 7.41) | 8 | 10.02 (8.47, 11.6) | 8 | 10.98 (9.26, 12.73) | 8 |  | 1.47 (1.37, 1.56) | 1.68 (1.57, 1.76) |
| New Zealand |  | 2.69 (2.47, 2.91) | 20 | 7.95 (7.31, 8.6) | 11 | 8.21 (7.53, 8.9) | 11 |  | 3.81 (3.81, 3.81) | 3.66 (3.66, 3.67) |
| **Aging countries** |  |  |  |  |  |  |  |  |  |  |
| Viet Nam |  | 8.03 (6.09, 9.92) | 5 | 12.18 (9.18, 15.16) | 7 | 12.9 (9.57, 16.22) | 7 |  | 1.45 (1.43, 1.47) | 1.54 (1.47, 1.6) |
| Palau |  | 4.89 (3.4, 6.36) | 11 | 9.47 (7.2, 11.73) | 10 | 10.16 (7.64, 12.76) | 9 |  | 2.31 (2.13, 2.62) | 2.39 (2.27, 2.65) |
| Northern Mariana Islands |  | 2.3 (1.73, 2.87) | 23 | 6.57 (5.25, 7.91) | 14 | 7.36 (5.95, 8.76) | 13 |  | 3.69 (3.56, 3.9) | 3.82 (3.67, 4.07) |
| Guam |  | 2.02 (1.58, 2.47) | 27 | 6.72 (5.67, 7.77) | 13 | 7.22 (5.99, 8.45) | 14 |  | 4.23 (4.03, 4.5) | 4.19 (4.05, 4.39) |
| American Samoa |  | 1.85 (1.57, 2.14) | 29 | 5.85 (4.73, 6.98) | 16 | 6.19 (4.93, 7.49) | 16 |  | 4.05 (3.88, 4.16) | 3.97 (3.76, 4.12) |
| Niue |  | 3.88 (2.85, 4.91) | 12 | 6.14 (4.31, 7.95) | 15 | 6.3 (4.44, 8.12) | 15 |  | 1.6 (1.44, 1.68) | 1.58 (1.44, 1.64) |
| Malaysia |  | 2.25 (1.83, 2.68) | 24 | 4.94 (4.03, 5.85) | 17 | 5.07 (4.08, 6.07) | 17 |  | 2.75 (2.73, 2.76) | 2.66 (2.62, 2.67) |
| Tokelau |  | 3.07 (1.96, 4.23) | 18 | 4.5 (3.14, 5.88) | 19 | 4.93 (3.41, 6.47) | 18 |  | 1.33 (1.14, 1.64) | 1.54 (1.38, 1.8) |
| Tuvalu |  | 3.22 (2.29, 4.17) | 17 | 3.93 (2.87, 4.98) | 22 | 3.96 (2.88, 5.04) | 22 |  | 0.69 (0.61, 0.78) | 0.67 (0.61, 0.74) |
| **Young countries** |  |  |  |  |  |  |  |  |  |  |
| Mongolia |  | 28.35 (21.49, 35.33) | 1 | 48.79 (39.01, 58.61) | 1 | 51.01 (40.46, 61.57) | 1 |  | 1.89 (1.76, 2.08) | 1.91 (1.81, 2.06) |
| Tonga |  | 11.99 (8.89, 15) | 4 | 15.4 (11.69, 19.11) | 4 | 15.7 (11.77, 19.61) | 4 |  | 0.87 (0.84, 0.95) | 0.87 (0.87, 0.91) |
| Brunei Darussalam |  | 5.74 (4.39, 7.11) | 9 | 6.88 (5.51, 8.27) | 12 | 7.65 (6.05, 9.25) | 12 |  | 0.63 (0.52, 0.79) | 0.93 (0.85, 1.04) |
| Cambodia |  | 3.79 (2.26, 5.33) | 13 | 4.55 (2.74, 6.35) | 18 | 4.75 (2.95, 6.56) | 19 |  | 0.63 (0.61, 0.67) | 0.73 (0.67, 0.86) |
| Vanuatu |  | 1.99 (1.16, 2.8) | 28 | 4.43 (4, 4.86) | 20 | 4.5 (3.92, 5.1) | 20 |  | 2.8 (1.92, 4.36) | 2.67 (1.95, 4.01) |
| Laos |  | 5.16 (3.83, 6.48) | 10 | 4.18 (3.2, 5.16) | 21 | 4.32 (3.27, 5.36) | 21 |  | -0.72 (-0.78, -0.62) | -0.57 (-0.61, -0.51) |
| Kiribati |  | 3.49 (2.76, 4.23) | 16 | 3.78 (2.82, 4.71) | 24 | 3.85 (2.91, 4.79) | 25 |  | 0.28 (0.07, 0.37) | 0.32 (0.17, 0.4) |
| Federated States of Micronesia |  | 2.42 (1.72, 3.11) | 22 | 3.7 (2.41, 5.03) | 25 | 3.89 (2.53, 5.24) | 24 |  | 1.47 (1.17, 1.67) | 1.54 (1.25, 1.7) |
| Nauru |  | 3.57 (2.75, 4.41) | 14 | 3.39 (2.33, 4.48) | 26 | 3.47 (2.35, 4.6) | 26 |  | -0.18 (-0.57, 0.05) | -0.09 (-0.51, 0.14) |
| Fiji |  | 2.12 (1.58, 2.67) | 25 | 3.8 (2.95, 4.64) | 23 | 3.95 (2.99, 4.91) | 23 |  | 2.03 (1.92, 2.18) | 2.03 (1.98, 2.08) |
| Samoa |  | 2.62 (2.07, 3.17) | 21 | 3.12 (2.45, 3.81) | 27 | 3.18 (2.46, 3.91) | 27 |  | 0.6 (0.58, 0.64) | 0.63 (0.56, 0.68) |
| Solomon Islands |  | 2.79 (1.06, 4.52) | 19 | 2.65 (1.73, 3.56) | 28 | 2.67 (1.74, 3.57) | 28 |  | -0.18 (-0.82, 1.7) | -0.14 (-0.76, 1.61) |
| Philippines |  | 3.53 (2.86, 4.19) | 15 | 2.44 (1.46, 3.41) | 29 | 2.58 (1.56, 3.6) | 29 |  | -1.27 (-2.29, -0.71) | -1.01 (-1.94, -0.49) |
| Marshall Islands |  | 1.23 (0.75, 1.71) | 31 | 2.37 (1.64, 3.07) | 30 | 2.44 (1.66, 3.22) | 30 |  | 2.29 (2.04, 2.73) | 2.23 (2.06, 2.6) |
| Papua New Guinea |  | 1.74 (0.61, 2.89) | 30 | 1.26 (0.38, 2.14) | 31 | 1.3 (0.41, 2.18) | 31 |  | -1.11 (-1.62, -1.03) | -0.94 (-1.27, -0.91) |

Abbreviation: UI, Uncertainty interval, EAPC, Estimated annual percentage change; CI, Confidence interval

Table S4. Age-standardized liver cancer incidence rates (per 100,000) in the Western Pacific region by member state and sex, in 1990, 2019, and 2021.

| **Location** |  | **Age-standardized incidence rate, per 100, 000 (95% UI)** | | | | | |  | **EAPC, % (95%CI)** | |
| --- | --- | --- | --- | --- | --- | --- | --- | --- | --- | --- |
|  |  | **1990** | **Rank** | **2019** | **Rank** | **2021** | **Rank** |  | **1990-2019** | **1990-2021** |
| **Male** |  |  |  |  |  |  |  |  |  |  |
| American Samoa |  | 2.47 (1.83, 3.11) | 29 | 6.97 (5.33, 8.6) | 17 | 7.36 (5.64, 9.09) | 17 |  | 3.64 (1.88, 5.48) | 3.58(1.94, 5.31) |
| Australia |  | 3.11 (2.63, 3.6) | 22 | 13.84 (11.75, 15.94) | 10 | 14.23 (11.96, 16.49) | 10 |  | 5.28 (4.16, 6.41) | 5.03(3.95, 6.1) |
| Brunei Darussalam |  | 7.58 (5.73, 9.43) | 10 | 8.78 (6.87, 10.69) | 14 | 9.71 (7.57, 11.86) | 14 |  | 0.51 (-1.09, 2.17) | 0.8(-0.71, 2.37) |
| Cambodia |  | 4.05 (1.75, 6.36) | 16 | 4.81 (1.95, 7.68) | 23 | 5.04 (2.07, 8.01) | 22 |  | 0.59 (-3.99, 5.23) | 0.71(-3.56, 5.03) |
| China |  | 11.37 (9.48, 13.25) | 6 | 20 (15.67, 24.34) | 6 | 19.7 (15.35, 24.06) | 7 |  | 1.97 (0.58, 3.3) | 1.79(0.48, 3.05) |
| Cook Islands |  | 8.3 (6.33, 10.26) | 9 | 20.96 (16.39, 25.52) | 5 | 22.58 (17.37, 27.79) | 5 |  | 3.25 (1.63, 4.92) | 3.28(1.71, 4.89) |
| Federated States of Micronesia |  | 3 (1.8, 4.2) | 24 | 4.68 (2.48, 6.88) | 25 | 4.94 (2.65, 7.22) | 24 |  | 1.55 (-1.8, 4.73) | 1.62(-1.47, 4.58) |
| Fiji |  | 2.7 (1.91, 3.49) | 27 | 4.88 (3.54, 6.22) | 21 | 5.09 (3.58, 6.6) | 21 |  | 2.06 (0.05, 4.16) | 2.07(0.08, 4.08) |
| Guam |  | 2.54 (2.14, 2.95) | 28 | 10.73 (8.95, 12.51) | 11 | 11.43 (9.39, 13.47) | 11 |  | 5.09 (3.9, 6.28) | 4.97(3.81, 6.11) |
| Japan |  | 29.76 (28.16, 31.36) | 3 | 39.73 (36.99, 42.47) | 3 | 41.64 (38.5, 44.77) | 3 |  | 1 (0.57, 1.43) | 1.09(0.66, 1.51) |
| Kiribati |  | 3.44 (2.6, 4.28) | 20 | 3.41 (2.16, 4.67) | 28 | 3.48 (2.25, 4.7) | 28 |  | -0.03 (-2.33, 2.04) | 0.04(-2.05, 1.93) |
| Laos |  | 7.49 (5.35, 9.63) | 11 | 5.86 (4.24, 7.47) | 19 | 6.05 (4.39, 7.72) | 20 |  | -0.84 (-2.79, 1.16) | -0.69(-2.5, 1.19) |
| Malaysia |  | 3.13 (2.46, 3.8) | 21 | 7.21 (5.75, 8.67) | 16 | 7.37 (5.74, 9) | 16 |  | 2.92 (1.44, 4.44) | 2.8(1.34, 4.27) |
| Marshall Islands |  | 1.33 (0.69, 1.96) | 31 | 2.81 (1.23, 4.4) | 30 | 2.99 (1.29, 4.68) | 30 |  | 2.61 (-1.59, 6.6) | 2.65(-1.34, 6.37) |
| Mongolia |  | 36.52 (26.98, 46.06) | 2 | 55.4 (42.87, 67.93) | 1 | 57.67 (44.54, 70.81) | 1 |  | 1.45 (-0.25, 3.24) | 1.48(-0.11, 3.16) |
| Nauru |  | 5.16 (3.8, 6.52) | 13 | 4.8 (2.97, 6.64) | 24 | 4.89 (3, 6.79) | 25 |  | -0.25 (-2.67, 1.94) | -0.17(-2.47, 1.89) |
| New Zealand |  | 3.63 (3.28, 3.99) | 19 | 10.4 (9.4, 11.4) | 12 | 10.74 (9.66, 11.81) | 12 |  | 3.7 (3, 4.39) | 3.56(2.89, 4.22) |
| Niue |  | 4.85 (3.36, 6.34) | 14 | 8.68 (5.62, 11.74) | 15 | 8.98 (5.8, 12.16) | 15 |  | 2.03 (-0.41, 4.41) | 2.01(-0.29, 4.24) |
| Northern Mariana Islands |  | 2.92 (2.08, 3.76) | 25 | 8.99 (6.97, 11.02) | 13 | 10.05 (7.93, 12.18) | 13 |  | 3.95 (2.15, 5.92) | 4.07(2.44, 5.87) |
| Palau |  | 9.47 (6.58, 12.37) | 8 | 17.29 (13.1, 21.47) | 8 | 18.4 (13.71, 23.09) | 8 |  | 2.1 (0.2, 4.16) | 2.17(0.33, 4.13) |
| Papua New Guinea |  | 2.22 (0.47, 3.96) | 30 | 1.59 (0.17, 3.02) | 31 | 1.64 (0.21, 3.07) | 31 |  | -1.14 (-10.29, 6.62) | -0.97(-9.04, 6.24) |
| Philippines |  | 5.49 (4.43, 6.54) | 12 | 6.37 (5.64, 7.09) | 18 | 6.47 (5.4, 7.54) | 18 |  | 0.51 (-0.51, 1.63) | 0.53(-0.62, 1.73) |
| Samoa |  | 3.82 (2.94, 4.7) | 18 | 4.4 (3.32, 5.48) | 26 | 4.5 (3.35, 5.64) | 26 |  | 0.49 (-1.19, 2.17) | 0.53(-1.09, 2.12) |
| Singapore |  | 9.75 (8.51, 10.99) | 7 | 14.17 (11.91, 16.44) | 9 | 15.57 (13.14, 18.01) | 9 |  | 1.3 (0.28, 2.3) | 1.52(0.58, 2.45) |
| Solomon Islands |  | 4.26 (1.43, 7.09) | 15 | 3.8 (2.23, 5.36) | 27 | 3.84 (2.31, 5.36) | 27 |  | -0.39 (-3.91, 4.66) | -0.33(-3.55, 4.35) |
| South Korea |  | 38.02 (29.21, 46.83) | 1 | 47.71 (39.93, 55.49) | 2 | 53.17 (43.44, 62.9) | 2 |  | 0.79 (-0.55, 2.24) | 1.09(-0.24, 2.51) |
| Tokelau |  | 3.11 (1.67, 4.55) | 23 | 5.77 (3.77, 7.76) | 20 | 6.33 (4.09, 8.56) | 19 |  | 2.15 (-0.65, 5.44) | 2.32(-0.34, 5.41) |
| Tonga |  | 17.86 (12.98, 22.73) | 4 | 23.93 (17.4, 30.46) | 4 | 24.38 (17.56, 31.19) | 4 |  | 1.01 (-0.92, 2.99) | 1.01(-0.83, 2.87) |
| Tuvalu |  | 3.85 (2.54, 5.17) | 17 | 4.88 (3.25, 6.5) | 22 | 4.94 (3.23, 6.64) | 23 |  | 0.82 (-1.59, 3.29) | 0.81(-1.51, 3.15) |
| Vanuatu |  | 2.76 (1.44, 4.07) | 26 | 3.18 (2, 4.37) | 29 | 3.29 (2.06, 4.53) | 29 |  | 0.49 (-2.42, 3.9) | 0.57(-2.17, 3.77) |
| Viet Nam |  | 12.93 (9.6, 16.27) | 5 | 19.09 (13.81, 24.37) | 7 | 20.11 (14.15, 26.07) | 17 |  | 1.35 (-0.56, 3.26) | 1.43(-0.45, 3.28) |
| Western Pacific Region |  | 13.14 (11.52, 14.75) |  | 20.61 (17.18, 24.03) |  | 20.68 (17.29, 24.07) |  |  | 1.56 (0.53, 2.57) | 1.47(0.51, 2.41) |
| **Female** |  |  |  |  |  |  |  |  |  |  |
| American Samoa |  | 1.54 (1.17, 1.92) | 20 | 4.68 (3.68, 5.68) | 12 | 4.97 (3.85, 6.08) | 12 |  | 3.91 (2.27, 5.6) | 3.85(2.27, 5.46) |
| Australia |  | 1.1 (0.94, 1.27) | 29 | 5.26 (4.43, 6.1) | 10 | 5.34 (4.46, 6.21) | 11 |  | 5.54 (4.4, 6.66) | 5.23(4.14, 6.28) |
| Brunei Darussalam |  | 3.67 (2.73, 4.62) | 7 | 4.79 (3.65, 5.93) | 11 | 5.37 (3.97, 6.78) | 10 |  | 0.92 (-0.81, 2.71) | 1.24(-0.49, 2.98) |
| Cambodia |  | 3.55 (1.91, 5.19) | 9 | 4.31 (2.82, 5.8) | 13 | 4.51 (3, 6.02) | 13 |  | 0.67 (-2.08, 3.9) | 0.78(-1.75, 3.77) |
| China |  | 4.42 (3.87, 4.97) | 6 | 7.61 (6.6, 8.62) | 5 | 7.57 (6.48, 8.67) | 5 |  | 1.89 (0.98, 2.8) | 1.75(0.86, 2.64) |
| Cook Islands |  | 4.88 (3.82, 5.93) | 5 | 7.91 (6.29, 9.54) | 4 | 8.48 (6.65, 10.31) | 4 |  | 1.68 (0.2, 3.21) | 1.8(0.37, 3.25) |
| Federated States of Micronesia |  | 1.52 (1.04, 2.01) | 22 | 2.67 (2.11, 3.24) | 20 | 2.8 (2.17, 3.42) | 20 |  | 1.96 (0.17, 4) | 1.99(0.25, 3.91) |
| Fiji |  | 1.06 (0.87, 1.24) | 30 | 2.58 (2.16, 3) | 21 | 2.89 (2.33, 3.45) | 18 |  | 3.11 (1.93, 4.36) | 3.29(2.06, 4.54) |
| Guam |  | 9.8 (9.19, 10.41) | 3 | 19.19 (16.01, 22.37) | 2 | 20.21 (16.81, 23.61) | 2 |  | 2.34 (1.5, 3.12) | 2.36(1.56, 3.09) |
| Japan |  | 3.56 (2.78, 4.34) | 8 | 4.14 (3.17, 5.12) | 14 | 4.22 (3.19, 5.25) | 15 |  | 0.52 (-1.08, 2.13) | 0.55(-0.99, 2.07) |
| Kiribati |  | 2.91 (2.22, 3.6) | 13 | 2.48 (1.92, 3.04) | 23 | 2.56 (1.96, 3.15) | 23 |  | -0.55 (-2.14, 1.09) | -0.41(-1.94, 1.14) |
| Laos |  | 1.36 (1.09, 1.62) | 23 | 2.52 (2.05, 2.99) | 22 | 2.59 (2.08, 3.1) | 22 |  | 2.15 (0.82, 3.54) | 2.1(0.81, 3.43) |
| Malaysia |  | 1.14 (0.72, 1.56) | 28 | 2.04 (1.47, 2.62) | 25 | 2.14 (1.53, 2.75) | 25 |  | 2.03 (-0.2, 4.55) | 2.05(-0.06, 4.42) |
| Marshall Islands |  | 1.81 (1.37, 2.26) | 17 | 2.68 (1.85, 3.5) | 19 | 2.78 (1.92, 3.64) | 21 |  | 1.36 (-0.69, 3.29) | 1.39(-0.52, 3.2) |
| Mongolia |  | 20.16 (14.56, 25.75) | 1 | 42.35 (34.05, 50.65) | 1 | 44.37 (35.32, 53.41) | 1 |  | 2.59 (0.97, 4.39) | 2.58(1.02, 4.28) |
| Nauru |  | 1.87 (1.43, 2.31) | 16 | 1.96 (1.35, 2.56) | 26 | 2.03 (1.42, 2.64) | 26 |  | 0.16 (-1.84, 2.03) | 0.27(-1.56, 2) |
| New Zealand |  | 1.76 (1.61, 1.92) | 18 | 5.53 (4.98, 6.07) | 8 | 5.69 (5.11, 6.28) | 8 |  | 4.03 (3.34, 4.68) | 3.86(3.21, 4.49) |
| Niue |  | 2.9 (2.11, 3.7) | 14 | 3.59 (2.63, 4.55) | 16 | 3.61 (2.66, 4.56) | 16 |  | 0.74 (-1.17, 2.69) | 0.71(-1.06, 2.52) |
| Northern Mariana Islands |  | 1.61 (1.25, 1.98) | 19 | 3.9 (3.04, 4.77) | 15 | 4.39 (3.49, 5.29) | 14 |  | 3.1 (1.49, 4.73) | 3.29(1.85, 4.76) |
| Palau |  | 0.01 (0.01, 0.01) | 31 | 0.02 (0.01, 0.02) | 31 | 0.02 (0.01, 0.02) | 31 |  | 2.42 (0, 2.42) | 2.26(0, 2.26) |
| Papua New Guinea |  | 1.22 (0.55, 1.89) | 25 | 0.9 (0.5, 1.3) | 30 | 0.92 (0.49, 1.34) | 30 |  | -1.04 (-4.48, 3.01) | -0.91(-4.26, 2.91) |
| Philippines |  | 1.54 (1.15, 1.93) | 21 | 2.43 (2.21, 2.66) | 24 | 2.49 (2.16, 2.81) | 24 |  | 1.59 (0.47, 2.93) | 1.56(0.36, 2.92) |
| Samoa |  | 1.29 (1.01, 1.57) | 24 | 1.78 (1.34, 2.21) | 27 | 1.78 (1.34, 2.22) | 27 |  | 1.12 (-0.54, 2.74) | 1.04(-0.51, 2.57) |
| Singapore |  | 3.32 (2.77, 3.87) | 11 | 5.69 (4.68, 6.71) | 7 | 6.22 (5.08, 7.36) | 7 |  | 1.88 (0.66, 3.1) | 2.05(0.88, 3.2) |
| Solomon Islands |  | 1.17 (0.54, 1.79) | 27 | 1.43 (0.98, 1.89) | 29 | 1.44 (0.97, 1.92) | 29 |  | 0.69 (-2.06, 4.41) | 0.67(-1.96, 4.18) |
| South Korea |  | 12.99 (10.26, 15.72) | 2 | 16.88 (13.69, 20.07) | 3 | 18.96 (15.18, 22.74) | 3 |  | 0.91 (-0.48, 2.34) | 1.23(-0.11, 2.6) |
| Tokelau |  | 3.04 (1.91, 4.18) | 12 | 3.19 (2.24, 4.15) | 17 | 3.51 (2.43, 4.59) | 17 |  | 0.17 (-2.13, 2.71) | 0.46(-1.73, 2.87) |
| Tonga |  | 6.05 (4.19, 7.9) | 4 | 6.92 (5.35, 8.49) | 6 | 7.01 (5.38, 8.64) | 6 |  | 0.46 (-1.34, 2.47) | 0.48(-1.23, 2.36) |
| Tuvalu |  | 2.62 (1.75, 3.5) | 15 | 2.89 (2.12, 3.67) | 18 | 2.89 (2.14, 3.64) | 19 |  | 0.34 (-1.71, 2.59) | 0.32(-1.57, 2.39) |
| Vanuatu |  | 1.17 (0.63, 1.71) | 26 | 1.53 (1.11, 1.95) | 28 | 1.57 (1.12, 2.01) | 28 |  | 0.93 (-1.48, 3.97) | 0.95(-1.36, 3.81) |
| Viet Nam |  | 3.4 (2.67, 4.13) | 10 | 5.35 (4.12, 6.58) | 9 | 5.65 (4.34, 6.96) | 9 |  | 1.58 (-0.01, 3.16) | 1.65(0.16, 3.14) |
| Western Pacific Region |  | 4.88 (4.38, 5.38) |  | 8.1 (7.2, 9.01) |  | 8.21 (7.23, 9.19) |  |  | 1.76 (1.01, 2.52) | 1.69(0.96, 2.42) |

Abbreviation: UI, Uncertainty interval; EAPC, Estimated annual percentage change; CI, Confidence interval

Table S5. Age-standardized liver cancer prevalence rates (per 100,000) in the Western Pacific region by member state, in 1990, 2019, and 2021.

| **Location** |  | **Age-standardized prevalence rate, per 100, 000 (95% UI)** | | | | | |  | **EAPC, % (95%CI)** | |
| --- | --- | --- | --- | --- | --- | --- | --- | --- | --- | --- |
|  |  | **1990** | **Rank** | **2019** | **Rank** | **2021** | **Rank** |  | **1990-2019** | **2019-2021** |
| Western Pacific region |  | 11.62 (10.52, 12.72) |  | 20.62 (17.99, 23.26) |  | 20.79 (18.15, 23.44) |  |  | 2 (1.87, 2.1) | 1.89 (1.77, 1.99) |
| **Aged countries** |  |  |  |  |  |  |  |  |  |  |
| South Korea |  | 30.59 (24.08, 37.22) | 3 | 60.6 (51.33, 69.92) | 1 | 67.55 (56.16, 78.73) | 1 |  | 2.39 (2.2, 2.64) | 2.59 (2.45, 2.77) |
| Cook Islands |  | 7.5 (5.89, 9.06) | 8 | 16.73 (13.22, 20.19) | 7 | 18.01 (14.17, 21.89) | 6 |  | 2.81 (2.8, 2.83) | 2.87 (2.87, 2.89) |
| Japan |  | 31.23 (29.54, 32.92) | 1 | 54.24 (48.95, 59.46) | 2 | 56.71 (50.65, 62.85) | 2 |  | 1.92 (1.76, 2.06) | 1.94 (1.75, 2.11) |
| China |  | 9.74 (8.47, 11.06) | 5 | 18.55 (15.33, 21.81) | 4 | 18.31 (15.19, 21.47) | 5 |  | 2.25 (2.07, 2.37) | 2.06 (1.9, 2.16) |
| Australia |  | 2.46 (2.12, 2.81) | 26 | 14.08 (12.03, 16.15) | 10 | 14.07 (11.98, 16.17) | 10 |  | 6.2 (6.17, 6.22) | 5.79 (5.75, 5.81) |
| Singapore |  | 8.21 (7.19, 9.21) | 7 | 18.44 (15.59, 21.26) | 5 | 20.23 (17.05, 23.42) | 4 |  | 2.83 (2.7, 2.93) | 2.95 (2.82, 3.06) |
| New Zealand |  | 3.67 (3.37, 3.97) | 17 | 14.12 (12.94, 15.33) | 9 | 14.48 (13.2, 15.73) | 9 |  | 4.76 (4.75, 4.77) | 4.53 (4.5, 4.54) |
| **Aging countries** |  |  |  |  |  |  |  |  |  |  |
| Viet Nam |  | 9.12 (6.91, 11.28) | 6 | 14.78 (11, 18.52) | 8 | 15.61 (11.49, 19.68) | 8 |  | 1.68 (1.62, 1.72) | 1.75 (1.65, 1.81) |
| Palau |  | 5.68 (3.85, 7.5) | 11 | 11.48 (8.65, 14.32) | 11 | 12.24 (9.11, 15.31) | 11 |  | 2.46 (2.26, 2.83) | 2.51 (2.33, 2.82) |
| Northern Mariana Islands |  | 2.93 (2.17, 3.68) | 21 | 7.84 (6.2, 9.51) | 14 | 8.79 (7.02, 10.49) | 13 |  | 3.45 (3.33, 3.69) | 3.61 (3.44, 3.86) |
| Guam |  | 2.38 (1.83, 2.9) | 27 | 8.07 (6.84, 9.33) | 13 | 8.56 (7.13, 9.97) | 14 |  | 4.3 (4.11, 4.65) | 4.22 (4.06, 4.48) |
| American Samoa |  | 2.21 (1.86, 2.54) | 29 | 6.74 (5.41, 8.06) | 16 | 7.11 (5.58, 8.62) | 15 |  | 3.92 (3.75, 4.06) | 3.84 (3.61, 4.02) |
| Niue |  | 4.12 (3, 5.22) | 15 | 6.78 (4.76, 8.79) | 15 | 6.99 (4.87, 9.1) | 16 |  | 1.73 (1.6, 1.81) | 1.72 (1.58, 1.81) |
| Malaysia |  | 2.52 (2.05, 3) | 24 | 5.76 (4.68, 6.85) | 17 | 5.88 (4.69, 7.07) | 17 |  | 2.89 (2.89, 2.89) | 2.77 (2.71, 2.8) |
| Tokelau |  | 3.27 (2.05, 4.5) | 19 | 5.08 (3.48, 6.71) | 20 | 5.57 (3.83, 7.27) | 18 |  | 1.53 (1.39, 1.84) | 1.73 (1.56, 2.04) |
| Tuvalu |  | 3.58 (2.57, 4.59) | 18 | 4.46 (3.23, 5.71) | 23 | 4.5 (3.23, 5.76) | 25 |  | 0.76 (0.76, 0.79) | 0.74 (0.74, 0.74) |
| **Young countries** |  |  |  |  |  |  |  |  |  |  |
| Mongolia |  | 30.76 (23.26, 38.36) | 2 | 53.08 (42.43, 63.9) | 3 | 55.25 (44.05, 66.59) | 3 |  | 1.9 (1.78, 2.09) | 1.91 (1.8, 2.08) |
| Tonga |  | 13.61 (10.08, 17.09) | 4 | 17.55 (13.15, 21.95) | 6 | 17.89 (13.18, 22.59) | 7 |  | 0.88 (0.87, 0.92) | 0.89 (0.87, 0.9) |
| Brunei Darussalam |  | 6.61 (5.08, 8.18) | 9 | 8.48 (6.76, 10.23) | 12 | 9.39 (7.36, 11.39) | 12 |  | 0.86 (0.77, 0.99) | 1.14 (1.07, 1.2) |
| Cambodia |  | 4.28 (2.49, 6.02) | 14 | 5.14 (3.11, 7.29) | 18 | 5.37 (3.26, 7.48) | 19 |  | 0.63 (0.66, 0.77) | 0.73 (0.7, 0.87) |
| Vanuatu |  | 2.31 (1.32, 3.3) | 28 | 5.1 (4.6, 5.6) | 19 | 5.19 (4.49, 5.88) | 20 |  | 2.77 (1.84, 4.4) | 2.65 (1.88, 4.03) |
| Laos |  | 5.77 (4.27, 7.26) | 10 | 4.8 (3.67, 5.91) | 21 | 4.97 (3.73, 6.21) | 21 |  | -0.63 (-0.71, -0.52) | -0.48 (-0.5, -0.44) |
| Kiribati |  | 4.1 (3.22, 4.98) | 16 | 4.49 (3.31, 5.66) | 22 | 4.56 (3.43, 5.73) | 22 |  | 0.31 (0.1, 0.44) | 0.34 (0.2, 0.45) |
| Federated States of Micronesia |  | 2.79 (1.97, 3.62) | 23 | 4.33 (2.76, 5.89) | 24 | 4.54 (2.91, 6.12) | 23 |  | 1.53 (1.17, 1.69) | 1.58 (1.27, 1.71) |
| Nauru |  | 4.35 (3.32, 5.39) | 12 | 4.27 (2.9, 5.66) | 26 | 4.35 (2.89, 5.78) | 26 |  | -0.06 (-0.47, 0.17) | 0 (-0.45, 0.23) |
| Fiji |  | 2.5 (1.85, 3.14) | 25 | 4.33 (3.33, 5.33) | 25 | 4.53 (3.4, 5.62) | 24 |  | 1.91 (1.84, 2.05) | 1.94 (1.9, 1.98) |
| Samoa |  | 2.88 (2.26, 3.49) | 22 | 3.54 (2.76, 4.32) | 27 | 3.62 (2.71, 4.47) | 27 |  | 0.71 (0.69, 0.74) | 0.74 (0.59, 0.8) |
| Solomon Islands |  | 3.22 (1.18, 5.29) | 20 | 3.21 (2.07, 4.35) | 28 | 3.23 (2.13, 4.32) | 28 |  | -0.01 (-0.67, 1.96) | 0.01 (-0.65, 1.92) |
| Philippines |  | 4.35 (3.54, 5.17) | 13 | 2.92 (1.75, 4.08) | 29 | 3.08 (1.82, 4.34) | 29 |  | -1.37 (-2.4, -0.81) | -1.11 (-2.12, -0.56) |
| Marshall Islands |  | 1.44 (0.87, 1.99) | 31 | 2.73 (1.89, 3.59) | 30 | 2.81 (1.89, 3.73) | 30 |  | 2.23 (2.06, 2.71) | 2.18 (2.05, 2.53) |
| Papua New Guinea |  | 2.04 (0.7, 3.41) | 30 | 1.51 (0.48, 2.55) | 31 | 1.55 (0.47, 2.63) | 31 |  | -1.03 (-1.29, -1) | -0.88 (-1.28, -0.83) |

Abbreviation: UI, Uncertainty interval, EAPC, Estimated annual percentage change; CI, Confidence interval

Table S6. Age-standardized liver cancer prevalence rates (per 100,000) in the Western Pacific region by member state and sex, in 1990, 2019, and 2021.

| **Location** |  | **Age-standardized prevalence rate, per 100, 000 (95% UI)** | | | | | |  | **EAPC, % (95%CI)** | |
| --- | --- | --- | --- | --- | --- | --- | --- | --- | --- | --- |
|  |  | **1990** | **Rank** | **2019** | **Rank** | **2021** | **Rank** |  | **1990-2019** | **1990-2021** |
| **Male** |  |  |  |  |  |  |  |  |  |  |
| American Samoa |  | 2.91 (2.16, 3.67) | 29 | 8.09 (6.17, 10) | 17 | 8.52 (6.41, 10.62) | 17 |  | 3.59 (1.81, 5.43) | 3.53(1.82, 5.27) |
| Australia |  | 3.74 (3.17, 4.3) | 21 | 22.2 (18.76, 25.64) | 9 | 22.22 (18.73, 25.72) | 9 |  | 6.33 (5.21, 7.47) | 5.92(4.86, 6.99) |
| Brunei Darussalam |  | 8.89 (6.7, 11.09) | 10 | 11.07 (8.6, 13.53) | 13 | 12.21 (9.45, 14.96) | 13 |  | 0.76 (-0.87, 2.45) | 1.03(-0.51, 2.63) |
| Cambodia |  | 4.63 (1.95, 7.31) | 17 | 5.59 (2.18, 9) | 24 | 5.85 (2.34, 9.37) | 22 |  | 0.65 (-4.09, 5.42) | 0.76(-3.61, 5.19) |
| China |  | 14.2 (11.8, 16.6) | 6 | 27.33 (21.32, 33.35) | 5 | 26.93 (20.88, 32.98) | 6 |  | 2.28 (0.87, 3.65) | 2.09(0.74, 3.37) |
| Cook Islands |  | 9.37 (7.05, 11.7) | 9 | 24.55 (18.93, 30.16) | 7 | 26.51 (20.13, 32.89) | 7 |  | 3.38 (1.67, 5.14) | 3.41(1.77, 5.09) |
| Federated States of Micronesia |  | 3.47 (2.07, 4.87) | 24 | 5.54 (2.9, 8.18) | 25 | 5.83 (3.07, 8.58) | 23 |  | 1.63 (-1.77, 4.85) | 1.69(-1.48, 4.69) |
| Fiji |  | 3.16 (2.22, 4.09) | 27 | 5.6 (4.02, 7.18) | 23 | 5.82 (4.05, 7.6) | 24 |  | 1.99 (-0.06, 4.13) | 1.99(-0.03, 4.05) |
| Guam |  | 3.06 (2.58, 3.53) | 28 | 12.93 (10.81, 15.04) | 12 | 13.64 (11.25, 16.03) | 12 |  | 5.09 (3.93, 6.27) | 4.94(3.81, 6.07) |
| Japan |  | 47.38 (44.66, 50.1) | 1 | 76.75 (70.44, 83.06) | 2 | 80.33 (73.29, 87.37) | 2 |  | 1.68 (1.18, 2.16) | 1.72(1.23, 2.19) |
| Kiribati |  | 4.02 (3.01, 5.03) | 20 | 4.11 (2.57, 5.64) | 28 | 4.18 (2.67, 5.7) | 28 |  | 0.08 (-2.29, 2.19) | 0.13(-2.02, 2.08) |
| Laos |  | 8.49 (6.06, 10.91) | 11 | 6.83 (4.9, 8.76) | 19 | 7.05 (5.07, 9.03) | 20 |  | -0.75 (-2.72, 1.28) | -0.6(-2.44, 1.29) |
| Malaysia |  | 3.55 (2.77, 4.32) | 23 | 8.45 (6.68, 10.21) | 16 | 8.6 (6.68, 10.52) | 16 |  | 3.04 (1.51, 4.6) | 2.9(1.42, 4.4) |
| Marshall Islands |  | 1.54 (0.77, 2.3) | 31 | 3.36 (1.46, 5.26) | 30 | 3.55 (1.49, 5.62) | 30 |  | 2.73 (-1.55, 6.85) | 2.73(-1.39, 6.62) |
| Mongolia |  | 39.41 (29.03, 49.79) | 3 | 62.18 (48.06, 76.29) | 3 | 64.51 (49.52, 79.5) | 3 |  | 1.58 (-0.12, 3.39) | 1.6(-0.02, 3.3) |
| Nauru |  | 6.26 (4.56, 7.95) | 13 | 6.02 (3.68, 8.35) | 21 | 6.13 (3.69, 8.56) | 21 |  | -0.13 (-2.62, 2.11) | -0.07(-2.45, 2.05) |
| New Zealand |  | 5.18 (4.67, 5.69) | 15 | 19.51 (17.54, 21.48) | 11 | 19.95 (17.83, 22.06) | 11 |  | 4.68 (3.96, 5.4) | 4.45(3.75, 5.14) |
| Niue |  | 5.32 (3.69, 6.95) | 14 | 9.72 (6.18, 13.27) | 15 | 10.09 (6.35, 13.82) | 15 |  | 2.1 (-0.4, 4.51) | 2.09(-0.29, 4.35) |
| Northern Mariana Islands |  | 3.7 (2.58, 4.81) | 22 | 10.69 (8.2, 13.18) | 14 | 11.9 (9.31, 14.49) | 14 |  | 3.73 (1.86, 5.78) | 3.84(2.15, 5.72) |
| Palau |  | 11.01 (7.42, 14.6) | 8 | 20.91 (15.86, 25.96) | 10 | 22.17 (16.53, 27.81) | 10 |  | 2.24 (0.29, 4.41) | 2.28(0.4, 4.35) |
| Papua New Guinea |  | 2.58 (0.53, 4.64) | 30 | 1.9 (0.17, 3.63) | 31 | 1.95 (0.19, 3.72) | 31 |  | -1.05 (-10.78, 6.86) | -0.9(-9.79, 6.49) |
| Philippines |  | 6.88 (5.57, 8.19) | 12 | 7.48 (6.62, 8.33) | 18 | 7.62 (6.35, 8.88) | 18 |  | 0.29 (-0.73, 1.4) | 0.33(-0.82, 1.52) |
| Samoa |  | 4.22 (3.2, 5.25) | 19 | 5.02 (3.75, 6.29) | 26 | 5.15 (3.78, 6.53) | 26 |  | 0.6 (-1.15, 2.36) | 0.64(-1.05, 2.33) |
| Singapore |  | 12.42 (10.91, 13.92) | 7 | 26.26 (22.15, 30.37) | 6 | 28.94 (24.41, 33.47) | 4 |  | 2.62 (1.61, 3.59) | 2.77(1.83, 3.68) |
| Solomon Islands |  | 4.95 (1.58, 8.31) | 16 | 4.61 (2.68, 6.55) | 27 | 4.67 (2.81, 6.54) | 27 |  | -0.25 (-3.83, 5.03) | -0.19(-3.44, 4.69) |
| South Korea |  | 46.5 (35.11, 57.89) | 2 | 90.83 (76.01, 105.64) | 1 | 101.36 (83.02, 119.71) | 1 |  | 2.34 (0.94, 3.87) | 2.55(1.17, 4.04) |
| Tokelau |  | 3.32 (1.76, 4.88) | 25 | 6.53 (4.16, 8.91) | 20 | 7.15 (4.54, 9.76) | 19 |  | 2.36 (-0.55, 5.75) | 2.51(-0.23, 5.68) |
| Tonga |  | 20.2 (14.67, 25.73) | 4 | 27.46 (19.79, 35.14) | 4 | 28.02 (19.77, 36.26) | 5 |  | 1.06 (-0.9, 3.06) | 1.06(-0.85, 2.96) |
| Tuvalu |  | 4.31 (2.79, 5.82) | 18 | 5.63 (3.64, 7.61) | 22 | 5.7 (3.71, 7.69) | 25 |  | 0.93 (-1.61, 3.52) | 0.91(-1.44, 3.32) |
| Vanuatu |  | 3.18 (1.67, 4.68) | 26 | 3.68 (2.28, 5.08) | 29 | 3.8 (2.3, 5.3) | 29 |  | 0.5 (-2.45, 3.91) | 0.58(-2.27, 3.8) |
| Viet Nam |  | 14.9 (11.12, 18.68) | 5 | 23.31 (16.73, 29.89) | 8 | 24.61 (17.27, 31.94) | 8 |  | 1.56 (-0.38, 3.47) | 1.63(-0.25, 3.46) |
| Western Pacific Region |  | 17.11 (15.08, 19.15) |  | 30.1 (25.33, 34.87) |  | 30.3 (25.53, 35.06) |  |  | 1.97 (0.97, 2.93) | 1.86(0.93, 2.76) |
| **Female** |  |  |  |  |  |  |  |  |  |  |
| American Samoa |  | 1.81 (1.37, 2.24) | 21 | 5.34 (4.2, 6.47) | 12 | 5.65 (4.37, 6.93) | 12 |  | 3.8 (2.19, 5.5) | 3.74(2.18, 5.37) |
| Australia |  | 1.2 (1.03, 1.37) | 30 | 6.16 (5.22, 7.1) | 10 | 6.11 (5.14, 7.07) | 11 |  | 5.8 (4.72, 6.88) | 5.39(4.36, 6.41) |
| Brunei Darussalam |  | 4.01 (3, 5.02) | 8 | 5.6 (4.26, 6.93) | 11 | 6.24 (4.62, 7.87) | 10 |  | 1.16 (-0.56, 2.93) | 1.44(-0.27, 3.16) |
| Cambodia |  | 3.93 (2.17, 5.7) | 10 | 4.72 (3.1, 6.33) | 15 | 4.92 (3.27, 6.57) | 15 |  | 0.63 (-2.08, 3.76) | 0.73(-1.78, 3.64) |
| China |  | 4.99 (4.36, 5.61) | 6 | 9.37 (8.11, 10.63) | 5 | 9.31 (7.93, 10.68) | 6 |  | 2.2 (1.28, 3.12) | 2.03(1.12, 2.93) |
| Cook Islands |  | 5.47 (4.32, 6.63) | 5 | 9.28 (7.44, 11.11) | 6 | 9.95 (7.85, 12.04) | 5 |  | 1.84 (0.4, 3.31) | 1.95(0.55, 3.36) |
| Federated States of Micronesia |  | 1.82 (1.25, 2.4) | 20 | 3.05 (2.41, 3.69) | 21 | 3.17 (2.46, 3.89) | 21 |  | 1.8 (0.01, 3.8) | 1.81(0.08, 3.73) |
| Fiji |  | 1.24 (1.04, 1.44) | 29 | 3.07 (2.57, 3.57) | 20 | 3.35 (2.73, 3.97) | 18 |  | 3.18 (2.02, 4.34) | 3.26(2.08, 4.42) |
| Guam |  | 15.61 (14.62, 16.6) | 2 | 32.86 (27.22, 38.49) | 2 | 34.22 (28.11, 40.33) | 2 |  | 2.6 (1.72, 3.39) | 2.56(1.71, 3.33) |
| Japan |  | 4.18 (3.26, 5.1) | 7 | 4.86 (3.68, 6.03) | 13 | 4.94 (3.72, 6.16) | 14 |  | 0.52 (-1.12, 2.14) | 0.54(-1.01, 2.07) |
| Kiribati |  | 3.15 (2.41, 3.9) | 13 | 2.75 (2.12, 3.37) | 23 | 2.83 (2.18, 3.48) | 23 |  | -0.47 (-2.08, 1.16) | -0.34(-1.86, 1.19) |
| Laos |  | 1.47 (1.2, 1.75) | 23 | 2.9 (2.37, 3.43) | 22 | 2.97 (2.4, 3.54) | 22 |  | 2.37 (1.05, 3.69) | 2.29(1.02, 3.55) |
| Malaysia |  | 1.33 (0.85, 1.81) | 28 | 2.46 (1.76, 3.17) | 26 | 2.57 (1.82, 3.31) | 25 |  | 2.14 (-0.1, 4.64) | 2.15(0.02, 4.48) |
| Marshall Islands |  | 2.07 (1.55, 2.59) | 19 | 3.08 (2.12, 4.04) | 19 | 3.2 (2.19, 4.2) | 20 |  | 1.38 (-0.69, 3.36) | 1.42(-0.54, 3.27) |
| Mongolia |  | 22.11 (16.2, 28.02) | 1 | 44.12 (35.59, 52.65) | 1 | 46.02 (36.65, 55.4) | 1 |  | 2.41 (0.83, 4.15) | 2.39(0.87, 4.05) |
| Nauru |  | 2.3 (1.75, 2.85) | 16 | 2.48 (1.71, 3.24) | 25 | 2.54 (1.78, 3.31) | 26 |  | 0.26 (-1.75, 2.15) | 0.32(-1.51, 2.08) |
| New Zealand |  | 2.2 (2.01, 2.39) | 17 | 8.79 (7.89, 9.69) | 7 | 9.04 (8.08, 10) | 7 |  | 4.89 (4.2, 5.57) | 4.66(4.01, 5.31) |
| Niue |  | 2.91 (2.1, 3.73) | 14 | 3.81 (2.78, 4.84) | 16 | 3.85 (2.86, 4.85) | 17 |  | 0.93 (-1.01, 2.92) | 0.91(-0.85, 2.74) |
| Northern Mariana Islands |  | 2.08 (1.6, 2.57) | 18 | 4.73 (3.67, 5.79) | 14 | 5.32 (4.19, 6.44) | 13 |  | 2.87 (1.24, 4.53) | 3.08(1.59, 4.59) |
| Palau |  | 0.01 (0.01, 0.01) | 31 | 0.02 (0.01, 0.02) | 31 | 0.02 (0.01, 0.02) | 31 |  | 2.42 (0, 2.42) | 2.26(0, 2.26) |
| Papua New Guinea |  | 1.44 (0.66, 2.23) | 24 | 1.09 (0.61, 1.58) | 30 | 1.11 (0.6, 1.63) | 30 |  | -0.96 (-4.37, 3.06) | -0.84(-4.15, 2.96) |
| Philippines |  | 1.77 (1.33, 2.22) | 22 | 2.65 (2.4, 2.9) | 24 | 2.7 (2.34, 3.06) | 24 |  | 1.4 (0.27, 2.72) | 1.37(0.17, 2.72) |
| Samoa |  | 1.38 (1.07, 1.69) | 25 | 1.97 (1.47, 2.47) | 27 | 1.98 (1.47, 2.5) | 27 |  | 1.23 (-0.48, 2.93) | 1.17(-0.45, 2.78) |
| Singapore |  | 3.94 (3.31, 4.57) | 9 | 10.27 (8.28, 12.25) | 4 | 11.24 (9.11, 13.36) | 4 |  | 3.36 (2.07, 4.62) | 3.44(2.25, 4.6) |
| Solomon Islands |  | 1.37 (0.63, 2.11) | 26 | 1.73 (1.18, 2.29) | 29 | 1.74 (1.16, 2.33) | 29 |  | 0.81 (-1.98, 4.55) | 0.77(-1.91, 4.31) |
| South Korea |  | 14.59 (11.55, 17.64) | 3 | 29.96 (24.2, 35.73) | 3 | 33.63 (26.81, 40.45) | 3 |  | 2.51 (1.1, 3.97) | 2.73(1.36, 4.13) |
| Tokelau |  | 3.22 (2.03, 4.41) | 12 | 3.56 (2.5, 4.62) | 17 | 3.93 (2.77, 5.1) | 16 |  | 0.35 (-1.94, 2.88) | 0.64(-1.49, 3.02) |
| Tonga |  | 6.88 (4.81, 8.95) | 4 | 7.71 (5.96, 9.46) | 8 | 7.82 (6.01, 9.63) | 8 |  | 0.39 (-1.39, 2.36) | 0.41(-1.28, 2.26) |
| Tuvalu |  | 2.91 (1.97, 3.84) | 15 | 3.2 (2.35, 4.05) | 18 | 3.2 (2.36, 4.05) | 19 |  | 0.33 (-1.68, 2.52) | 0.31(-1.56, 2.35) |
| Vanuatu |  | 1.36 (0.72, 2) | 27 | 1.76 (1.27, 2.26) | 28 | 1.8 (1.29, 2.31) | 28 |  | 0.89 (-1.55, 4.02) | 0.91(-1.4, 3.83) |
| Viet Nam |  | 3.64 (2.86, 4.43) | 11 | 6.27 (4.81, 7.74) | 9 | 6.66 (5.1, 8.23) | 9 |  | 1.89 (0.28, 3.49) | 1.97(0.46, 3.47) |
| Western Pacific Region |  | 5.88 (5.32, 6.44) |  | 10.83 (9.64, 12.02) |  | 10.97 (9.7, 12.23) |  |  | 2.13 (1.4, 2.85) | 2.03(1.33, 2.72) |

Abbreviation: UI, Uncertainty interval; EAPC, Estimated annual percentage change; CI, Confidence interval

Table S7. Age-standardized liver cancer mortality rates (per 100,000) in the Western Pacific region by member state, in 1990, 2019, and 2021.

| **Location** |  | **Age-standardized mortality rate, per 100, 000 (95% UI)** | | | | | |  | **EAPC, % (95%CI)** | |
| --- | --- | --- | --- | --- | --- | --- | --- | --- | --- | --- |
|  |  | **1990** | **Rank** | **2019** | **Rank** | **2021** | **Rank** |  | **1990-2019** | **1990-2021** |
| Western Pacific region |  | 8.76 (7.92, 9.6) |  | 12.54 (10.91, 14.16) |  | 12.59 (10.99, 14.18) |  |  | 1.24 (1.11, 1.35) | 1.18 (1.06, 1.27) |
| **Aged countries** |  |  |  |  |  |  |  |  |  |  |
| South Korea |  | 24.91 (19.93, 29.82) | 2 | 23.81 (20.07, 27.6) | 2 | 26.5 (21.95, 31.05) | 2 |  | -0.16 (-0.27, 0.02) | 0.2 (0.13, 0.31) |
| Cook Islands |  | 6.83 (5.45, 8.25) | 7 | 14.1 (11.36, 16.9) | 5 | 15.11 (12.01, 18.18) | 5 |  | 2.53 (2.5, 2.57) | 2.59 (2.58, 2.58) |
| Japan |  | 16.12 (15.34, 16.93) | 3 | 23.03 (20.82, 25.19) | 3 | 24.34 (21.87, 26.79) | 3 |  | 1.24 (1.06, 1.38) | 1.34 (1.15, 1.49) |
| China |  | 7.93 (6.92, 8.94) | 6 | 12.26 (10.24, 14.28) | 6 | 12.08 (10.15, 14.05) | 7 |  | 1.51 (1.36, 1.63) | 1.37 (1.24, 1.47) |
| Australia |  | 2.11 (1.8, 2.43) | 26 | 8.51 (7.26, 9.73) | 9 | 8.82 (7.49, 10.12) | 9 |  | 4.93 (4.9, 4.93) | 4.72 (4.71, 4.71) |
| Singapore |  | 6.26 (5.43, 7.1) | 8 | 7.64 (6.48, 8.82) | 10 | 8.37 (7.04, 9.7) | 10 |  | 0.69 (0.61, 0.75) | 0.94 (0.84, 1.01) |
| New Zealand |  | 2.29 (2.11, 2.48) | 23 | 5.87 (5.39, 6.35) | 16 | 6.11 (5.59, 6.62) | 16 |  | 3.3 (3.29, 3.3) | 3.22 (3.19, 3.22) |
| **Aging countries** |  |  |  |  |  |  |  |  |  |  |
| Viet Nam |  | 8.28 (6.36, 10.18) | 5 | 12.03 (9.06, 14.98) | 7 | 12.59 (9.32, 15.83) | 6 |  | 1.3 (1.23, 1.34) | 1.36 (1.24, 1.43) |
| Palau |  | 4.95 (3.45, 6.44) | 11 | 9.28 (7, 11.63) | 8 | 9.98 (7.4, 12.53) | 8 |  | 2.19 (2.06, 2.47) | 2.29 (2.17, 2.49) |
| Northern Mariana Islands |  | 2.23 (1.68, 2.78) | 24 | 6.42 (5.08, 7.71) | 13 | 7.2 (5.84, 8.57) | 12 |  | 3.71 (3.58, 3.89) | 3.85 (3.7, 4.1) |
| Guam |  | 2.04 (1.59, 2.49) | 27 | 6.61 (5.57, 7.66) | 12 | 7.15 (5.94, 8.34) | 13 |  | 4.14 (3.95, 4.42) | 4.13 (3.98, 4.34) |
| American Samoa |  | 1.85 (1.56, 2.14) | 29 | 5.9 (4.74, 7.04) | 15 | 6.26 (4.94, 7.55) | 15 |  | 4.08 (3.91, 4.19) | 4.01 (3.79, 4.15) |
| Niue |  | 4.11 (3.04, 5.19) | 12 | 6.35 (4.59, 8.08) | 14 | 6.47 (4.66, 8.27) | 14 |  | 1.51 (1.43, 1.54) | 1.47 (1.39, 1.51) |
| Malaysia |  | 2.33 (1.9, 2.77) | 22 | 4.96 (4.06, 5.85) | 17 | 5.08 (4.07, 6.1) | 17 |  | 2.64 (2.61, 2.65) | 2.55 (2.49, 2.58) |
| Tokelau |  | 3.26 (2.09, 4.44) | 18 | 4.6 (3.22, 5.98) | 19 | 5.07 (3.51, 6.62) | 18 |  | 1.19 (1.03, 1.5) | 1.43 (1.3, 1.69) |
| Tuvalu |  | \| 3.3 (2.37, 4.26) \| \| --- \| | 17 | 4.01 (2.96, 5.06) | 22 | 4.03 (2.92, 5.16) | 22 |  | 0.67 (0.6, 0.77) | 0.65 (0.62, 0.68) |
| **Young countries** |  |  |  |  |  |  |  |  |  |  |
| Mongolia |  | 29.54 (22.32, 36.77) | 1 | 50.74 (40.11, 61.14) | 1 | 53.13 (42.21, 63.95) | 1 |  | 1.88 (1.77, 2.04) | 1.91 (1.8, 2.08) |
| Tonga |  | 12.28 (9.22, 15.37) | 4 | 15.73 (12.1, 19.36) | 4 | 15.94 (12.14, 19.7) | 4 |  | 0.86 (0.8, 0.94) | 0.85 (0.8, 0.89) |
| Brunei Darussalam |  | 5.8 (4.44, 7.18) | 9 | 6.63 (5.31, 7.95) | 11 | 7.37 (5.84, 8.93) | 11 |  | 0.46 (0.35, 0.62) | 0.78 (0.71, 0.89) |
| Cambodia |  | 3.91 (2.34, 5.55) | 13 | 4.7 (2.86, 6.57) | 18 | 4.91 (3.01, 6.79) | 19 |  | 0.64 (0.58, 0.69) | 0.74 (0.65, 0.82) |
| Vanuatu |  | 2.03 (1.17, 2.88) | 28 | 4.5 (4.08, 4.94) | 20 | 4.58 (4.01, 5.16) | 20 |  | 2.78 (1.88, 4.4) | 2.66 (1.9, 4.05) |
| Laos |  | 5.3 (3.93, 6.62) | 10 | 4.3 (3.3, 5.3) | 21 | 4.42 (3.34, 5.5) | 21 |  | -0.72 (-0.76, -0.6) | -0.58 (-0.6, -0.52) |
| Kiribati |  | 3.55 (2.8, 4.28) | 14 | 3.81 (2.87, 4.74) | 24 | 3.88 (2.94, 4.86) | 24 |  | 0.24 (0.09, 0.35) | 0.29 (0.16, 0.41) |
| Federated States of Micronesia |  | 2.46 (1.75, 3.18) | 21 | 3.7 (2.43, 4.96) | 25 | 3.86 (2.54, 5.2) | 25 |  | 1.42 (1.14, 1.54) | 1.46 (1.21, 1.6) |
| Nauru |  | 3.54 (2.73, 4.36) | 16 | 3.3 (2.26, 4.34) | 26 | 3.38 (2.28, 4.46) | 26 |  | -0.24 (-0.65, -0.02) | -0.15 (-0.58, 0.07) |
| Fiji |  | 2.13 (1.57, 2.69) | 25 | 3.84 (2.99, 4.71) | 23 | 4.01 (3.04, 4.97) | 23 |  | 2.05 (1.95, 2.25) | 2.06 (2, 2.15) |
| Samoa |  | 2.72 (2.15, 3.3) | 20 | 3.2 (2.53, 3.87) | 27 | 3.25 (2.52, 3.99) | 27 |  | 0.56 (0.55, 0.56) | 0.58 (0.51, 0.61) |
| Solomon Islands |  | 2.79 (1.06, 4.57) | 19 | 2.62 (1.72, 3.5) | 28 | 2.64 (1.74, 3.53) | 28 |  | -0.22 (-0.92, 1.68) | -0.18 (-0.83, 1.61) |
| Philippines |  | 3.55 (2.9, 4.21) | 15 | 2.43 (1.47, 3.39) | 29 | 2.57 (1.54, 3.6) | 29 |  | -1.3 (-2.32, -0.74) | -1.04 (-2.02, -0.5) |
| Marshall Islands |  | 1.26 (0.76, 1.75) | 31 | 2.41 (1.67, 3.15) | 30 | 2.48 (1.7, 3.26) | 30 |  | 2.26 (2.05, 2.75) | 2.21 (2.03, 2.63) |
| Papua New Guinea |  | 1.76 (0.65, 2.9) | 30 | 1.26 (0.4, 2.1) | 31 | 1.3 (0.43, 2.19) | 31 |  | -1.15 (-1.66, -1.11) | -0.97 (-1.32, -0.9) |

Abbreviation: UI, Uncertainty interval, EAPC, Estimated annual percentage change; CI, Confidence interval

Table S8. Age-standardized liver cancer mortality rates (per 100,000) in the Western Pacific region by member state and sex, in 1990, 2019, and 2021.

| **Location** |  | **Age-standardized mortality rate, per 100, 000 (95% UI)** | | | | | |  | **EAPC, % (95%CI)** | |
| --- | --- | --- | --- | --- | --- | --- | --- | --- | --- | --- |
|  |  | **1990** | **Rank** | **2019** | **Rank** | **2021** | **Rank** |  | **1990-2019** | **1990-2021** |
| **Male** |  |  |  |  |  |  |  |  |  |  |
| American Samoa |  | 2.49 (1.84, 3.14) | 29 | 7.01 (5.44, 8.58) | 17 | 7.42 (5.71, 9.13) | 16 |  | 3.63 (1.91, 5.45) | 3.59(1.95, 5.3) |
| Australia |  | 3.07 (2.58, 3.56) | 23 | 11.67 (9.87, 13.47) | 9 | 12.17 (10.22, 14.11) | 9 |  | 4.71 (3.58, 5.86) | 4.54(3.46, 5.63) |
| Brunei Darussalam |  | 7.55 (5.74, 9.37) | 11 | 8.35 (6.56, 10.14) | 14 | 9.24 (7.23, 11.26) | 13 |  | 0.35 (-1.22, 1.98) | 0.65(-0.83, 2.2) |
| Cambodia |  | 4.14 (1.81, 6.47) | 16 | 4.9 (2.07, 7.74) | 23 | 5.13 (2.13, 8.13) | 22 |  | 0.58 (-3.85, 5.14) | 0.69(-3.52, 4.97) |
| China |  | 11.12 (9.3, 12.94) | 6 | 17.12 (13.48, 20.76) | 7 | 16.8 (13.19, 20.42) | 8 |  | 1.5 (0.14, 2.81) | 1.34(0.06, 2.57) |
| Cook Islands |  | 8.48 (6.51, 10.46) | 9 | 20.74 (16.28, 25.21) | 5 | 22.32 (17.4, 27.23) | 5 |  | 3.13 (1.54, 4.78) | 3.17(1.66, 4.72) |
| Federated States of Micronesia |  | 3.04 (1.84, 4.23) | 24 | 4.65 (2.55, 6.76) | 25 | 4.92 (2.64, 7.2) | 24 |  | 1.48 (-1.73, 4.59) | 1.57(-1.51, 4.5) |
| Fiji |  | 2.72 (1.92, 3.53) | 27 | 4.93 (3.59, 6.27) | 22 | 5.14 (3.68, 6.61) | 21 |  | 2.07 (0.06, 4.17) | 2.07(0.13, 4.07) |
| Guam |  | 2.54 (2.14, 2.94) | 28 | 10.56 (8.79, 12.32) | 11 | 11.3 (9.29, 13.3) | 11 |  | 5.04 (3.85, 6.22) | 4.93(3.78, 6.07) |
| Japan |  | 24.38 (23.14, 25.62) | 3 | 30.77 (28.71, 32.84) | 3 | 32.53 (30.21, 34.85) | 3 |  | 0.81 (0.39, 1.21) | 0.93(0.53, 1.33) |
| Kiribati |  | 3.46 (2.63, 4.29) | 19 | 3.39 (2.18, 4.6) | 28 | 3.46 (2.24, 4.67) | 28 |  | -0.07 (-2.31, 1.95) | 0(-2.07, 1.87) |
| Laos |  | 7.64 (5.46, 9.81) | 10 | 5.97 (4.33, 7.6) | 19 | 6.16 (4.44, 7.89) | 20 |  | -0.85 (-2.78, 1.15) | -0.69(-2.52, 1.19) |
| Malaysia |  | 3.23 (2.54, 3.92) | 21 | 7.24 (5.8, 8.68) | 16 | 7.41 (5.8, 9.03) | 17 |  | 2.82 (1.36, 4.33) | 2.71(1.27, 4.18) |
| Marshall Islands |  | 1.34 (0.71, 1.97) | 31 | 2.81 (1.27, 4.34) | 30 | 2.99 (1.28, 4.69) | 30 |  | 2.59 (-1.5, 6.44) | 2.62(-1.38, 6.28) |
| Mongolia |  | 37.96 (28.05, 47.88) | 1 | 56.46 (43.67, 69.24) | 1 | 58.94 (45.56, 72.32) | 1 |  | 1.38 (-0.32, 3.16) | 1.43(-0.16, 3.1) |
| Nauru |  | 5.09 (3.76, 6.42) | 13 | 4.67 (2.88, 6.46) | 24 | 4.76 (2.88, 6.63) | 25 |  | -0.3 (-2.73, 1.88) | -0.22(-2.55, 1.85) |
| New Zealand |  | 3.14 (2.85, 3.44) | 22 | 7.73 (7, 8.46) | 15 | 8.04 (7.24, 8.84) | 15 |  | 3.16 (2.48, 3.82) | 3.08(2.43, 3.72) |
| Niue |  | 5.04 (3.52, 6.56) | 14 | 8.86 (5.79, 11.93) | 12 | 9.14 (5.98, 12.3) | 14 |  | 1.96 (-0.43, 4.3) | 1.94(-0.3, 4.12) |
| Northern Mariana Islands |  | 2.84 (2.04, 3.64) | 25 | 8.84 (6.89, 10.78) | 13 | 9.91 (7.85, 11.98) | 12 |  | 3.99 (2.22, 5.91) | 4.11(2.51, 5.88) |
| Palau |  | 9.59 (6.7, 12.49) | 7 | 16.95 (12.77, 21.14) | 8 | 18.07 (13.44, 22.71) | 7 |  | 1.98 (0.08, 4.04) | 2.06(0.24, 4.02) |
| Papua New Guinea |  | 2.24 (0.49, 4) | 30 | 1.6 (0.18, 3.03) | 31 | 1.65 (0.22, 3.08) | 31 |  | -1.15 (-10.14, 6.48) | -0.98(-8.93, 6.11) |
| Philippines |  | 5.48 (4.42, 6.54) | 12 | 6.41 (5.68, 7.14) | 18 | 6.51 (5.44, 7.58) | 18 |  | 0.54 (-0.48, 1.67) | 0.56(-0.59, 1.76) |
| Samoa |  | 3.94 (3.05, 4.84) | 17 | 4.49 (3.4, 5.57) | 26 | 4.58 (3.44, 5.72) | 26 |  | 0.45 (-1.21, 2.1) | 0.49(-1.1, 2.05) |
| Singapore |  | 9.23 (8.03, 10.42) | 8 | 10.82 (9.11, 12.54) | 10 | 11.88 (9.99, 13.78) | 10 |  | 0.55 (-0.46, 1.55) | 0.82(-0.14, 1.76) |
| Solomon Islands |  | 4.29 (1.4, 7.19) | 15 | 3.76 (2.25, 5.27) | 27 | 3.8 (2.27, 5.32) | 27 |  | -0.45 (-3.93, 4.68) | -0.39(-3.65, 4.4) |
| South Korea |  | 36.58 (28.28, 44.87) | 2 | 34.77 (28.98, 40.57) | 2 | 38.69 (31.53, 45.84) | 2 |  | -0.17 (-1.5, 1.25) | 0.18(-1.13, 1.57) |
| Tokelau |  | 3.29 (1.78, 4.81) | 20 | 5.91 (3.89, 7.93) | 20 | 6.49 (4.22, 8.76) | 19 |  | 2.04 (-0.73, 5.29) | 2.22(-0.42, 5.28) |
| Tonga |  | 18.28 (13.36, 23.19) | 4 | 24.29 (17.86, 30.73) | 4 | 24.72 (18.13, 31.31) | 4 |  | 0.99 (-0.9, 2.91) | 0.98(-0.79, 2.79) |
| Tuvalu |  | 3.93 (2.62, 5.25) | 18 | 4.95 (3.32, 6.58) | 21 | 5.01 (3.29, 6.73) | 23 |  | 0.8 (-1.57, 3.23) | 0.79(-1.5, 3.09) |
| Vanuatu |  | 2.8 (1.47, 4.13) | 26 | 3.22 (1.98, 4.47) | 29 | 3.34 (2.04, 4.64) | 29 |  | 0.48 (-2.5, 3.91) | 0.57(-2.25, 3.78) |
| Viet Nam |  | 13.21 (9.89, 16.53) | 5 | 18.69 (13.57, 23.81) | 6 | 19.62 (13.92, 25.31) | 6 |  | 1.2 (-0.68, 3.08) | 1.28(-0.55, 3.08) |
| Western Pacific Region |  | 12.49 (10.95, 14.04) |  | 17.48 (14.64, 20.32) |  | 17.49 (14.63, 20.36) |  |  | 1.17 (0.14, 2.15) | 1.09(0.13, 2.02) |
| **Female** |  |  |  |  |  |  |  |  |  |  |
| American Samoa |  | 1.57 (1.19, 1.95) | 20 | 4.75 (3.75, 5.74) | 9 | 5.03 (3.94, 6.13) | 10 |  | 3.89 (2.28, 5.58) | 3.83(2.29, 5.43) |
| Australia |  | 1.16 (0.99, 1.34) | 29 | 5.41 (4.52, 6.31) | 7 | 5.55 (4.6, 6.49) | 8 |  | 5.45 (4.28, 6.6) | 5.18(4.06, 6.25) |
| Brunei Darussalam |  | 3.8 (2.82, 4.79) | 7 | 4.71 (3.59, 5.82) | 10 | 5.28 (3.9, 6.67) | 9 |  | 0.74 (-0.99, 2.53) | 1.07(-0.66, 2.82) |
| Cambodia |  | 3.69 (1.98, 5.39) | 8 | 4.51 (2.93, 6.09) | 11 | 4.71 (3.12, 6.3) | 12 |  | 0.69 (-2.08, 3.95) | 0.79(-1.75, 3.8) |
| China |  | 4.55 (3.98, 5.12) | 6 | 7.17 (6.21, 8.13) | 5 | 7.13 (6.11, 8.14) | 6 |  | 1.58 (0.67, 2.49) | 1.46(0.57, 2.33) |
| Cook Islands |  | 5.05 (3.96, 6.13) | 5 | 7.79 (6.18, 9.4) | 4 | 8.34 (6.52, 10.16) | 4 |  | 1.51 (0.03, 3.03) | 1.63(0.2, 3.09) |
| Federated States of Micronesia |  | 1.53 (1.05, 2.02) | 21 | 2.72 (2.14, 3.29) | 19 | 2.84 (2.21, 3.48) | 20 |  | 2 (0.2, 4.02) | 2.02(0.29, 3.94) |
| Fiji |  | 1.06 (0.88, 1.25) | 30 | 2.54 (2.12, 2.97) | 22 | 2.89 (2.31, 3.46) | 19 |  | 3.06 (1.84, 4.28) | 3.29(2, 4.52) |
| Guam |  | 8.14 (7.64, 8.64) | 3 | 15.64 (13.08, 18.2) | 2 | 16.61 (13.84, 19.38) | 2 |  | 2.28 (1.44, 3.04) | 2.33(1.53, 3.05) |
| Japan |  | 3.63 (2.84, 4.42) | 9 | 4.21 (3.23, 5.18) | 13 | 4.29 (3.26, 5.31) | 13 |  | 0.51 (-1.08, 2.09) | 0.54(-0.98, 2.04) |
| Kiribati |  | 3.03 (2.3, 3.76) | 14 | 2.58 (1.99, 3.17) | 21 | 2.66 (2.03, 3.28) | 22 |  | -0.55 (-2.17, 1.11) | -0.42(-1.97, 1.15) |
| Laos |  | 1.42 (1.14, 1.7) | 23 | 2.53 (2.06, 3) | 24 | 2.6 (2.09, 3.11) | 23 |  | 2.01 (0.66, 3.39) | 1.97(0.67, 3.29) |
| Malaysia |  | 1.17 (0.73, 1.61) | 28 | 2.03 (1.46, 2.6) | 25 | 2.13 (1.51, 2.74) | 25 |  | 1.92 (-0.34, 4.48) | 1.95(-0.21, 4.36) |
| Marshall Islands |  | 1.87 (1.41, 2.33) | 17 | 2.7 (1.88, 3.52) | 20 | 2.8 (1.94, 3.66) | 21 |  | 1.27 (-0.74, 3.21) | 1.31(-0.59, 3.12) |
| Mongolia |  | 21.1 (15.32, 26.89) | 1 | 45.32 (36.42, 54.22) | 1 | 47.55 (37.74, 57.35) | 1 |  | 2.67 (1.05, 4.45) | 2.66(1.1, 4.35) |
| Nauru |  | 1.87 (1.45, 2.29) | 16 | 1.9 (1.33, 2.47) | 26 | 1.97 (1.39, 2.56) | 26 |  | 0.05 (-1.86, 1.85) | 0.17(-1.6, 1.85) |
| New Zealand |  | 1.47 (1.34, 1.6) | 22 | 4.04 (3.64, 4.43) | 14 | 4.17 (3.76, 4.58) | 15 |  | 3.55 (2.87, 4.21) | 3.42(2.79, 4.04) |
| Niue |  | 3.17 (2.28, 4.06) | 13 | 3.77 (2.74, 4.8) | 15 | 3.78 (2.78, 4.79) | 16 |  | 0.6 (-1.35, 2.6) | 0.57(-1.21, 2.42) |
| Northern Mariana Islands |  | 1.57 (1.22, 1.91) | 19 | 3.75 (2.92, 4.58) | 16 | 4.22 (3.35, 5.08) | 14 |  | 3.05 (1.47, 4.67) | 3.24(1.83, 4.71) |
| Palau |  | 0.01 (0.01, 0.01) | 31 | 0.02 (0.01, 0.02) | 31 | 0.02 (0.01, 0.02) | 31 |  | 2.42 (0, 2.42) | 2.26(0, 2.26) |
| Papua New Guinea |  | 1.23 (0.56, 1.91) | 25 | 0.9 (0.5, 1.31) | 30 | 0.92 (0.49, 1.35) | 30 |  | -1.07 (-4.52, 2.97) | -0.93(-4.29, 2.88) |
| Philippines |  | 1.6 (1.19, 2.01) | 18 | 2.53 (2.3, 2.77) | 23 | 2.59 (2.25, 2.92) | 24 |  | 1.59 (0.47, 2.96) | 1.57(0.36, 2.94) |
| Samoa |  | 1.36 (1.07, 1.66) | 24 | 1.84 (1.4, 2.29) | 27 | 1.84 (1.4, 2.29) | 27 |  | 1.05 (-0.59, 2.66) | 0.98(-0.55, 2.48) |
| Singapore |  | 3.25 (2.71, 3.79) | 11 | 4.33 (3.55, 5.11) | 12 | 4.73 (3.85, 5.61) | 11 |  | 0.99 (-0.23, 2.21) | 1.22(0.05, 2.37) |
| Solomon Islands |  | 1.18 (0.55, 1.81) | 27 | 1.43 (0.99, 1.88) | 29 | 1.44 (0.98, 1.9) | 29 |  | 0.66 (-2.06, 4.33) | 0.64(-1.96, 4.08) |
| South Korea |  | 13.11 (10.36, 15.87) | 2 | 12.7 (10.21, 15.18) | 3 | 14.24 (11.33, 17.15) | 3 |  | -0.11 (-1.51, 1.33) | 0.27(-1.08, 1.64) |
| Tokelau |  | 3.22 (2, 4.45) | 12 | 3.27 (2.28, 4.25) | 17 | 3.57 (2.48, 4.66) | 17 |  | 0.05 (-2.28, 2.63) | 0.33(-1.87, 2.77) |
| Tonga |  | 6.19 (4.28, 8.11) | 4 | 7.16 (5.52, 8.79) | 6 | 7.24 (5.57, 8.91) | 5 |  | 0.5 (-1.32, 2.51) | 0.51(-1.2, 2.39) |
| Tuvalu |  | 2.71 (1.8, 3.62) | 15 | 2.99 (2.21, 3.77) | 18 | 2.98 (2.2, 3.77) | 18 |  | 0.34 (-1.69, 2.58) | 0.31(-1.59, 2.41) |
| Vanuatu |  | 1.2 (0.65, 1.75) | 26 | 1.56 (1.13, 1.99) | 28 | 1.6 (1.14, 2.07) | 28 |  | 0.91 (-1.5, 3.93) | 0.93(-1.37, 3.81) |
| Viet Nam |  | 3.6 (2.83, 4.38) | 10 | 5.37 (4.15, 6.59) | 8 | 5.62 (4.32, 6.92) | 7 |  | 1.39 (-0.19, 2.96) | 1.45(-0.04, 2.93) |
| Western Pacific Region |  | 4.85 (4.34, 5.37) |  | 7.43 (6.58, 8.27) |  | 7.51 (6.59, 8.43) |  |  | 1.48 (0.7, 2.25) | 1.42(0.66, 2.16) |

Abbreviation: UI, Uncertainty interval; EAPC, Estimated annual percentage change; CI, Confidence interval

Table S9. Age-standardized DALYs rates (per 100,000) for specific liver cancer in the Western Pacific region by member state and sex, in 1990 to 2021.

| **Region/Liver cancer cause** |  | **Both sexes combined** | | |  | **Male** | | |  | **Female** | | |
| --- | --- | --- | --- | --- | --- | --- | --- | --- | --- | --- | --- | --- |
|  |  | **1990 (95% UI)** | **2021 (95% UI)** | **EAPC, %**  **(95% CI)** |  | **1990 (95% UI)** | **2021 (95% UI)** | **EAPC, %**  **(95% CI)** |  | **1990 (95% UI)** | **2021 (95% UI)** | **EAPC, %**  **(95% CI)** |
| **American Samoa** |  |  |  |  |  |  |  |  |  |  |  |  |
| LCHB |  | 39.99 (28.72, 55.68) | 106.07 (75.47, 143.36) | 3.15 (0.98, 5.19) |  | 55.97 (39.07, 80.23) | 148.96 (103.26, 207.76) | 3.16 (0.81, 5.39) |  | 23.06 (15.45, 35.02) | 61.17 (41.49, 91.74) | 3.15 (0.55, 5.75) |
| LCHC |  | 9.9 (6.12, 15.55) | 30.89 (20.12, 46.17) | 3.67 (0.83, 6.52) |  | 8.54 (5.03, 14.24) | 25.88 (15.97, 39.84) | 3.58 (0.37, 6.68) |  | 11.33 (6.9, 17.89) | 36.15 (23.27, 54.32) | 3.74 (0.85, 6.66) |
| LCAL |  | 6.06 (3.59, 9.52) | 17.93 (11.16, 27.56) | 3.5 (0.51, 6.57) |  | 8.91 (5.33, 14.49) | 26.19 (15.8, 41.4) | 3.48 (0.28, 6.61) |  | 3.04 (1.83, 4.96) | 9.28 (5.46, 15.14) | 3.6 (0.31, 6.82) |
| LCNA |  | 7.35 (4.8, 10.96) | 24.66 (16.24, 35.77) | 3.9 (1.27, 6.48) |  | 6.38 (3.93, 10.1) | 21.32 (13.08, 33.07) | 3.89 (0.83, 6.87) |  | 8.37 (5.4, 12.78) | 28.15 (17.92, 42.67) | 3.91 (1.09, 6.67) |
| LCOT |  | 3.24 (2.07, 5.01) | 7.92 (4.9, 12.05) | 2.88 (-0.07, 5.68) |  | 2.45 (1.49, 4) | 6.09 (3.77, 9.89) | 2.94 (-0.19, 6.11) |  | 4.08 (2.47, 6.38) | 9.85 (5.89, 15.64) | 2.84 (-0.26, 5.95) |
| **Australia** |  |  |  |  |  |  |  |  |  |  |  |  |
| LCHB |  | 10.43 (7.46, 14.13) | 27.59 (18.44, 38.83) | 3.14 (0.86, 5.32) |  | 16.95 (12.08, 23.14) | 44.14 (29.29, 62.29) | 3.09 (0.76, 5.29) |  | 3.99 (2.81, 5.35) | 11.43 (7.55, 16.47) | 3.4 (1.11, 5.7) |
| LCHC |  | 14.01 (10.24, 18.44) | 59.45 (44.39, 77.7) | 4.66 (2.83, 6.54) |  | 16.47 (11.4, 22.95) | 67.43 (47.53, 93.23) | 4.55 (2.35, 6.78) |  | 11.58 (8.89, 14.7) | 51.67 (39.49, 65.65) | 4.82 (3.19, 6.45) |
| LCAL |  | 22.93 (18.01, 28.01) | 79.72 (61.8, 98.91) | 4.02 (2.55, 5.49) |  | 39.58 (31.23, 48.82) | 136 (106.92, 170.11) | 3.98 (2.53, 5.47) |  | 6.47 (4.66, 8.79) | 24.75 (17.13, 34.01) | 4.33 (2.15, 6.41) |
| LCNA |  | 4.16 (2.95, 5.67) | 21.06 (14.53, 29.22) | 5.23 (3.04, 7.4) |  | 4.67 (3.17, 6.76) | 23.24 (15.3, 32.8) | 5.18 (2.63, 7.54) |  | 3.65 (2.52, 4.85) | 18.93 (12.83, 26.32) | 5.31 (3.14, 7.57) |
| LCOT |  | 2.62 (1.88, 3.49) | 9.12 (6.4, 12.48) | 4.02 (1.96, 6.11) |  | 2.72 (1.81, 3.74) | 9.23 (6.19, 13.2) | 3.94 (1.63, 6.41) |  | 2.51 (1.81, 3.43) | 9.01 (6.15, 12.47) | 4.12 (1.88, 6.23) |
| **Brunei Darussalam** |  |  |  |  |  |  |  |  |  |  |  |  |
| LCHB |  | 101.73 (71.84, 141.23) | 114.19 (81.64, 157.88) | 0.37 (-1.77, 2.54) |  | 154.78 (108.5, 216.04) | 174.45 (126.52, 238.49) | 0.39 (-1.73, 2.54) |  | 41.86 (28.7, 61.14) | 46.88 (30.16, 71.84) | 0.37 (-2.28, 2.96) |
| LCHC |  | 42.28 (25.21, 64.29) | 60.81 (39.43, 86.89) | 1.17 (-1.58, 3.99) |  | 44.17 (24.4, 68.23) | 61.4 (37.55, 95.3) | 1.06 (-1.93, 4.39) |  | 40.15 (23.77, 61.43) | 60.15 (37.28, 92.1) | 1.3 (-1.61, 4.37) |
| LCAL |  | 13.02 (7.72, 21.17) | 16.65 (10.55, 26.06) | 0.79 (-2.25, 3.92) |  | 20.21 (11.93, 32.89) | 25.43 (15.97, 40.11) | 0.74 (-2.33, 3.91) |  | 4.91 (2.81, 8.04) | 6.84 (4.08, 11.33) | 1.07 (-2.19, 4.5) |
| LCNA |  | 8.03 (4.85, 13.06) | 12.1 (7.67, 18.71) | 1.32 (-1.72, 4.36) |  | 8.14 (4.71, 13.65) | 11.63 (7.13, 18.32) | 1.15 (-2.09, 4.38) |  | 7.9 (4.77, 12.91) | 12.61 (7.58, 20.35) | 1.51 (-1.72, 4.68) |
| LCOT |  | 6.43 (3.95, 10.01) | 8.27 (5.23, 13.4) | 0.81 (-2.09, 3.94) |  | 6.46 (3.69, 10.54) | 8.11 (4.82, 13.4) | 0.73 (-2.52, 4.16) |  | 6.39 (4.12, 10.02) | 8.44 (5.1, 13.93) | 0.9 (-2.18, 3.93) |
| **Cambodia** |  |  |  |  |  |  |  |  |  |  |  |  |
| LCHB |  | 64.2 (33.96, 124.14) | 59.94 (28.99, 117.39) | -0.22 (-4.69, 4) |  | 78.56 (38.6, 182.95) | 76.02 (30.23, 184.68) | -0.11 (-5.81, 5.05) |  | 51.22 (21.59, 99.9) | 44.42 (20.25, 76.88) | -0.46 (-5.15, 4.1) |
| LCHC |  | 27.26 (13.07, 51.9) | 31.87 (16.41, 58.6) | 0.5 (-3.71, 4.84) |  | 20.97 (9.33, 51) | 23.18 (9.1, 60.01) | 0.32 (-5.56, 6) |  | 32.93 (13.03, 66.73) | 40.25 (18.79, 71.05) | 0.65 (-4.09, 5.47) |
| LCAL |  | 16.65 (7.55, 35.34) | 33.97 (15.92, 70.43) | 2.3 (-2.57, 7.2) |  | 23.89 (10.11, 55.46) | 47.82 (19.07, 118.22) | 2.24 (-3.44, 7.93) |  | 10.1 (3.92, 23.17) | 20.6 (9.05, 39.85) | 2.3 (-3.03, 7.48) |
| LCNA |  | 9.18 (4.25, 17.96) | 11.9 (5.87, 22.03) | 0.84 (-3.61, 5.31) |  | 6.49 (2.67, 15) | 8.74 (3.38, 21.14) | 0.96 (-4.81, 6.67) |  | 11.6 (4.51, 24.21) | 14.96 (7.13, 27.5) | 0.82 (-3.94, 5.83) |
| LCOT |  | 4.82 (2.44, 9.48) | 4.78 (2.33, 9.44) | -0.03 (-4.53, 4.36) |  | 3.27 (1.48, 7.77) | 3.48 (1.34, 8.75) | 0.2 (-5.67, 5.73) |  | 6.22 (2.52, 12.6) | 6.02 (2.71, 10.78) | -0.11 (-4.96, 4.69) |
| **China** |  |  |  |  |  |  |  |  |  |  |  |  |
| LCHB |  | 190.07 (156.62, 226.39) | 221.3 (171.7, 288.81) | 0.49 (-0.89, 1.97) |  | 313.22 (251.89, 382.17) | 375.41 (281.12, 508.06) | 0.58 (-0.99, 2.26) |  | 58.87 (46.41, 72.03) | 59.77 (44.17, 79.81) | 0.05 (-1.58, 1.75) |
| LCHC |  | 32.85 (26.28, 40.11) | 52.79 (41.15, 65.63) | 1.53 (0.08, 2.95) |  | 28.66 (21.65, 37.33) | 48.27 (34.99, 67.27) | 1.68 (-0.21, 3.66) |  | 37.32 (28.68, 46.48) | 57.52 (43.72, 74.63) | 1.4 (-0.2, 3.08) |
| LCAL |  | 19.34 (14.84, 24.91) | 33.59 (24.78, 44.83) | 1.78 (-0.02, 3.57) |  | 25.01 (18.65, 33.58) | 45.12 (30.25, 64.46) | 1.9 (-0.34, 4) |  | 13.3 (9.82, 17.3) | 21.5 (15.44, 28.6) | 1.55 (-0.37, 3.45) |
| LCNA |  | 10.64 (8.55, 13.03) | 18.01 (13.66, 22.92) | 1.7 (0.15, 3.18) |  | 11.1 (8.54, 14.24) | 19.74 (13.81, 27.06) | 1.86 (-0.1, 3.72) |  | 10.14 (7.79, 12.59) | 16.2 (12.1, 21.89) | 1.51 (-0.13, 3.33) |
| LCOT |  | 15.38 (12.37, 19.13) | 16.69 (12.7, 21.82) | 0.26 (-1.32, 1.83) |  | 15.41 (11.93, 19.7) | 18.42 (13.06, 26.42) | 0.58 (-1.33, 2.56) |  | 15.35 (11.62, 19.52) | 14.87 (10.89, 19.81) | -0.1 (-1.88, 1.72) |
| **Cook Islands** |  |  |  |  |  |  |  |  |  |  |  |  |
| LCHB |  | 118.66 (84.84, 160.9) | 182.32 (125.44, 261.02) | 1.39 (-0.8, 3.63) |  | 168.45 (118.57, 239.06) | 308.46 (206.73, 447.85) | 1.95 (-0.47, 4.29) |  | 64.66 (43.84, 90.65) | 63.25 (41.01, 92.95) | -0.07 (-2.56, 2.42) |
| LCHC |  | 34.33 (21.8, 51.35) | 64.74 (41.92, 94.96) | 2.05 (-0.65, 4.75) |  | 31.1 (18.31, 50.69) | 73.16 (45.95, 112.54) | 2.76 (-0.32, 5.86) |  | 37.83 (25.07, 55.99) | 56.8 (36.84, 83.65) | 1.31 (-1.35, 3.89) |
| LCAL |  | 21.19 (13.01, 33.54) | 94.66 (60.55, 145.61) | 4.83 (1.91, 7.79) |  | 31.67 (19.2, 50.15) | 162.52 (103.5, 251.47) | 5.28 (2.34, 8.3) |  | 9.83 (5.95, 15.71) | 30.61 (18.49, 46.27) | 3.66 (0.53, 6.62) |
| LCNA |  | 19.96 (13.14, 29.62) | 47.13 (29.34, 70.76) | 2.77 (-0.03, 5.43) |  | 18.31 (11.42, 28.54) | 56.01 (33.03, 86.17) | 3.61 (0.47, 6.52) |  | 21.76 (14.11, 32.12) | 38.74 (23.62, 58.45) | 1.86 (-0.99, 4.58) |
| LCOT |  | 9.2 (6.1, 13.58) | 12.53 (7.72, 19.87) | 1 (-1.82, 3.81) |  | 7.31 (4.46, 11.68) | 14 (8.06, 22.61) | 2.1 (-1.2, 5.24) |  | 11.25 (7.4, 17.32) | 11.14 (6.75, 17.05) | -0.03 (-3.04, 2.69) |
| **Federated States of Micronesia** |  |  |  |  |  |  |  |  |  |  |  |  |
| LCHB |  | 48.77 (31.07, 76.88) | 73.99 (41.98, 127.21) | 1.34 (-1.95, 4.55) |  | 68.52 (39.07, 119.22) | 109.16 (58.16, 209.87) | 1.5 (-2.32, 5.42) |  | 28 (17.44, 41.58) | 37.45 (17.52, 61.97) | 0.94 (-2.79, 4.09) |
| LCHC |  | 12.62 (7.87, 20.29) | 20.93 (11.46, 36.68) | 1.63 (-1.84, 4.97) |  | 10.84 (5.5, 21.15) | 18.82 (9.07, 41.87) | 1.78 (-2.73, 6.55) |  | 14.49 (8.67, 22.94) | 23.12 (10.39, 37.3) | 1.51 (-2.55, 4.71) |
| LCAL |  | 8.74 (4.83, 14.8) | 14.1 (7.47, 27.2) | 1.54 (-2.21, 5.58) |  | 12.86 (6.43, 22.4) | 21.26 (10.1, 44.85) | 1.62 (-2.57, 6.27) |  | 4.41 (2.49, 7.32) | 6.66 (3.16, 11.89) | 1.33 (-2.71, 5.04) |
| LCNA |  | 6.65 (4.09, 10.59) | 12.85 (7.23, 20.51) | 2.12 (-1.23, 5.2) |  | 5.72 (2.92, 10.41) | 11.95 (5.72, 24.37) | 2.38 (-1.93, 6.84) |  | 7.63 (4.59, 12.09) | 13.79 (6.54, 22.44) | 1.91 (-1.98, 5.12) |
| LCOT |  | 4.04 (2.46, 6.29) | 5.84 (3.11, 9.92) | 1.19 (-2.27, 4.5) |  | 3.06 (1.57, 5.86) | 4.86 (2.21, 10.88) | 1.49 (-3.15, 6.24) |  | 5.07 (3.03, 7.92) | 6.86 (3.19, 11.91) | 0.98 (-2.93, 4.42) |
| **Fiji** |  |  |  |  |  |  |  |  |  |  |  |  |
| LCHB |  | 44.98 (31.32, 65.45) | 68.14 (43.86, 97.86) | 1.34 (-1.29, 3.68) |  | 63.39 (42.6, 94.63) | 101.01 (62.75, 152.11) | 1.5 (-1.33, 4.11) |  | 26 (15.87, 43.02) | 34.42 (22.03, 50.21) | 0.9 (-2.16, 3.72) |
| LCHC |  | 10.88 (6.26, 18.8) | 21.87 (13.49, 33.4) | 2.25 (-1.07, 5.4) |  | 9.86 (5.54, 16.64) | 20.42 (11.15, 34.3) | 2.35 (-1.29, 5.88) |  | 11.95 (6.52, 21.81) | 23.36 (15, 34.38) | 2.16 (-1.21, 5.36) |
| LCAL |  | 6.68 (3.89, 10.97) | 14.09 (7.96, 23.46) | 2.41 (-1.03, 5.8) |  | 9.95 (5.6, 16.36) | 21.56 (11.8, 35.82) | 2.49 (-1.05, 5.99) |  | 3.3 (1.75, 6.32) | 6.42 (3.77, 10.34) | 2.15 (-1.67, 5.73) |
| LCNA |  | 5.75 (3.48, 9.27) | 13.23 (8.02, 20.57) | 2.69 (-0.47, 5.73) |  | 5.07 (2.95, 8.36) | 12.58 (6.94, 21.45) | 2.93 (-0.6, 6.4) |  | 6.45 (3.66, 11.52) | 13.9 (8.59, 21.28) | 2.48 (-0.95, 5.68) |
| LCOT |  | 3.63 (2.16, 6.01) | 5.53 (3.3, 8.66) | 1.36 (-1.93, 4.48) |  | 2.74 (1.59, 4.91) | 4.67 (2.51, 8.25) | 1.72 (-2.16, 5.31) |  | 4.54 (2.47, 7.95) | 6.42 (3.86, 9.73) | 1.12 (-2.33, 4.42) |
| **Guam** |  |  |  |  |  |  |  |  |  |  |  |  |
| LCHB |  | 36.45 (29.31, 44.87) | 124.56 (95.75, 153.34) | 3.96 (2.45, 5.34) |  | 55.83 (44.48, 68.14) | 216.88 (165.39, 269.85) | 4.38 (2.86, 5.82) |  | 14.3 (10.84, 18.18) | 29.8 (21.2, 39.74) | 2.37 (0.5, 4.19) |
| LCHC |  | 8.57 (5.57, 12.33) | 30.22 (19.61, 43.42) | 4.07 (1.5, 6.62) |  | 8.95 (5.51, 13.19) | 39.24 (24.56, 58.39) | 4.77 (2.01, 7.61) |  | 8.14 (5.37, 11.4) | 20.97 (13.44, 28.93) | 3.05 (0.53, 5.43) |
| LCAL |  | 6.54 (4.31, 9.47) | 28.04 (18.14, 42.89) | 4.7 (2.1, 7.41) |  | 10.21 (6.68, 14.89) | 49.09 (31.4, 75.37) | 5.07 (2.41, 7.82) |  | 2.34 (1.52, 3.53) | 6.43 (3.99, 9.98) | 3.26 (0.4, 6.07) |
| LCNA |  | 5.08 (3.61, 7.15) | 21.32 (14.43, 30.78) | 4.63 (2.27, 6.91) |  | 5.5 (3.79, 8.06) | 28.77 (19.14, 43.11) | 5.34 (2.79, 7.84) |  | 4.61 (3.23, 6.45) | 13.67 (9.05, 19.9) | 3.51 (1.09, 5.87) |
| LCOT |  | 2.41 (1.67, 3.45) | 6.77 (4.39, 9.88) | 3.33 (0.78, 5.73) |  | 2.44 (1.6, 3.63) | 8.8 (5.36, 13.68) | 4.14 (1.26, 6.92) |  | 2.38 (1.7, 3.37) | 4.68 (3.08, 6.52) | 2.18 (-0.29, 4.34) |
| **Japan** |  |  |  |  |  |  |  |  |  |  |  |  |
| LCHB |  | 71.95 (61.97, 83.45) | 46.52 (37.75, 55.91) | -1.41 (-2.56, -0.33) |  | 123.05 (106, 143.42) | 73.01 (60.21, 86.79) | -1.68 (-2.8, -0.64) |  | 22.53 (19, 26.09) | 21.36 (16.25, 26.54) | -0.17 (-1.53, 1.08) |
| LCHC |  | 262.57 (246.8, 278.21) | 272.89 (236.51, 297.04) | 0.12 (-0.52, 0.6) |  | 408.95 (382.9, 435.57) | 383 (349.64, 411.62) | -0.21 (-0.71, 0.23) |  | 121.02 (111.62, 128.68) | 168.28 (130.99, 192.3) | 1.06 (0.06, 1.75) |
| LCAL |  | 57.42 (49.33, 68.16) | 45.04 (38.12, 54.16) | -0.78 (-1.87, 0.3) |  | 102.45 (87.72, 121.79) | 77.57 (66.42, 92.72) | -0.9 (-1.96, 0.18) |  | 13.89 (11.74, 16.72) | 14.14 (10.99, 17.64) | 0.06 (-1.35, 1.31) |
| LCNA |  | 16.39 (14.04, 18.97) | 17.64 (13.94, 21.43) | 0.24 (-0.99, 1.36) |  | 21.11 (17.81, 24.76) | 19.35 (15.74, 23.17) | -0.28 (-1.46, 0.85) |  | 11.83 (9.95, 13.84) | 16.01 (11.71, 20.22) | 0.98 (-0.54, 2.29) |
| LCOT |  | 12.39 (10.58, 14.58) | 9.85 (7.95, 11.86) | -0.74 (-1.96, 0.37) |  | 16.62 (13.89, 19.86) | 11.28 (9.33, 13.36) | -1.25 (-2.44, -0.13) |  | 8.3 (7.1, 9.64) | 8.5 (6.36, 10.54) | 0.08 (-1.34, 1.27) |
| **Kiribati** |  |  |  |  |  |  |  |  |  |  |  |  |
| LCHB |  | 72.57 (51.77, 101.08) | 74.49 (50.15, 111.56) | 0.08 (-2.26, 2.48) |  | 84.69 (57.86, 117.33) | 85 (53.09, 139.39) | 0.01 (-2.56, 2.84) |  | 60.65 (41.58, 85.91) | 64.41 (41.88, 98.35) | 0.19 (-2.32, 2.78) |
| LCHC |  | 20.72 (13.76, 29.71) | 24.82 (14.56, 38.16) | 0.58 (-2.3, 3.29) |  | 12.61 (7.48, 19.85) | 13.26 (6.99, 23.69) | 0.16 (-3.37, 3.72) |  | 28.7 (18.73, 42.48) | 35.91 (20.11, 53.82) | 0.72 (-2.41, 3.4) |
| LCAL |  | 9.64 (6.03, 15.38) | 10.61 (6.2, 17.61) | 0.31 (-2.93, 3.46) |  | 12.19 (7.42, 19.96) | 12.61 (6.69, 22.33) | 0.11 (-3.53, 3.55) |  | 7.13 (4.3, 11.27) | 8.69 (5.1, 14.21) | 0.64 (-2.56, 3.86) |
| LCNA |  | 10.64 (6.9, 15.99) | 13.45 (8.48, 19.51) | 0.76 (-2.05, 3.35) |  | 6.39 (4.02, 10.2) | 7.41 (4.16, 12.77) | 0.48 (-2.89, 3.73) |  | 14.83 (9.55, 22.62) | 19.23 (11.66, 28.53) | 0.84 (-2.14, 3.53) |
| LCOT |  | 7.1 (4.69, 10.61) | 7.96 (4.99, 12.33) | 0.37 (-2.43, 3.12) |  | 3.6 (2.2, 5.6) | 3.8 (2.05, 7.25) | 0.17 (-3.24, 3.85) |  | 10.55 (6.72, 15.81) | 11.96 (7.2, 18.81) | 0.4 (-2.54, 3.32) |
| **Laos** |  |  |  |  |  |  |  |  |  |  |  |  |
| LCHB |  | 88 (53.33, 127.67) | 65 (40.87, 97.3) | -0.98 (-3.67, 1.94) |  | 140.21 (82.6, 207.11) | 101.08 (61.42, 154.51) | -1.06 (-3.92, 2.02) |  | 37.64 (24.26, 55.91) | 28.48 (16.9, 43.05) | -0.9 (-3.86, 1.85) |
| LCHC |  | 29.78 (17.87, 46) | 23.36 (14.2, 37.08) | -0.78 (-3.79, 2.35) |  | 32.87 (17.69, 53.69) | 24.57 (13.53, 41.6) | -0.94 (-4.45, 2.76) |  | 26.8 (15.99, 40.68) | 22.13 (13.91, 33.3) | -0.62 (-3.46, 2.37) |
| LCAL |  | 32.98 (19.3, 52.39) | 33.44 (19.14, 51.09) | 0.04 (-3.25, 3.14) |  | 55.68 (31.07, 92.05) | 54.64 (30.74, 85.19) | -0.06 (-3.54, 3.25) |  | 11.09 (6.72, 17.35) | 11.98 (6.84, 18.71) | 0.25 (-3, 3.3) |
| LCNA |  | 10.94 (6.61, 17.68) | 10.02 (5.92, 15.71) | -0.28 (-3.53, 2.79) |  | 11.98 (6.28, 20.25) | 10.64 (5.71, 17.77) | -0.38 (-4.08, 3.36) |  | 9.94 (6.17, 15.68) | 9.39 (5.4, 14.43) | -0.18 (-3.44, 2.74) |
| LCOT |  | 5.54 (3.47, 8.29) | 4.2 (2.5, 6.8) | -0.89 (-3.87, 2.17) |  | 5.94 (3.16, 9.58) | 4.27 (2.41, 7.36) | -1.06 (-4.45, 2.73) |  | 5.16 (3.2, 7.73) | 4.14 (2.38, 6.69) | -0.71 (-3.8, 2.38) |
| **Malaysia** |  |  |  |  |  |  |  |  |  |  |  |  |
| LCHB |  | 43.18 (32.43, 56.08) | 84.4 (60.91, 112.17) | 2.16 (0.27, 4) |  | 68.33 (50.41, 90.68) | 139.6 (100.13, 186.47) | 2.3 (0.32, 4.22) |  | 17.56 (13.09, 23.52) | 25.24 (17.06, 34.66) | 1.17 (-1.04, 3.14) |
| LCHC |  | 9.68 (6.44, 14.06) | 21.14 (13.69, 30.59) | 2.52 (-0.09, 5.03) |  | 8.97 (5.49, 13.49) | 22.89 (13.72, 34.68) | 3.02 (0.05, 5.95) |  | 10.4 (6.87, 15.02) | 19.27 (13.27, 27.42) | 1.99 (-0.4, 4.46) |
| LCAL |  | 8.4 (5.45, 12.35) | 19.05 (11.82, 29.9) | 2.64 (-0.14, 5.49) |  | 13.08 (8.15, 19.6) | 30.8 (18.92, 48.92) | 2.76 (-0.11, 5.78) |  | 3.64 (2.29, 5.69) | 6.46 (3.93, 10.12) | 1.85 (-1.19, 4.79) |
| LCNA |  | 5.42 (3.61, 7.85) | 14.42 (9.49, 20.89) | 3.16 (0.61, 5.66) |  | 5.22 (3.33, 7.86) | 16.26 (10.11, 25.22) | 3.67 (0.81, 6.53) |  | 5.63 (3.69, 8.3) | 12.45 (8.08, 18.12) | 2.56 (-0.09, 5.13) |
| LCOT |  | 2.24 (1.54, 3.21) | 4.08 (2.66, 6.06) | 1.93 (-0.61, 4.42) |  | 2.14 (1.34, 3.29) | 4.49 (2.69, 6.9) | 2.39 (-0.65, 5.29) |  | 2.35 (1.6, 3.33) | 3.64 (2.4, 5.47) | 1.41 (-1.06, 3.97) |
| **Marshall Islands** |  |  |  |  |  |  |  |  |  |  |  |  |
| LCHB |  | 25.22 (15.01, 45.57) | 51.36 (27.87, 95.8) | 2.29 (-1.59, 5.98) |  | 31.13 (17.45, 61.43) | 68.37 (32.94, 147.5) | 2.54 (-2.01, 6.89) |  | 19.01 (11.21, 34.76) | 33.57 (19.91, 54.29) | 1.83 (-1.8, 5.09) |
| LCHC |  | 6.77 (3.57, 12.02) | 14.21 (7.7, 26.23) | 2.39 (-1.44, 6.43) |  | 4.76 (2.3, 9.96) | 11.08 (4.75, 27.08) | 2.73 (-2.39, 7.95) |  | 8.88 (4.62, 16.77) | 17.48 (9.24, 30.85) | 2.18 (-1.92, 6.12) |
| LCAL |  | 3.91 (2.01, 7.49) | 9.42 (4.64, 18.59) | 2.84 (-1.54, 7.18) |  | 5.22 (2.52, 11.04) | 13.11 (5.78, 29.33) | 2.97 (-2.09, 7.92) |  | 2.53 (1.3, 4.93) | 5.56 (3.23, 9.77) | 2.54 (-1.36, 6.51) |
| LCNA |  | 3.21 (1.72, 5.77) | 7.74 (4.4, 12.36) | 2.84 (-0.87, 6.36) |  | 2.21 (1.03, 4.49) | 6 (2.66, 13.23) | 3.22 (-1.69, 8.24) |  | 4.25 (2.25, 8.25) | 9.57 (5.55, 15.5) | 2.62 (-1.28, 6.23) |
| LCOT |  | 2.43 (1.28, 4.33) | 4.58 (2.6, 7.73) | 2.04 (-1.65, 5.8) |  | 1.43 (0.69, 2.88) | 3.06 (1.31, 6.7) | 2.45 (-2.54, 7.33) |  | 3.48 (1.79, 6.91) | 6.17 (3.53, 10.8) | 1.85 (-2.17, 5.8) |
| **Mongolia** |  |  |  |  |  |  |  |  |  |  |  |  |
| LCHB |  | 331.19 (215.46, 499.57) | 457.52 (298.74, 684.51) | 1.04 (-1.66, 3.73) |  | 474.06 (303.95, 709.98) | 664.72 (426.63, 990.79) | 1.09 (-1.64, 3.81) |  | 188.69 (113.24, 294.56) | 255.54 (161.95, 386.79) | 0.98 (-1.93, 3.96) |
| LCHC |  | 315.92 (196.74, 486.29) | 567.61 (392.44, 794.78) | 1.89 (-0.69, 4.5) |  | 341.32 (203.54, 548.24) | 514.06 (336.97, 767.42) | 1.32 (-1.57, 4.28) |  | 290.59 (181.44, 442.94) | 619.81 (443.11, 850.52) | 2.44 (0, 4.98) |
| LCAL |  | 150.69 (91.92, 246.13) | 351.94 (233.32, 551.38) | 2.74 (-0.17, 5.78) |  | 237.49 (139.18, 388.85) | 521.09 (339.45, 804.33) | 2.53 (-0.44, 5.66) |  | 64.11 (37.53, 105.67) | 187.04 (122.64, 298.8) | 3.45 (0.48, 6.69) |
| LCNA |  | 45.8 (28.47, 72.37) | 93.67 (59, 146.19) | 2.31 (-0.66, 5.28) |  | 42.55 (25.07, 70.34) | 76.9 (45.37, 121.32) | 1.91 (-1.41, 5.09) |  | 49.04 (30.33, 76.97) | 110.01 (70.62, 170.05) | 2.61 (-0.28, 5.56) |
| LCOT |  | 33.44 (20.58, 53.86) | 47.51 (29.97, 72.91) | 1.13 (-1.89, 4.08) |  | 27.25 (15.39, 45.61) | 37.11 (22.62, 61.08) | 1 (-2.26, 4.45) |  | 39.61 (23.58, 64.02) | 57.64 (36.37, 84.79) | 1.21 (-1.82, 4.13) |
| **Nauru** |  |  |  |  |  |  |  |  |  |  |  |  |
| LCHB |  | 85.4 (58.1, 116.81) | 78.14 (44.93, 127.44) | -0.29 (-3.08, 2.53) |  | 133.07 (89.31, 187.36) | 122.99 (66.68, 210.29) | -0.25 (-3.33, 2.76) |  | 34.25 (23.06, 49.93) | 32.52 (17.92, 52.96) | -0.17 (-3.31, 2.68) |
| LCHC |  | 15.11 (9.31, 23.21) | 15.98 (8.8, 27.03) | 0.18 (-3.13, 3.44) |  | 16.43 (9.78, 26.9) | 16.21 (7.89, 30.77) | -0.04 (-3.96, 3.7) |  | 13.7 (8.61, 21.89) | 15.75 (8.21, 27.71) | 0.45 (-3.16, 3.77) |
| LCAL |  | 13.61 (8.09, 21.69) | 15.1 (8.22, 26.61) | 0.34 (-3.13, 3.84) |  | 21.86 (12.63, 36.39) | 24.01 (12.3, 44.74) | 0.3 (-3.5, 4.08) |  | 4.75 (2.87, 7.53) | 6.03 (3.16, 10.2) | 0.77 (-2.8, 4.09) |
| LCNA |  | 9.45 (5.92, 14.18) | 10.87 (6.15, 16.99) | 0.45 (-2.69, 3.4) |  | 10.27 (6.04, 16.37) | 11.2 (5.61, 19.96) | 0.28 (-3.45, 3.86) |  | 8.56 (5.35, 12.91) | 10.54 (5.7, 17.24) | 0.67 (-2.64, 3.77) |
| LCOT |  | 5.76 (3.66, 8.64) | 6.06 (3.34, 9.62) | 0.16 (-3.07, 3.12) |  | 5.63 (3.38, 9.02) | 5.69 (2.82, 10.37) | 0.03 (-3.75, 3.62) |  | 5.91 (3.76, 8.93) | 6.44 (3.2, 10.75) | 0.28 (-3.31, 3.39) |
| **New Zealand** |  |  |  |  |  |  |  |  |  |  |  |  |
| LCHB |  | 12.44 (10.23, 14.86) | 24.26 (19.73, 29.89) | 2.15 (0.91, 3.46) |  | 19.87 (15.95, 24.02) | 39.07 (31.39, 48.44) | 2.18 (0.86, 3.58) |  | 5.19 (4.39, 6.05) | 9.48 (7.66, 11.39) | 1.94 (0.76, 3.08) |
| LCHC |  | 14.82 (12.63, 17.28) | 41.48 (35.35, 47.57) | 3.32 (2.31, 4.28) |  | 16.22 (13.17, 19.7) | 45.19 (37.15, 54.33) | 3.31 (2.05, 4.57) |  | 13.46 (11.47, 15.55) | 37.78 (31.83, 43.29) | 3.33 (2.31, 4.28) |
| LCAL |  | 25.84 (22.3, 29.29) | 57.83 (49.43, 66.66) | 2.6 (1.69, 3.53) |  | 43.55 (37.25, 49.95) | 96.6 (80.79, 112.22) | 2.57 (1.55, 3.56) |  | 8.55 (7.27, 10.06) | 19.14 (16.01, 22.91) | 2.6 (1.5, 3.7) |
| LCNA |  | 4.81 (4.09, 5.57) | 15.72 (13.13, 18.47) | 3.82 (2.77, 4.86) |  | 5 (4.05, 5.95) | 16.7 (13.7, 20.13) | 3.89 (2.69, 5.17) |  | 4.62 (3.89, 5.44) | 14.75 (12.15, 17.36) | 3.74 (2.59, 4.83) |
| LCOT |  | 3.36 (2.87, 3.94) | 7.67 (6.58, 8.9) | 2.66 (1.65, 3.65) |  | 3.32 (2.69, 4.07) | 7.81 (6.35, 9.44) | 2.76 (1.43, 4.05) |  | 3.41 (2.95, 3.94) | 7.54 (6.33, 8.81) | 2.56 (1.53, 3.53) |
| **Niue** |  |  |  |  |  |  |  |  |  |  |  |  |
| LCHB |  | 62.67 (39.43, 94.66) | 93.71 (56.48, 152.5) | 1.3 (-1.67, 4.36) |  | 94.26 (58.94, 145.37) | 153.94 (89.69, 265.16) | 1.58 (-1.56, 4.85) |  | 30.76 (18.42, 50.31) | 32.77 (19.2, 52.06) | 0.2 (-3.11, 3.35) |
| LCHC |  | 20.8 (12.46, 33.49) | 31.73 (18.86, 51.15) | 1.36 (-1.85, 4.56) |  | 18.32 (10.17, 32.09) | 34.92 (18.55, 64.86) | 2.08 (-1.77, 5.98) |  | 23.3 (13.69, 38.39) | 28.49 (16.25, 44.35) | 0.65 (-2.77, 3.79) |
| LCAL |  | 13.57 (7.82, 22.07) | 26.79 (15.44, 48.51) | 2.19 (-1.15, 5.89) |  | 20.9 (11.58, 33.87) | 44.57 (24.69, 84.57) | 2.44 (-1.02, 6.41) |  | 6.17 (3.49, 10.72) | 8.8 (4.81, 14.92) | 1.15 (-2.59, 4.69) |
| LCNA |  | 10.27 (6.23, 16.51) | 20.51 (12, 33.01) | 2.23 (-1.03, 5.38) |  | 9.67 (5.37, 16.48) | 23.73 (12.54, 42.86) | 2.9 (-0.88, 6.7) |  | 10.88 (6.43, 17.9) | 17.24 (9.65, 26.8) | 1.48 (-1.99, 4.6) |
| LCOT |  | 4.62 (2.68, 8.02) | 6.48 (3.62, 10.66) | 1.09 (-2.57, 4.45) |  | 4.09 (2.33, 6.98) | 7.11 (3.38, 13.7) | 1.78 (-2.34, 5.71) |  | 5.15 (2.96, 9.04) | 5.85 (3.27, 9.6) | 0.41 (-3.28, 3.8) |
| **Northern Mariana Islands** |  |  |  |  |  |  |  |  |  |  |  |  |
| LCHB |  | 52.33 (34.96, 75.7) | 120.13 (89.96, 158.14) | 2.68 (0.56, 4.87) |  | 73.86 (48.53, 111.9) | 184.13 (137.84, 247.07) | 2.95 (0.67, 5.25) |  | 28.56 (18.58, 41.88) | 49.33 (32.37, 69.91) | 1.76 (-0.83, 4.27) |
| LCHC |  | 9.99 (6.38, 15.64) | 34.38 (21.17, 50.63) | 3.99 (0.98, 6.68) |  | 9.14 (5.28, 15.76) | 36.13 (21.01, 57.62) | 4.43 (0.93, 7.71) |  | 10.92 (6.89, 16.35) | 32.44 (21.08, 47.82) | 3.51 (0.82, 6.25) |
| LCAL |  | 7.52 (4.45, 11.54) | 26.94 (16.64, 43.97) | 4.12 (1.18, 7.39) |  | 11.17 (6.36, 17.35) | 42.36 (25.31, 72) | 4.3 (1.22, 7.83) |  | 3.48 (2.06, 5.6) | 9.88 (5.87, 15.85) | 3.37 (0.15, 6.58) |
| LCNA |  | 8.12 (5.34, 12.25) | 25.41 (16.32, 37.41) | 3.68 (0.93, 6.28) |  | 7.35 (4.35, 12.03) | 27.46 (16.61, 42.71) | 4.25 (1.04, 7.37) |  | 8.98 (5.65, 13.26) | 23.13 (14.56, 34.43) | 3.05 (0.3, 5.83) |
| LCOT |  | 3.76 (2.34, 5.92) | 7.28 (4.46, 11.41) | 2.13 (-0.91, 5.11) |  | 2.97 (1.73, 5.01) | 7.22 (4.3, 11.9) | 2.87 (-0.49, 6.22) |  | 4.63 (2.74, 7.39) | 7.35 (4.23, 11.97) | 1.49 (-1.8, 4.76) |
| **Palau** |  |  |  |  |  |  |  |  |  |  |  |  |
| LCHB |  | 108.93 (68.33, 171.37) | 209.17 (141.28, 297.8) | 2.1 (-0.62, 4.75) |  | 211.19 (132.46, 332.27) | 379.71 (256.47, 540.61) | 1.89 (-0.84, 4.54) |  | 0.13 (0.08, 0.22) | 0.17 (0.11, 0.25) | 0.87 (-2.24, 3.68) |
| LCHC |  | 17.31 (9.58, 30.05) | 36.78 (20.81, 58.91) | 2.43 (-1.19, 5.86) |  | 33.51 (18.54, 58.21) | 66.68 (37.7, 106.84) | 2.22 (-1.4, 5.65) |  | 0.07 (0.04, 0.13) | 0.13 (0.08, 0.19) | 2 (-1.57, 5.03) |
| LCAL |  | 19.7 (11.33, 33.48) | 46.62 (27.61, 73.73) | 2.78 (-0.62, 6.04) |  | 38.2 (21.97, 64.91) | 84.64 (50.11, 133.85) | 2.57 (-0.83, 5.83) |  | 0.02 (0.01, 0.04) | 0.04 (0.02, 0.06) | 2.24 (-2.24, 5.78) |
| LCNA |  | 10.41 (6.1, 17.7) | 27.87 (15.28, 44.82) | 3.18 (-0.47, 6.43) |  | 20.16 (11.81, 34.28) | 50.53 (27.7, 81.31) | 2.96 (-0.69, 6.22) |  | 0.04 (0.02, 0.07) | 0.09 (0.06, 0.13) | 2.62 (-0.5, 6.04) |
| LCOT |  | 4.63 (2.6, 8.3) | 8.8 (5.04, 14.24) | 2.07 (-1.61, 5.49) |  | 8.95 (5.03, 16.08) | 15.96 (9.13, 25.83) | 1.87 (-1.83, 5.28) |  | 0.02 (0.01, 0.05) | 0.03 (0.02, 0.05) | 1.31 (-2.96, 5.19) |
| **Papua New Guinea** |  |  |  |  |  |  |  |  |  |  |  |  |
| LCHB |  | 36.72 (16.98, 90.78) | 26.79 (12.07, 72.09) | -1.02 (-6.51, 4.66) |  | 51.26 (24.02, 138.92) | 37.66 (15.78, 117.14) | -0.99 (-7.02, 5.11) |  | 20.98 (8.39, 46.9) | 15.09 (7.37, 31.75) | -1.06 (-5.97, 4.29) |
| LCHC |  | 9.14 (3.68, 23.05) | 6.93 (2.93, 16.38) | -0.89 (-6.65, 4.82) |  | 8.34 (3.52, 23.69) | 6.26 (2.28, 18.87) | -0.93 (-7.55, 5.42) |  | 10.01 (3.75, 24.96) | 7.65 (3.31, 17.09) | -0.87 (-6.52, 4.89) |
| LCAL |  | 6 (2.62, 16.92) | 4.56 (1.78, 13.25) | -0.89 (-7.26, 5.23) |  | 8.92 (4.04, 27.69) | 6.77 (2.52, 21.75) | -0.89 (-7.73, 5.43) |  | 2.85 (1.04, 7.08) | 2.18 (0.9, 4.96) | -0.86 (-6.65, 5.04) |
| LCNA |  | 3.91 (1.66, 9.81) | 3.29 (1.4, 7.78) | -0.56 (-6.28, 4.98) |  | 3.46 (1.51, 9.88) | 2.89 (1.01, 8.89) | -0.58 (-7.36, 5.72) |  | 4.4 (1.69, 10.41) | 3.72 (1.7, 7.97) | -0.54 (-5.85, 5) |
| LCOT |  | 2.96 (1.17, 7.77) | 2.27 (1, 5.18) | -0.86 (-6.61, 4.8) |  | 2.24 (0.96, 6.51) | 1.7 (0.64, 5.25) | -0.89 (-7.48, 5.48) |  | 3.74 (1.31, 9.17) | 2.89 (1.3, 6.18) | -0.83 (-6.3, 5) |
| **Philippines** |  |  |  |  |  |  |  |  |  |  |  |  |
| LCHB |  | 74.51 (52.24, 93.64) | 69.91 (56.14, 86.68) | -0.21 (-1.65, 1.63) |  | 125.59 (86.17, 154.8) | 112.8 (86.8, 146.73) | -0.35 (-1.87, 1.72) |  | 22.65 (15.73, 35.44) | 25.81 (19.4, 33.19) | 0.42 (-1.94, 2.41) |
| LCHC |  | 14.98 (10.18, 21.77) | 22.11 (16.91, 28.09) | 1.26 (-0.81, 3.27) |  | 18.44 (12.06, 25.46) | 24.54 (17.58, 32.48) | 0.92 (-1.19, 3.2) |  | 11.47 (7.56, 19) | 19.6 (14.79, 24.97) | 1.73 (-0.81, 3.85) |
| LCAL |  | 23 (15.38, 31.26) | 33.89 (25.61, 43.61) | 1.25 (-0.64, 3.36) |  | 38.92 (25.1, 51.79) | 54.92 (39.93, 73.39) | 1.11 (-0.84, 3.46) |  | 6.83 (4.45, 11.48) | 12.26 (8.83, 16.11) | 1.89 (-0.85, 4.15) |
| LCNA |  | 7.45 (5.06, 10.31) | 11 (8.67, 13.86) | 1.26 (-0.56, 3.25) |  | 9.27 (6.05, 12.24) | 12.67 (9.39, 17.03) | 1.01 (-0.86, 3.34) |  | 5.6 (3.76, 9.04) | 9.28 (6.99, 12.25) | 1.63 (-0.83, 3.81) |
| LCOT |  | 4.2 (2.96, 5.61) | 4.46 (3.45, 5.71) | 0.19 (-1.57, 2.12) |  | 5.45 (3.64, 6.96) | 5.26 (3.86, 6.96) | -0.11 (-1.9, 2.09) |  | 2.93 (2.03, 4.64) | 3.64 (2.61, 4.75) | 0.7 (-1.86, 2.74) |
| **Samoa** |  |  |  |  |  |  |  |  |  |  |  |  |
| LCHB |  | 47.37 (34.03, 64.92) | 54.71 (36.56, 77.78) | 0.46 (-1.85, 2.67) |  | 75.62 (52.5, 105.42) | 87.11 (58.06, 125.66) | 0.46 (-1.92, 2.82) |  | 16.21 (10.66, 23.65) | 20.38 (11.98, 33.1) | 0.74 (-2.19, 3.65) |
| LCHC |  | 12.26 (7.65, 18.73) | 15.68 (9.4, 24.3) | 0.79 (-2.22, 3.73) |  | 14.22 (8.36, 23.16) | 17.13 (9.6, 28.47) | 0.6 (-2.84, 3.95) |  | 10.11 (6.34, 14.97) | 14.13 (8.29, 21.81) | 1.08 (-1.91, 3.99) |
| LCAL |  | 9.64 (5.75, 15.1) | 11.61 (6.7, 18.61) | 0.6 (-2.62, 3.79) |  | 15.82 (9.32, 25.08) | 18.87 (10.66, 30.81) | 0.57 (-2.76, 3.86) |  | 2.82 (1.72, 4.53) | 3.92 (2.23, 6.43) | 1.06 (-2.29, 4.25) |
| LCNA |  | 6.96 (4.24, 10.95) | 9.39 (5.77, 13.91) | 0.97 (-2.07, 3.83) |  | 8.19 (4.65, 13.55) | 10.5 (6.28, 17.27) | 0.8 (-2.48, 4.23) |  | 5.59 (3.4, 8.67) | 8.21 (4.58, 12.17) | 1.24 (-2.06, 4.11) |
| LCOT |  | 3.19 (2, 4.94) | 3.9 (2.28, 5.96) | 0.65 (-2.49, 3.52) |  | 3.46 (2.06, 5.65) | 4.03 (2.31, 6.59) | 0.49 (-2.89, 3.75) |  | 2.9 (1.76, 4.48) | 3.76 (2.13, 5.84) | 0.84 (-2.4, 3.87) |
| **Singapore** |  |  |  |  |  |  |  |  |  |  |  |  |
| LCHB |  | 126.18 (108.9, 143.81) | 120.52 (97.84, 142.78) | -0.15 (-1.24, 0.87) |  | 206.4 (178.95, 235.39) | 193.34 (157.06, 228.68) | -0.21 (-1.31, 0.79) |  | 44.83 (35.62, 54.62) | 45.08 (33.76, 57.87) | 0.02 (-1.55, 1.57) |
| LCHC |  | 29.41 (20.13, 40.5) | 42.48 (28.51, 60.4) | 1.19 (-1.13, 3.54) |  | 34.01 (21.99, 49) | 49.76 (31.67, 73.61) | 1.23 (-1.41, 3.9) |  | 24.74 (17.25, 33.09) | 34.94 (24.09, 45.98) | 1.11 (-1.02, 3.16) |
| LCAL |  | 10.39 (6.77, 15.4) | 14.03 (8.86, 21.22) | 0.97 (-1.78, 3.69) |  | 17.74 (11.56, 26.73) | 23.57 (14.59, 35.97) | 0.92 (-1.95, 3.66) |  | 2.94 (1.88, 4.41) | 4.15 (2.59, 6.22) | 1.11 (-1.72, 3.86) |
| LCNA |  | 6.33 (4.24, 9.42) | 9.67 (6.39, 14.79) | 1.37 (-1.25, 4.03) |  | 7.53 (4.92, 11.61) | 11.09 (6.82, 17.57) | 1.25 (-1.72, 4.11) |  | 5.12 (3.34, 7.38) | 8.19 (5.42, 12.02) | 1.52 (-1, 4.13) |
| LCOT |  | 4.5 (3.19, 6.41) | 4.84 (3.18, 6.97) | 0.23 (-2.26, 2.52) |  | 5.46 (3.76, 8.11) | 5.62 (3.56, 8.42) | 0.09 (-2.66, 2.6) |  | 3.52 (2.47, 5.05) | 4.02 (2.6, 5.73) | 0.43 (-2.14, 2.71) |
| **Solomon Islands** |  |  |  |  |  |  |  |  |  |  |  |  |
| LCHB |  | 63.79 (18.81, 137.99) | 60.17 (36.34, 97.97) | -0.19 (-4.3, 5.32) |  | 103.74 (30.02, 228.11) | 95.26 (57.04, 164.27) | -0.28 (-4.47, 5.48) |  | 20.74 (6.64, 44.75) | 23.62 (14.12, 40.96) | 0.42 (-3.72, 5.87) |
| LCHC |  | 11.89 (3.99, 28.04) | 12.47 (6.84, 22.73) | 0.15 (-4.55, 5.61) |  | 14.55 (4.24, 35.43) | 13.35 (6.91, 25.78) | -0.28 (-5.27, 5.82) |  | 9.02 (3.22, 21.42) | 11.56 (6.25, 21.3) | 0.8 (-3.97, 6.09) |
| LCAL |  | 9.38 (2.92, 21.98) | 9.57 (5.29, 16.34) | 0.06 (-4.59, 5.55) |  | 15.69 (4.45, 36.89) | 15.43 (8.32, 27.17) | -0.05 (-4.8, 5.84) |  | 2.59 (0.87, 6.49) | 3.46 (1.9, 6.22) | 0.93 (-3.96, 6.35) |
| LCNA |  | 5.83 (1.96, 13.52) | 7.04 (3.87, 11.82) | 0.61 (-4.04, 5.8) |  | 7.07 (2.14, 18.04) | 7.64 (4.06, 14.18) | 0.25 (-4.81, 6.1) |  | 4.49 (1.5, 10.22) | 6.42 (3.73, 10.96) | 1.15 (-3.25, 6.42) |
| LCOT |  | 4.06 (1.36, 9.58) | 4.44 (2.65, 7.49) | 0.29 (-4.15, 5.5) |  | 4.46 (1.4, 10.51) | 4.38 (2.35, 7.76) | -0.06 (-4.83, 5.52) |  | 3.62 (1.12, 8.24) | 4.5 (2.6, 7.67) | 0.7 (-3.72, 6.21) |
| **South Korea** |  |  |  |  |  |  |  |  |  |  |  |  |
| LCHB |  | 528.42 (376.7, 693.67) | 376.05 (295.13, 481.24) | -1.1 (-2.76, 0.79) |  | 908.79 (637.45, 1219.36) | 662.09 (517.61, 849.26) | -1.02 (-2.76, 0.93) |  | 144.69 (96.51, 197.15) | 87.84 (61.89, 123.69) | -1.61 (-3.74, 0.8) |
| LCHC |  | 88.12 (56.78, 131.03) | 93.97 (62.45, 136.38) | 0.21 (-2.39, 2.83) |  | 70.82 (41.9, 112.65) | 86.6 (53.32, 133.68) | 0.65 (-2.41, 3.74) |  | 105.56 (65.85, 153.29) | 101.39 (68.53, 141.18) | -0.13 (-2.6, 2.46) |
| LCAL |  | 88.58 (54.81, 132.27) | 105.18 (68.55, 157.44) | 0.55 (-2.12, 3.4) |  | 140.32 (84.26, 215.06) | 174.84 (110.43, 260.97) | 0.71 (-2.15, 3.65) |  | 36.39 (21.46, 56.66) | 35 (22.08, 51.29) | -0.13 (-3.04, 2.81) |
| LCNA |  | 34.89 (23.29, 51.24) | 38.79 (25.19, 58.77) | 0.34 (-2.29, 2.99) |  | 33.78 (20.65, 52.27) | 41.35 (24.78, 66.93) | 0.65 (-2.41, 3.79) |  | 36.02 (22.01, 52.99) | 36.21 (23.05, 55.26) | 0.02 (-2.69, 2.97) |
| LCOT |  | 23.33 (14.85, 35.25) | 18.38 (11.66, 26.98) | -0.77 (-3.57, 1.93) |  | 24.25 (14.45, 38.6) | 21.14 (13.44, 32.62) | -0.44 (-3.4, 2.63) |  | 22.4 (13.85, 34.68) | 15.6 (9.6, 22.18) | -1.17 (-4.14, 1.52) |
| **Tokelau** |  |  |  |  |  |  |  |  |  |  |  |  |
| LCHB |  | 48.04 (27.73, 86.16) | 79.7 (48.12, 128.03) | 1.63 (-1.88, 4.93) |  | 57.33 (31.18, 112.41) | 116.2 (65.84, 195.75) | 2.28 (-1.73, 5.93) |  | 38.71 (20.94, 71.54) | 41.71 (25.16, 67.73) | 0.24 (-3.37, 3.79) |
| LCHC |  | 18.95 (10.27, 36.51) | 25.31 (14.31, 43.07) | 0.93 (-3.02, 4.62) |  | 12.43 (6.24, 25.97) | 23.44 (12.48, 40.71) | 2.05 (-2.36, 6.05) |  | 25.51 (13.34, 50.67) | 27.26 (15.93, 47.28) | 0.21 (-3.73, 4.08) |
| LCAL |  | 9.49 (4.77, 18.94) | 17.66 (9.5, 31.15) | 2 (-2.23, 6.05) |  | 12.27 (6.01, 26.49) | 26.78 (14.36, 47.35) | 2.52 (-1.98, 6.66) |  | 6.69 (3.11, 14.39) | 8.17 (4.17, 14.78) | 0.64 (-4, 5.03) |
| LCNA |  | 8.25 (4.4, 15.36) | 14.31 (7.81, 24.18) | 1.78 (-2.18, 5.5) |  | 5.53 (2.68, 11.64) | 13.65 (7.17, 24.25) | 2.91 (-1.56, 7.11) |  | 10.98 (5.52, 20.76) | 14.99 (7.91, 27.06) | 1 (-3.11, 5.13) |
| LCOT |  | 4.48 (2.37, 8.74) | 5.66 (3.26, 9.85) | 0.75 (-3.18, 4.6) |  | 2.55 (1.25, 5.34) | 4.83 (2.5, 8.68) | 2.06 (-2.45, 6.25) |  | 6.42 (3.37, 12.52) | 6.52 (3.75, 11.79) | 0.05 (-3.89, 4.04) |
| **Tonga** |  |  |  |  |  |  |  |  |  |  |  |  |
| LCHB |  | 231.33 (158.3, 334.63) | 276.24 (181.52, 409.36) | 0.57 (-1.97, 3.06) |  | 373.2 (260.49, 540.18) | 475.79 (307.94, 724.41) | 0.78 (-1.81, 3.3) |  | 87.03 (51.94, 140.06) | 77.86 (50.16, 115.33) | -0.36 (-3.31, 2.57) |
| LCHC |  | 58.29 (33.14, 98.83) | 75.33 (45.63, 117.45) | 0.83 (-2.49, 4.08) |  | 67.78 (38.42, 115.91) | 95.05 (52.8, 159.65) | 1.09 (-2.54, 4.59) |  | 48.64 (25.51, 86.24) | 55.74 (33.06, 82.19) | 0.44 (-3.09, 3.77) |
| LCAL |  | 39.54 (22.49, 65.4) | 53.09 (30.62, 87.03) | 0.95 (-2.45, 4.37) |  | 65.79 (37.77, 109.49) | 92.63 (53.23, 152.01) | 1.1 (-2.33, 4.49) |  | 12.83 (6.38, 22.72) | 13.79 (7.56, 22.15) | 0.23 (-3.55, 4.02) |
| LCNA |  | 31.72 (18.83, 52.67) | 48.53 (29.39, 72.81) | 1.37 (-1.88, 4.36) |  | 37.17 (21.9, 63.07) | 62.88 (35.25, 104.34) | 1.7 (-1.88, 5.04) |  | 26.17 (14.28, 46.03) | 34.27 (20.8, 52.58) | 0.87 (-2.56, 4.2) |
| LCOT |  | 15.5 (9.44, 25.19) | 18.18 (10.86, 28.85) | 0.51 (-2.71, 3.6) |  | 16.47 (9.68, 27.59) | 21.88 (12.38, 38.85) | 0.92 (-2.59, 4.48) |  | 14.51 (8.11, 25.28) | 14.51 (8.59, 23.22) | 0 (-3.48, 3.39) |
| **Tuvalu** |  |  |  |  |  |  |  |  |  |  |  |  |
| LCHB |  | 60.12 (39.47, 91.73) | 69.35 (43.4, 106.28) | 0.46 (-2.41, 3.2) |  | 82.82 (53.59, 136.78) | 101.23 (60.65, 165.33) | 0.65 (-2.62, 3.63) |  | 38.88 (22.61, 65.77) | 34.69 (21.82, 53.66) | -0.37 (-3.56, 2.79) |
| LCHC |  | 18.39 (10.79, 32.17) | 21.29 (12.85, 34.7) | 0.47 (-2.96, 3.77) |  | 14.72 (8, 26.93) | 18.85 (9.83, 33.98) | 0.8 (-3.25, 4.67) |  | 21.82 (12.08, 39.49) | 23.95 (14.71, 40.95) | 0.3 (-3.19, 3.94) |
| LCAL |  | 10.55 (6.06, 18.13) | 14.09 (7.92, 23.91) | 0.93 (-2.67, 4.43) |  | 15.47 (8.59, 26.83) | 20.95 (11.26, 37.56) | 0.98 (-2.8, 4.76) |  | 5.95 (3.03, 11.11) | 6.63 (3.78, 11.29) | 0.35 (-3.48, 4.24) |
| LCNA |  | 8.27 (4.86, 14.47) | 11.83 (7.14, 18.83) | 1.15 (-2.28, 4.37) |  | 6.71 (3.76, 11.97) | 10.93 (5.9, 20.21) | 1.57 (-2.28, 5.43) |  | 9.73 (5.42, 18.55) | 12.8 (7.99, 20.49) | 0.88 (-2.72, 4.29) |
| LCOT |  | 4.96 (2.96, 8.83) | 5.29 (3.24, 8.78) | 0.21 (-3.23, 3.51) |  | 3.5 (1.91, 6.39) | 4.52 (2.4, 8.42) | 0.82 (-3.16, 4.79) |  | 6.32 (3.61, 11.34) | 6.13 (3.76, 10.57) | -0.1 (-3.56, 3.47) |
| **Vanuatu** |  |  |  |  |  |  |  |  |  |  |  |  |
| LCHB |  | 42.81 (25.17, 79.68) | 47.81 (29.37, 78.73) | 0.36 (-3.22, 3.68) |  | 64.71 (39.34, 124.57) | 72.47 (42.67, 124.99) | 0.37 (-3.46, 3.73) |  | 19.61 (9.3, 41.49) | 22.66 (13.72, 36.83) | 0.47 (-3.57, 4.44) |
| LCHC |  | 9.61 (5.13, 19.17) | 12.62 (7.21, 21.45) | 0.88 (-3.15, 4.61) |  | 9.95 (5.27, 19.89) | 12.48 (6.16, 23.94) | 0.73 (-3.78, 4.88) |  | 9.24 (4.41, 20.04) | 12.77 (7.77, 20.76) | 1.04 (-3.06, 5) |
| LCAL |  | 6.36 (3.45, 12.82) | 8.23 (4.3, 13.74) | 0.83 (-3.52, 4.46) |  | 10.03 (5.54, 19.92) | 12.92 (6.48, 22.66) | 0.82 (-3.62, 4.54) |  | 2.46 (1.08, 5.57) | 3.45 (2, 5.91) | 1.09 (-3.3, 5.48) |
| LCNA |  | 4.66 (2.43, 9.37) | 6.8 (4.05, 11.1) | 1.22 (-2.71, 4.9) |  | 4.74 (2.54, 9.66) | 6.78 (3.39, 12.99) | 1.15 (-3.38, 5.26) |  | 4.57 (2.1, 9.76) | 6.83 (4.03, 11.18) | 1.3 (-2.85, 5.39) |
| LCOT |  | 3.12 (1.68, 6.32) | 3.74 (2.13, 6.47) | 0.58 (-3.51, 4.35) |  | 2.82 (1.6, 5.52) | 3.3 (1.59, 6.45) | 0.51 (-4.01, 4.5) |  | 3.43 (1.63, 7.3) | 4.2 (2.4, 6.93) | 0.65 (-3.59, 4.67) |
| **Viet Nam** |  |  |  |  |  |  |  |  |  |  |  |  |
| LCHB |  | 145.59 (96.73, 198.94) | 182.98 (119.49, 282) | 0.74 (-1.64, 3.45) |  | 251.77 (167.56, 350.98) | 310.48 (193.62, 494.93) | 0.68 (-1.92, 3.49) |  | 45.36 (30.2, 62.65) | 56.09 (34.28, 83.64) | 0.68 (-1.95, 3.29) |
| LCHC |  | 36.89 (22.32, 59.78) | 52.63 (30.31, 84.67) | 1.15 (-2.19, 4.3) |  | 52.66 (29.68, 87.31) | 72.76 (37.85, 128.18) | 1.04 (-2.7, 4.72) |  | 22.01 (13.94, 33.83) | 32.58 (18.53, 49.76) | 1.27 (-1.94, 4.1) |
| LCAL |  | 42.94 (26.63, 67.19) | 104.13 (61.68, 168.47) | 2.86 (-0.28, 5.95) |  | 76.95 (46.56, 123.71) | 181.56 (105.4, 296.12) | 2.77 (-0.52, 5.97) |  | 10.84 (6.9, 16.82) | 27.08 (15.85, 44.82) | 2.95 (-0.19, 6.04) |
| LCNA |  | 16.49 (10.04, 25.78) | 27.47 (17.03, 42.28) | 1.65 (-1.34, 4.64) |  | 20.75 (12.07, 34.64) | 34.1 (19.64, 55.91) | 1.6 (-1.83, 4.95) |  | 12.46 (7.81, 19.53) | 20.88 (12.73, 31.68) | 1.67 (-1.38, 4.52) |
| LCOT |  | 7.73 (4.79, 11.35) | 10.14 (5.88, 16.12) | 0.88 (-2.12, 3.91) |  | 9.94 (6.01, 14.88) | 12.79 (7.14, 21.78) | 0.81 (-2.37, 4.15) |  | 5.65 (3.49, 8.6) | 7.51 (4.5, 11.77) | 0.92 (-2.09, 3.92) |
| **Western Pacific Region** |  |  |  |  |  |  |  |  |  |  |  |  |
| LCHB |  | 177.79 (151.15, 206.44) | 194.37 (154.61, 245.83) | 0.29 (-0.93, 1.57) |  | 295.5 (244.06, 350.65) | 329.97 (259.06, 431.87) | 0.36 (-0.98, 1.84) |  | 54.85 (44.17, 65.42) | 54.07 (41.44, 69.43) | -0.05 (-1.47, 1.46) |
| LCHC |  | 52.39 (45.78, 60.05) | 66.48 (55.14, 78.94) | 0.77 (-0.28, 1.76) |  | 60.27 (52.48, 69.69) | 70.45 (57.39, 86.75) | 0.5 (-0.63, 1.62) |  | 44.15 (36.39, 52.56) | 62.38 (50.6, 75.73) | 1.12 (-0.12, 2.36) |
| LCAL |  | 25.69 (20.43, 32.2) | 40.71 (30.82, 53.63) | 1.49 (-0.14, 3.11) |  | 37.44 (29.06, 47.55) | 59.78 (44.17, 80.24) | 1.51 (-0.24, 3.28) |  | 13.42 (10.41, 16.91) | 20.97 (15.78, 26.78) | 1.44 (-0.22, 3.05) |
| LCNA |  | 11.8 (9.75, 14.13) | 18.53 (14.39, 22.85) | 1.46 (0.06, 2.75) |  | 12.7 (10.05, 15.67) | 20.42 (14.96, 27.22) | 1.53 (-0.15, 3.21) |  | 10.86 (8.69, 13.26) | 16.57 (13.08, 21.06) | 1.36 (-0.04, 2.86) |
| LCOT |  | 14.11 (11.5, 17.27) | 14.65 (11.4, 18.82) | 0.12 (-1.34, 1.59) |  | 14.66 (11.7, 18.24) | 16.3 (12.03, 22.62) | 0.34 (-1.34, 2.13) |  | 13.54 (10.52, 17.08) | 12.95 (9.75, 16.78) | -0.14 (-1.81, 1.51) |
| **Whole World** |  |  |  |  |  |  |  |  |  |  |  |  |
| LCHB |  | 70.27 (61.04, 81.79) | 71.83 (59.65, 87.25) | 0.07 (-1.02, 1.15) |  | 114.38 (97.78, 132.73) | 117.92 (95.84, 146.57) | 0.1 (-1.05, 1.31) |  | 25.54 (20.81, 30.88) | 25.41 (20.72, 30.93) | -0.02 (-1.29, 1.28) |
| LCHC |  | 29.48 (25.26, 35.07) | 39.27 (33.74, 45.73) | 0.92 (-0.12, 1.91) |  | 31.87 (27.16, 37.81) | 41.77 (34.61, 50) | 0.87 (-0.29, 1.97) |  | 27.05 (22.78, 32.77) | 36.75 (31.77, 42.25) | 0.99 (-0.1, 1.99) |
| LCAL |  | 19.54 (15.99, 24.01) | 29.35 (23.91, 36.06) | 1.31 (-0.01, 2.62) |  | 29.98 (24.65, 36.9) | 45.87 (37.32, 56.74) | 1.37 (0.04, 2.69) |  | 8.95 (7.1, 11.16) | 12.71 (10.14, 15.61) | 1.13 (-0.31, 2.54) |
| LCNA |  | 7.57 (6.02, 9.37) | 12.61 (10.25, 15.23) | 1.65 (0.29, 2.99) |  | 7.64 (6.04, 9.42) | 13.15 (10.24, 16.48) | 1.75 (0.27, 3.24) |  | 7.51 (6.03, 9.47) | 12.08 (9.85, 14.64) | 1.53 (0.13, 2.86) |
| LCOT |  | 6.76 (5.61, 8.33) | 7.55 (6.14, 9.28) | 0.36 (-0.98, 1.62) |  | 6.69 (5.51, 8.36) | 7.8 (6.09, 9.95) | 0.5 (-1.02, 1.91) |  | 6.84 (5.51, 8.61) | 7.29 (5.9, 8.94) | 0.21 (-1.22, 1.56) |

Abbreviation: DALYs, Disability-adjusted life years; UI, Uncertainty interval; EAPC, Estimated annual percentage change; CI, Confidence interval; LCHB, Liver cancer by HBC; LCHC, Liver cancer by HCV; LCAL, Liver cancer by alcoholic use; LCNA; Liver cancer by nonalcoholic steatohepatitis (NASH); LCOT, Liver cancer by other causes

Table S10. Age-standardized incidence rates (per 100,000) for specific liver cancer in the Western Pacific region by member state and sex, in 1990 to 2021.

| **Region/Liver cancer cause** |  | **Both sexes combined** | | |  | **Male** | | |  | **Female** | | |
| --- | --- | --- | --- | --- | --- | --- | --- | --- | --- | --- | --- | --- |
|  |  | **1990 (95% UI)** | **2021 (95% UI)** | **EAPC, %**  **(95% CI)** |  | **1990 (95% UI)** | **2021 (95% UI)** | **EAPC, %**  **(95% CI)** |  | **1990 (95% UI)** | **2021 (95% UI)** | **EAPC, %**  **(95% CI)** |
| **American Samoa** |  |  |  |  |  |  |  |  |  |  |  |  |
| LCHB |  | 1.13 (0.8, 1.57) | 3.26 (2.29, 4.48) | 3.42 (1.22, 5.56) |  | 1.59 (1.11, 2.29) | 4.55 (3.16, 6.31) | 3.39 (1.04, 5.61) |  | 0.64 (0.42, 1.01) | 1.9 (1.27, 2.85) | 3.51 (0.74, 6.18) |
| LCHC |  | 0.36 (0.23, 0.56) | 1.2 (0.78, 1.78) | 3.88 (1.07, 6.6) |  | 0.3 (0.18, 0.51) | 0.97 (0.6, 1.5) | 3.79 (0.52, 6.84) |  | 0.42 (0.27, 0.64) | 1.44 (0.94, 2.13) | 3.97 (1.24, 6.66) |
| LCAL |  | 0.2 (0.12, 0.32) | 0.62 (0.39, 0.97) | 3.65 (0.64, 6.74) |  | 0.29 (0.17, 0.49) | 0.91 (0.56, 1.42) | 3.69 (0.43, 6.85) |  | 0.1 (0.06, 0.16) | 0.33 (0.2, 0.54) | 3.85 (0.72, 7.09) |
| LCNA |  | 0.24 (0.15, 0.35) | 0.87 (0.57, 1.28) | 4.15 (1.57, 6.92) |  | 0.21 (0.13, 0.32) | 0.74 (0.45, 1.14) | 4.06 (1.1, 7) |  | 0.27 (0.17, 0.41) | 1 (0.63, 1.52) | 4.22 (1.39, 7.07) |
| LCOT |  | 0.09 (0.06, 0.14) | 0.24 (0.15, 0.37) | 3.16 (0.22, 5.87) |  | 0.07 (0.04, 0.12) | 0.19 (0.12, 0.3) | 3.22 (0, 6.5) |  | 0.11 (0.06, 0.17) | 0.3 (0.18, 0.47) | 3.24 (0.18, 6.64) |
| **Australia** |  |  |  |  |  |  |  |  |  |  |  |  |
| LCHB |  | 0.35 (0.25, 0.49) | 1.23 (0.83, 1.77) | 4.05 (1.7, 6.31) |  | 0.57 (0.4, 0.81) | 2.03 (1.36, 2.93) | 4.1 (1.67, 6.42) |  | 0.13 (0.09, 0.18) | 0.45 (0.3, 0.65) | 4.01 (1.65, 6.38) |
| LCHC |  | 0.59 (0.43, 0.79) | 3.08 (2.32, 4.08) | 5.33 (3.48, 7.26) |  | 0.69 (0.48, 0.98) | 3.65 (2.56, 5) | 5.37 (3.1, 7.56) |  | 0.49 (0.37, 0.62) | 2.52 (1.89, 3.23) | 5.28 (3.6, 6.99) |
| LCAL |  | 0.91 (0.69, 1.11) | 3.96 (3.08, 4.99) | 4.74 (3.29, 6.38) |  | 1.57 (1.21, 1.92) | 6.88 (5.36, 8.68) | 4.77 (3.31, 6.36) |  | 0.25 (0.18, 0.34) | 1.1 (0.77, 1.56) | 4.78 (2.64, 6.97) |
| LCNA |  | 0.17 (0.12, 0.23) | 1.06 (0.72, 1.48) | 5.9 (3.68, 8.1) |  | 0.19 (0.12, 0.27) | 1.22 (0.79, 1.73) | 6 (3.46, 8.61) |  | 0.15 (0.1, 0.2) | 0.9 (0.6, 1.27) | 5.78 (3.54, 8.2) |
| LCOT |  | 0.09 (0.06, 0.12) | 0.4 (0.28, 0.55) | 4.81 (2.73, 7.15) |  | 0.09 (0.06, 0.13) | 0.44 (0.29, 0.61) | 5.12 (2.59, 7.48) |  | 0.08 (0.06, 0.12) | 0.37 (0.24, 0.52) | 4.94 (2.24, 6.97) |
| **Brunei Darussalam** |  |  |  |  |  |  |  |  |  |  |  |  |
| LCHB |  | 3.13 (2.21, 4.44) | 3.81 (2.7, 5.18) | 0.63 (-1.6, 2.75) |  | 4.71 (3.35, 6.57) | 5.7 (4.11, 7.71) | 0.62 (-1.51, 2.69) |  | 1.34 (0.88, 1.97) | 1.69 (1.07, 2.61) | 0.75 (-1.97, 3.51) |
| LCHC |  | 1.64 (0.97, 2.47) | 2.46 (1.61, 3.49) | 1.31 (-1.38, 4.13) |  | 1.65 (0.92, 2.53) | 2.37 (1.48, 3.58) | 1.17 (-1.73, 4.38) |  | 1.63 (0.98, 2.46) | 2.57 (1.63, 3.86) | 1.47 (-1.33, 4.42) |
| LCAL |  | 0.47 (0.28, 0.75) | 0.63 (0.4, 0.97) | 0.95 (-2.03, 4.01) |  | 0.72 (0.43, 1.16) | 0.94 (0.59, 1.46) | 0.86 (-2.18, 3.94) |  | 0.19 (0.11, 0.31) | 0.28 (0.16, 0.46) | 1.25 (-2.13, 4.62) |
| LCNA |  | 0.3 (0.18, 0.48) | 0.48 (0.3, 0.74) | 1.52 (-1.52, 4.56) |  | 0.29 (0.17, 0.49) | 0.43 (0.26, 0.68) | 1.27 (-2.04, 4.47) |  | 0.31 (0.17, 0.53) | 0.53 (0.32, 0.86) | 1.73 (-1.63, 5.23) |
| LCOT |  | 0.21 (0.13, 0.32) | 0.29 (0.18, 0.46) | 1.04 (-1.86, 4.08) |  | 0.2 (0.12, 0.32) | 0.28 (0.16, 0.45) | 1.09 (-2.24, 4.26) |  | 0.21 (0.13, 0.33) | 0.31 (0.18, 0.52) | 1.26 (-1.96, 4.47) |
| **Cambodia** |  |  |  |  |  |  |  |  |  |  |  |  |
| LCHB |  | 1.82 (0.93, 3.49) | 1.8 (0.87, 3.56) | -0.04 (-4.48, 4.33) |  | 2.22 (1.09, 5.17) | 2.2 (0.88, 5.43) | -0.03 (-5.71, 5.18) |  | 1.46 (0.61, 2.99) | 1.41 (0.64, 2.44) | -0.11 (-4.97, 4.47) |
| LCHC |  | 0.98 (0.47, 1.91) | 1.23 (0.65, 2.15) | 0.73 (-3.48, 4.9) |  | 0.74 (0.34, 1.82) | 0.84 (0.34, 2.23) | 0.41 (-5.41, 6.07) |  | 1.2 (0.47, 2.5) | 1.61 (0.77, 2.78) | 0.95 (-3.8, 5.73) |
| LCAL |  | 0.55 (0.24, 1.18) | 1.17 (0.54, 2.37) | 2.43 (-2.52, 7.39) |  | 0.79 (0.33, 1.82) | 1.61 (0.64, 3.91) | 2.3 (-3.37, 7.97) |  | 0.34 (0.13, 0.79) | 0.75 (0.32, 1.43) | 2.55 (-2.92, 7.74) |
| LCNA |  | 0.3 (0.14, 0.59) | 0.42 (0.21, 0.77) | 1.09 (-3.33, 5.5) |  | 0.21 (0.09, 0.51) | 0.29 (0.12, 0.7) | 1.04 (-4.67, 6.62) |  | 0.38 (0.14, 0.83) | 0.55 (0.25, 0.98) | 1.19 (-3.87, 6.28) |
| LCOT |  | 0.13 (0.06, 0.25) | 0.15 (0.07, 0.28) | 0.46 (-4.11, 4.97) |  | 0.09 (0.04, 0.22) | 0.1 (0.04, 0.25) | 0.34 (-5.5, 5.91) |  | 0.17 (0.06, 0.35) | 0.19 (0.08, 0.35) | 0.36 (-4.76, 5.69) |
| **China** |  |  |  |  |  |  |  |  |  |  |  |  |
| LCHB |  | 5.37 (4.42, 6.39) | 8.34 (6.49, 10.79) | 1.42 (0.05, 2.88) |  | 8.77 (7.03, 10.72) | 13.99 (10.48, 18.93) | 1.51 (-0.07, 3.2) |  | 1.74 (1.36, 2.16) | 2.42 (1.78, 3.2) | 1.06 (-0.62, 2.76) |
| LCHC |  | 1.23 (0.99, 1.49) | 2.56 (2, 3.15) | 2.36 (0.95, 3.73) |  | 1.01 (0.77, 1.3) | 2.27 (1.66, 3.07) | 2.61 (0.79, 4.46) |  | 1.45 (1.13, 1.78) | 2.87 (2.19, 3.67) | 2.2 (0.67, 3.8) |
| LCAL |  | 0.64 (0.49, 0.81) | 1.44 (1.07, 1.92) | 2.62 (0.9, 4.41) |  | 0.81 (0.61, 1.08) | 1.91 (1.31, 2.7) | 2.77 (0.62, 4.8) |  | 0.45 (0.34, 0.59) | 0.95 (0.69, 1.25) | 2.41 (0.51, 4.2) |
| LCNA |  | 0.34 (0.28, 0.42) | 0.79 (0.61, 1.01) | 2.72 (1.2, 4.14) |  | 0.35 (0.27, 0.45) | 0.85 (0.6, 1.16) | 2.86 (0.93, 4.7) |  | 0.34 (0.26, 0.42) | 0.74 (0.56, 0.98) | 2.51 (0.93, 4.28) |
| LCOT |  | 0.43 (0.34, 0.54) | 0.65 (0.49, 0.83) | 1.33 (-0.31, 2.88) |  | 0.43 (0.33, 0.55) | 0.7 (0.5, 1.01) | 1.57 (-0.31, 3.61) |  | 0.43 (0.33, 0.56) | 0.6 (0.44, 0.79) | 1.07 (-0.78, 2.82) |
| **Cook Islands** |  |  |  |  |  |  |  |  |  |  |  |  |
| LCHB |  | 3.61 (2.6, 5) | 6.37 (4.39, 9.05) | 1.83 (-0.42, 4.02) |  | 5.15 (3.75, 7.3) | 10.59 (7.22, 15.4) | 2.33 (-0.04, 4.56) |  | 1.93 (1.27, 2.78) | 2.39 (1.51, 3.62) | 0.69 (-1.97, 3.38) |
| LCHC |  | 1.34 (0.86, 1.96) | 2.88 (1.89, 4.15) | 2.47 (-0.12, 5.08) |  | 1.17 (0.71, 1.84) | 3.08 (1.99, 4.6) | 3.12 (0.25, 6.03) |  | 1.52 (0.99, 2.23) | 2.69 (1.73, 3.98) | 1.84 (-0.82, 4.49) |
| LCAL |  | 0.74 (0.45, 1.16) | 3.68 (2.39, 5.62) | 5.17 (2.33, 8.14) |  | 1.1 (0.67, 1.73) | 6.22 (3.96, 9.49) | 5.59 (2.67, 8.55) |  | 0.35 (0.22, 0.57) | 1.29 (0.78, 1.98) | 4.21 (1.01, 7.09) |
| LCNA |  | 0.7 (0.46, 1.04) | 1.94 (1.25, 2.92) | 3.29 (0.59, 5.96) |  | 0.64 (0.4, 1.01) | 2.21 (1.34, 3.37) | 4 (0.91, 6.87) |  | 0.76 (0.48, 1.15) | 1.69 (1.03, 2.53) | 2.58 (-0.36, 5.36) |
| LCOT |  | 0.27 (0.18, 0.41) | 0.46 (0.28, 0.7) | 1.72 (-1.23, 4.38) |  | 0.23 (0.13, 0.37) | 0.49 (0.29, 0.78) | 2.44 (-0.79, 5.78) |  | 0.32 (0.21, 0.5) | 0.42 (0.25, 0.67) | 0.88 (-2.24, 3.74) |
| **Federated States of Micronesia** |  |  |  |  |  |  |  |  |  |  |  |  |
| LCHB |  | 1.36 (0.87, 2.15) | 2.11 (1.2, 3.63) | 1.42 (-1.88, 4.61) |  | 1.92 (1.1, 3.34) | 3.1 (1.65, 5.92) | 1.55 (-2.27, 5.43) |  | 0.77 (0.48, 1.14) | 1.08 (0.51, 1.79) | 1.09 (-2.59, 4.25) |
| LCHC |  | 0.46 (0.29, 0.73) | 0.74 (0.42, 1.26) | 1.53 (-1.78, 4.74) |  | 0.38 (0.2, 0.75) | 0.64 (0.32, 1.4) | 1.68 (-2.75, 6.28) |  | 0.53 (0.32, 0.83) | 0.84 (0.38, 1.34) | 1.49 (-2.52, 4.62) |
| LCAL |  | 0.29 (0.16, 0.49) | 0.46 (0.25, 0.88) | 1.49 (-2.17, 5.5) |  | 0.42 (0.21, 0.77) | 0.68 (0.33, 1.39) | 1.55 (-2.73, 6.1) |  | 0.15 (0.08, 0.24) | 0.22 (0.11, 0.39) | 1.24 (-2.52, 5.11) |
| LCNA |  | 0.21 (0.13, 0.34) | 0.41 (0.24, 0.66) | 2.16 (-1.12, 5.24) |  | 0.18 (0.09, 0.34) | 0.38 (0.18, 0.78) | 2.41 (-2.05, 6.97) |  | 0.24 (0.14, 0.37) | 0.45 (0.21, 0.73) | 2.03 (-1.83, 5.33) |
| LCOT |  | 0.11 (0.07, 0.17) | 0.17 (0.09, 0.28) | 1.4 (-2.05, 4.47) |  | 0.09 (0.04, 0.16) | 0.14 (0.06, 0.3) | 1.43 (-3.16, 6.5) |  | 0.13 (0.08, 0.2) | 0.19 (0.09, 0.33) | 1.22 (-2.58, 4.57) |
| **Fiji** |  |  |  |  |  |  |  |  |  |  |  |  |
| LCHB |  | 1.25 (0.87, 1.83) | 2.05 (1.33, 2.97) | 1.6 (-1.03, 3.96) |  | 1.79 (1.18, 2.66) | 3.04 (1.91, 4.67) | 1.71 (-1.07, 4.44) |  | 0.69 (0.41, 1.16) | 1.03 (0.65, 1.54) | 1.29 (-1.87, 4.27) |
| LCHC |  | 0.38 (0.22, 0.66) | 0.81 (0.52, 1.22) | 2.44 (-0.77, 5.53) |  | 0.35 (0.2, 0.57) | 0.74 (0.42, 1.22) | 2.42 (-0.99, 5.83) |  | 0.42 (0.23, 0.77) | 0.88 (0.58, 1.29) | 2.39 (-0.91, 5.56) |
| LCAL |  | 0.22 (0.13, 0.35) | 0.48 (0.28, 0.78) | 2.52 (-0.72, 5.78) |  | 0.32 (0.18, 0.53) | 0.73 (0.4, 1.19) | 2.66 (-0.91, 6.09) |  | 0.1 (0.05, 0.2) | 0.22 (0.13, 0.35) | 2.54 (-1.39, 6.28) |
| LCNA |  | 0.18 (0.11, 0.29) | 0.45 (0.28, 0.7) | 2.96 (-0.11, 5.97) |  | 0.16 (0.1, 0.27) | 0.43 (0.24, 0.71) | 3.19 (-0.38, 6.32) |  | 0.19 (0.11, 0.35) | 0.47 (0.29, 0.72) | 2.92 (-0.61, 6.06) |
| LCOT |  | 0.1 (0.06, 0.16) | 0.16 (0.09, 0.26) | 1.52 (-1.86, 4.73) |  | 0.08 (0.04, 0.14) | 0.14 (0.08, 0.25) | 1.81 (-1.81, 5.91) |  | 0.11 (0.06, 0.2) | 0.19 (0.11, 0.29) | 1.76 (-1.93, 5.08) |
| **Guam** |  |  |  |  |  |  |  |  |  |  |  |  |
| LCHB |  | 1.07 (0.85, 1.31) | 3.98 (3.02, 5.04) | 4.24 (2.69, 5.74) |  | 1.62 (1.29, 1.98) | 6.86 (5.18, 8.66) | 4.66 (3.1, 6.14) |  | 0.43 (0.32, 0.56) | 1.03 (0.71, 1.41) | 2.82 (0.77, 4.78) |
| LCHC |  | 0.32 (0.21, 0.45) | 1.22 (0.8, 1.72) | 4.32 (1.86, 6.78) |  | 0.32 (0.2, 0.47) | 1.52 (0.96, 2.21) | 5.03 (2.3, 7.75) |  | 0.32 (0.21, 0.44) | 0.91 (0.59, 1.27) | 3.37 (0.95, 5.81) |
| LCAL |  | 0.22 (0.14, 0.32) | 1 (0.66, 1.5) | 4.88 (2.34, 7.65) |  | 0.34 (0.22, 0.5) | 1.74 (1.11, 2.63) | 5.27 (2.57, 8) |  | 0.08 (0.05, 0.12) | 0.25 (0.16, 0.38) | 3.68 (0.93, 6.54) |
| LCNA |  | 0.17 (0.12, 0.25) | 0.79 (0.54, 1.14) | 4.96 (2.48, 7.26) |  | 0.18 (0.12, 0.27) | 1.03 (0.68, 1.51) | 5.63 (2.98, 8.17) |  | 0.16 (0.11, 0.23) | 0.54 (0.35, 0.81) | 3.92 (1.35, 6.44) |
| LCOT |  | 0.07 (0.05, 0.1) | 0.22 (0.14, 0.32) | 3.69 (1.09, 5.99) |  | 0.07 (0.05, 0.11) | 0.28 (0.17, 0.44) | 4.47 (1.4, 7.02) |  | 0.07 (0.05, 0.1) | 0.16 (0.1, 0.23) | 2.67 (0, 4.92) |
| **Japan** |  |  |  |  |  |  |  |  |  |  |  |  |
| LCHB |  | 2.95 (2.49, 3.41) | 3.18 (2.52, 3.89) | 0.24 (-0.98, 1.44) |  | 4.83 (4.12, 5.62) | 4.74 (3.85, 5.75) | -0.06 (-1.22, 1.08) |  | 1.12 (0.94, 1.32) | 1.7 (1.24, 2.15) | 1.35 (-0.2, 2.67) |
| LCHC |  | 12.71 (11.88, 13.49) | 22.03 (18.79, 24.27) | 1.77 (1.07, 2.3) |  | 18.77 (17.55, 19.96) | 29.22 (26.25, 31.85) | 1.43 (0.88, 1.92) |  | 6.84 (6.19, 7.28) | 15.21 (11.18, 17.82) | 2.58 (1.38, 3.41) |
| LCAL |  | 2.6 (2.25, 3.09) | 3.26 (2.73, 3.93) | 0.73 (-0.4, 1.8) |  | 4.52 (3.91, 5.34) | 5.45 (4.57, 6.54) | 0.6 (-0.5, 1.66) |  | 0.75 (0.64, 0.9) | 1.17 (0.89, 1.49) | 1.43 (-0.04, 2.73) |
| LCNA |  | 0.8 (0.69, 0.93) | 1.44 (1.09, 1.78) | 1.9 (0.51, 3.06) |  | 0.95 (0.8, 1.1) | 1.45 (1.15, 1.77) | 1.36 (0.14, 2.56) |  | 0.66 (0.55, 0.78) | 1.42 (1.01, 1.84) | 2.47 (0.83, 3.9) |
| LCOT |  | 0.55 (0.47, 0.65) | 0.74 (0.57, 0.9) | 0.96 (-0.42, 2.1) |  | 0.69 (0.58, 0.82) | 0.78 (0.64, 0.94) | 0.4 (-0.8, 1.56) |  | 0.42 (0.36, 0.49) | 0.7 (0.5, 0.9) | 1.65 (0.07, 2.96) |
| **Kiribati** |  |  |  |  |  |  |  |  |  |  |  |  |
| LCHB |  | 1.96 (1.4, 2.71) | 2.03 (1.37, 3) | 0.11 (-2.2, 2.46) |  | 2.33 (1.61, 3.17) | 2.31 (1.43, 3.75) | -0.03 (-2.57, 2.73) |  | 1.6 (1.1, 2.33) | 1.75 (1.13, 2.7) | 0.29 (-2.33, 2.9) |
| LCHC |  | 0.72 (0.49, 1.04) | 0.87 (0.52, 1.32) | 0.61 (-2.24, 3.2) |  | 0.43 (0.26, 0.67) | 0.44 (0.24, 0.78) | 0.07 (-3.31, 3.54) |  | 1.02 (0.67, 1.48) | 1.28 (0.74, 1.88) | 0.73 (-2.24, 3.33) |
| LCAL |  | 0.31 (0.19, 0.48) | 0.34 (0.2, 0.56) | 0.3 (-2.82, 3.49) |  | 0.39 (0.24, 0.62) | 0.39 (0.22, 0.7) | 0 (-3.34, 3.45) |  | 0.23 (0.13, 0.36) | 0.28 (0.16, 0.46) | 0.63 (-2.62, 4.08) |
| LCNA |  | 0.32 (0.21, 0.49) | 0.41 (0.26, 0.59) | 0.8 (-2.04, 3.33) |  | 0.2 (0.12, 0.32) | 0.23 (0.13, 0.39) | 0.45 (-2.91, 3.8) |  | 0.45 (0.28, 0.68) | 0.6 (0.36, 0.89) | 0.93 (-2.05, 3.73) |
| LCOT |  | 0.18 (0.12, 0.28) | 0.21 (0.13, 0.33) | 0.5 (-2.48, 3.26) |  | 0.1 (0.06, 0.16) | 0.1 (0.05, 0.19) | 0 (-3.75, 3.72) |  | 0.26 (0.16, 0.41) | 0.31 (0.19, 0.5) | 0.57 (-2.48, 3.68) |
| **Laos** |  |  |  |  |  |  |  |  |  |  |  |  |
| LCHB |  | 2.5 (1.53, 3.65) | 1.87 (1.19, 2.81) | -0.94 (-3.62, 1.96) |  | 3.95 (2.33, 5.89) | 2.87 (1.75, 4.41) | -1.03 (-3.91, 2.06) |  | 1.09 (0.68, 1.61) | 0.85 (0.5, 1.3) | -0.8 (-3.77, 2.09) |
| LCHC |  | 1.06 (0.63, 1.63) | 0.87 (0.55, 1.34) | -0.64 (-3.5, 2.43) |  | 1.15 (0.63, 1.87) | 0.89 (0.51, 1.45) | -0.83 (-4.19, 2.69) |  | 0.98 (0.58, 1.48) | 0.85 (0.54, 1.24) | -0.46 (-3.25, 2.45) |
| LCAL |  | 1.09 (0.64, 1.71) | 1.12 (0.67, 1.67) | 0.09 (-3.02, 3.09) |  | 1.83 (1.02, 2.97) | 1.82 (1.05, 2.78) | -0.02 (-3.35, 3.23) |  | 0.37 (0.22, 0.59) | 0.42 (0.24, 0.66) | 0.41 (-2.9, 3.54) |
| LCNA |  | 0.36 (0.22, 0.57) | 0.34 (0.19, 0.53) | -0.18 (-3.54, 2.84) |  | 0.39 (0.2, 0.67) | 0.35 (0.19, 0.57) | -0.35 (-4.07, 3.38) |  | 0.33 (0.2, 0.52) | 0.32 (0.19, 0.49) | -0.1 (-3.25, 2.89) |
| LCOT |  | 0.15 (0.09, 0.23) | 0.12 (0.07, 0.19) | -0.72 (-3.84, 2.41) |  | 0.17 (0.09, 0.27) | 0.12 (0.07, 0.2) | -1.12 (-4.35, 2.58) |  | 0.14 (0.09, 0.22) | 0.12 (0.07, 0.19) | -0.5 (-3.69, 2.41) |
| **Malaysia** |  |  |  |  |  |  |  |  |  |  |  |  |
| LCHB |  | 1.32 (0.97, 1.71) | 2.78 (2, 3.73) | 2.4 (0.51, 4.34) |  | 2.07 (1.5, 2.74) | 4.55 (3.24, 6.14) | 2.54 (0.54, 4.55) |  | 0.55 (0.39, 0.74) | 0.89 (0.58, 1.25) | 1.55 (-0.79, 3.76) |
| LCHC |  | 0.38 (0.25, 0.54) | 0.87 (0.57, 1.24) | 2.67 (0.17, 5.17) |  | 0.35 (0.21, 0.51) | 0.92 (0.56, 1.38) | 3.12 (0.3, 6.07) |  | 0.41 (0.27, 0.58) | 0.82 (0.56, 1.17) | 2.24 (-0.11, 4.73) |
| LCAL |  | 0.3 (0.19, 0.44) | 0.71 (0.44, 1.11) | 2.78 (0, 5.69) |  | 0.46 (0.29, 0.68) | 1.14 (0.71, 1.81) | 2.93 (0.14, 5.91) |  | 0.13 (0.08, 0.21) | 0.25 (0.15, 0.4) | 2.11 (-1.09, 5.19) |
| LCNA |  | 0.19 (0.13, 0.28) | 0.56 (0.37, 0.82) | 3.49 (0.9, 5.94) |  | 0.19 (0.12, 0.28) | 0.61 (0.38, 0.94) | 3.76 (0.99, 6.64) |  | 0.2 (0.13, 0.3) | 0.5 (0.33, 0.73) | 2.96 (0.31, 5.57) |
| LCOT |  | 0.07 (0.05, 0.1) | 0.14 (0.09, 0.21) | 2.24 (-0.34, 4.63) |  | 0.06 (0.04, 0.1) | 0.15 (0.09, 0.23) | 2.96 (-0.34, 5.64) |  | 0.07 (0.05, 0.1) | 0.13 (0.08, 0.19) | 2 (-0.72, 4.31) |
| **Marshall Islands** |  |  |  |  |  |  |  |  |  |  |  |  |
| LCHB |  | 0.7 (0.4, 1.28) | 1.42 (0.76, 2.65) | 2.28 (-1.68, 6.1) |  | 0.87 (0.49, 1.69) | 1.91 (0.9, 4.1) | 2.54 (-2.03, 6.85) |  | 0.51 (0.29, 0.95) | 0.91 (0.54, 1.45) | 1.87 (-1.82, 5.19) |
| LCHC |  | 0.25 (0.13, 0.45) | 0.49 (0.27, 0.87) | 2.17 (-1.65, 6.13) |  | 0.17 (0.08, 0.35) | 0.38 (0.16, 0.9) | 2.59 (-2.53, 7.81) |  | 0.33 (0.17, 0.62) | 0.6 (0.33, 1.04) | 1.93 (-2.03, 5.84) |
| LCAL |  | 0.13 (0.07, 0.25) | 0.3 (0.15, 0.59) | 2.7 (-1.65, 6.88) |  | 0.17 (0.08, 0.36) | 0.42 (0.19, 0.95) | 2.92 (-2.06, 7.98) |  | 0.08 (0.04, 0.17) | 0.18 (0.1, 0.3) | 2.62 (-1.71, 6.5) |
| LCNA |  | 0.1 (0.05, 0.19) | 0.24 (0.13, 0.38) | 2.82 (-1.22, 6.54) |  | 0.07 (0.03, 0.15) | 0.19 (0.09, 0.41) | 3.22 (-1.65, 8.44) |  | 0.13 (0.07, 0.27) | 0.29 (0.17, 0.47) | 2.59 (-1.49, 6.14) |
| LCOT |  | 0.06 (0.03, 0.11) | 0.12 (0.07, 0.21) | 2.24 (-1.46, 6.28) |  | 0.04 (0.02, 0.08) | 0.09 (0.04, 0.19) | 2.62 (-2.24, 7.26) |  | 0.09 (0.05, 0.17) | 0.16 (0.09, 0.29) | 1.86 (-2.05, 5.67) |
| **Mongolia** |  |  |  |  |  |  |  |  |  |  |  |  |
| LCHB |  | 9.47 (6.2, 14.24) | 13.48 (8.72, 20.23) | 1.14 (-1.58, 3.81) |  | 13.91 (8.92, 21.01) | 19.32 (12.4, 28.69) | 1.06 (-1.7, 3.77) |  | 5.04 (2.98, 8.05) | 7.78 (4.94, 11.78) | 1.4 (-1.58, 4.43) |
| LCHC |  | 11.32 (7.25, 17.2) | 20.86 (14.66, 28.78) | 1.97 (-0.52, 4.45) |  | 12.27 (7.46, 19.43) | 17.7 (11.82, 25.98) | 1.18 (-1.6, 4.03) |  | 10.38 (6.52, 16.01) | 23.93 (17.03, 32.02) | 2.69 (0.2, 5.13) |
| LCAL |  | 5.15 (3.1, 8.37) | 11.84 (7.97, 17.84) | 2.69 (-0.16, 5.65) |  | 8.15 (4.96, 13.42) | 17.03 (11.16, 25.87) | 2.38 (-0.59, 5.33) |  | 2.15 (1.24, 3.65) | 6.78 (4.41, 10.66) | 3.7 (0.61, 6.94) |
| LCNA |  | 1.5 (0.92, 2.36) | 3.31 (2.06, 5.13) | 2.55 (-0.44, 5.54) |  | 1.43 (0.83, 2.34) | 2.53 (1.46, 3.99) | 1.84 (-1.52, 5.06) |  | 1.58 (0.95, 2.52) | 4.06 (2.55, 6.3) | 3.04 (0.04, 6.1) |
| LCOT |  | 0.89 (0.54, 1.44) | 1.45 (0.92, 2.24) | 1.57 (-1.45, 4.59) |  | 0.77 (0.44, 1.28) | 1.08 (0.65, 1.75) | 1.09 (-2.19, 4.45) |  | 1.01 (0.59, 1.65) | 1.81 (1.16, 2.75) | 1.88 (-1.14, 4.97) |
| **Nauru** |  |  |  |  |  |  |  |  |  |  |  |  |
| LCHB |  | 2.25 (1.55, 3.09) | 2.04 (1.17, 3.25) | -0.32 (-3.13, 2.39) |  | 3.52 (2.37, 4.93) | 3.21 (1.76, 5.35) | -0.3 (-3.32, 2.63) |  | 0.88 (0.58, 1.29) | 0.86 (0.47, 1.42) | -0.07 (-3.26, 2.89) |
| LCHC |  | 0.5 (0.31, 0.76) | 0.51 (0.29, 0.84) | 0.06 (-3.11, 3.22) |  | 0.53 (0.31, 0.84) | 0.51 (0.25, 0.93) | -0.12 (-3.91, 3.54) |  | 0.46 (0.29, 0.71) | 0.52 (0.27, 0.89) | 0.4 (-3.12, 3.62) |
| LCAL |  | 0.41 (0.25, 0.63) | 0.45 (0.25, 0.76) | 0.3 (-2.98, 3.59) |  | 0.66 (0.39, 1.03) | 0.71 (0.37, 1.27) | 0.24 (-3.3, 3.81) |  | 0.14 (0.09, 0.22) | 0.19 (0.1, 0.32) | 0.99 (-2.54, 4.09) |
| LCNA |  | 0.28 (0.17, 0.41) | 0.31 (0.18, 0.49) | 0.33 (-2.66, 3.41) |  | 0.3 (0.18, 0.49) | 0.32 (0.17, 0.56) | 0.21 (-3.41, 3.66) |  | 0.25 (0.15, 0.37) | 0.31 (0.17, 0.5) | 0.69 (-2.51, 3.88) |
| LCOT |  | 0.15 (0.09, 0.22) | 0.15 (0.09, 0.25) | 0 (-2.88, 3.3) |  | 0.15 (0.09, 0.24) | 0.15 (0.07, 0.27) | 0 (-3.97, 3.54) |  | 0.14 (0.09, 0.22) | 0.16 (0.08, 0.28) | 0.43 (-3.26, 3.66) |
| **New Zealand** |  |  |  |  |  |  |  |  |  |  |  |  |
| LCHB |  | 0.47 (0.39, 0.56) | 1.16 (0.94, 1.42) | 2.91 (1.67, 4.17) |  | 0.72 (0.58, 0.88) | 1.8 (1.45, 2.26) | 2.96 (1.61, 4.39) |  | 0.22 (0.18, 0.26) | 0.51 (0.41, 0.63) | 2.71 (1.47, 4.04) |
| LCHC |  | 0.73 (0.62, 0.85) | 2.56 (2.17, 2.95) | 4.05 (3.02, 5.03) |  | 0.72 (0.59, 0.88) | 2.53 (2.07, 3.03) | 4.05 (2.76, 5.28) |  | 0.73 (0.61, 0.84) | 2.58 (2.11, 3.04) | 4.07 (2.97, 5.18) |
| LCAL |  | 1.14 (0.98, 1.29) | 3.16 (2.7, 3.66) | 3.29 (2.38, 4.25) |  | 1.85 (1.58, 2.14) | 5.12 (4.24, 5.95) | 3.28 (2.21, 4.28) |  | 0.43 (0.36, 0.52) | 1.21 (1, 1.48) | 3.34 (2.11, 4.56) |
| LCNA |  | 0.22 (0.19, 0.26) | 0.94 (0.78, 1.12) | 4.68 (3.54, 5.72) |  | 0.21 (0.17, 0.25) | 0.91 (0.73, 1.1) | 4.73 (3.46, 6.02) |  | 0.24 (0.19, 0.28) | 0.98 (0.76, 1.18) | 4.54 (3.22, 5.89) |
| LCOT |  | 0.13 (0.11, 0.15) | 0.39 (0.33, 0.46) | 3.54 (2.54, 4.62) |  | 0.12 (0.1, 0.15) | 0.37 (0.3, 0.45) | 3.63 (2.24, 4.85) |  | 0.14 (0.12, 0.16) | 0.41 (0.34, 0.49) | 3.47 (2.43, 4.54) |
| **Niue** |  |  |  |  |  |  |  |  |  |  |  |  |
| LCHB |  | 1.97 (1.26, 3) | 3.04 (1.84, 4.99) | 1.4 (-1.58, 4.44) |  | 2.91 (1.84, 4.58) | 4.92 (2.87, 8.52) | 1.69 (-1.51, 4.94) |  | 1.02 (0.62, 1.65) | 1.12 (0.65, 1.79) | 0.3 (-3.01, 3.42) |
| LCHC |  | 0.88 (0.53, 1.39) | 1.31 (0.78, 2.06) | 1.28 (-1.86, 4.38) |  | 0.72 (0.42, 1.2) | 1.36 (0.73, 2.42) | 2.05 (-1.6, 5.65) |  | 1.04 (0.62, 1.69) | 1.26 (0.7, 1.96) | 0.62 (-2.84, 3.71) |
| LCAL |  | 0.49 (0.29, 0.79) | 0.97 (0.56, 1.72) | 2.2 (-1.11, 5.74) |  | 0.74 (0.41, 1.21) | 1.59 (0.9, 2.99) | 2.47 (-0.95, 6.41) |  | 0.24 (0.14, 0.42) | 0.34 (0.19, 0.57) | 1.12 (-2.56, 4.53) |
| LCNA |  | 0.39 (0.23, 0.63) | 0.78 (0.46, 1.24) | 2.24 (-1.01, 5.43) |  | 0.35 (0.2, 0.59) | 0.86 (0.46, 1.55) | 2.9 (-0.8, 6.61) |  | 0.43 (0.25, 0.72) | 0.69 (0.38, 1.1) | 1.53 (-2.06, 4.78) |
| LCOT |  | 0.15 (0.09, 0.24) | 0.22 (0.12, 0.35) | 1.24 (-2.24, 4.38) |  | 0.13 (0.07, 0.22) | 0.23 (0.11, 0.43) | 1.84 (-2.24, 5.86) |  | 0.17 (0.1, 0.29) | 0.2 (0.11, 0.33) | 0.52 (-3.13, 3.85) |
| **Northern Mariana Islands** |  |  |  |  |  |  |  |  |  |  |  |  |
| LCHB |  | 1.39 (0.96, 2.02) | 3.94 (2.91, 5.22) | 3.36 (1.18, 5.46) |  | 1.98 (1.33, 2.93) | 6 (4.46, 8.03) | 3.58 (1.36, 5.8) |  | 0.74 (0.48, 1.09) | 1.66 (1.1, 2.37) | 2.61 (0.03, 5.15) |
| LCHC |  | 0.34 (0.22, 0.52) | 1.31 (0.81, 1.9) | 4.35 (1.43, 6.95) |  | 0.3 (0.18, 0.49) | 1.34 (0.78, 2.07) | 4.83 (1.5, 7.88) |  | 0.38 (0.24, 0.55) | 1.27 (0.83, 1.78) | 3.89 (1.33, 6.46) |
| LCAL |  | 0.23 (0.14, 0.35) | 0.96 (0.6, 1.55) | 4.61 (1.74, 7.76) |  | 0.34 (0.2, 0.52) | 1.5 (0.89, 2.54) | 4.79 (1.73, 8.2) |  | 0.11 (0.06, 0.17) | 0.36 (0.21, 0.57) | 3.82 (0.68, 7.26) |
| LCNA |  | 0.24 (0.16, 0.36) | 0.92 (0.57, 1.35) | 4.33 (1.48, 6.88) |  | 0.22 (0.13, 0.36) | 0.98 (0.59, 1.51) | 4.82 (1.59, 7.91) |  | 0.27 (0.17, 0.4) | 0.85 (0.52, 1.26) | 3.7 (0.85, 6.46) |
| LCOT |  | 0.1 (0.06, 0.15) | 0.24 (0.15, 0.38) | 2.82 (0, 5.95) |  | 0.08 (0.05, 0.14) | 0.24 (0.14, 0.39) | 3.54 (0, 6.63) |  | 0.12 (0.07, 0.18) | 0.25 (0.14, 0.4) | 2.37 (-0.81, 5.62) |
| **Palau** |  |  |  |  |  |  |  |  |  |  |  |  |
| LCHB |  | 3.12 (1.99, 4.77) | 6.14 (4.1, 8.77) | 2.18 (-0.49, 4.78) |  | 6.05 (3.86, 9.24) | 11.14 (7.44, 15.92) | 1.97 (-0.7, 4.57) |  | 0 (0, 0.01) | 0.01 (0, 0.01) | - |
| LCHC |  | 0.63 (0.34, 1.06) | 1.29 (0.73, 2.04) | 2.31 (-1.2, 5.78) |  | 1.22 (0.66, 2.06) | 2.34 (1.33, 3.69) | 2.1 (-1.41, 5.55) |  | 0 (0, 0.01) | 0.01 (0, 0.01) | - |
| LCAL |  | 0.66 (0.39, 1.1) | 1.53 (0.89, 2.38) | 2.71 (-0.68, 5.83) |  | 1.28 (0.76, 2.13) | 2.78 (1.62, 4.32) | 2.5 (-0.88, 5.61) |  | 0 (0, 0) | 0 (0, 0) | - |
| LCNA |  | 0.35 (0.21, 0.59) | 0.92 (0.51, 1.45) | 3.12 (-0.47, 6.23) |  | 0.67 (0.4, 1.15) | 1.66 (0.93, 2.63) | 2.93 (-0.68, 6.08) |  | 0 (0, 0) | 0 (0, 0) | - |
| LCOT |  | 0.13 (0.08, 0.24) | 0.26 (0.15, 0.42) | 2.24 (-1.52, 5.35) |  | 0.26 (0.15, 0.47) | 0.48 (0.27, 0.77) | 1.98 (-1.79, 5.28) |  | 0 (0, 0) | 0 (0, 0) | - |
| **Papua New Guinea** |  |  |  |  |  |  |  |  |  |  |  |  |
| LCHB |  | 1.03 (0.48, 2.56) | 0.74 (0.33, 1.98) | -1.07 (-6.61, 4.57) |  | 1.46 (0.7, 4.03) | 1.06 (0.45, 3.18) | -1.03 (-7.07, 4.88) |  | 0.56 (0.22, 1.28) | 0.4 (0.19, 0.84) | -1.09 (-6.15, 4.32) |
| LCHC |  | 0.32 (0.13, 0.81) | 0.24 (0.1, 0.58) | -0.93 (-6.75, 4.82) |  | 0.29 (0.12, 0.83) | 0.22 (0.08, 0.64) | -0.89 (-7.55, 5.4) |  | 0.35 (0.14, 0.86) | 0.27 (0.12, 0.6) | -0.84 (-6.35, 4.69) |
| LCAL |  | 0.2 (0.09, 0.56) | 0.15 (0.06, 0.42) | -0.93 (-7.21, 4.97) |  | 0.29 (0.13, 0.88) | 0.22 (0.08, 0.7) | -0.89 (-7.74, 5.43) |  | 0.09 (0.03, 0.23) | 0.07 (0.03, 0.16) | -0.81 (-6.57, 5.4) |
| LCNA |  | 0.12 (0.05, 0.3) | 0.1 (0.04, 0.25) | -0.59 (-6.5, 5.19) |  | 0.11 (0.05, 0.32) | 0.09 (0.03, 0.28) | -0.65 (-7.64, 5.56) |  | 0.13 (0.05, 0.32) | 0.11 (0.05, 0.24) | -0.54 (-5.99, 5.06) |
| LCOT |  | 0.08 (0.03, 0.21) | 0.06 (0.03, 0.13) | -0.93 (-6.28, 4.73) |  | 0.06 (0.03, 0.18) | 0.05 (0.02, 0.15) | -0.59 (-7.09, 5.19) |  | 0.09 (0.03, 0.24) | 0.07 (0.03, 0.15) | -0.81 (-6.71, 5.19) |
| **Philippines** |  |  |  |  |  |  |  |  |  |  |  |  |
| LCHB |  | 1.94 (1.35, 2.48) | 2.02 (1.62, 2.53) | 0.13 (-1.37, 2.03) |  | 3.23 (2.21, 4.07) | 3.2 (2.44, 4.2) | -0.03 (-1.65, 2.07) |  | 0.62 (0.42, 1) | 0.8 (0.61, 1.02) | 0.82 (-1.59, 2.86) |
| LCHC |  | 0.53 (0.36, 0.78) | 0.84 (0.65, 1.04) | 1.49 (-0.59, 3.42) |  | 0.63 (0.41, 0.88) | 0.88 (0.65, 1.15) | 1.08 (-0.98, 3.33) |  | 0.43 (0.29, 0.72) | 0.79 (0.61, 0.99) | 1.96 (-0.53, 3.96) |
| LCAL |  | 0.72 (0.48, 0.99) | 1.14 (0.87, 1.46) | 1.48 (-0.42, 3.59) |  | 1.21 (0.77, 1.64) | 1.82 (1.32, 2.39) | 1.32 (-0.7, 3.65) |  | 0.23 (0.15, 0.38) | 0.44 (0.33, 0.58) | 2.09 (-0.46, 4.36) |
| LCNA |  | 0.23 (0.16, 0.33) | 0.38 (0.3, 0.48) | 1.62 (-0.31, 3.54) |  | 0.28 (0.18, 0.38) | 0.42 (0.31, 0.55) | 1.31 (-0.66, 3.6) |  | 0.18 (0.12, 0.3) | 0.34 (0.25, 0.44) | 2.05 (-0.59, 4.19) |
| LCOT |  | 0.11 (0.08, 0.15) | 0.13 (0.1, 0.17) | 0.54 (-1.31, 2.43) |  | 0.14 (0.09, 0.18) | 0.15 (0.11, 0.2) | 0.22 (-1.59, 2.58) |  | 0.08 (0.05, 0.12) | 0.11 (0.08, 0.14) | 1.03 (-1.31, 3.32) |
| **Samoa** |  |  |  |  |  |  |  |  |  |  |  |  |
| LCHB |  | 1.46 (1.04, 1.97) | 1.69 (1.14, 2.39) | 0.47 (-1.76, 2.68) |  | 2.33 (1.65, 3.19) | 2.69 (1.8, 3.84) | 0.46 (-1.85, 2.72) |  | 0.49 (0.32, 0.71) | 0.64 (0.38, 1.01) | 0.86 (-2.02, 3.71) |
| LCHC |  | 0.48 (0.3, 0.71) | 0.62 (0.38, 0.92) | 0.83 (-2.02, 3.61) |  | 0.54 (0.32, 0.87) | 0.65 (0.38, 1.06) | 0.6 (-2.67, 3.86) |  | 0.41 (0.26, 0.59) | 0.58 (0.35, 0.86) | 1.12 (-1.68, 3.86) |
| LCAL |  | 0.34 (0.21, 0.54) | 0.41 (0.25, 0.64) | 0.6 (-2.48, 3.59) |  | 0.56 (0.33, 0.87) | 0.66 (0.38, 1.06) | 0.53 (-2.67, 3.76) |  | 0.1 (0.06, 0.16) | 0.14 (0.08, 0.23) | 1.09 (-2.24, 4.33) |
| LCNA |  | 0.25 (0.15, 0.38) | 0.34 (0.21, 0.5) | 0.99 (-1.91, 3.88) |  | 0.29 (0.16, 0.47) | 0.37 (0.22, 0.61) | 0.79 (-2.45, 4.32) |  | 0.2 (0.12, 0.31) | 0.3 (0.17, 0.44) | 1.31 (-1.94, 4.19) |
| LCOT |  | 0.1 (0.06, 0.15) | 0.12 (0.07, 0.18) | 0.59 (-2.46, 3.54) |  | 0.11 (0.06, 0.17) | 0.13 (0.07, 0.2) | 0.54 (-2.86, 3.88) |  | 0.08 (0.05, 0.13) | 0.11 (0.06, 0.18) | 1.03 (-2.49, 4.13) |
| **Singapore** |  |  |  |  |  |  |  |  |  |  |  |  |
| LCHB |  | 4.47 (3.8, 5.2) | 6.59 (5.27, 7.98) | 1.25 (0.04, 2.39) |  | 7.19 (6.19, 8.31) | 10.31 (8.32, 12.32) | 1.16 (0, 2.22) |  | 1.71 (1.32, 2.12) | 2.74 (2.04, 3.62) | 1.52 (-0.12, 3.25) |
| LCHC |  | 1.26 (0.86, 1.73) | 2.7 (1.83, 3.74) | 2.46 (0.18, 4.74) |  | 1.39 (0.91, 2.02) | 2.98 (1.93, 4.33) | 2.46 (-0.15, 5.03) |  | 1.12 (0.78, 1.49) | 2.41 (1.67, 3.2) | 2.47 (0.37, 4.55) |
| LCAL |  | 0.41 (0.26, 0.6) | 0.81 (0.52, 1.24) | 2.2 (-0.46, 5.04) |  | 0.68 (0.44, 1.02) | 1.33 (0.84, 2.05) | 2.16 (-0.63, 4.96) |  | 0.13 (0.08, 0.19) | 0.27 (0.16, 0.41) | 2.36 (-0.55, 5.27) |
| LCNA |  | 0.26 (0.17, 0.39) | 0.6 (0.39, 0.92) | 2.7 (0, 5.45) |  | 0.3 (0.19, 0.46) | 0.65 (0.39, 1.05) | 2.49 (-0.53, 5.51) |  | 0.23 (0.15, 0.34) | 0.55 (0.36, 0.82) | 2.81 (0.18, 5.48) |
| LCOT |  | 0.16 (0.11, 0.23) | 0.28 (0.18, 0.4) | 1.81 (-0.79, 4.16) |  | 0.19 (0.13, 0.28) | 0.3 (0.19, 0.46) | 1.47 (-1.25, 4.08) |  | 0.14 (0.09, 0.19) | 0.25 (0.16, 0.35) | 1.87 (-0.55, 4.38) |
| **Solomon Islands** |  |  |  |  |  |  |  |  |  |  |  |  |
| LCHB |  | 1.78 (0.55, 3.83) | 1.62 (0.99, 2.66) | -0.3 (-4.36, 5.08) |  | 2.91 (0.87, 6.3) | 2.57 (1.55, 4.45) | -0.4 (-4.52, 5.27) |  | 0.56 (0.18, 1.21) | 0.63 (0.38, 1.12) | 0.38 (-3.74, 5.9) |
| LCHC |  | 0.41 (0.15, 0.96) | 0.42 (0.24, 0.75) | 0.08 (-4.47, 5.19) |  | 0.5 (0.15, 1.24) | 0.44 (0.24, 0.84) | -0.41 (-5.3, 5.56) |  | 0.31 (0.12, 0.72) | 0.4 (0.22, 0.73) | 0.82 (-3.82, 5.82) |
| LCAL |  | 0.3 (0.09, 0.72) | 0.3 (0.17, 0.52) | 0 (-4.66, 5.66) |  | 0.5 (0.15, 1.21) | 0.48 (0.27, 0.87) | -0.13 (-4.84, 5.67) |  | 0.08 (0.03, 0.2) | 0.11 (0.06, 0.2) | 1.03 (-3.88, 6.12) |
| LCNA |  | 0.18 (0.06, 0.43) | 0.21 (0.12, 0.36) | 0.5 (-4.12, 5.78) |  | 0.22 (0.07, 0.56) | 0.23 (0.12, 0.45) | 0.14 (-4.97, 6) |  | 0.13 (0.05, 0.3) | 0.19 (0.11, 0.33) | 1.22 (-3.24, 6.09) |
| LCOT |  | 0.11 (0.04, 0.25) | 0.12 (0.07, 0.2) | 0.28 (-4.11, 5.19) |  | 0.12 (0.04, 0.29) | 0.12 (0.06, 0.21) | 0 (-5.08, 5.35) |  | 0.09 (0.03, 0.22) | 0.12 (0.07, 0.2) | 0.93 (-3.69, 6.12) |
| **South Korea** |  |  |  |  |  |  |  |  |  |  |  |  |
| LCHB |  | 16.84 (12.09, 21.63) | 20.09 (15.83, 25.37) | 0.57 (-1.01, 2.39) |  | 28.6 (20.36, 37.21) | 34.54 (27.25, 43.89) | 0.61 (-1, 2.48) |  | 4.96 (3.29, 6.82) | 5.52 (3.77, 7.83) | 0.35 (-1.91, 2.8) |
| LCHC |  | 3.49 (2.27, 5.13) | 6.36 (4.27, 9.15) | 1.94 (-0.59, 4.5) |  | 2.6 (1.57, 4.1) | 5.24 (3.17, 8.12) | 2.26 (-0.83, 5.3) |  | 4.39 (2.77, 6.4) | 7.48 (4.98, 10.42) | 1.72 (-0.81, 4.27) |
| LCAL |  | 3.14 (1.95, 4.52) | 6.1 (4, 9.03) | 2.14 (-0.39, 4.94) |  | 4.86 (2.94, 7.24) | 9.83 (6.32, 14.74) | 2.27 (-0.44, 5.2) |  | 1.41 (0.82, 2.18) | 2.35 (1.43, 3.51) | 1.65 (-1.36, 4.69) |
| LCNA |  | 1.32 (0.84, 1.97) | 2.51 (1.62, 3.84) | 2.07 (-0.63, 4.9) |  | 1.19 (0.72, 1.84) | 2.43 (1.44, 3.87) | 2.3 (-0.79, 5.43) |  | 1.44 (0.88, 2.2) | 2.6 (1.63, 4.05) | 1.91 (-0.97, 4.92) |
| LCOT |  | 0.77 (0.49, 1.17) | 1.07 (0.68, 1.54) | 1.06 (-1.75, 3.69) |  | 0.77 (0.48, 1.21) | 1.13 (0.71, 1.73) | 1.24 (-1.72, 4.14) |  | 0.78 (0.48, 1.19) | 1.01 (0.61, 1.48) | 0.83 (-2.16, 3.63) |
| **Tokelau** |  |  |  |  |  |  |  |  |  |  |  |  |
| LCHB |  | 1.53 (0.88, 2.74) | 2.57 (1.53, 4.13) | 1.67 (-1.88, 4.99) |  | 1.85 (1.03, 3.65) | 3.74 (2.13, 6.23) | 2.27 (-1.74, 5.81) |  | 1.2 (0.63, 2.22) | 1.35 (0.78, 2.31) | 0.38 (-3.37, 4.19) |
| LCHC |  | 0.76 (0.41, 1.44) | 1.02 (0.57, 1.74) | 0.95 (-2.99, 4.66) |  | 0.51 (0.25, 1.06) | 0.94 (0.5, 1.62) | 1.97 (-2.42, 6.03) |  | 1.02 (0.55, 1.99) | 1.1 (0.64, 1.91) | 0.24 (-3.66, 4.02) |
| LCAL |  | 0.35 (0.17, 0.69) | 0.65 (0.35, 1.13) | 2 (-2.19, 6.11) |  | 0.46 (0.22, 0.99) | 0.98 (0.52, 1.72) | 2.44 (-2.08, 6.63) |  | 0.24 (0.11, 0.49) | 0.3 (0.15, 0.55) | 0.72 (-3.82, 5.19) |
| LCNA |  | 0.3 (0.16, 0.57) | 0.53 (0.29, 0.9) | 1.84 (-2.18, 5.57) |  | 0.21 (0.1, 0.45) | 0.51 (0.27, 0.91) | 2.86 (-1.65, 7.12) |  | 0.39 (0.2, 0.76) | 0.55 (0.29, 0.98) | 1.11 (-3.11, 5.13) |
| LCOT |  | 0.14 (0.07, 0.27) | 0.18 (0.1, 0.32) | 0.81 (-3.2, 4.9) |  | 0.08 (0.04, 0.17) | 0.16 (0.08, 0.29) | 2.24 (-2.43, 6.39) |  | 0.19 (0.1, 0.38) | 0.21 (0.12, 0.38) | 0.32 (-3.72, 4.31) |
| **Tonga** |  |  |  |  |  |  |  |  |  |  |  |  |
| LCHB |  | 6.96 (4.7, 10.05) | 8.49 (5.57, 12.47) | 0.64 (-1.9, 3.15) |  | 11.3 (7.81, 16.65) | 14.54 (9.44, 21.81) | 0.81 (-1.83, 3.31) |  | 2.54 (1.48, 4.18) | 2.46 (1.54, 3.67) | -0.1 (-3.22, 2.93) |
| LCHC |  | 2.16 (1.24, 3.54) | 3 (1.86, 4.54) | 1.06 (-2.08, 4.19) |  | 2.51 (1.43, 4.27) | 3.68 (2.12, 5.96) | 1.23 (-2.26, 4.6) |  | 1.8 (0.96, 3.14) | 2.32 (1.37, 3.4) | 0.82 (-2.68, 4.08) |
| LCAL |  | 1.35 (0.78, 2.24) | 1.87 (1.09, 3.02) | 1.05 (-2.32, 4.37) |  | 2.26 (1.3, 3.87) | 3.24 (1.89, 5.34) | 1.16 (-2.31, 4.56) |  | 0.43 (0.21, 0.75) | 0.5 (0.28, 0.81) | 0.49 (-3.18, 4.35) |
| LCNA |  | 1.08 (0.62, 1.78) | 1.75 (1.05, 2.62) | 1.56 (-1.7, 4.65) |  | 1.28 (0.75, 2.16) | 2.23 (1.28, 3.66) | 1.79 (-1.69, 5.11) |  | 0.87 (0.46, 1.52) | 1.27 (0.77, 1.96) | 1.22 (-2.19, 4.68) |
| LCOT |  | 0.46 (0.28, 0.76) | 0.57 (0.33, 0.89) | 0.69 (-2.69, 3.73) |  | 0.5 (0.28, 0.86) | 0.68 (0.39, 1.15) | 0.99 (-2.55, 4.56) |  | 0.41 (0.23, 0.73) | 0.45 (0.26, 0.73) | 0.3 (-3.33, 3.73) |
| **Tuvalu** |  |  |  |  |  |  |  |  |  |  |  |  |
| LCHB |  | 1.77 (1.15, 2.77) | 2.08 (1.3, 3.17) | 0.52 (-2.44, 3.27) |  | 2.47 (1.6, 4.03) | 3.01 (1.84, 4.97) | 0.64 (-2.53, 3.66) |  | 1.12 (0.64, 1.93) | 1.07 (0.66, 1.66) | -0.15 (-3.46, 3.07) |
| LCHC |  | 0.67 (0.39, 1.16) | 0.82 (0.5, 1.32) | 0.65 (-2.71, 3.93) |  | 0.53 (0.29, 0.98) | 0.7 (0.37, 1.24) | 0.9 (-3.14, 4.69) |  | 0.8 (0.45, 1.49) | 0.94 (0.6, 1.55) | 0.52 (-2.93, 3.99) |
| LCAL |  | 0.36 (0.2, 0.63) | 0.49 (0.27, 0.83) | 0.99 (-2.73, 4.59) |  | 0.52 (0.29, 0.93) | 0.72 (0.38, 1.27) | 1.05 (-2.89, 4.76) |  | 0.2 (0.1, 0.38) | 0.24 (0.13, 0.42) | 0.59 (-3.46, 4.63) |
| LCNA |  | 0.27 (0.16, 0.48) | 0.41 (0.25, 0.66) | 1.35 (-2.1, 4.57) |  | 0.23 (0.13, 0.41) | 0.37 (0.2, 0.67) | 1.53 (-2.32, 5.29) |  | 0.32 (0.17, 0.61) | 0.45 (0.27, 0.75) | 1.1 (-2.63, 4.79) |
| LCOT |  | 0.14 (0.08, 0.25) | 0.16 (0.1, 0.27) | 0.43 (-2.96, 3.92) |  | 0.1 (0.06, 0.19) | 0.14 (0.07, 0.26) | 1.09 (-3.22, 4.73) |  | 0.18 (0.1, 0.31) | 0.18 (0.11, 0.32) | 0 (-3.34, 3.75) |
| **Vanuatu** |  |  |  |  |  |  |  |  |  |  |  |  |
| LCHB |  | 1.2 (0.72, 2.26) | 1.37 (0.84, 2.24) | 0.43 (-3.19, 3.66) |  | 1.84 (1.12, 3.63) | 2.08 (1.24, 3.53) | 0.4 (-3.46, 3.7) |  | 0.53 (0.25, 1.12) | 0.64 (0.38, 1.07) | 0.61 (-3.49, 4.69) |
| LCHC |  | 0.34 (0.18, 0.66) | 0.46 (0.27, 0.78) | 0.98 (-2.88, 4.73) |  | 0.36 (0.19, 0.74) | 0.45 (0.23, 0.88) | 0.72 (-3.77, 4.94) |  | 0.33 (0.16, 0.72) | 0.48 (0.29, 0.78) | 1.21 (-2.93, 5.11) |
| LCAL |  | 0.21 (0.11, 0.43) | 0.28 (0.15, 0.47) | 0.93 (-3.4, 4.68) |  | 0.33 (0.18, 0.69) | 0.43 (0.21, 0.78) | 0.85 (-3.84, 4.73) |  | 0.08 (0.04, 0.18) | 0.12 (0.07, 0.2) | 1.31 (-3.05, 5.19) |
| LCNA |  | 0.15 (0.08, 0.3) | 0.22 (0.13, 0.38) | 1.24 (-2.7, 5.03) |  | 0.16 (0.08, 0.32) | 0.23 (0.11, 0.44) | 1.17 (-3.44, 5.5) |  | 0.14 (0.07, 0.3) | 0.22 (0.13, 0.37) | 1.46 (-2.7, 5.37) |
| LCOT |  | 0.08 (0.05, 0.17) | 0.1 (0.06, 0.18) | 0.72 (-3.36, 4.13) |  | 0.08 (0.05, 0.16) | 0.1 (0.05, 0.19) | 0.72 (-3.75, 4.31) |  | 0.09 (0.04, 0.19) | 0.11 (0.06, 0.19) | 0.65 (-3.72, 5.03) |
| **Viet Nam** |  |  |  |  |  |  |  |  |  |  |  |  |
| LCHB |  | 4.35 (2.84, 6.08) | 5.8 (3.81, 8.86) | 0.93 (-1.51, 3.67) |  | 7.41 (4.73, 10.54) | 9.6 (5.95, 15.41) | 0.84 (-1.84, 3.81) |  | 1.46 (0.96, 2.04) | 2.02 (1.22, 3.01) | 1.05 (-1.66, 3.69) |
| LCHC |  | 1.39 (0.87, 2.21) | 2.06 (1.2, 3.26) | 1.27 (-1.97, 4.26) |  | 1.92 (1.11, 3.1) | 2.68 (1.46, 4.65) | 1.08 (-2.43, 4.62) |  | 0.9 (0.57, 1.35) | 1.43 (0.83, 2.13) | 1.49 (-1.57, 4.25) |
| LCAL |  | 1.48 (0.92, 2.31) | 3.66 (2.22, 5.79) | 2.92 (-0.13, 5.93) |  | 2.61 (1.6, 4.07) | 6.25 (3.65, 10.03) | 2.82 (-0.35, 5.92) |  | 0.4 (0.25, 0.62) | 1.07 (0.64, 1.77) | 3.17 (0.1, 6.31) |
| LCNA |  | 0.58 (0.36, 0.91) | 1.01 (0.63, 1.53) | 1.79 (-1.19, 4.67) |  | 0.7 (0.4, 1.15) | 1.18 (0.7, 1.9) | 1.68 (-1.6, 5.03) |  | 0.46 (0.28, 0.73) | 0.85 (0.51, 1.29) | 1.98 (-1.16, 4.93) |
| LCOT |  | 0.23 (0.14, 0.34) | 0.33 (0.19, 0.52) | 1.16 (-1.88, 4.23) |  | 0.29 (0.17, 0.45) | 0.4 (0.22, 0.66) | 1.04 (-2.31, 4.38) |  | 0.18 (0.11, 0.27) | 0.27 (0.16, 0.42) | 1.31 (-1.69, 4.32) |
| **Western Pacific Region** |  |  |  |  |  |  |  |  |  |  |  |  |
| LCHB |  | 5.16 (4.39, 6.01) | 7.47 (5.98, 9.37) | 1.19 (-0.02, 2.45) |  | 8.48 (7.03, 10.13) | 12.51 (9.73, 16.16) | 1.25 (-0.13, 2.69) |  | 1.68 (1.34, 2.04) | 2.26 (1.73, 2.89) | 0.96 (-0.53, 2.48) |
| LCHC |  | 2.2 (1.94, 2.48) | 3.8 (3.19, 4.4) | 1.76 (0.81, 2.64) |  | 2.48 (2.2, 2.81) | 3.99 (3.37, 4.75) | 1.53 (0.59, 2.48) |  | 1.91 (1.6, 2.22) | 3.61 (2.88, 4.3) | 2.05 (0.84, 3.19) |
| LCAL |  | 0.92 (0.74, 1.13) | 1.81 (1.4, 2.34) | 2.18 (0.69, 3.71) |  | 1.32 (1.05, 1.65) | 2.63 (1.97, 3.48) | 2.22 (0.57, 3.87) |  | 0.49 (0.38, 0.61) | 0.97 (0.74, 1.22) | 2.2 (0.62, 3.76) |
| LCNA |  | 0.41 (0.34, 0.5) | 0.86 (0.68, 1.07) | 2.39 (0.99, 3.7) |  | 0.43 (0.34, 0.53) | 0.91 (0.67, 1.19) | 2.42 (0.76, 4.04) |  | 0.4 (0.31, 0.48) | 0.82 (0.64, 1.03) | 2.32 (0.93, 3.87) |
| LCOT |  | 0.42 (0.34, 0.51) | 0.6 (0.46, 0.75) | 1.15 (-0.33, 2.55) |  | 0.43 (0.34, 0.53) | 0.64 (0.47, 0.88) | 1.28 (-0.39, 3.07) |  | 0.4 (0.32, 0.5) | 0.55 (0.42, 0.7) | 1.03 (-0.56, 2.53) |
| **Whole World** |  |  |  |  |  |  |  |  |  |  |  |  |
| LCHB |  | 2.05 (1.78, 2.39) | 2.62 (2.15, 3.19) | 0.79 (-0.34, 1.88) |  | 3.31 (2.84, 3.85) | 4.27 (3.43, 5.31) | 0.82 (-0.37, 2.02) |  | 0.78 (0.62, 0.96) | 0.95 (0.76, 1.18) | 0.64 (-0.75, 2.08) |
| LCHC |  | 1.21 (1.04, 1.41) | 1.95 (1.67, 2.25) | 1.54 (0.55, 2.49) |  | 1.28 (1.11, 1.49) | 2.06 (1.75, 2.43) | 1.53 (0.52, 2.53) |  | 1.14 (0.97, 1.36) | 1.84 (1.56, 2.11) | 1.54 (0.44, 2.51) |
| LCAL |  | 0.72 (0.59, 0.87) | 1.26 (1.03, 1.53) | 1.81 (0.54, 3.07) |  | 1.1 (0.91, 1.34) | 1.97 (1.62, 2.38) | 1.88 (0.61, 3.1) |  | 0.34 (0.27, 0.42) | 0.55 (0.44, 0.67) | 1.55 (0.15, 2.93) |
| LCNA |  | 0.27 (0.22, 0.33) | 0.54 (0.43, 0.65) | 2.24 (0.85, 3.49) |  | 0.27 (0.21, 0.33) | 0.55 (0.43, 0.68) | 2.3 (0.85, 3.79) |  | 0.28 (0.22, 0.35) | 0.52 (0.43, 0.64) | 2 (0.66, 3.44) |
| LCOT |  | 0.2 (0.17, 0.25) | 0.29 (0.23, 0.35) | 1.2 (-0.27, 2.33) |  | 0.2 (0.16, 0.25) | 0.3 (0.23, 0.38) | 1.31 (-0.27, 2.79) |  | 0.21 (0.16, 0.26) | 0.28 (0.22, 0.34) | 0.93 (-0.54, 2.43) |

Abbreviation: UI, Uncertainty interval; EAPC, Estimated annual percentage change; CI, Confidence interval; LCHB, Liver cancer by HBC; LCHC, Liver cancer by HCV; LCAL, Liver cancer by alcoholic use; LCNA; Liver cancer by nonalcoholic steatohepatitis (NASH); LCOT, Liver cancer by other causes

Table S11. Age-standardized prevalence rates (per 100,000) for specific liver cancer in the Western Pacific region by member state and sex, in 1990 to 2021.

| **Region/Liver cancer cause** |  | **Both sexes combined** | | |  | **Male** | | |  | **Female** | | |
| --- | --- | --- | --- | --- | --- | --- | --- | --- | --- | --- | --- | --- |
|  |  | **1990 (95% UI)** | **2021 (95% UI)** | **EAPC, %**  **(95% CI)** |  | **1990 (95% UI)** | **2021 (95% UI)** | **EAPC, %**  **(95% CI)** |  | **1990 (95% UI)** | **2021 (95% UI)** | **EAPC, %**  **(95% CI)** |
| **American Samoa** |  |  |  |  |  |  |  |  |  |  |  |  |
| LCHB |  | 1.4 (1, 1.94) | 3.94 (2.79, 5.37) | 3.34 (1.17, 5.42) |  | 1.95 (1.37, 2.78) | 5.47 (3.79, 7.7) | 3.33 (1, 5.57) |  | 0.82 (0.54, 1.24) | 2.34 (1.57, 3.49) | 3.38 (0.76, 6.02) |
| LCHC |  | 0.37 (0.23, 0.58) | 1.24 (0.8, 1.86) | 3.9 (1.04, 6.74) |  | 0.32 (0.19, 0.54) | 1.01 (0.62, 1.56) | 3.71 (0.45, 6.79) |  | 0.43 (0.27, 0.67) | 1.47 (0.95, 2.23) | 3.97 (1.13, 6.81) |
| LCAL |  | 0.22 (0.13, 0.35) | 0.69 (0.43, 1.07) | 3.69 (0.66, 6.8) |  | 0.32 (0.19, 0.54) | 1 (0.61, 1.57) | 3.68 (0.39, 6.81) |  | 0.11 (0.07, 0.18) | 0.37 (0.22, 0.6) | 3.91 (0.65, 6.93) |
| LCNA |  | 0.27 (0.17, 0.4) | 0.96 (0.63, 1.41) | 4.09 (1.47, 6.82) |  | 0.23 (0.14, 0.37) | 0.81 (0.5, 1.26) | 4.06 (0.97, 7.09) |  | 0.31 (0.2, 0.47) | 1.11 (0.71, 1.68) | 4.11 (1.33, 6.87) |
| LCOT |  | 0.11 (0.07, 0.17) | 0.29 (0.18, 0.45) | 3.13 (0.18, 6) |  | 0.08 (0.05, 0.14) | 0.22 (0.14, 0.36) | 3.26 (0, 6.37) |  | 0.14 (0.08, 0.22) | 0.37 (0.22, 0.58) | 3.14 (0, 6.39) |
| **Australia** |  |  |  |  |  |  |  |  |  |  |  |  |
| LCHB |  | 0.46 (0.33, 0.63) | 2.03 (1.35, 2.86) | 4.79 (2.46, 6.97) |  | 0.77 (0.55, 1.06) | 3.51 (2.32, 5.04) | 4.89 (2.53, 7.15) |  | 0.16 (0.11, 0.22) | 0.59 (0.39, 0.84) | 4.21 (1.85, 6.56) |
| LCHC |  | 0.64 (0.47, 0.85) | 4.03 (2.99, 5.35) | 5.94 (4.06, 7.85) |  | 0.78 (0.54, 1.09) | 5.34 (3.74, 7.35) | 6.21 (3.98, 8.42) |  | 0.5 (0.39, 0.63) | 2.75 (2.1, 3.52) | 5.5 (3.88, 7.1) |
| LCAL |  | 1.06 (0.82, 1.29) | 5.99 (4.67, 7.49) | 5.59 (4.15, 7.14) |  | 1.85 (1.45, 2.27) | 10.8 (8.43, 13.53) | 5.69 (4.23, 7.2) |  | 0.28 (0.2, 0.37) | 1.3 (0.9, 1.8) | 4.95 (2.87, 7.09) |
| LCNA |  | 0.19 (0.13, 0.26) | 1.42 (0.97, 2) | 6.49 (4.25, 8.82) |  | 0.22 (0.15, 0.32) | 1.84 (1.22, 2.65) | 6.85 (4.32, 9.26) |  | 0.16 (0.11, 0.21) | 1.01 (0.68, 1.42) | 5.94 (3.79, 8.25) |
| LCOT |  | 0.11 (0.08, 0.15) | 0.59 (0.41, 0.81) | 5.42 (3.24, 7.47) |  | 0.12 (0.08, 0.17) | 0.73 (0.48, 1.05) | 5.82 (3.35, 8.3) |  | 0.1 (0.07, 0.14) | 0.46 (0.31, 0.64) | 4.92 (2.56, 7.14) |
| **Brunei Darussalam** |  |  |  |  |  |  |  |  |  |  |  |  |
| LCHB |  | 3.83 (2.72, 5.35) | 4.94 (3.51, 6.81) | 0.82 (-1.36, 2.96) |  | 5.8 (4.12, 8.06) | 7.46 (5.4, 10.16) | 0.81 (-1.29, 2.91) |  | 1.61 (1.1, 2.35) | 2.12 (1.37, 3.26) | 0.89 (-1.74, 3.5) |
| LCHC |  | 1.7 (1.02, 2.55) | 2.79 (1.81, 3.96) | 1.6 (-1.11, 4.38) |  | 1.75 (0.96, 2.69) | 2.75 (1.7, 4.18) | 1.46 (-1.48, 4.75) |  | 1.64 (0.98, 2.49) | 2.83 (1.77, 4.28) | 1.76 (-1.1, 4.76) |
| LCAL |  | 0.51 (0.3, 0.83) | 0.74 (0.47, 1.16) | 1.2 (-1.83, 4.36) |  | 0.79 (0.47, 1.28) | 1.13 (0.71, 1.77) | 1.15 (-1.9, 4.28) |  | 0.2 (0.11, 0.33) | 0.32 (0.19, 0.53) | 1.52 (-1.78, 5.07) |
| LCNA |  | 0.32 (0.19, 0.51) | 0.55 (0.35, 0.85) | 1.75 (-1.21, 4.83) |  | 0.32 (0.18, 0.53) | 0.52 (0.32, 0.81) | 1.57 (-1.63, 4.85) |  | 0.32 (0.19, 0.54) | 0.59 (0.36, 0.95) | 1.97 (-1.31, 5.19) |
| LCOT |  | 0.24 (0.15, 0.38) | 0.37 (0.23, 0.59) | 1.4 (-1.62, 4.42) |  | 0.24 (0.14, 0.39) | 0.35 (0.21, 0.58) | 1.22 (-2, 4.59) |  | 0.24 (0.15, 0.38) | 0.38 (0.23, 0.64) | 1.48 (-1.62, 4.68) |
| **Cambodia** |  |  |  |  |  |  |  |  |  |  |  |  |
| LCHB |  | 2.19 (1.13, 4.19) | 2.2 (1.07, 4.31) | 0.01 (-4.4, 4.32) |  | 2.68 (1.33, 6.19) | 2.73 (1.09, 6.71) | 0.06 (-5.6, 5.22) |  | 1.75 (0.74, 3.46) | 1.68 (0.77, 2.89) | -0.13 (-4.85, 4.39) |
| LCHC |  | 1 (0.48, 1.91) | 1.26 (0.65, 2.27) | 0.75 (-3.48, 5.01) |  | 0.76 (0.34, 1.86) | 0.88 (0.35, 2.3) | 0.47 (-5.39, 6.17) |  | 1.21 (0.47, 2.48) | 1.62 (0.77, 2.83) | 0.94 (-3.77, 5.79) |
| LCAL |  | 0.59 (0.27, 1.26) | 1.29 (0.61, 2.65) | 2.52 (-2.34, 7.37) |  | 0.85 (0.36, 1.97) | 1.79 (0.71, 4.4) | 2.4 (-3.29, 8.08) |  | 0.36 (0.14, 0.81) | 0.81 (0.36, 1.58) | 2.62 (-2.62, 7.82) |
| LCNA |  | 0.32 (0.15, 0.63) | 0.46 (0.23, 0.83) | 1.17 (-3.25, 5.52) |  | 0.23 (0.1, 0.53) | 0.32 (0.13, 0.76) | 1.07 (-4.53, 6.54) |  | 0.41 (0.16, 0.86) | 0.59 (0.28, 1.06) | 1.17 (-3.62, 6.1) |
| LCOT |  | 0.16 (0.08, 0.31) | 0.18 (0.09, 0.34) | 0.38 (-3.99, 4.67) |  | 0.11 (0.05, 0.27) | 0.12 (0.05, 0.31) | 0.28 (-5.44, 5.89) |  | 0.21 (0.08, 0.42) | 0.22 (0.1, 0.41) | 0.15 (-4.63, 5.27) |
| **China** |  |  |  |  |  |  |  |  |  |  |  |  |
| LCHB |  | 6.81 (5.6, 8.1) | 11.61 (9.07, 15.12) | 1.72 (0.36, 3.2) |  | 11.22 (9.01, 13.72) | 19.64 (14.75, 26.53) | 1.81 (0.23, 3.48) |  | 2.12 (1.66, 2.61) | 3.2 (2.34, 4.23) | 1.33 (-0.35, 3.02) |
| LCHC |  | 1.27 (1.01, 1.54) | 3 (2.33, 3.72) | 2.77 (1.34, 4.21) |  | 1.09 (0.83, 1.42) | 2.75 (2, 3.79) | 2.99 (1.1, 4.9) |  | 1.45 (1.12, 1.8) | 3.25 (2.48, 4.21) | 2.6 (1.03, 4.27) |
| LCAL |  | 0.72 (0.55, 0.93) | 1.84 (1.36, 2.46) | 3.03 (1.23, 4.83) |  | 0.93 (0.7, 1.24) | 2.49 (1.69, 3.55) | 3.18 (1, 5.24) |  | 0.5 (0.37, 0.65) | 1.17 (0.85, 1.55) | 2.74 (0.87, 4.62) |
| LCNA |  | 0.39 (0.32, 0.48) | 0.99 (0.75, 1.26) | 3.01 (1.44, 4.42) |  | 0.41 (0.32, 0.53) | 1.08 (0.76, 1.49) | 3.12 (1.16, 4.96) |  | 0.38 (0.29, 0.47) | 0.89 (0.67, 1.2) | 2.75 (1.14, 4.58) |
| LCOT |  | 0.55 (0.43, 0.68) | 0.88 (0.68, 1.15) | 1.52 (0, 3.17) |  | 0.55 (0.43, 0.7) | 0.97 (0.69, 1.39) | 1.83 (-0.05, 3.78) |  | 0.54 (0.41, 0.69) | 0.79 (0.58, 1.05) | 1.23 (-0.56, 3.03) |
| **Cook Islands** |  |  |  |  |  |  |  |  |  |  |  |  |
| LCHB |  | 4.28 (3.07, 5.85) | 7.92 (5.41, 11.38) | 1.99 (-0.25, 4.23) |  | 6.05 (4.29, 8.57) | 13.05 (8.68, 18.92) | 2.48 (0.04, 4.79) |  | 2.37 (1.59, 3.33) | 3.08 (2.02, 4.53) | 0.85 (-1.61, 3.38) |
| LCHC |  | 1.34 (0.86, 2.02) | 3.07 (2, 4.53) | 2.67 (-0.03, 5.36) |  | 1.19 (0.69, 1.89) | 3.28 (2.06, 4.99) | 3.27 (0.28, 6.38) |  | 1.5 (0.99, 2.25) | 2.88 (1.86, 4.29) | 2.1 (-0.61, 4.73) |
| LCAL |  | 0.8 (0.49, 1.25) | 4.24 (2.71, 6.63) | 5.38 (2.5, 8.4) |  | 1.18 (0.72, 1.89) | 7.12 (4.52, 11.14) | 5.8 (2.81, 8.84) |  | 0.38 (0.23, 0.61) | 1.52 (0.92, 2.34) | 4.47 (1.33, 7.48) |
| LCNA |  | 0.75 (0.49, 1.12) | 2.19 (1.38, 3.29) | 3.46 (0.67, 6.14) |  | 0.68 (0.42, 1.08) | 2.47 (1.48, 3.75) | 4.16 (1.02, 7.06) |  | 0.83 (0.54, 1.23) | 1.93 (1.17, 2.93) | 2.72 (-0.16, 5.46) |
| LCOT |  | 0.33 (0.21, 0.49) | 0.57 (0.35, 0.88) | 1.76 (-1.09, 4.62) |  | 0.26 (0.16, 0.42) | 0.6 (0.35, 0.96) | 2.7 (-0.59, 5.78) |  | 0.4 (0.26, 0.62) | 0.54 (0.32, 0.84) | 0.97 (-2.13, 3.78) |
| **Federated States of Micronesia** |  |  |  |  |  |  |  |  |  |  |  |  |
| LCHB |  | 1.65 (1.06, 2.59) | 2.58 (1.47, 4.42) | 1.44 (-1.83, 4.61) |  | 2.32 (1.33, 4) | 3.78 (2.03, 7.23) | 1.57 (-2.19, 5.46) |  | 0.95 (0.59, 1.43) | 1.34 (0.62, 2.19) | 1.11 (-2.7, 4.23) |
| LCHC |  | 0.46 (0.29, 0.75) | 0.78 (0.43, 1.34) | 1.7 (-1.79, 4.94) |  | 0.39 (0.2, 0.77) | 0.69 (0.33, 1.5) | 1.84 (-2.73, 6.5) |  | 0.53 (0.32, 0.83) | 0.87 (0.39, 1.41) | 1.6 (-2.44, 4.78) |
| LCAL |  | 0.31 (0.17, 0.52) | 0.51 (0.27, 0.98) | 1.61 (-2.11, 5.65) |  | 0.45 (0.23, 0.79) | 0.76 (0.37, 1.57) | 1.69 (-2.45, 6.2) |  | 0.16 (0.09, 0.25) | 0.25 (0.12, 0.44) | 1.44 (-2.37, 5.12) |
| LCNA |  | 0.23 (0.15, 0.37) | 0.46 (0.26, 0.74) | 2.24 (-1.14, 5.15) |  | 0.2 (0.1, 0.36) | 0.43 (0.2, 0.87) | 2.47 (-1.9, 6.98) |  | 0.27 (0.16, 0.42) | 0.5 (0.24, 0.82) | 1.99 (-1.81, 5.27) |
| LCOT |  | 0.13 (0.08, 0.21) | 0.2 (0.11, 0.34) | 1.39 (-2.09, 4.67) |  | 0.1 (0.05, 0.19) | 0.17 (0.08, 0.36) | 1.71 (-2.79, 6.37) |  | 0.16 (0.1, 0.26) | 0.24 (0.11, 0.41) | 1.31 (-2.77, 4.55) |
| **Fiji** |  |  |  |  |  |  |  |  |  |  |  |  |
| LCHB |  | 1.54 (1.07, 2.25) | 2.46 (1.58, 3.55) | 1.51 (-1.14, 3.87) |  | 2.17 (1.46, 3.23) | 3.62 (2.26, 5.55) | 1.65 (-1.15, 4.31) |  | 0.89 (0.54, 1.48) | 1.27 (0.8, 1.87) | 1.15 (-1.98, 4.01) |
| LCHC |  | 0.4 (0.23, 0.69) | 0.84 (0.53, 1.28) | 2.39 (-0.85, 5.54) |  | 0.36 (0.2, 0.6) | 0.77 (0.43, 1.29) | 2.45 (-1.07, 6.01) |  | 0.44 (0.24, 0.8) | 0.91 (0.6, 1.34) | 2.34 (-0.93, 5.55) |
| LCAL |  | 0.24 (0.14, 0.39) | 0.53 (0.3, 0.86) | 2.56 (-0.85, 5.86) |  | 0.35 (0.2, 0.59) | 0.8 (0.44, 1.3) | 2.67 (-0.95, 6.04) |  | 0.12 (0.06, 0.22) | 0.24 (0.15, 0.39) | 2.24 (-1.24, 6.04) |
| LCNA |  | 0.2 (0.12, 0.33) | 0.5 (0.3, 0.77) | 2.96 (-0.31, 6) |  | 0.18 (0.11, 0.3) | 0.47 (0.26, 0.78) | 3.1 (-0.46, 6.32) |  | 0.23 (0.13, 0.41) | 0.52 (0.33, 0.8) | 2.63 (-0.7, 5.86) |
| LCOT |  | 0.12 (0.07, 0.2) | 0.2 (0.12, 0.31) | 1.65 (-1.65, 4.8) |  | 0.09 (0.05, 0.17) | 0.17 (0.09, 0.3) | 2.05 (-2.05, 5.78) |  | 0.15 (0.08, 0.26) | 0.23 (0.14, 0.35) | 1.38 (-2, 4.76) |
| **Guam** |  |  |  |  |  |  |  |  |  |  |  |  |
| LCHB |  | 1.34 (1.08, 1.65) | 4.95 (3.81, 6.16) | 4.22 (2.7, 5.62) |  | 2.03 (1.63, 2.46) | 8.51 (6.53, 10.63) | 4.62 (3.15, 6.05) |  | 0.55 (0.41, 0.7) | 1.3 (0.91, 1.75) | 2.77 (0.85, 4.68) |
| LCHC |  | 0.34 (0.22, 0.48) | 1.3 (0.84, 1.86) | 4.33 (1.81, 6.89) |  | 0.35 (0.21, 0.51) | 1.63 (1.02, 2.4) | 4.96 (2.24, 7.86) |  | 0.33 (0.22, 0.47) | 0.96 (0.61, 1.33) | 3.44 (0.84, 5.8) |
| LCAL |  | 0.25 (0.16, 0.36) | 1.15 (0.75, 1.76) | 4.92 (2.37, 7.74) |  | 0.39 (0.25, 0.56) | 1.99 (1.27, 3.04) | 5.26 (2.64, 8.06) |  | 0.09 (0.06, 0.14) | 0.29 (0.18, 0.44) | 3.77 (0.81, 6.43) |
| LCNA |  | 0.2 (0.14, 0.28) | 0.89 (0.6, 1.3) | 4.82 (2.46, 7.19) |  | 0.21 (0.14, 0.31) | 1.17 (0.78, 1.74) | 5.54 (2.98, 8.13) |  | 0.18 (0.13, 0.25) | 0.61 (0.4, 0.89) | 3.94 (1.52, 6.21) |
| LCOT |  | 0.09 (0.06, 0.13) | 0.27 (0.18, 0.41) | 3.54 (1.05, 6.2) |  | 0.09 (0.06, 0.13) | 0.35 (0.21, 0.53) | 4.38 (1.55, 7.03) |  | 0.09 (0.06, 0.12) | 0.2 (0.13, 0.29) | 2.58 (0.26, 5.08) |
| **Japan** |  |  |  |  |  |  |  |  |  |  |  |  |
| LCHB |  | 4.96 (4.22, 5.77) | 6.29 (5.03, 7.66) | 0.77 (-0.44, 1.92) |  | 8.1 (6.89, 9.43) | 9.61 (7.82, 11.83) | 0.55 (-0.6, 1.74) |  | 1.93 (1.61, 2.26) | 3.13 (2.29, 3.93) | 1.56 (0.04, 2.88) |
| LCHC |  | 19.9 (18.53, 21.18) | 40.08 (33.36, 44.85) | 2.26 (1.47, 2.85) |  | 29.4 (27.31, 31.45) | 55.66 (48.96, 61.79) | 2.06 (1.43, 2.63) |  | 10.71 (9.74, 11.51) | 25.29 (18.44, 30.39) | 2.77 (1.52, 3.67) |
| LCAL |  | 4.18 (3.59, 4.95) | 6.31 (5.28, 7.67) | 1.33 (0.21, 2.45) |  | 7.25 (6.22, 8.57) | 10.72 (8.98, 12.96) | 1.26 (0.15, 2.37) |  | 1.22 (1.03, 1.47) | 2.12 (1.57, 2.76) | 1.78 (0.21, 3.18) |
| LCNA |  | 1.27 (1.08, 1.48) | 2.6 (1.97, 3.21) | 2.31 (0.92, 3.51) |  | 1.5 (1.25, 1.76) | 2.79 (2.23, 3.4) | 2 (0.76, 3.23) |  | 1.05 (0.87, 1.23) | 2.42 (1.7, 3.18) | 2.69 (1.04, 4.18) |
| LCOT |  | 0.92 (0.78, 1.08) | 1.4 (1.1, 1.72) | 1.35 (0.06, 2.55) |  | 1.13 (0.94, 1.34) | 1.54 (1.26, 1.84) | 1 (-0.2, 2.17) |  | 0.71 (0.6, 0.84) | 1.26 (0.9, 1.6) | 1.85 (0.22, 3.16) |
| **Kiribati** |  |  |  |  |  |  |  |  |  |  |  |  |
| LCHB |  | 2.43 (1.74, 3.34) | 2.55 (1.72, 3.79) | 0.16 (-2.14, 2.51) |  | 2.82 (1.94, 3.84) | 2.88 (1.8, 4.7) | 0.07 (-2.44, 2.85) |  | 2.04 (1.4, 2.92) | 2.23 (1.44, 3.42) | 0.29 (-2.28, 2.88) |
| LCHC |  | 0.74 (0.5, 1.06) | 0.91 (0.53, 1.39) | 0.67 (-2.24, 3.3) |  | 0.44 (0.26, 0.7) | 0.47 (0.25, 0.84) | 0.21 (-3.32, 3.78) |  | 1.03 (0.68, 1.53) | 1.32 (0.75, 1.96) | 0.8 (-2.3, 3.41) |
| LCAL |  | 0.33 (0.21, 0.53) | 0.38 (0.22, 0.63) | 0.46 (-2.84, 3.54) |  | 0.42 (0.26, 0.68) | 0.44 (0.24, 0.79) | 0.15 (-3.36, 3.59) |  | 0.25 (0.15, 0.39) | 0.31 (0.18, 0.51) | 0.69 (-2.49, 3.95) |
| LCNA |  | 0.37 (0.24, 0.56) | 0.47 (0.3, 0.68) | 0.77 (-2.01, 3.36) |  | 0.22 (0.14, 0.35) | 0.26 (0.15, 0.45) | 0.54 (-2.73, 3.77) |  | 0.51 (0.33, 0.77) | 0.68 (0.41, 1.01) | 0.93 (-2.03, 3.61) |
| LCOT |  | 0.23 (0.15, 0.35) | 0.27 (0.17, 0.41) | 0.52 (-2.33, 3.24) |  | 0.12 (0.07, 0.18) | 0.13 (0.07, 0.24) | 0.26 (-3.05, 3.97) |  | 0.34 (0.22, 0.51) | 0.4 (0.24, 0.64) | 0.52 (-2.43, 3.44) |
| **Laos** |  |  |  |  |  |  |  |  |  |  |  |  |
| LCHB |  | 2.97 (1.8, 4.33) | 2.31 (1.46, 3.44) | -0.81 (-3.51, 2.09) |  | 4.74 (2.8, 6.95) | 3.58 (2.18, 5.43) | -0.91 (-3.74, 2.14) |  | 1.27 (0.81, 1.87) | 1.03 (0.61, 1.52) | -0.68 (-3.61, 2.03) |
| LCHC |  | 1.07 (0.63, 1.66) | 0.89 (0.55, 1.41) | -0.59 (-3.56, 2.6) |  | 1.17 (0.64, 1.93) | 0.93 (0.51, 1.53) | -0.74 (-4.29, 2.81) |  | 0.97 (0.58, 1.47) | 0.86 (0.54, 1.27) | -0.39 (-3.23, 2.53) |
| LCAL |  | 1.16 (0.67, 1.83) | 1.24 (0.72, 1.89) | 0.22 (-3.01, 3.35) |  | 1.96 (1.1, 3.18) | 2.01 (1.15, 3.12) | 0.08 (-3.28, 3.36) |  | 0.39 (0.24, 0.61) | 0.45 (0.26, 0.7) | 0.46 (-2.75, 3.45) |
| LCNA |  | 0.38 (0.23, 0.61) | 0.37 (0.22, 0.58) | -0.09 (-3.29, 2.98) |  | 0.42 (0.22, 0.71) | 0.39 (0.21, 0.64) | -0.24 (-3.93, 3.44) |  | 0.35 (0.22, 0.56) | 0.35 (0.2, 0.54) | 0 (-3.32, 2.9) |
| LCOT |  | 0.18 (0.11, 0.27) | 0.15 (0.09, 0.24) | -0.59 (-3.54, 2.52) |  | 0.2 (0.11, 0.32) | 0.15 (0.08, 0.25) | -0.93 (-4.47, 2.65) |  | 0.17 (0.1, 0.25) | 0.15 (0.08, 0.23) | -0.4 (-3.68, 2.69) |
| **Malaysia** |  |  |  |  |  |  |  |  |  |  |  |  |
| LCHB |  | 1.55 (1.16, 2.01) | 3.39 (2.44, 4.53) | 2.52 (0.63, 4.39) |  | 2.45 (1.8, 3.24) | 5.53 (3.95, 7.43) | 2.63 (0.64, 4.57) |  | 0.64 (0.47, 0.85) | 1.1 (0.74, 1.51) | 1.75 (-0.45, 3.76) |
| LCHC |  | 0.37 (0.25, 0.54) | 0.92 (0.6, 1.33) | 2.94 (0.34, 5.39) |  | 0.34 (0.21, 0.51) | 0.96 (0.59, 1.45) | 3.35 (0.47, 6.23) |  | 0.4 (0.27, 0.58) | 0.87 (0.6, 1.24) | 2.51 (0.11, 4.92) |
| LCAL |  | 0.31 (0.21, 0.46) | 0.79 (0.5, 1.25) | 3.02 (0.27, 5.75) |  | 0.49 (0.31, 0.73) | 1.26 (0.78, 2.02) | 3.05 (0.21, 6.05) |  | 0.14 (0.09, 0.22) | 0.29 (0.17, 0.45) | 2.35 (-0.83, 5.19) |
| LCNA |  | 0.2 (0.14, 0.3) | 0.61 (0.4, 0.89) | 3.6 (0.93, 5.97) |  | 0.19 (0.13, 0.29) | 0.67 (0.42, 1.04) | 4.07 (1.19, 6.71) |  | 0.21 (0.14, 0.32) | 0.55 (0.36, 0.81) | 3.11 (0.38, 5.66) |
| LCOT |  | 0.08 (0.05, 0.12) | 0.17 (0.11, 0.25) | 2.43 (-0.28, 5.19) |  | 0.08 (0.05, 0.11) | 0.18 (0.11, 0.27) | 2.62 (0, 5.44) |  | 0.08 (0.06, 0.12) | 0.16 (0.1, 0.24) | 2.24 (-0.59, 4.47) |
| **Marshall Islands** |  |  |  |  |  |  |  |  |  |  |  |  |
| LCHB |  | 0.86 (0.51, 1.55) | 1.78 (0.97, 3.3) | 2.35 (-1.51, 6.02) |  | 1.06 (0.58, 2.04) | 2.36 (1.15, 5.08) | 2.58 (-1.85, 7) |  | 0.65 (0.38, 1.18) | 1.17 (0.69, 1.87) | 1.9 (-1.73, 5.14) |
| LCHC |  | 0.25 (0.13, 0.44) | 0.52 (0.28, 0.94) | 2.36 (-1.46, 6.38) |  | 0.17 (0.08, 0.36) | 0.4 (0.17, 0.98) | 2.76 (-2.42, 8.08) |  | 0.33 (0.17, 0.62) | 0.64 (0.35, 1.12) | 2.14 (-1.84, 6.08) |
| LCAL |  | 0.14 (0.07, 0.27) | 0.34 (0.17, 0.66) | 2.86 (-1.49, 7.24) |  | 0.18 (0.09, 0.39) | 0.47 (0.21, 1.06) | 3.1 (-2, 7.96) |  | 0.09 (0.05, 0.18) | 0.2 (0.12, 0.35) | 2.58 (-1.31, 6.28) |
| LCNA |  | 0.11 (0.06, 0.2) | 0.28 (0.16, 0.44) | 3.01 (-0.72, 6.43) |  | 0.08 (0.04, 0.16) | 0.21 (0.1, 0.47) | 3.11 (-1.52, 7.95) |  | 0.15 (0.08, 0.29) | 0.34 (0.2, 0.55) | 2.64 (-1.2, 6.22) |
| LCOT |  | 0.08 (0.04, 0.14) | 0.16 (0.09, 0.26) | 2.24 (-1.43, 6.04) |  | 0.05 (0.02, 0.1) | 0.11 (0.05, 0.23) | 2.54 (-2.24, 7.88) |  | 0.11 (0.06, 0.23) | 0.21 (0.12, 0.37) | 2.09 (-2.1, 5.87) |
| **Mongolia** |  |  |  |  |  |  |  |  |  |  |  |  |
| LCHB |  | 11.24 (7.39, 16.89) | 15.98 (10.4, 23.75) | 1.14 (-1.56, 3.77) |  | 16.18 (10.37, 24.43) | 23.04 (14.78, 34.25) | 1.14 (-1.62, 3.85) |  | 6.31 (3.78, 9.94) | 9.1 (5.8, 13.72) | 1.18 (-1.74, 4.16) |
| LCHC |  | 11.43 (7.21, 17.58) | 21.25 (14.78, 29.4) | 2 (-0.56, 4.53) |  | 12.34 (7.37, 19.7) | 18.73 (12.34, 27.72) | 1.35 (-1.51, 4.27) |  | 10.52 (6.56, 16.11) | 23.71 (16.97, 32.1) | 2.62 (0.17, 5.12) |
| LCAL |  | 5.38 (3.28, 8.81) | 12.78 (8.49, 19.54) | 2.79 (-0.12, 5.76) |  | 8.49 (5.06, 13.86) | 18.69 (12.18, 28.73) | 2.55 (-0.42, 5.6) |  | 2.28 (1.33, 3.81) | 7.01 (4.59, 11.13) | 3.62 (0.6, 6.85) |
| LCNA |  | 1.61 (1, 2.5) | 3.46 (2.18, 5.37) | 2.47 (-0.44, 5.42) |  | 1.5 (0.88, 2.48) | 2.76 (1.62, 4.32) | 1.97 (-1.37, 5.13) |  | 1.72 (1.06, 2.72) | 4.15 (2.64, 6.35) | 2.84 (-0.1, 5.77) |
| LCOT |  | 1.09 (0.67, 1.77) | 1.67 (1.05, 2.58) | 1.38 (-1.68, 4.35) |  | 0.9 (0.51, 1.5) | 1.28 (0.77, 2.11) | 1.14 (-2.15, 4.58) |  | 1.27 (0.74, 2.07) | 2.05 (1.3, 3.06) | 1.54 (-1.5, 4.58) |
| **Nauru** |  |  |  |  |  |  |  |  |  |  |  |  |
| LCHB |  | 2.84 (1.94, 3.88) | 2.66 (1.54, 4.25) | -0.21 (-2.98, 2.53) |  | 4.41 (2.96, 6.19) | 4.15 (2.25, 6.91) | -0.2 (-3.26, 2.73) |  | 1.16 (0.77, 1.7) | 1.15 (0.62, 1.85) | -0.03 (-3.25, 2.83) |
| LCHC |  | 0.53 (0.33, 0.82) | 0.58 (0.32, 0.95) | 0.29 (-3.04, 3.41) |  | 0.57 (0.34, 0.91) | 0.57 (0.28, 1.06) | 0 (-3.8, 3.67) |  | 0.49 (0.31, 0.77) | 0.58 (0.3, 1.02) | 0.54 (-3.04, 3.84) |
| LCAL |  | 0.47 (0.28, 0.73) | 0.53 (0.29, 0.92) | 0.39 (-2.98, 3.84) |  | 0.74 (0.43, 1.2) | 0.83 (0.43, 1.51) | 0.37 (-3.31, 4.05) |  | 0.17 (0.1, 0.25) | 0.22 (0.12, 0.37) | 0.83 (-2.37, 4.22) |
| LCNA |  | 0.32 (0.2, 0.48) | 0.38 (0.22, 0.59) | 0.55 (-2.52, 3.49) |  | 0.35 (0.21, 0.56) | 0.39 (0.2, 0.68) | 0.35 (-3.32, 3.79) |  | 0.29 (0.19, 0.45) | 0.38 (0.21, 0.61) | 0.87 (-2.46, 3.76) |
| LCOT |  | 0.19 (0.12, 0.28) | 0.21 (0.11, 0.32) | 0.32 (-3.01, 3.16) |  | 0.19 (0.11, 0.3) | 0.19 (0.09, 0.35) | 0 (-3.88, 3.73) |  | 0.19 (0.12, 0.29) | 0.22 (0.11, 0.38) | 0.47 (-3.13, 3.72) |
| **New Zealand** |  |  |  |  |  |  |  |  |  |  |  |  |
| LCHB |  | 0.72 (0.6, 0.86) | 2.26 (1.84, 2.79) | 3.69 (2.45, 4.96) |  | 1.14 (0.93, 1.38) | 3.64 (2.91, 4.57) | 3.75 (2.41, 5.14) |  | 0.31 (0.26, 0.37) | 0.89 (0.71, 1.07) | 3.4 (2.1, 4.56) |
| LCHC |  | 0.91 (0.77, 1.06) | 4.2 (3.52, 4.86) | 4.93 (3.87, 5.94) |  | 0.96 (0.78, 1.17) | 4.43 (3.62, 5.35) | 4.93 (3.64, 6.21) |  | 0.86 (0.72, 1) | 3.98 (3.2, 4.78) | 4.94 (3.75, 6.11) |
| LCAL |  | 1.56 (1.33, 1.77) | 5.71 (4.84, 6.65) | 4.19 (3.24, 5.19) |  | 2.6 (2.2, 3.01) | 9.5 (7.81, 11.22) | 4.18 (3.08, 5.26) |  | 0.54 (0.45, 0.64) | 1.93 (1.59, 2.34) | 4.11 (2.94, 5.32) |
| LCNA |  | 0.29 (0.25, 0.34) | 1.6 (1.32, 1.88) | 5.51 (4.38, 6.51) |  | 0.29 (0.24, 0.35) | 1.65 (1.33, 2.01) | 5.61 (4.31, 6.86) |  | 0.29 (0.24, 0.35) | 1.55 (1.22, 1.88) | 5.41 (4.03, 6.64) |
| LCOT |  | 0.19 (0.16, 0.22) | 0.71 (0.6, 0.83) | 4.25 (3.24, 5.31) |  | 0.19 (0.15, 0.23) | 0.73 (0.59, 0.89) | 4.34 (3.04, 5.74) |  | 0.2 (0.17, 0.23) | 0.7 (0.57, 0.83) | 4.04 (2.93, 5.11) |
| **Niue** |  |  |  |  |  |  |  |  |  |  |  |  |
| LCHB |  | 2.24 (1.43, 3.4) | 3.56 (2.14, 5.84) | 1.49 (-1.49, 4.54) |  | 3.34 (2.12, 5.13) | 5.78 (3.36, 10.11) | 1.77 (-1.37, 5.04) |  | 1.13 (0.68, 1.84) | 1.32 (0.76, 2.08) | 0.5 (-2.85, 3.61) |
| LCHC |  | 0.82 (0.49, 1.3) | 1.3 (0.78, 2.06) | 1.49 (-1.65, 4.63) |  | 0.7 (0.4, 1.2) | 1.39 (0.74, 2.55) | 2.21 (-1.56, 5.98) |  | 0.94 (0.56, 1.55) | 1.22 (0.69, 1.91) | 0.84 (-2.61, 3.96) |
| LCAL |  | 0.51 (0.29, 0.81) | 1.05 (0.6, 1.9) | 2.33 (-0.97, 6.06) |  | 0.77 (0.43, 1.25) | 1.73 (0.95, 3.26) | 2.61 (-0.89, 6.53) |  | 0.24 (0.14, 0.42) | 0.37 (0.2, 0.61) | 1.4 (-2.39, 4.75) |
| LCNA |  | 0.39 (0.24, 0.62) | 0.82 (0.48, 1.33) | 2.4 (-0.83, 5.52) |  | 0.36 (0.2, 0.6) | 0.92 (0.49, 1.67) | 3.03 (-0.65, 6.85) |  | 0.42 (0.25, 0.69) | 0.72 (0.4, 1.11) | 1.74 (-1.76, 4.81) |
| LCOT |  | 0.17 (0.1, 0.28) | 0.25 (0.14, 0.41) | 1.24 (-2.24, 4.55) |  | 0.14 (0.08, 0.25) | 0.27 (0.13, 0.51) | 2.12 (-2.11, 5.98) |  | 0.19 (0.11, 0.32) | 0.23 (0.13, 0.39) | 0.62 (-2.91, 4.08) |
| **Northern Mariana Islands** |  |  |  |  |  |  |  |  |  |  |  |  |
| LCHB |  | 1.85 (1.25, 2.66) | 4.83 (3.6, 6.42) | 3.1 (0.98, 5.28) |  | 2.59 (1.69, 3.83) | 7.28 (5.42, 9.85) | 3.33 (1.12, 5.69) |  | 1.04 (0.67, 1.5) | 2.12 (1.38, 3.02) | 2.3 (-0.27, 4.86) |
| LCHC |  | 0.38 (0.24, 0.58) | 1.47 (0.9, 2.16) | 4.36 (1.42, 7.09) |  | 0.34 (0.2, 0.56) | 1.49 (0.86, 2.34) | 4.77 (1.38, 7.93) |  | 0.42 (0.27, 0.63) | 1.44 (0.93, 2.07) | 3.97 (1.26, 6.57) |
| LCAL |  | 0.27 (0.16, 0.42) | 1.11 (0.69, 1.82) | 4.56 (1.6, 7.84) |  | 0.4 (0.23, 0.63) | 1.73 (1.03, 2.95) | 4.72 (1.59, 8.23) |  | 0.13 (0.08, 0.21) | 0.43 (0.26, 0.69) | 3.86 (0.69, 6.95) |
| LCNA |  | 0.3 (0.2, 0.44) | 1.07 (0.67, 1.58) | 4.1 (1.36, 6.67) |  | 0.26 (0.16, 0.43) | 1.12 (0.67, 1.75) | 4.71 (1.43, 7.72) |  | 0.33 (0.21, 0.5) | 1.01 (0.62, 1.51) | 3.61 (0.69, 6.36) |
| LCOT |  | 0.13 (0.08, 0.21) | 0.3 (0.18, 0.46) | 2.7 (-0.5, 5.64) |  | 0.1 (0.06, 0.17) | 0.29 (0.17, 0.47) | 3.43 (0, 6.64) |  | 0.16 (0.1, 0.26) | 0.31 (0.18, 0.51) | 2.13 (-1.19, 5.26) |
| **Palau** |  |  |  |  |  |  |  |  |  |  |  |  |
| LCHB |  | 3.79 (2.4, 5.92) | 7.66 (5.16, 10.82) | 2.27 (-0.44, 4.86) |  | 7.35 (4.66, 11.47) | 13.91 (9.37, 19.65) | 2.06 (-0.65, 4.64) |  | 0 (0, 0.01) | 0.01 (0, 0.01) | - |
| LCHC |  | 0.64 (0.36, 1.11) | 1.42 (0.8, 2.29) | 2.57 (-1.06, 5.97) |  | 1.24 (0.69, 2.15) | 2.57 (1.46, 4.16) | 2.35 (-1.25, 5.8) |  | 0 (0, 0) | 0.01 (0, 0.01) | - |
| LCAL |  | 0.71 (0.42, 1.19) | 1.76 (1.03, 2.77) | 2.93 (-0.47, 6.08) |  | 1.38 (0.81, 2.31) | 3.2 (1.87, 5.02) | 2.71 (-0.68, 5.88) |  | 0 (0, 0) | 0 (0, 0) | - |
| LCNA |  | 0.38 (0.22, 0.64) | 1.05 (0.58, 1.68) | 3.28 (-0.32, 6.56) |  | 0.73 (0.43, 1.23) | 1.91 (1.04, 3.04) | 3.1 (-0.54, 6.31) |  | 0 (0, 0) | 0 (0, 0.01) | - |
| LCOT |  | 0.16 (0.09, 0.29) | 0.32 (0.18, 0.51) | 2.24 (-1.54, 5.6) |  | 0.31 (0.18, 0.56) | 0.59 (0.33, 0.93) | 2.08 (-1.71, 5.3) |  | 0 (0, 0) | 0 (0, 0) | - |
| **Papua New Guinea** |  |  |  |  |  |  |  |  |  |  |  |  |
| LCHB |  | 1.25 (0.58, 3.07) | 0.93 (0.43, 2.45) | -0.95 (-6.34, 4.65) |  | 1.76 (0.83, 4.78) | 1.31 (0.56, 3.96) | -0.95 (-6.92, 5.04) |  | 0.71 (0.29, 1.58) | 0.52 (0.25, 1.08) | -1 (-5.95, 4.24) |
| LCHC |  | 0.33 (0.13, 0.84) | 0.26 (0.11, 0.61) | -0.77 (-6.56, 4.99) |  | 0.3 (0.13, 0.87) | 0.23 (0.09, 0.69) | -0.86 (-7.32, 5.38) |  | 0.36 (0.14, 0.9) | 0.28 (0.12, 0.63) | -0.81 (-6.5, 4.85) |
| LCAL |  | 0.21 (0.09, 0.62) | 0.17 (0.07, 0.48) | -0.68 (-7.04, 5.4) |  | 0.32 (0.14, 0.96) | 0.25 (0.09, 0.78) | -0.8 (-7.64, 5.54) |  | 0.1 (0.04, 0.25) | 0.08 (0.03, 0.18) | -0.72 (-6.84, 4.85) |
| LCNA |  | 0.14 (0.06, 0.34) | 0.12 (0.05, 0.28) | -0.5 (-6.18, 4.97) |  | 0.12 (0.05, 0.35) | 0.1 (0.04, 0.32) | -0.59 (-7, 5.99) |  | 0.15 (0.06, 0.37) | 0.13 (0.06, 0.29) | -0.46 (-5.87, 5.08) |
| LCOT |  | 0.1 (0.04, 0.26) | 0.08 (0.03, 0.17) | -0.72 (-6.97, 4.67) |  | 0.08 (0.03, 0.21) | 0.06 (0.02, 0.18) | -0.93 (-7.59, 5.78) |  | 0.12 (0.04, 0.3) | 0.1 (0.04, 0.2) | -0.59 (-6.5, 5.19) |
| **Philippines** |  |  |  |  |  |  |  |  |  |  |  |  |
| LCHB |  | 2.56 (1.79, 3.23) | 2.5 (2.01, 3.14) | -0.08 (-1.53, 1.81) |  | 4.3 (2.95, 5.32) | 4 (3.09, 5.23) | -0.23 (-1.75, 1.85) |  | 0.79 (0.54, 1.24) | 0.95 (0.72, 1.22) | 0.59 (-1.75, 2.63) |
| LCHC |  | 0.56 (0.38, 0.81) | 0.85 (0.66, 1.08) | 1.35 (-0.66, 3.37) |  | 0.68 (0.44, 0.94) | 0.93 (0.67, 1.22) | 1.01 (-1.09, 3.29) |  | 0.44 (0.29, 0.72) | 0.78 (0.59, 0.99) | 1.85 (-0.64, 3.96) |
| LCAL |  | 0.83 (0.55, 1.12) | 1.26 (0.96, 1.61) | 1.35 (-0.5, 3.46) |  | 1.39 (0.9, 1.85) | 2.03 (1.48, 2.69) | 1.22 (-0.72, 3.53) |  | 0.25 (0.16, 0.42) | 0.47 (0.34, 0.62) | 2.04 (-0.68, 4.37) |
| LCNA |  | 0.27 (0.18, 0.37) | 0.41 (0.33, 0.52) | 1.35 (-0.37, 3.42) |  | 0.33 (0.21, 0.44) | 0.47 (0.35, 0.62) | 1.14 (-0.74, 3.49) |  | 0.2 (0.14, 0.33) | 0.36 (0.27, 0.47) | 1.9 (-0.65, 3.91) |
| LCOT |  | 0.14 (0.1, 0.19) | 0.16 (0.12, 0.2) | 0.43 (-1.48, 2.24) |  | 0.18 (0.12, 0.24) | 0.19 (0.14, 0.25) | 0.17 (-1.74, 2.37) |  | 0.1 (0.07, 0.15) | 0.13 (0.09, 0.17) | 0.85 (-1.65, 2.86) |
| **Samoa** |  |  |  |  |  |  |  |  |  |  |  |  |
| LCHB |  | 1.68 (1.2, 2.29) | 2.03 (1.36, 2.88) | 0.61 (-1.68, 2.82) |  | 2.68 (1.87, 3.72) | 3.21 (2.14, 4.64) | 0.58 (-1.78, 2.93) |  | 0.58 (0.38, 0.84) | 0.78 (0.46, 1.25) | 0.96 (-1.94, 3.84) |
| LCHC |  | 0.47 (0.29, 0.71) | 0.63 (0.38, 0.96) | 0.95 (-2.02, 3.86) |  | 0.54 (0.32, 0.87) | 0.67 (0.38, 1.1) | 0.7 (-2.67, 3.98) |  | 0.39 (0.25, 0.57) | 0.58 (0.35, 0.88) | 1.28 (-1.57, 4.06) |
| LCAL |  | 0.36 (0.22, 0.56) | 0.45 (0.26, 0.72) | 0.72 (-2.48, 3.82) |  | 0.59 (0.35, 0.91) | 0.72 (0.41, 1.18) | 0.64 (-2.57, 3.92) |  | 0.11 (0.07, 0.17) | 0.16 (0.09, 0.26) | 1.21 (-2.05, 4.23) |
| LCNA |  | 0.26 (0.16, 0.4) | 0.37 (0.22, 0.54) | 1.14 (-1.93, 3.92) |  | 0.3 (0.17, 0.5) | 0.4 (0.24, 0.67) | 0.93 (-2.37, 4.42) |  | 0.21 (0.13, 0.32) | 0.33 (0.18, 0.49) | 1.46 (-1.86, 4.28) |
| LCOT |  | 0.11 (0.07, 0.17) | 0.14 (0.08, 0.22) | 0.78 (-2.43, 3.69) |  | 0.12 (0.07, 0.2) | 0.15 (0.08, 0.24) | 0.72 (-2.96, 3.97) |  | 0.1 (0.06, 0.15) | 0.14 (0.08, 0.22) | 1.09 (-2.03, 4.19) |
| **Singapore** |  |  |  |  |  |  |  |  |  |  |  |  |
| LCHB |  | 5.77 (4.95, 6.63) | 12.45 (10, 15.1) | 2.48 (1.33, 3.6) |  | 9.36 (8.11, 10.71) | 19.46 (15.69, 23.29) | 2.36 (1.23, 3.4) |  | 2.14 (1.67, 2.63) | 5.2 (3.85, 6.9) | 2.86 (1.23, 4.58) |
| LCHC |  | 1.43 (0.98, 1.98) | 4.73 (3.16, 6.55) | 3.86 (1.51, 6.13) |  | 1.62 (1.05, 2.33) | 5.32 (3.51, 7.76) | 3.84 (1.32, 6.45) |  | 1.24 (0.86, 1.64) | 4.13 (2.84, 5.64) | 3.88 (1.77, 6.07) |
| LCAL |  | 0.49 (0.32, 0.73) | 1.47 (0.94, 2.24) | 3.54 (0.82, 6.28) |  | 0.83 (0.54, 1.25) | 2.42 (1.53, 3.7) | 3.45 (0.65, 6.21) |  | 0.14 (0.09, 0.22) | 0.48 (0.3, 0.72) | 3.97 (1, 6.71) |
| LCNA |  | 0.31 (0.2, 0.46) | 1.07 (0.69, 1.6) | 4 (1.31, 6.71) |  | 0.35 (0.23, 0.56) | 1.17 (0.7, 1.84) | 3.89 (0.72, 6.71) |  | 0.25 (0.17, 0.37) | 0.97 (0.63, 1.43) | 4.37 (1.72, 6.87) |
| LCOT |  | 0.21 (0.15, 0.29) | 0.52 (0.33, 0.75) | 2.92 (0.42, 5.19) |  | 0.25 (0.17, 0.36) | 0.57 (0.35, 0.85) | 2.66 (-0.09, 5.19) |  | 0.17 (0.12, 0.24) | 0.46 (0.29, 0.67) | 3.21 (0.61, 5.55) |
| **Solomon Islands** |  |  |  |  |  |  |  |  |  |  |  |  |
| LCHB |  | 2.14 (0.65, 4.58) | 2.05 (1.25, 3.31) | -0.14 (-4.19, 5.25) |  | 3.49 (1.03, 7.54) | 3.23 (1.96, 5.55) | -0.25 (-4.35, 5.43) |  | 0.69 (0.22, 1.49) | 0.82 (0.49, 1.46) | 0.56 (-3.59, 6.11) |
| LCHC |  | 0.42 (0.15, 0.98) | 0.45 (0.25, 0.81) | 0.22 (-4.41, 5.44) |  | 0.52 (0.16, 1.27) | 0.48 (0.25, 0.91) | -0.26 (-5.24, 5.61) |  | 0.32 (0.12, 0.76) | 0.42 (0.23, 0.77) | 0.88 (-3.86, 6) |
| LCAL |  | 0.33 (0.1, 0.77) | 0.34 (0.19, 0.58) | 0.1 (-4.51, 5.67) |  | 0.55 (0.16, 1.28) | 0.54 (0.3, 0.97) | -0.06 (-4.68, 5.81) |  | 0.09 (0.03, 0.22) | 0.12 (0.07, 0.23) | 0.93 (-3.69, 6.57) |
| LCNA |  | 0.2 (0.07, 0.47) | 0.25 (0.14, 0.42) | 0.72 (-3.91, 5.78) |  | 0.25 (0.07, 0.62) | 0.27 (0.14, 0.51) | 0.25 (-4.8, 6.41) |  | 0.15 (0.05, 0.35) | 0.23 (0.13, 0.39) | 1.38 (-3.19, 6.63) |
| LCOT |  | 0.13 (0.04, 0.31) | 0.15 (0.09, 0.26) | 0.46 (-3.99, 6.04) |  | 0.15 (0.05, 0.34) | 0.15 (0.08, 0.26) | 0 (-4.67, 5.32) |  | 0.12 (0.04, 0.27) | 0.15 (0.09, 0.26) | 0.72 (-3.54, 6.04) |
| **South Korea** |  |  |  |  |  |  |  |  |  |  |  |  |
| LCHB |  | 20.85 (14.9, 27.26) | 38.91 (30.63, 48.81) | 2.01 (0.38, 3.83) |  | 35.61 (25.18, 47.11) | 67.09 (52.55, 84.17) | 2.04 (0.35, 3.89) |  | 5.97 (3.96, 8.17) | 10.51 (7.27, 14.9) | 1.82 (-0.38, 4.27) |
| LCHC |  | 3.75 (2.43, 5.53) | 10.98 (7.26, 15.88) | 3.47 (0.88, 6.06) |  | 2.91 (1.72, 4.69) | 9.44 (5.64, 14.47) | 3.8 (0.6, 6.87) |  | 4.6 (2.89, 6.69) | 12.54 (8.23, 17.93) | 3.24 (0.67, 5.89) |
| LCAL |  | 3.62 (2.25, 5.37) | 11.27 (7.38, 16.96) | 3.66 (1.03, 6.52) |  | 5.66 (3.42, 8.58) | 18.24 (11.81, 27.5) | 3.77 (1.03, 6.72) |  | 1.56 (0.92, 2.42) | 4.24 (2.58, 6.36) | 3.23 (0.21, 6.24) |
| LCNA |  | 1.46 (0.96, 2.16) | 4.45 (2.85, 6.85) | 3.6 (0.89, 6.34) |  | 1.37 (0.83, 2.12) | 4.44 (2.63, 6.93) | 3.79 (0.7, 6.85) |  | 1.55 (0.96, 2.31) | 4.47 (2.8, 6.92) | 3.42 (0.62, 6.37) |
| LCOT |  | 0.93 (0.6, 1.4) | 2.02 (1.29, 2.91) | 2.5 (-0.26, 5.09) |  | 0.95 (0.57, 1.5) | 2.16 (1.38, 3.32) | 2.65 (-0.27, 5.68) |  | 0.92 (0.57, 1.42) | 1.87 (1.14, 2.7) | 2.29 (-0.71, 5.02) |
| **Tokelau** |  |  |  |  |  |  |  |  |  |  |  |  |
| LCHB |  | 1.72 (0.99, 3.08) | 3.05 (1.84, 4.89) | 1.85 (-1.66, 5.15) |  | 2.07 (1.14, 4.04) | 4.41 (2.51, 7.38) | 2.44 (-1.54, 6.02) |  | 1.37 (0.75, 2.54) | 1.63 (0.97, 2.69) | 0.56 (-3.11, 4.12) |
| LCHC |  | 0.73 (0.4, 1.39) | 1.04 (0.58, 1.76) | 1.14 (-2.82, 4.78) |  | 0.48 (0.24, 1.01) | 0.95 (0.51, 1.63) | 2.2 (-2.2, 6.18) |  | 0.97 (0.51, 1.89) | 1.12 (0.66, 1.93) | 0.46 (-3.39, 4.29) |
| LCAL |  | 0.36 (0.18, 0.71) | 0.7 (0.38, 1.22) | 2.15 (-2.02, 6.17) |  | 0.46 (0.23, 0.99) | 1.06 (0.57, 1.88) | 2.69 (-1.78, 6.78) |  | 0.25 (0.12, 0.51) | 0.33 (0.17, 0.59) | 0.9 (-3.54, 5.14) |
| LCNA |  | 0.31 (0.16, 0.58) | 0.57 (0.31, 0.95) | 1.96 (-2.02, 5.75) |  | 0.21 (0.1, 0.43) | 0.54 (0.29, 0.94) | 3.05 (-1.27, 7.23) |  | 0.4 (0.21, 0.76) | 0.6 (0.32, 1.07) | 1.31 (-2.79, 5.25) |
| LCOT |  | 0.16 (0.08, 0.31) | 0.22 (0.12, 0.38) | 1.03 (-3.06, 5.03) |  | 0.09 (0.04, 0.19) | 0.18 (0.1, 0.33) | 2.24 (-2.07, 6.81) |  | 0.22 (0.12, 0.43) | 0.25 (0.14, 0.46) | 0.41 (-3.62, 4.33) |
| **Tonga** |  |  |  |  |  |  |  |  |  |  |  |  |
| LCHB |  | 8.21 (5.59, 11.88) | 10.24 (6.75, 15.15) | 0.71 (-1.82, 3.22) |  | 13.25 (9.19, 19.37) | 17.5 (11.33, 26.61) | 0.9 (-1.73, 3.43) |  | 3.09 (1.84, 4.98) | 3.02 (1.91, 4.5) | -0.07 (-3.09, 2.88) |
| LCHC |  | 2.21 (1.25, 3.69) | 3.03 (1.83, 4.64) | 1.02 (-2.26, 4.23) |  | 2.57 (1.45, 4.37) | 3.75 (2.12, 6.18) | 1.22 (-2.33, 4.68) |  | 1.85 (0.98, 3.27) | 2.32 (1.39, 3.4) | 0.73 (-2.76, 4.01) |
| LCAL |  | 1.46 (0.84, 2.43) | 2.05 (1.19, 3.34) | 1.09 (-2.3, 4.45) |  | 2.44 (1.39, 4.11) | 3.55 (2.04, 5.86) | 1.21 (-2.26, 4.64) |  | 0.47 (0.24, 0.83) | 0.55 (0.31, 0.88) | 0.51 (-3.18, 4.19) |
| LCNA |  | 1.17 (0.69, 1.94) | 1.89 (1.16, 2.86) | 1.55 (-1.66, 4.59) |  | 1.37 (0.81, 2.32) | 2.41 (1.36, 3.97) | 1.82 (-1.72, 5.13) |  | 0.96 (0.52, 1.69) | 1.38 (0.84, 2.13) | 1.17 (-2.26, 4.55) |
| LCOT |  | 0.54 (0.33, 0.88) | 0.68 (0.4, 1.07) | 0.74 (-2.54, 3.79) |  | 0.58 (0.34, 0.98) | 0.81 (0.46, 1.41) | 1.08 (-2.44, 4.59) |  | 0.5 (0.28, 0.88) | 0.55 (0.32, 0.89) | 0.31 (-3.26, 3.73) |
| **Tuvalu** |  |  |  |  |  |  |  |  |  |  |  |  |
| LCHB |  | 2.07 (1.36, 3.15) | 2.51 (1.58, 3.82) | 0.62 (-2.23, 3.33) |  | 2.86 (1.85, 4.69) | 3.63 (2.2, 5.92) | 0.77 (-2.44, 3.75) |  | 1.34 (0.78, 2.24) | 1.29 (0.8, 2.02) | -0.12 (-3.32, 3.07) |
| LCHC |  | 0.67 (0.39, 1.16) | 0.83 (0.5, 1.35) | 0.69 (-2.71, 4.01) |  | 0.53 (0.29, 0.97) | 0.72 (0.38, 1.28) | 0.99 (-3.02, 4.79) |  | 0.8 (0.45, 1.45) | 0.95 (0.58, 1.58) | 0.55 (-2.96, 4.05) |
| LCAL |  | 0.38 (0.22, 0.66) | 0.53 (0.3, 0.89) | 1.07 (-2.54, 4.51) |  | 0.55 (0.31, 0.98) | 0.78 (0.42, 1.37) | 1.13 (-2.73, 4.79) |  | 0.21 (0.11, 0.4) | 0.26 (0.14, 0.44) | 0.69 (-3.39, 4.47) |
| LCNA |  | 0.29 (0.17, 0.51) | 0.45 (0.27, 0.71) | 1.42 (-2.05, 4.61) |  | 0.24 (0.14, 0.43) | 0.41 (0.22, 0.75) | 1.73 (-2.16, 5.41) |  | 0.34 (0.19, 0.65) | 0.49 (0.3, 0.79) | 1.18 (-2.49, 4.6) |
| LCOT |  | 0.17 (0.1, 0.3) | 0.19 (0.12, 0.32) | 0.36 (-2.96, 3.75) |  | 0.12 (0.07, 0.22) | 0.16 (0.09, 0.31) | 0.93 (-2.88, 4.8) |  | 0.21 (0.12, 0.38) | 0.22 (0.14, 0.38) | 0.15 (-3.22, 3.72) |
| **Vanuatu** |  |  |  |  |  |  |  |  |  |  |  |  |
| LCHB |  | 1.46 (0.86, 2.72) | 1.66 (1.02, 2.72) | 0.41 (-3.16, 3.71) |  | 2.2 (1.36, 4.27) | 2.51 (1.48, 4.32) | 0.43 (-3.42, 3.73) |  | 0.67 (0.32, 1.41) | 0.8 (0.48, 1.31) | 0.57 (-3.48, 4.55) |
| LCHC |  | 0.35 (0.19, 0.68) | 0.47 (0.27, 0.8) | 0.95 (-2.98, 4.64) |  | 0.36 (0.19, 0.73) | 0.46 (0.23, 0.89) | 0.79 (-3.73, 4.98) |  | 0.34 (0.17, 0.73) | 0.48 (0.3, 0.78) | 1.11 (-2.87, 4.91) |
| LCAL |  | 0.22 (0.12, 0.45) | 0.3 (0.16, 0.5) | 1 (-3.34, 4.6) |  | 0.35 (0.2, 0.73) | 0.47 (0.24, 0.82) | 0.95 (-3.59, 4.55) |  | 0.09 (0.04, 0.2) | 0.13 (0.07, 0.22) | 1.19 (-3.39, 5.5) |
| LCNA |  | 0.16 (0.08, 0.33) | 0.25 (0.15, 0.4) | 1.44 (-2.54, 5.19) |  | 0.17 (0.09, 0.34) | 0.24 (0.12, 0.48) | 1.11 (-3.36, 5.4) |  | 0.16 (0.07, 0.34) | 0.25 (0.14, 0.41) | 1.44 (-2.86, 5.7) |
| LCOT |  | 0.1 (0.06, 0.21) | 0.13 (0.07, 0.22) | 0.85 (-3.54, 4.19) |  | 0.1 (0.05, 0.19) | 0.11 (0.06, 0.22) | 0.31 (-3.72, 4.78) |  | 0.11 (0.05, 0.24) | 0.14 (0.08, 0.24) | 0.78 (-3.54, 5.06) |
| **Viet Nam** |  |  |  |  |  |  |  |  |  |  |  |  |
| LCHB |  | 5.21 (3.45, 7.21) | 7.41 (4.84, 11.35) | 1.14 (-1.29, 3.84) |  | 8.96 (5.92, 12.56) | 12.29 (7.74, 19.49) | 1.02 (-1.56, 3.84) |  | 1.67 (1.1, 2.32) | 2.55 (1.54, 3.78) | 1.37 (-1.32, 3.98) |
| LCHC |  | 1.41 (0.86, 2.27) | 2.28 (1.3, 3.69) | 1.55 (-1.8, 4.7) |  | 1.98 (1.12, 3.26) | 3.02 (1.58, 5.32) | 1.36 (-2.34, 5.03) |  | 0.87 (0.56, 1.32) | 1.54 (0.89, 2.35) | 1.84 (-1.27, 4.63) |
| LCAL |  | 1.6 (0.99, 2.51) | 4.32 (2.56, 6.94) | 3.2 (0.06, 6.28) |  | 2.85 (1.73, 4.51) | 7.4 (4.26, 12.03) | 3.08 (-0.18, 6.26) |  | 0.42 (0.27, 0.65) | 1.26 (0.74, 2.05) | 3.54 (0.42, 6.54) |
| LCNA |  | 0.62 (0.38, 0.97) | 1.18 (0.72, 1.76) | 2.08 (-0.96, 4.94) |  | 0.76 (0.44, 1.28) | 1.39 (0.8, 2.26) | 1.95 (-1.52, 5.28) |  | 0.48 (0.3, 0.76) | 0.97 (0.59, 1.49) | 2.27 (-0.82, 5.17) |
| LCOT |  | 0.27 (0.17, 0.4) | 0.42 (0.24, 0.66) | 1.43 (-1.65, 4.38) |  | 0.35 (0.21, 0.53) | 0.51 (0.28, 0.85) | 1.21 (-2.06, 4.51) |  | 0.2 (0.12, 0.31) | 0.34 (0.2, 0.53) | 1.71 (-1.41, 4.79) |
| **Western Pacific Region** |  |  |  |  |  |  |  |  |  |  |  |  |
| LCHB |  | 6.62 (5.65, 7.69) | 10.77 (8.64, 13.45) | 1.57 (0.38, 2.8) |  | 10.95 (9.11, 13.03) | 18.15 (14.26, 23.3) | 1.63 (0.29, 3.03) |  | 2.09 (1.69, 2.5) | 3.13 (2.41, 3.96) | 1.3 (-0.12, 2.75) |
| LCHC |  | 2.83 (2.53, 3.15) | 5.49 (4.66, 6.3) | 2.14 (1.26, 2.94) |  | 3.39 (3.03, 3.79) | 6.2 (5.31, 7.34) | 1.95 (1.09, 2.85) |  | 2.24 (1.92, 2.58) | 4.75 (3.84, 5.62) | 2.42 (1.28, 3.46) |
| LCAL |  | 1.14 (0.92, 1.4) | 2.54 (1.96, 3.27) | 2.58 (1.09, 4.09) |  | 1.68 (1.35, 2.08) | 3.76 (2.84, 4.91) | 2.6 (1, 4.17) |  | 0.57 (0.45, 0.71) | 1.27 (0.96, 1.61) | 2.58 (0.97, 4.11) |
| LCNA |  | 0.5 (0.41, 0.59) | 1.16 (0.91, 1.43) | 2.71 (1.4, 4.03) |  | 0.53 (0.43, 0.64) | 1.25 (0.93, 1.64) | 2.77 (1.21, 4.32) |  | 0.46 (0.37, 0.55) | 1.06 (0.84, 1.34) | 2.69 (1.37, 4.15) |
| LCOT |  | 0.54 (0.44, 0.66) | 0.85 (0.67, 1.07) | 1.46 (0.05, 2.87) |  | 0.57 (0.46, 0.7) | 0.93 (0.7, 1.26) | 1.58 (0, 3.25) |  | 0.52 (0.41, 0.65) | 0.77 (0.58, 0.97) | 1.27 (-0.37, 2.78) |
| **Whole World** |  |  |  |  |  |  |  |  |  |  |  |  |
| LCHB |  | 2.6 (2.28, 3.02) | 3.65 (3.01, 4.43) | 1.09 (-0.01, 2.14) |  | 4.22 (3.64, 4.88) | 6.02 (4.88, 7.48) | 1.15 (0, 2.32) |  | 0.96 (0.78, 1.16) | 1.26 (1.02, 1.55) | 0.88 (-0.41, 2.22) |
| LCHC |  | 1.42 (1.25, 1.64) | 2.55 (2.2, 2.9) | 1.89 (0.95, 2.71) |  | 1.59 (1.4, 1.84) | 2.88 (2.47, 3.36) | 1.92 (0.95, 2.82) |  | 1.24 (1.07, 1.47) | 2.22 (1.87, 2.53) | 1.88 (0.78, 2.78) |
| LCAL |  | 0.84 (0.69, 1.02) | 1.67 (1.36, 2.02) | 2.22 (0.93, 3.47) |  | 1.3 (1.09, 1.58) | 2.67 (2.2, 3.2) | 2.32 (1.07, 3.47) |  | 0.37 (0.3, 0.46) | 0.67 (0.54, 0.82) | 1.92 (0.52, 3.24) |
| LCNA |  | 0.31 (0.25, 0.38) | 0.66 (0.54, 0.8) | 2.44 (1.13, 3.75) |  | 0.31 (0.25, 0.38) | 0.71 (0.56, 0.88) | 2.67 (1.25, 4.06) |  | 0.31 (0.24, 0.38) | 0.62 (0.5, 0.75) | 2.24 (0.89, 3.68) |
| LCOT |  | 0.26 (0.22, 0.32) | 0.4 (0.33, 0.49) | 1.39 (0.1, 2.58) |  | 0.26 (0.22, 0.32) | 0.43 (0.34, 0.54) | 1.62 (0.2, 2.9) |  | 0.26 (0.21, 0.33) | 0.37 (0.3, 0.46) | 1.14 (-0.31, 2.53) |

Abbreviation: UI, Uncertainty interval; EAPC, Estimated annual percentage change; CI, Confidence interval; LCHB, Liver cancer by HBC; LCHC, Liver cancer by HCV; LCAL, Liver cancer by alcoholic use; LCNA; Liver cancer by nonalcoholic steatohepatitis (NASH); LCOT, Liver cancer by other causes

Table S12. Age-standardized mortality rates (per 100,000) for specific liver cancer in the Western Pacific region by member state and sex, in 1990 to 2021.

| **Region/Liver cancer cause** |  | **Both sexes combined** | | |  | **Male** | | |  | **Female** | | |
| --- | --- | --- | --- | --- | --- | --- | --- | --- | --- | --- | --- | --- |
|  |  | **1990 (95% UI)** | **2021 (95% UI)** | **EAPC, %**  **(95% CI)** |  | **1990 (95% UI)** | **2021 (95% UI)** | **EAPC, %**  **(95% CI)** |  | **1990 (95% UI)** | **2021 (95% UI)** | **EAPC, %**  **(95% CI)** |
| **American Samoa** |  |  |  |  |  |  |  |  |  |  |  |  |
| LCHB |  | 1.12 (0.79, 1.56) | 3.21 (2.26, 4.45) | 3.4 (1.2, 5.58) |  | 1.59 (1.09, 2.3) | 4.51 (3.12, 6.2) | 3.36 (0.98, 5.61) |  | 0.63 (0.42, 0.99) | 1.85 (1.24, 2.77) | 3.47 (0.73, 6.08) |
| LCHC |  | 0.38 (0.24, 0.58) | 1.27 (0.83, 1.85) | 3.89 (1.16, 6.59) |  | 0.32 (0.19, 0.53) | 1.03 (0.64, 1.57) | 3.77 (0.61, 6.81) |  | 0.45 (0.28, 0.68) | 1.53 (1.01, 2.21) | 3.95 (1.28, 6.66) |
| LCAL |  | 0.21 (0.12, 0.32) | 0.64 (0.4, 0.98) | 3.59 (0.72, 6.77) |  | 0.3 (0.18, 0.49) | 0.93 (0.57, 1.45) | 3.65 (0.49, 6.73) |  | 0.1 (0.06, 0.16) | 0.33 (0.2, 0.54) | 3.85 (0.72, 7.09) |
| LCNA |  | 0.24 (0.16, 0.36) | 0.9 (0.59, 1.32) | 4.26 (1.59, 6.81) |  | 0.21 (0.13, 0.34) | 0.77 (0.47, 1.16) | 4.19 (1.04, 7.06) |  | 0.28 (0.18, 0.42) | 1.03 (0.64, 1.56) | 4.2 (1.36, 6.97) |
| LCOT |  | 0.09 (0.06, 0.14) | 0.24 (0.15, 0.37) | 3.16 (0.22, 5.87) |  | 0.07 (0.04, 0.11) | 0.19 (0.12, 0.3) | 3.22 (0.28, 6.5) |  | 0.11 (0.07, 0.17) | 0.29 (0.18, 0.46) | 3.13 (0.18, 6.07) |
| **Australia** |  |  |  |  |  |  |  |  |  |  |  |  |
| LCHB |  | 0.33 (0.23, 0.48) | 1.02 (0.68, 1.49) | 3.64 (1.12, 6.03) |  | 0.54 (0.37, 0.77) | 1.63 (1.06, 2.36) | 3.56 (1.03, 5.98) |  | 0.13 (0.09, 0.19) | 0.43 (0.29, 0.63) | 3.86 (1.36, 6.28) |
| LCHC |  | 0.62 (0.45, 0.82) | 2.97 (2.24, 3.94) | 5.05 (3.24, 7) |  | 0.7 (0.49, 0.99) | 3.26 (2.33, 4.48) | 4.96 (2.76, 7.14) |  | 0.53 (0.4, 0.67) | 2.69 (2.01, 3.47) | 5.24 (3.54, 6.97) |
| LCAL |  | 0.9 (0.69, 1.11) | 3.45 (2.67, 4.37) | 4.33 (2.83, 5.95) |  | 1.55 (1.19, 1.91) | 5.84 (4.55, 7.39) | 4.28 (2.8, 5.89) |  | 0.26 (0.19, 0.36) | 1.12 (0.76, 1.62) | 4.71 (2.41, 6.91) |
| LCNA |  | 0.17 (0.12, 0.24) | 1.01 (0.69, 1.41) | 5.75 (3.41, 7.95) |  | 0.19 (0.12, 0.27) | 1.08 (0.69, 1.52) | 5.61 (3.03, 8.19) |  | 0.16 (0.11, 0.22) | 0.94 (0.62, 1.34) | 5.71 (3.34, 8.06) |
| LCOT |  | 0.09 (0.06, 0.12) | 0.36 (0.26, 0.5) | 4.47 (2.49, 6.84) |  | 0.09 (0.06, 0.13) | 0.36 (0.23, 0.51) | 4.47 (1.84, 6.9) |  | 0.09 (0.06, 0.12) | 0.36 (0.24, 0.52) | 4.47 (2.24, 6.97) |
| **Brunei Darussalam** |  |  |  |  |  |  |  |  |  |  |  |  |
| LCHB |  | 3.08 (2.18, 4.37) | 3.56 (2.51, 4.85) | 0.47 (-1.79, 2.58) |  | 4.62 (3.31, 6.42) | 5.32 (3.82, 7.16) | 0.46 (-1.67, 2.49) |  | 1.34 (0.88, 2) | 1.6 (0.99, 2.48) | 0.57 (-2.27, 3.34) |
| LCHC |  | 1.72 (1.01, 2.58) | 2.45 (1.6, 3.47) | 1.14 (-1.54, 3.98) |  | 1.7 (0.96, 2.63) | 2.33 (1.48, 3.54) | 1.02 (-1.85, 4.21) |  | 1.74 (1.04, 2.59) | 2.59 (1.65, 3.89) | 1.28 (-1.45, 4.26) |
| LCAL |  | 0.48 (0.29, 0.77) | 0.61 (0.39, 0.95) | 0.77 (-2.19, 3.83) |  | 0.73 (0.44, 1.19) | 0.91 (0.57, 1.42) | 0.71 (-2.37, 3.78) |  | 0.19 (0.11, 0.32) | 0.27 (0.16, 0.45) | 1.13 (-2.24, 4.54) |
| LCNA |  | 0.31 (0.18, 0.51) | 0.47 (0.29, 0.72) | 1.34 (-1.82, 4.47) |  | 0.3 (0.17, 0.5) | 0.43 (0.26, 0.66) | 1.16 (-2.11, 4.38) |  | 0.32 (0.19, 0.56) | 0.53 (0.31, 0.85) | 1.63 (-1.91, 4.83) |
| LCOT |  | 0.21 (0.13, 0.32) | 0.28 (0.17, 0.43) | 0.93 (-2.04, 3.86) |  | 0.2 (0.12, 0.32) | 0.26 (0.15, 0.42) | 0.85 (-2.44, 4.04) |  | 0.21 (0.13, 0.33) | 0.3 (0.17, 0.49) | 1.15 (-2.14, 4.28) |
| **Cambodia** |  |  |  |  |  |  |  |  |  |  |  |  |
| LCHB |  | 1.83 (0.94, 3.52) | 1.79 (0.86, 3.58) | -0.07 (-4.55, 4.31) |  | 2.22 (1.09, 5.19) | 2.18 (0.86, 5.38) | -0.06 (-5.8, 5.15) |  | 1.47 (0.62, 3.02) | 1.42 (0.64, 2.48) | -0.11 (-5, 4.47) |
| LCHC |  | 1.05 (0.5, 2.05) | 1.32 (0.7, 2.3) | 0.74 (-3.47, 4.92) |  | 0.79 (0.36, 1.91) | 0.89 (0.36, 2.33) | 0.38 (-5.38, 6.02) |  | 1.29 (0.5, 2.68) | 1.74 (0.82, 3.01) | 0.97 (-3.82, 5.79) |
| LCAL |  | 0.57 (0.25, 1.23) | 1.21 (0.55, 2.47) | 2.43 (-2.6, 7.39) |  | 0.82 (0.35, 1.86) | 1.66 (0.67, 4.01) | 2.28 (-3.29, 7.87) |  | 0.35 (0.13, 0.83) | 0.78 (0.33, 1.45) | 2.59 (-2.98, 7.78) |
| LCNA |  | 0.31 (0.15, 0.62) | 0.44 (0.22, 0.81) | 1.13 (-3.34, 5.44) |  | 0.22 (0.09, 0.52) | 0.3 (0.12, 0.74) | 1 (-4.73, 6.8) |  | 0.4 (0.14, 0.88) | 0.58 (0.26, 1.04) | 1.2 (-3.93, 6.47) |
| LCOT |  | 0.13 (0.06, 0.26) | 0.15 (0.07, 0.28) | 0.46 (-4.23, 4.97) |  | 0.09 (0.04, 0.22) | 0.1 (0.04, 0.25) | 0.34 (-5.5, 5.91) |  | 0.17 (0.06, 0.36) | 0.19 (0.08, 0.35) | 0.36 (-4.85, 5.69) |
| **China** |  |  |  |  |  |  |  |  |  |  |  |  |
| LCHB |  | 5.22 (4.31, 6.22) | 7.04 (5.46, 9.08) | 0.96 (-0.42, 2.4) |  | 8.49 (6.81, 10.37) | 11.68 (8.78, 15.76) | 1.03 (-0.54, 2.71) |  | 1.74 (1.35, 2.15) | 2.18 (1.61, 2.86) | 0.73 (-0.93, 2.42) |
| LCHC |  | 1.3 (1.05, 1.57) | 2.45 (1.93, 3.02) | 2.04 (0.67, 3.41) |  | 1.05 (0.8, 1.34) | 2.1 (1.54, 2.84) | 2.24 (0.45, 4.09) |  | 1.56 (1.21, 1.92) | 2.83 (2.17, 3.58) | 1.92 (0.39, 3.5) |
| LCAL |  | 0.64 (0.5, 0.82) | 1.29 (0.96, 1.7) | 2.26 (0.51, 3.95) |  | 0.81 (0.61, 1.08) | 1.68 (1.17, 2.34) | 2.35 (0.26, 4.34) |  | 0.47 (0.35, 0.6) | 0.88 (0.64, 1.17) | 2.02 (0.21, 3.89) |
| LCNA |  | 0.35 (0.28, 0.43) | 0.73 (0.56, 0.93) | 2.37 (0.85, 3.87) |  | 0.35 (0.27, 0.45) | 0.76 (0.53, 1.04) | 2.5 (0.53, 4.35) |  | 0.35 (0.27, 0.44) | 0.7 (0.53, 0.94) | 2.24 (0.6, 4.02) |
| LCOT |  | 0.42 (0.34, 0.53) | 0.56 (0.43, 0.72) | 0.93 (-0.67, 2.42) |  | 0.42 (0.32, 0.54) | 0.59 (0.42, 0.85) | 1.1 (-0.81, 3.15) |  | 0.43 (0.32, 0.55) | 0.54 (0.4, 0.71) | 0.73 (-1.03, 2.57) |
| **Cook Islands** |  |  |  |  |  |  |  |  |  |  |  |  |
| LCHB |  | 3.62 (2.61, 4.96) | 6.09 (4.19, 8.63) | 1.68 (-0.54, 3.86) |  | 5.18 (3.74, 7.28) | 10.19 (7.1, 14.65) | 2.18 (-0.08, 4.4) |  | 1.92 (1.26, 2.73) | 2.22 (1.39, 3.41) | 0.47 (-2.18, 3.21) |
| LCHC |  | 1.44 (0.93, 2.08) | 2.99 (2, 4.33) | 2.36 (-0.13, 4.96) |  | 1.26 (0.75, 1.93) | 3.21 (2.1, 4.7) | 3.02 (0.27, 5.92) |  | 1.64 (1.07, 2.42) | 2.79 (1.8, 4.07) | 1.71 (-0.95, 4.31) |
| LCAL |  | 0.77 (0.47, 1.19) | 3.65 (2.35, 5.58) | 5.02 (2.2, 7.98) |  | 1.14 (0.7, 1.78) | 6.2 (3.97, 9.32) | 5.46 (2.59, 8.35) |  | 0.36 (0.22, 0.58) | 1.25 (0.75, 1.95) | 4.02 (0.83, 7.04) |
| LCNA |  | 0.73 (0.49, 1.09) | 1.96 (1.25, 2.89) | 3.19 (0.44, 5.72) |  | 0.67 (0.42, 1.05) | 2.24 (1.37, 3.39) | 3.89 (0.86, 6.74) |  | 0.8 (0.51, 1.21) | 1.68 (1.01, 2.56) | 2.39 (-0.58, 5.2) |
| LCOT |  | 0.27 (0.18, 0.41) | 0.44 (0.27, 0.67) | 1.58 (-1.35, 4.24) |  | 0.23 (0.14, 0.37) | 0.48 (0.28, 0.75) | 2.37 (-0.9, 5.41) |  | 0.32 (0.21, 0.51) | 0.39 (0.23, 0.63) | 0.64 (-2.57, 3.54) |
| **Federated States of Micronesia** |  |  |  |  |  |  |  |  |  |  |  |  |
| LCHB |  | 1.36 (0.87, 2.15) | 2.07 (1.17, 3.57) | 1.36 (-1.96, 4.55) |  | 1.92 (1.1, 3.31) | 3.04 (1.61, 5.84) | 1.48 (-2.32, 5.39) |  | 0.77 (0.48, 1.14) | 1.06 (0.49, 1.74) | 1.03 (-2.72, 4.15) |
| LCHC |  | 0.48 (0.31, 0.78) | 0.77 (0.44, 1.3) | 1.52 (-1.85, 4.62) |  | 0.4 (0.21, 0.79) | 0.66 (0.33, 1.46) | 1.62 (-2.82, 6.26) |  | 0.57 (0.34, 0.9) | 0.87 (0.39, 1.38) | 1.36 (-2.7, 4.52) |
| LCAL |  | 0.3 (0.16, 0.51) | 0.46 (0.25, 0.9) | 1.38 (-2.3, 5.57) |  | 0.44 (0.22, 0.82) | 0.69 (0.33, 1.43) | 1.45 (-2.94, 6.04) |  | 0.15 (0.09, 0.25) | 0.22 (0.11, 0.39) | 1.24 (-2.65, 4.73) |
| LCNA |  | 0.22 (0.14, 0.35) | 0.42 (0.24, 0.67) | 2.09 (-1.22, 5.05) |  | 0.19 (0.1, 0.36) | 0.38 (0.18, 0.79) | 2.24 (-2.24, 6.67) |  | 0.25 (0.15, 0.39) | 0.46 (0.21, 0.74) | 1.97 (-2, 5.15) |
| LCOT |  | 0.11 (0.07, 0.17) | 0.16 (0.09, 0.28) | 1.21 (-2.05, 4.47) |  | 0.09 (0.04, 0.17) | 0.14 (0.06, 0.3) | 1.43 (-3.36, 6.5) |  | 0.13 (0.08, 0.2) | 0.19 (0.09, 0.33) | 1.22 (-2.58, 4.57) |
| **Fiji** |  |  |  |  |  |  |  |  |  |  |  |  |
| LCHB |  | 1.24 (0.85, 1.82) | 2.03 (1.29, 2.93) | 1.59 (-1.11, 3.99) |  | 1.78 (1.17, 2.68) | 3.02 (1.91, 4.55) | 1.71 (-1.09, 4.38) |  | 0.68 (0.4, 1.13) | 1.02 (0.63, 1.53) | 1.31 (-1.88, 4.33) |
| LCHC |  | 0.4 (0.23, 0.69) | 0.86 (0.55, 1.28) | 2.47 (-0.73, 5.54) |  | 0.36 (0.21, 0.6) | 0.78 (0.46, 1.27) | 2.49 (-0.86, 5.81) |  | 0.44 (0.25, 0.8) | 0.93 (0.61, 1.34) | 2.41 (-0.87, 5.42) |
| LCAL |  | 0.22 (0.13, 0.37) | 0.49 (0.29, 0.8) | 2.58 (-0.79, 5.86) |  | 0.33 (0.19, 0.55) | 0.75 (0.41, 1.23) | 2.65 (-0.95, 6.02) |  | 0.11 (0.06, 0.2) | 0.23 (0.13, 0.36) | 2.38 (-1.39, 5.78) |
| LCNA |  | 0.18 (0.11, 0.3) | 0.46 (0.29, 0.72) | 3.03 (-0.11, 6.06) |  | 0.17 (0.1, 0.28) | 0.44 (0.25, 0.74) | 3.07 (-0.37, 6.46) |  | 0.2 (0.11, 0.35) | 0.48 (0.3, 0.74) | 2.82 (-0.5, 6.15) |
| LCOT |  | 0.1 (0.06, 0.16) | 0.16 (0.09, 0.25) | 1.52 (-1.86, 4.6) |  | 0.08 (0.05, 0.14) | 0.14 (0.08, 0.25) | 1.81 (-1.81, 5.19) |  | 0.11 (0.06, 0.2) | 0.18 (0.11, 0.28) | 1.59 (-1.93, 4.97) |
| **Guam** |  |  |  |  |  |  |  |  |  |  |  |  |
| LCHB |  | 1.05 (0.83, 1.29) | 3.85 (2.92, 4.91) | 4.19 (2.64, 5.73) |  | 1.6 (1.27, 1.95) | 6.65 (5.02, 8.41) | 4.6 (3.05, 6.1) |  | 0.42 (0.31, 0.55) | 0.98 (0.67, 1.36) | 2.73 (0.64, 4.77) |
| LCHC |  | 0.33 (0.22, 0.46) | 1.27 (0.84, 1.76) | 4.35 (1.94, 6.71) |  | 0.34 (0.21, 0.49) | 1.58 (1.02, 2.26) | 4.96 (2.37, 7.66) |  | 0.33 (0.22, 0.45) | 0.95 (0.61, 1.34) | 3.41 (0.98, 5.83) |
| LCAL |  | 0.23 (0.15, 0.33) | 1 (0.65, 1.51) | 4.74 (2.19, 7.45) |  | 0.35 (0.23, 0.51) | 1.74 (1.13, 2.63) | 5.17 (2.57, 7.86) |  | 0.08 (0.05, 0.12) | 0.25 (0.15, 0.39) | 3.68 (0.72, 6.63) |
| LCNA |  | 0.18 (0.12, 0.25) | 0.8 (0.55, 1.15) | 4.81 (2.54, 7.29) |  | 0.19 (0.13, 0.28) | 1.05 (0.69, 1.52) | 5.51 (2.91, 7.93) |  | 0.16 (0.11, 0.23) | 0.55 (0.35, 0.84) | 3.98 (1.35, 6.56) |
| LCOT |  | 0.07 (0.05, 0.1) | 0.22 (0.14, 0.31) | 3.69 (1.09, 5.89) |  | 0.07 (0.05, 0.11) | 0.28 (0.17, 0.43) | 4.47 (1.4, 6.94) |  | 0.07 (0.05, 0.1) | 0.15 (0.1, 0.22) | 2.46 (0, 4.78) |
| **Japan** |  |  |  |  |  |  |  |  |  |  |  |  |
| LCHB |  | 2.33 (1.99, 2.7) | 2.38 (1.87, 2.91) | 0.07 (-1.18, 1.23) |  | 3.82 (3.26, 4.45) | 3.51 (2.87, 4.26) | -0.27 (-1.41, 0.86) |  | 0.89 (0.74, 1.04) | 1.31 (0.95, 1.67) | 1.25 (-0.29, 2.63) |
| LCHC |  | 10.56 (9.91, 11.15) | 17.79 (14.9, 19.51) | 1.68 (0.94, 2.19) |  | 15.54 (14.63, 16.43) | 23.17 (20.75, 24.91) | 1.29 (0.75, 1.72) |  | 5.75 (5.2, 6.11) | 12.67 (9.34, 14.75) | 2.55 (1.37, 3.36) |
| LCAL |  | 2.12 (1.83, 2.51) | 2.48 (2.08, 2.97) | 0.51 (-0.61, 1.56) |  | 3.68 (3.18, 4.34) | 4.13 (3.48, 4.91) | 0.37 (-0.71, 1.4) |  | 0.61 (0.52, 0.73) | 0.91 (0.67, 1.14) | 1.29 (-0.28, 2.53) |
| LCNA |  | 0.67 (0.57, 0.78) | 1.15 (0.87, 1.45) | 1.74 (0.35, 3.01) |  | 0.78 (0.66, 0.91) | 1.14 (0.91, 1.38) | 1.22 (0, 2.38) |  | 0.55 (0.46, 0.66) | 1.17 (0.81, 1.52) | 2.43 (0.66, 3.86) |
| LCOT |  | 0.44 (0.38, 0.52) | 0.57 (0.44, 0.71) | 0.84 (-0.54, 2.02) |  | 0.55 (0.47, 0.66) | 0.59 (0.48, 0.71) | 0.23 (-1.03, 1.33) |  | 0.34 (0.29, 0.39) | 0.56 (0.39, 0.71) | 1.61 (0, 2.89) |
| **Kiribati** |  |  |  |  |  |  |  |  |  |  |  |  |
| LCHB |  | 1.95 (1.39, 2.67) | 2 (1.35, 2.99) | 0.08 (-2.2, 2.47) |  | 2.32 (1.6, 3.14) | 2.27 (1.4, 3.7) | -0.07 (-2.61, 2.7) |  | 1.59 (1.1, 2.32) | 1.73 (1.12, 2.64) | 0.27 (-2.35, 2.82) |
| LCHC |  | 0.76 (0.52, 1.09) | 0.91 (0.55, 1.37) | 0.58 (-2.21, 3.12) |  | 0.44 (0.27, 0.7) | 0.46 (0.25, 0.8) | 0.14 (-3.32, 3.5) |  | 1.08 (0.71, 1.56) | 1.35 (0.8, 1.99) | 0.72 (-2.15, 3.32) |
| LCAL |  | 0.32 (0.19, 0.49) | 0.34 (0.21, 0.55) | 0.2 (-2.73, 3.43) |  | 0.4 (0.24, 0.65) | 0.4 (0.22, 0.7) | 0 (-3.49, 3.45) |  | 0.24 (0.14, 0.36) | 0.29 (0.17, 0.46) | 0.61 (-2.42, 3.84) |
| LCNA |  | 0.33 (0.22, 0.5) | 0.43 (0.26, 0.61) | 0.85 (-2.11, 3.29) |  | 0.2 (0.13, 0.32) | 0.23 (0.13, 0.4) | 0.45 (-2.91, 3.63) |  | 0.46 (0.29, 0.71) | 0.61 (0.37, 0.91) | 0.91 (-2.1, 3.69) |
| LCOT |  | 0.18 (0.12, 0.28) | 0.21 (0.13, 0.33) | 0.5 (-2.48, 3.26) |  | 0.1 (0.06, 0.16) | 0.1 (0.05, 0.19) | 0 (-3.75, 3.72) |  | 0.26 (0.16, 0.4) | 0.31 (0.19, 0.49) | 0.57 (-2.4, 3.61) |
| **Laos** |  |  |  |  |  |  |  |  |  |  |  |  |
| LCHB |  | 2.51 (1.55, 3.71) | 1.86 (1.19, 2.82) | -0.97 (-3.67, 1.93) |  | 3.96 (2.35, 5.93) | 2.85 (1.73, 4.45) | -1.06 (-3.97, 2.06) |  | 1.1 (0.69, 1.63) | 0.85 (0.5, 1.29) | -0.83 (-3.81, 2.02) |
| LCHC |  | 1.13 (0.68, 1.72) | 0.93 (0.59, 1.4) | -0.63 (-3.45, 2.33) |  | 1.21 (0.67, 1.96) | 0.94 (0.55, 1.55) | -0.81 (-4.1, 2.71) |  | 1.05 (0.63, 1.62) | 0.92 (0.57, 1.37) | -0.43 (-3.37, 2.51) |
| LCAL |  | 1.13 (0.67, 1.81) | 1.16 (0.68, 1.74) | 0.08 (-3.16, 3.08) |  | 1.9 (1.07, 3.12) | 1.88 (1.07, 2.89) | -0.03 (-3.45, 3.21) |  | 0.39 (0.23, 0.61) | 0.43 (0.25, 0.68) | 0.31 (-2.88, 3.5) |
| LCNA |  | 0.37 (0.23, 0.59) | 0.35 (0.2, 0.55) | -0.18 (-3.49, 2.81) |  | 0.4 (0.21, 0.7) | 0.37 (0.2, 0.59) | -0.25 (-4.04, 3.33) |  | 0.35 (0.21, 0.54) | 0.34 (0.2, 0.52) | -0.09 (-3.2, 2.92) |
| LCOT |  | 0.16 (0.09, 0.23) | 0.12 (0.07, 0.19) | -0.93 (-3.84, 2.41) |  | 0.17 (0.09, 0.27) | 0.12 (0.07, 0.21) | -1.12 (-4.35, 2.73) |  | 0.14 (0.09, 0.22) | 0.12 (0.07, 0.19) | -0.5 (-3.69, 2.41) |
| **Malaysia** |  |  |  |  |  |  |  |  |  |  |  |  |
| LCHB |  | 1.34 (0.99, 1.74) | 2.74 (1.97, 3.69) | 2.31 (0.4, 4.24) |  | 2.11 (1.52, 2.78) | 4.49 (3.22, 6.06) | 2.44 (0.47, 4.46) |  | 0.55 (0.39, 0.76) | 0.86 (0.56, 1.21) | 1.44 (-0.99, 3.65) |
| LCHC |  | 0.41 (0.27, 0.59) | 0.92 (0.61, 1.3) | 2.61 (0.11, 5.07) |  | 0.38 (0.23, 0.58) | 0.98 (0.6, 1.47) | 3.06 (0.11, 5.98) |  | 0.44 (0.29, 0.64) | 0.86 (0.59, 1.2) | 2.16 (-0.26, 4.58) |
| LCAL |  | 0.31 (0.2, 0.47) | 0.72 (0.46, 1.14) | 2.72 (-0.07, 5.61) |  | 0.49 (0.31, 0.72) | 1.16 (0.73, 1.82) | 2.78 (0.04, 5.71) |  | 0.14 (0.08, 0.22) | 0.25 (0.15, 0.4) | 1.87 (-1.24, 5.19) |
| LCNA |  | 0.21 (0.13, 0.3) | 0.57 (0.38, 0.85) | 3.22 (0.76, 6.06) |  | 0.2 (0.13, 0.3) | 0.64 (0.4, 0.98) | 3.75 (0.93, 6.52) |  | 0.21 (0.14, 0.32) | 0.51 (0.33, 0.75) | 2.86 (0.1, 5.41) |
| LCOT |  | 0.07 (0.05, 0.1) | 0.14 (0.09, 0.2) | 2.24 (-0.34, 4.47) |  | 0.07 (0.04, 0.1) | 0.15 (0.09, 0.23) | 2.46 (-0.34, 5.64) |  | 0.07 (0.05, 0.1) | 0.12 (0.08, 0.19) | 1.74 (-0.72, 4.31) |
| **Marshall Islands** |  |  |  |  |  |  |  |  |  |  |  |  |
| LCHB |  | 0.69 (0.4, 1.28) | 1.4 (0.74, 2.62) | 2.28 (-1.77, 6.06) |  | 0.87 (0.49, 1.68) | 1.88 (0.88, 4.08) | 2.49 (-2.09, 6.84) |  | 0.51 (0.28, 0.96) | 0.89 (0.52, 1.43) | 1.8 (-1.98, 5.26) |
| LCHC |  | 0.26 (0.14, 0.48) | 0.5 (0.28, 0.9) | 2.11 (-1.74, 6) |  | 0.18 (0.08, 0.36) | 0.4 (0.17, 0.95) | 2.58 (-2.42, 7.98) |  | 0.35 (0.18, 0.67) | 0.62 (0.34, 1.07) | 1.84 (-2.19, 5.75) |
| LCAL |  | 0.13 (0.07, 0.26) | 0.31 (0.16, 0.59) | 2.8 (-1.57, 6.88) |  | 0.18 (0.09, 0.38) | 0.43 (0.19, 0.96) | 2.81 (-2.24, 7.64) |  | 0.09 (0.04, 0.17) | 0.18 (0.1, 0.3) | 2.24 (-1.71, 6.5) |
| LCNA |  | 0.11 (0.06, 0.2) | 0.24 (0.13, 0.4) | 2.52 (-1.39, 6.12) |  | 0.07 (0.04, 0.15) | 0.19 (0.09, 0.42) | 3.22 (-1.65, 7.59) |  | 0.14 (0.07, 0.28) | 0.29 (0.17, 0.48) | 2.35 (-1.61, 6.21) |
| LCOT |  | 0.06 (0.03, 0.11) | 0.12 (0.07, 0.2) | 2.24 (-1.46, 6.12) |  | 0.04 (0.02, 0.08) | 0.09 (0.04, 0.19) | 2.62 (-2.24, 7.26) |  | 0.09 (0.05, 0.17) | 0.16 (0.09, 0.28) | 1.86 (-2.05, 5.56) |
| **Mongolia** |  |  |  |  |  |  |  |  |  |  |  |  |
| LCHB |  | 9.58 (6.28, 14.35) | 13.52 (8.75, 20.41) | 1.11 (-1.6, 3.8) |  | 14.12 (9.08, 21.38) | 19.23 (12.29, 28.5) | 1 (-1.79, 3.69) |  | 5.05 (2.98, 7.96) | 7.95 (5.02, 12.03) | 1.46 (-1.49, 4.5) |
| LCHC |  | 12.06 (7.68, 18.24) | 22.34 (15.78, 30.86) | 1.99 (-0.47, 4.49) |  | 13.03 (7.96, 20.65) | 18.51 (12.54, 27.27) | 1.13 (-1.61, 3.97) |  | 11.09 (7.06, 17) | 26.07 (18.65, 35.12) | 2.76 (0.3, 5.18) |
| LCAL |  | 5.38 (3.23, 8.66) | 12.29 (8.33, 18.47) | 2.66 (-0.13, 5.62) |  | 8.52 (5.18, 14) | 17.48 (11.36, 26.48) | 2.32 (-0.67, 5.26) |  | 2.26 (1.31, 3.82) | 7.23 (4.7, 11.33) | 3.75 (0.67, 6.96) |
| LCNA |  | 1.59 (0.98, 2.48) | 3.53 (2.19, 5.49) | 2.57 (-0.4, 5.56) |  | 1.51 (0.88, 2.48) | 2.63 (1.5, 4.12) | 1.79 (-1.62, 4.98) |  | 1.67 (1, 2.67) | 4.41 (2.75, 6.89) | 3.13 (0.1, 6.23) |
| LCOT |  | 0.91 (0.56, 1.49) | 1.5 (0.95, 2.3) | 1.61 (-1.45, 4.56) |  | 0.79 (0.45, 1.33) | 1.09 (0.66, 1.76) | 1.04 (-2.26, 4.4) |  | 1.04 (0.59, 1.7) | 1.89 (1.2, 2.89) | 1.93 (-1.12, 5.13) |
| **Nauru** |  |  |  |  |  |  |  |  |  |  |  |  |
| LCHB |  | 2.19 (1.52, 3.03) | 1.96 (1.12, 3.15) | -0.36 (-3.21, 2.35) |  | 3.43 (2.32, 4.82) | 3.09 (1.7, 5.24) | -0.34 (-3.36, 2.63) |  | 0.85 (0.56, 1.23) | 0.82 (0.45, 1.35) | -0.12 (-3.24, 2.84) |
| LCHC |  | 0.51 (0.33, 0.77) | 0.52 (0.29, 0.86) | 0.06 (-3.15, 3.09) |  | 0.55 (0.33, 0.86) | 0.51 (0.26, 0.95) | -0.24 (-3.86, 3.41) |  | 0.48 (0.31, 0.73) | 0.52 (0.28, 0.88) | 0.26 (-3.09, 3.37) |
| LCAL |  | 0.41 (0.25, 0.63) | 0.44 (0.25, 0.76) | 0.23 (-2.98, 3.59) |  | 0.66 (0.39, 1.03) | 0.7 (0.37, 1.28) | 0.19 (-3.3, 3.83) |  | 0.15 (0.09, 0.23) | 0.18 (0.1, 0.31) | 0.59 (-2.69, 3.99) |
| LCNA |  | 0.28 (0.17, 0.42) | 0.31 (0.18, 0.48) | 0.33 (-2.73, 3.35) |  | 0.31 (0.18, 0.49) | 0.32 (0.16, 0.55) | 0.1 (-3.61, 3.6) |  | 0.25 (0.15, 0.38) | 0.3 (0.16, 0.49) | 0.59 (-2.79, 3.82) |
| LCOT |  | 0.14 (0.09, 0.21) | 0.15 (0.08, 0.23) | 0.22 (-3.11, 3.03) |  | 0.15 (0.09, 0.23) | 0.14 (0.07, 0.26) | -0.22 (-3.84, 3.42) |  | 0.14 (0.09, 0.22) | 0.15 (0.08, 0.26) | 0.22 (-3.26, 3.42) |
| **New Zealand** |  |  |  |  |  |  |  |  |  |  |  |  |
| LCHB |  | 0.38 (0.31, 0.45) | 0.81 (0.65, 0.98) | 2.44 (1.19, 3.71) |  | 0.59 (0.47, 0.72) | 1.27 (1.02, 1.59) | 2.47 (1.12, 3.93) |  | 0.17 (0.14, 0.2) | 0.34 (0.28, 0.42) | 2.24 (1.09, 3.54) |
| LCHC |  | 0.64 (0.55, 0.74) | 1.97 (1.67, 2.28) | 3.63 (2.63, 4.59) |  | 0.65 (0.53, 0.79) | 1.99 (1.62, 2.38) | 3.61 (2.32, 4.85) |  | 0.64 (0.54, 0.73) | 1.96 (1.61, 2.26) | 3.61 (2.55, 4.62) |
| LCAL |  | 0.98 (0.85, 1.11) | 2.34 (2, 2.69) | 2.81 (1.9, 3.72) |  | 1.61 (1.39, 1.85) | 3.81 (3.18, 4.45) | 2.78 (1.75, 3.75) |  | 0.36 (0.3, 0.43) | 0.86 (0.71, 1.05) | 2.81 (1.62, 4.04) |
| LCNA |  | 0.19 (0.16, 0.23) | 0.71 (0.58, 0.84) | 4.25 (2.98, 5.35) |  | 0.19 (0.15, 0.22) | 0.69 (0.56, 0.84) | 4.16 (3.01, 5.56) |  | 0.2 (0.16, 0.24) | 0.72 (0.57, 0.88) | 4.13 (2.79, 5.5) |
| LCOT |  | 0.11 (0.09, 0.12) | 0.28 (0.23, 0.33) | 3.01 (2.1, 4.19) |  | 0.1 (0.08, 0.13) | 0.27 (0.22, 0.33) | 3.2 (1.7, 4.57) |  | 0.11 (0.09, 0.13) | 0.29 (0.23, 0.34) | 3.13 (1.84, 4.29) |
| **Niue** |  |  |  |  |  |  |  |  |  |  |  |  |
| LCHB |  | 2.02 (1.29, 3.06) | 3.02 (1.84, 4.9) | 1.3 (-1.64, 4.31) |  | 2.97 (1.85, 4.61) | 4.9 (2.87, 8.41) | 1.62 (-1.53, 4.88) |  | 1.07 (0.64, 1.74) | 1.12 (0.64, 1.8) | 0.15 (-3.23, 3.34) |
| LCHC |  | 0.99 (0.6, 1.54) | 1.42 (0.86, 2.21) | 1.16 (-1.88, 4.21) |  | 0.78 (0.46, 1.3) | 1.46 (0.79, 2.59) | 2.02 (-1.61, 5.57) |  | 1.19 (0.71, 1.96) | 1.37 (0.77, 2.16) | 0.45 (-3.01, 3.59) |
| LCAL |  | 0.52 (0.31, 0.84) | 1 (0.57, 1.77) | 2.11 (-1.25, 5.62) |  | 0.78 (0.43, 1.27) | 1.64 (0.92, 3.1) | 2.4 (-1.04, 6.37) |  | 0.26 (0.15, 0.44) | 0.36 (0.2, 0.59) | 1.05 (-2.54, 4.42) |
| LCNA |  | 0.43 (0.26, 0.68) | 0.82 (0.48, 1.29) | 2.08 (-1.12, 5.17) |  | 0.37 (0.21, 0.63) | 0.9 (0.48, 1.58) | 2.87 (-0.88, 6.51) |  | 0.48 (0.28, 0.81) | 0.73 (0.4, 1.15) | 1.35 (-2.28, 4.56) |
| LCOT |  | 0.15 (0.09, 0.25) | 0.22 (0.12, 0.34) | 1.24 (-2.37, 4.29) |  | 0.13 (0.07, 0.23) | 0.23 (0.11, 0.43) | 1.84 (-2.38, 5.86) |  | 0.18 (0.1, 0.31) | 0.2 (0.11, 0.33) | 0.34 (-3.34, 3.85) |
| **Northern Mariana Islands** |  |  |  |  |  |  |  |  |  |  |  |  |
| LCHB |  | 1.33 (0.93, 1.93) | 3.8 (2.86, 5.01) | 3.39 (1.27, 5.43) |  | 1.9 (1.3, 2.82) | 5.84 (4.37, 7.8) | 3.62 (1.41, 5.78) |  | 0.7 (0.46, 1.02) | 1.55 (1.02, 2.21) | 2.56 (0, 5.06) |
| LCHC |  | 0.34 (0.22, 0.52) | 1.32 (0.82, 1.92) | 4.38 (1.47, 6.99) |  | 0.3 (0.18, 0.5) | 1.37 (0.8, 2.1) | 4.9 (1.52, 7.92) |  | 0.39 (0.25, 0.56) | 1.26 (0.84, 1.79) | 3.78 (1.31, 6.35) |
| LCAL |  | 0.23 (0.14, 0.34) | 0.95 (0.59, 1.56) | 4.58 (1.78, 7.78) |  | 0.34 (0.2, 0.52) | 1.49 (0.89, 2.51) | 4.77 (1.73, 8.16) |  | 0.11 (0.06, 0.17) | 0.35 (0.21, 0.55) | 3.73 (0.68, 7.15) |
| LCNA |  | 0.24 (0.16, 0.36) | 0.91 (0.57, 1.32) | 4.3 (1.48, 6.81) |  | 0.22 (0.13, 0.36) | 0.98 (0.59, 1.54) | 4.82 (1.59, 7.97) |  | 0.26 (0.17, 0.39) | 0.83 (0.52, 1.22) | 3.74 (0.93, 6.36) |
| LCOT |  | 0.09 (0.06, 0.15) | 0.23 (0.14, 0.36) | 3.03 (-0.22, 5.78) |  | 0.08 (0.05, 0.13) | 0.23 (0.14, 0.39) | 3.41 (0.24, 6.63) |  | 0.11 (0.07, 0.17) | 0.23 (0.13, 0.38) | 2.38 (-0.87, 5.46) |
| **Palau** |  |  |  |  |  |  |  |  |  |  |  |  |
| LCHB |  | 3.11 (2, 4.75) | 5.93 (3.98, 8.57) | 2.08 (-0.57, 4.69) |  | 6.03 (3.87, 9.21) | 10.77 (7.23, 15.56) | 1.87 (-0.78, 4.49) |  | 0 (0, 0.01) | 0.01 (0, 0.01) | - |
| LCHC |  | 0.67 (0.37, 1.12) | 1.32 (0.75, 2.05) | 2.19 (-1.29, 5.52) |  | 1.29 (0.71, 2.17) | 2.39 (1.37, 3.72) | 1.99 (-1.48, 5.34) |  | 0 (0, 0.01) | 0.01 (0, 0.01) | - |
| LCAL |  | 0.68 (0.4, 1.14) | 1.53 (0.89, 2.41) | 2.62 (-0.8, 5.79) |  | 1.32 (0.78, 2.2) | 2.78 (1.61, 4.38) | 2.4 (-1.01, 5.57) |  | 0 (0, 0) | 0 (0, 0) | - |
| LCNA |  | 0.36 (0.21, 0.62) | 0.92 (0.52, 1.47) | 3.03 (-0.57, 6.28) |  | 0.7 (0.41, 1.21) | 1.67 (0.94, 2.66) | 2.8 (-0.81, 6.03) |  | 0 (0, 0) | 0 (0, 0) | - |
| LCOT |  | 0.13 (0.08, 0.24) | 0.26 (0.15, 0.42) | 2.24 (-1.52, 5.35) |  | 0.26 (0.15, 0.47) | 0.47 (0.27, 0.77) | 1.91 (-1.79, 5.28) |  | 0 (0, 0) | 0 (0, 0) | - |
| **Papua New Guinea** |  |  |  |  |  |  |  |  |  |  |  |  |
| LCHB |  | 1.02 (0.48, 2.56) | 0.73 (0.33, 1.97) | -1.08 (-6.61, 4.55) |  | 1.46 (0.71, 4.04) | 1.05 (0.45, 3.15) | -1.06 (-7.08, 4.81) |  | 0.55 (0.22, 1.25) | 0.39 (0.18, 0.83) | -1.11 (-6.25, 4.28) |
| LCHC |  | 0.33 (0.14, 0.84) | 0.25 (0.11, 0.61) | -0.9 (-6.56, 4.75) |  | 0.31 (0.13, 0.87) | 0.23 (0.08, 0.68) | -0.96 (-7.7, 5.34) |  | 0.36 (0.14, 0.91) | 0.28 (0.12, 0.62) | -0.81 (-6.54, 4.8) |
| LCAL |  | 0.2 (0.09, 0.57) | 0.15 (0.06, 0.44) | -0.93 (-7.26, 5.12) |  | 0.3 (0.14, 0.9) | 0.23 (0.08, 0.73) | -0.86 (-7.81, 5.33) |  | 0.09 (0.03, 0.23) | 0.07 (0.03, 0.16) | -0.81 (-6.57, 5.4) |
| LCNA |  | 0.12 (0.05, 0.31) | 0.1 (0.04, 0.26) | -0.59 (-6.61, 5.32) |  | 0.12 (0.05, 0.33) | 0.1 (0.03, 0.29) | -0.59 (-7.74, 5.67) |  | 0.13 (0.05, 0.32) | 0.11 (0.05, 0.25) | -0.54 (-5.99, 5.19) |
| LCOT |  | 0.08 (0.03, 0.2) | 0.06 (0.03, 0.13) | -0.93 (-6.12, 4.73) |  | 0.06 (0.03, 0.18) | 0.05 (0.02, 0.15) | -0.59 (-7.09, 5.19) |  | 0.09 (0.03, 0.24) | 0.07 (0.03, 0.15) | -0.81 (-6.71, 5.19) |
| **Philippines** |  |  |  |  |  |  |  |  |  |  |  |  |
| LCHB |  | 1.9 (1.32, 2.45) | 1.99 (1.59, 2.48) | 0.15 (-1.39, 2.03) |  | 3.16 (2.16, 3.99) | 3.14 (2.38, 4.12) | -0.02 (-1.67, 2.08) |  | 0.62 (0.42, 1) | 0.8 (0.61, 1.02) | 0.82 (-1.59, 2.86) |
| LCHC |  | 0.57 (0.38, 0.84) | 0.9 (0.7, 1.11) | 1.47 (-0.59, 3.46) |  | 0.66 (0.43, 0.94) | 0.93 (0.68, 1.21) | 1.11 (-1.04, 3.34) |  | 0.47 (0.31, 0.79) | 0.86 (0.66, 1.08) | 1.95 (-0.58, 4.03) |
| LCAL |  | 0.74 (0.49, 1.02) | 1.17 (0.89, 1.49) | 1.48 (-0.44, 3.59) |  | 1.23 (0.78, 1.67) | 1.86 (1.34, 2.44) | 1.33 (-0.71, 3.68) |  | 0.24 (0.15, 0.4) | 0.46 (0.34, 0.6) | 2.1 (-0.52, 4.47) |
| LCNA |  | 0.24 (0.16, 0.34) | 0.39 (0.31, 0.49) | 1.57 (-0.3, 3.61) |  | 0.29 (0.18, 0.39) | 0.43 (0.32, 0.57) | 1.27 (-0.64, 3.72) |  | 0.19 (0.12, 0.31) | 0.35 (0.27, 0.46) | 1.97 (-0.45, 4.33) |
| LCOT |  | 0.11 (0.08, 0.15) | 0.13 (0.1, 0.17) | 0.54 (-1.31, 2.43) |  | 0.14 (0.09, 0.18) | 0.15 (0.11, 0.2) | 0.22 (-1.59, 2.58) |  | 0.08 (0.05, 0.12) | 0.11 (0.08, 0.14) | 1.03 (-1.31, 3.32) |
| **Samoa** |  |  |  |  |  |  |  |  |  |  |  |  |
| LCHB |  | 1.48 (1.06, 2.01) | 1.69 (1.14, 2.38) | 0.43 (-1.83, 2.61) |  | 2.37 (1.68, 3.24) | 2.68 (1.79, 3.8) | 0.4 (-1.91, 2.63) |  | 0.51 (0.33, 0.73) | 0.64 (0.38, 1.01) | 0.73 (-2.11, 3.61) |
| LCHC |  | 0.52 (0.33, 0.77) | 0.66 (0.4, 0.98) | 0.77 (-2.11, 3.51) |  | 0.58 (0.36, 0.93) | 0.7 (0.42, 1.12) | 0.61 (-2.56, 3.66) |  | 0.45 (0.29, 0.65) | 0.62 (0.38, 0.92) | 1.03 (-1.73, 3.72) |
| LCAL |  | 0.36 (0.22, 0.56) | 0.42 (0.25, 0.67) | 0.5 (-2.6, 3.59) |  | 0.58 (0.35, 0.92) | 0.68 (0.4, 1.1) | 0.51 (-2.69, 3.69) |  | 0.11 (0.07, 0.17) | 0.15 (0.09, 0.23) | 1 (-2.05, 3.84) |
| LCNA |  | 0.26 (0.16, 0.41) | 0.35 (0.22, 0.52) | 0.96 (-2.01, 3.8) |  | 0.3 (0.17, 0.5) | 0.39 (0.23, 0.64) | 0.85 (-2.5, 4.28) |  | 0.21 (0.13, 0.34) | 0.31 (0.18, 0.47) | 1.26 (-2.05, 4.15) |
| LCOT |  | 0.1 (0.06, 0.15) | 0.12 (0.07, 0.18) | 0.59 (-2.46, 3.54) |  | 0.11 (0.06, 0.17) | 0.13 (0.07, 0.2) | 0.54 (-2.86, 3.88) |  | 0.09 (0.05, 0.13) | 0.11 (0.06, 0.18) | 0.65 (-2.49, 4.13) |
| **Singapore** |  |  |  |  |  |  |  |  |  |  |  |  |
| LCHB |  | 4.2 (3.54, 4.91) | 4.93 (3.93, 5.98) | 0.52 (-0.72, 1.69) |  | 6.73 (5.75, 7.79) | 7.74 (6.22, 9.29) | 0.45 (-0.73, 1.55) |  | 1.63 (1.26, 2.03) | 2.01 (1.45, 2.65) | 0.68 (-1.09, 2.4) |
| LCHC |  | 1.25 (0.87, 1.71) | 2.14 (1.46, 2.94) | 1.73 (-0.51, 3.93) |  | 1.37 (0.9, 1.98) | 2.37 (1.54, 3.47) | 1.77 (-0.81, 4.35) |  | 1.14 (0.79, 1.51) | 1.9 (1.32, 2.53) | 1.65 (-0.43, 3.75) |
| LCAL |  | 0.39 (0.25, 0.59) | 0.62 (0.39, 0.95) | 1.5 (-1.34, 4.31) |  | 0.66 (0.43, 0.99) | 1.03 (0.64, 1.58) | 1.44 (-1.41, 4.2) |  | 0.12 (0.08, 0.18) | 0.2 (0.12, 0.31) | 1.65 (-1.31, 4.37) |
| LCNA |  | 0.26 (0.17, 0.39) | 0.47 (0.31, 0.71) | 1.91 (-0.74, 4.61) |  | 0.29 (0.18, 0.45) | 0.51 (0.31, 0.82) | 1.82 (-1.2, 4.89) |  | 0.23 (0.15, 0.34) | 0.43 (0.27, 0.64) | 2.02 (-0.74, 4.68) |
| LCOT |  | 0.16 (0.11, 0.22) | 0.21 (0.13, 0.3) | 0.88 (-1.7, 3.24) |  | 0.18 (0.12, 0.27) | 0.23 (0.14, 0.35) | 0.79 (-2.12, 3.45) |  | 0.13 (0.09, 0.19) | 0.18 (0.12, 0.27) | 1.05 (-1.48, 3.54) |
| **Solomon Islands** |  |  |  |  |  |  |  |  |  |  |  |  |
| LCHB |  | 1.77 (0.55, 3.92) | 1.58 (0.96, 2.62) | -0.37 (-4.54, 5.04) |  | 2.9 (0.87, 6.4) | 2.5 (1.52, 4.41) | -0.48 (-4.64, 5.24) |  | 0.55 (0.18, 1.21) | 0.61 (0.37, 1.06) | 0.33 (-3.82, 5.72) |
| LCHC |  | 0.42 (0.15, 1.01) | 0.44 (0.24, 0.79) | 0.15 (-4.64, 5.36) |  | 0.52 (0.16, 1.3) | 0.46 (0.24, 0.9) | -0.4 (-5.45, 5.57) |  | 0.32 (0.12, 0.74) | 0.41 (0.23, 0.76) | 0.8 (-3.77, 5.95) |
| LCAL |  | 0.31 (0.09, 0.76) | 0.3 (0.17, 0.52) | -0.11 (-4.83, 5.66) |  | 0.52 (0.15, 1.28) | 0.48 (0.27, 0.88) | -0.26 (-5.02, 5.71) |  | 0.08 (0.03, 0.21) | 0.11 (0.06, 0.2) | 1.03 (-4.04, 6.12) |
| LCNA |  | 0.19 (0.06, 0.44) | 0.22 (0.12, 0.37) | 0.47 (-4.19, 5.87) |  | 0.23 (0.07, 0.59) | 0.24 (0.12, 0.45) | 0.14 (-5.14, 6) |  | 0.14 (0.05, 0.31) | 0.19 (0.11, 0.34) | 0.99 (-3.34, 6.18) |
| LCOT |  | 0.11 (0.04, 0.26) | 0.11 (0.07, 0.2) | 0 (-4.23, 5.19) |  | 0.12 (0.04, 0.3) | 0.12 (0.06, 0.21) | 0 (-5.19, 5.35) |  | 0.09 (0.03, 0.22) | 0.11 (0.07, 0.19) | 0.65 (-3.69, 5.95) |
| **South Korea** |  |  |  |  |  |  |  |  |  |  |  |  |
| LCHB |  | 16.13 (11.55, 20.6) | 14.35 (11.26, 18.33) | -0.38 (-1.95, 1.49) |  | 27.29 (19.57, 35.38) | 24.68 (19.36, 31.53) | -0.32 (-1.94, 1.54) |  | 4.87 (3.18, 6.69) | 3.94 (2.66, 5.7) | -0.68 (-2.98, 1.88) |
| LCHC |  | 3.58 (2.35, 5.3) | 4.94 (3.3, 7.04) | 1.04 (-1.53, 3.54) |  | 2.61 (1.59, 4.09) | 4.04 (2.46, 6.26) | 1.41 (-1.64, 4.42) |  | 4.57 (2.88, 6.6) | 5.85 (3.9, 8.18) | 0.8 (-1.7, 3.37) |
| LCAL |  | 3.1 (1.95, 4.48) | 4.53 (2.98, 6.6) | 1.22 (-1.32, 3.93) |  | 4.76 (2.93, 7.06) | 7.3 (4.69, 10.89) | 1.38 (-1.32, 4.23) |  | 1.42 (0.83, 2.19) | 1.73 (1.04, 2.62) | 0.64 (-2.4, 3.71) |
| LCNA |  | 1.33 (0.84, 2) | 1.92 (1.21, 2.88) | 1.18 (-1.62, 3.97) |  | 1.18 (0.71, 1.8) | 1.85 (1.11, 2.96) | 1.45 (-1.56, 4.61) |  | 1.49 (0.91, 2.26) | 1.99 (1.25, 3.1) | 0.93 (-1.91, 3.95) |
| LCOT |  | 0.75 (0.48, 1.14) | 0.78 (0.48, 1.12) | 0.13 (-2.79, 2.73) |  | 0.74 (0.46, 1.15) | 0.82 (0.51, 1.24) | 0.33 (-2.62, 3.2) |  | 0.77 (0.47, 1.18) | 0.73 (0.44, 1.09) | -0.17 (-3.18, 2.71) |
| **Tokelau** |  |  |  |  |  |  |  |  |  |  |  |  |
| LCHB |  | 1.58 (0.91, 2.83) | 2.57 (1.51, 4.15) | 1.57 (-2.03, 4.89) |  | 1.93 (1.07, 3.83) | 3.76 (2.14, 6.28) | 2.15 (-1.88, 5.71) |  | 1.23 (0.64, 2.28) | 1.32 (0.75, 2.24) | 0.23 (-3.59, 4.04) |
| LCHC |  | 0.84 (0.46, 1.6) | 1.09 (0.61, 1.88) | 0.84 (-3.11, 4.54) |  | 0.56 (0.29, 1.16) | 1.01 (0.54, 1.76) | 1.9 (-2.47, 5.82) |  | 1.12 (0.6, 2.23) | 1.17 (0.69, 2.03) | 0.14 (-3.78, 3.93) |
| LCAL |  | 0.37 (0.19, 0.73) | 0.67 (0.36, 1.15) | 1.92 (-2.28, 5.81) |  | 0.49 (0.24, 1.05) | 1.02 (0.55, 1.76) | 2.37 (-2.09, 6.43) |  | 0.25 (0.12, 0.53) | 0.3 (0.15, 0.57) | 0.59 (-4.07, 5.03) |
| LCNA |  | 0.32 (0.17, 0.63) | 0.55 (0.3, 0.96) | 1.75 (-2.39, 5.58) |  | 0.23 (0.11, 0.49) | 0.54 (0.28, 0.96) | 2.75 (-1.81, 6.99) |  | 0.42 (0.21, 0.84) | 0.57 (0.3, 1.01) | 0.99 (-3.32, 5.07) |
| LCOT |  | 0.14 (0.07, 0.28) | 0.18 (0.1, 0.32) | 0.81 (-3.32, 4.9) |  | 0.09 (0.04, 0.18) | 0.16 (0.08, 0.3) | 1.86 (-2.62, 6.5) |  | 0.2 (0.1, 0.4) | 0.21 (0.12, 0.38) | 0.16 (-3.88, 4.31) |
| **Tonga** |  |  |  |  |  |  |  |  |  |  |  |  |
| LCHB |  | 6.99 (4.74, 10.05) | 8.41 (5.51, 12.08) | 0.6 (-1.94, 3.02) |  | 11.38 (7.83, 16.66) | 14.42 (9.4, 21.18) | 0.76 (-1.85, 3.21) |  | 2.53 (1.47, 4.18) | 2.43 (1.52, 3.61) | -0.13 (-3.26, 2.9) |
| LCHC |  | 2.31 (1.33, 3.8) | 3.23 (2.01, 4.85) | 1.08 (-2.05, 4.17) |  | 2.69 (1.54, 4.57) | 3.95 (2.27, 6.33) | 1.24 (-2.26, 4.56) |  | 1.91 (1.02, 3.38) | 2.52 (1.49, 3.65) | 0.89 (-2.64, 4.11) |
| LCAL |  | 1.4 (0.81, 2.33) | 1.92 (1.13, 3.08) | 1.02 (-2.33, 4.31) |  | 2.35 (1.34, 4.01) | 3.34 (1.95, 5.38) | 1.13 (-2.33, 4.48) |  | 0.44 (0.22, 0.76) | 0.52 (0.29, 0.84) | 0.54 (-3.11, 4.32) |
| LCNA |  | 1.12 (0.64, 1.86) | 1.83 (1.11, 2.72) | 1.58 (-1.67, 4.67) |  | 1.34 (0.79, 2.26) | 2.33 (1.34, 3.8) | 1.78 (-1.69, 5.07) |  | 0.9 (0.48, 1.58) | 1.33 (0.79, 2.07) | 1.26 (-2.24, 4.71) |
| LCOT |  | 0.46 (0.28, 0.76) | 0.57 (0.34, 0.88) | 0.69 (-2.59, 3.69) |  | 0.51 (0.29, 0.86) | 0.69 (0.4, 1.15) | 0.98 (-2.47, 4.44) |  | 0.41 (0.23, 0.72) | 0.45 (0.26, 0.7) | 0.3 (-3.29, 3.59) |
| **Tuvalu** |  |  |  |  |  |  |  |  |  |  |  |  |
| LCHB |  | 1.79 (1.16, 2.8) | 2.07 (1.29, 3.17) | 0.47 (-2.5, 3.24) |  | 2.49 (1.6, 4.02) | 3 (1.83, 4.96) | 0.6 (-2.54, 3.65) |  | 1.13 (0.64, 1.95) | 1.07 (0.66, 1.67) | -0.18 (-3.49, 3.09) |
| LCHC |  | 0.71 (0.42, 1.23) | 0.87 (0.54, 1.4) | 0.66 (-2.66, 3.88) |  | 0.56 (0.31, 1.02) | 0.75 (0.4, 1.34) | 0.94 (-3.02, 4.72) |  | 0.86 (0.48, 1.59) | 1.01 (0.64, 1.68) | 0.52 (-2.94, 4.04) |
| LCAL |  | 0.37 (0.21, 0.65) | 0.5 (0.28, 0.87) | 0.97 (-2.72, 4.59) |  | 0.54 (0.3, 0.96) | 0.74 (0.39, 1.32) | 1.02 (-2.91, 4.78) |  | 0.21 (0.1, 0.39) | 0.25 (0.14, 0.43) | 0.56 (-3.3, 4.71) |
| LCNA |  | 0.29 (0.16, 0.5) | 0.43 (0.25, 0.7) | 1.27 (-2.24, 4.76) |  | 0.23 (0.13, 0.44) | 0.39 (0.2, 0.72) | 1.7 (-2.54, 5.52) |  | 0.34 (0.18, 0.63) | 0.47 (0.28, 0.79) | 1.04 (-2.62, 4.77) |
| LCOT |  | 0.14 (0.08, 0.26) | 0.16 (0.1, 0.27) | 0.43 (-3.08, 3.92) |  | 0.11 (0.06, 0.19) | 0.14 (0.07, 0.26) | 0.78 (-3.22, 4.73) |  | 0.18 (0.1, 0.32) | 0.19 (0.11, 0.31) | 0.17 (-3.44, 3.65) |
| **Vanuatu** |  |  |  |  |  |  |  |  |  |  |  |  |
| LCHB |  | 1.2 (0.72, 2.29) | 1.36 (0.84, 2.23) | 0.4 (-3.24, 3.65) |  | 1.84 (1.13, 3.65) | 2.08 (1.22, 3.63) | 0.4 (-3.54, 3.76) |  | 0.53 (0.25, 1.12) | 0.63 (0.38, 1.07) | 0.56 (-3.49, 4.69) |
| LCHC |  | 0.37 (0.2, 0.71) | 0.5 (0.29, 0.83) | 0.97 (-2.89, 4.59) |  | 0.38 (0.21, 0.78) | 0.48 (0.25, 0.95) | 0.75 (-3.67, 4.87) |  | 0.35 (0.18, 0.76) | 0.51 (0.31, 0.84) | 1.21 (-2.89, 4.97) |
| LCAL |  | 0.22 (0.12, 0.45) | 0.29 (0.15, 0.49) | 0.89 (-3.54, 4.54) |  | 0.34 (0.19, 0.73) | 0.45 (0.22, 0.81) | 0.9 (-3.87, 4.68) |  | 0.08 (0.04, 0.19) | 0.12 (0.07, 0.21) | 1.31 (-3.22, 5.35) |
| LCNA |  | 0.15 (0.08, 0.31) | 0.23 (0.13, 0.4) | 1.38 (-2.8, 5.19) |  | 0.16 (0.09, 0.34) | 0.24 (0.12, 0.45) | 1.31 (-3.36, 5.19) |  | 0.15 (0.07, 0.32) | 0.23 (0.14, 0.39) | 1.38 (-2.67, 5.54) |
| LCOT |  | 0.08 (0.05, 0.17) | 0.1 (0.06, 0.18) | 0.72 (-3.36, 4.13) |  | 0.08 (0.05, 0.16) | 0.1 (0.05, 0.19) | 0.72 (-3.75, 4.31) |  | 0.09 (0.04, 0.2) | 0.11 (0.06, 0.19) | 0.65 (-3.88, 5.03) |
| **Viet Nam** |  |  |  |  |  |  |  |  |  |  |  |  |
| LCHB |  | 4.39 (2.85, 6.08) | 5.54 (3.6, 8.47) | 0.75 (-1.69, 3.51) |  | 7.44 (4.72, 10.44) | 9.17 (5.67, 14.59) | 0.67 (-1.97, 3.64) |  | 1.5 (1, 2.11) | 1.93 (1.16, 2.9) | 0.81 (-1.93, 3.43) |
| LCHC |  | 1.5 (0.96, 2.37) | 2.11 (1.23, 3.3) | 1.1 (-2.12, 3.98) |  | 2.04 (1.2, 3.32) | 2.73 (1.51, 4.64) | 0.94 (-2.54, 4.36) |  | 1 (0.64, 1.49) | 1.5 (0.89, 2.2) | 1.31 (-1.66, 3.98) |
| LCAL |  | 1.53 (0.97, 2.36) | 3.61 (2.21, 5.73) | 2.77 (-0.21, 5.73) |  | 2.71 (1.68, 4.17) | 6.17 (3.68, 9.9) | 2.65 (-0.4, 5.72) |  | 0.42 (0.27, 0.65) | 1.07 (0.62, 1.77) | 3.02 (-0.15, 6.07) |
| LCNA |  | 0.61 (0.38, 0.96) | 1.02 (0.64, 1.52) | 1.66 (-1.31, 4.47) |  | 0.73 (0.42, 1.18) | 1.17 (0.69, 1.9) | 1.52 (-1.73, 4.87) |  | 0.5 (0.3, 0.78) | 0.86 (0.52, 1.3) | 1.75 (-1.31, 4.73) |
| LCOT |  | 0.24 (0.14, 0.35) | 0.32 (0.19, 0.5) | 0.93 (-1.97, 4.11) |  | 0.29 (0.17, 0.45) | 0.39 (0.22, 0.65) | 0.96 (-2.31, 4.33) |  | 0.18 (0.11, 0.28) | 0.26 (0.15, 0.41) | 1.19 (-2.01, 4.24) |
| **Western Pacific Region** |  |  |  |  |  |  |  |  |  |  |  |  |
| LCHB |  | 4.97 (4.25, 5.78) | 6.28 (5.03, 7.87) | 0.75 (-0.45, 1.99) |  | 8.15 (6.75, 9.71) | 10.41 (8.08, 13.5) | 0.79 (-0.59, 2.24) |  | 1.66 (1.33, 2.03) | 2.01 (1.53, 2.56) | 0.62 (-0.91, 2.11) |
| LCHC |  | 2.09 (1.83, 2.38) | 3.41 (2.83, 3.95) | 1.58 (0.56, 2.48) |  | 2.26 (1.98, 2.59) | 3.45 (2.9, 4.12) | 1.36 (0.36, 2.36) |  | 1.91 (1.59, 2.25) | 3.37 (2.68, 4.06) | 1.83 (0.56, 3.02) |
| LCAL |  | 0.88 (0.71, 1.09) | 1.6 (1.24, 2.05) | 1.93 (0.42, 3.42) |  | 1.26 (0.99, 1.59) | 2.29 (1.72, 3.03) | 1.93 (0.25, 3.61) |  | 0.49 (0.38, 0.61) | 0.89 (0.67, 1.12) | 1.93 (0.3, 3.49) |
| LCNA |  | 0.41 (0.33, 0.49) | 0.78 (0.62, 0.97) | 2.07 (0.76, 3.48) |  | 0.42 (0.33, 0.52) | 0.81 (0.6, 1.05) | 2.12 (0.46, 3.73) |  | 0.4 (0.31, 0.49) | 0.76 (0.6, 0.96) | 2.07 (0.65, 3.65) |
| LCOT |  | 0.4 (0.33, 0.49) | 0.51 (0.4, 0.64) | 0.78 (-0.65, 2.14) |  | 0.41 (0.33, 0.51) | 0.54 (0.4, 0.74) | 0.89 (-0.78, 2.61) |  | 0.4 (0.31, 0.49) | 0.49 (0.37, 0.63) | 0.65 (-0.91, 2.29) |
| **Whole World** |  |  |  |  |  |  |  |  |  |  |  |  |
| LCHB |  | 2 (1.72, 2.33) | 2.3 (1.89, 2.81) | 0.45 (-0.68, 1.58) |  | 3.2 (2.74, 3.75) | 3.7 (2.99, 4.61) | 0.47 (-0.73, 1.68) |  | 0.77 (0.62, 0.96) | 0.88 (0.7, 1.09) | 0.43 (-1.02, 1.82) |
| LCHC |  | 1.2 (1.04, 1.42) | 1.86 (1.6, 2.14) | 1.41 (0.38, 2.33) |  | 1.23 (1.06, 1.44) | 1.89 (1.59, 2.23) | 1.39 (0.32, 2.4) |  | 1.18 (1, 1.41) | 1.82 (1.54, 2.09) | 1.4 (0.28, 2.38) |
| LCAL |  | 0.72 (0.58, 0.87) | 1.17 (0.95, 1.42) | 1.57 (0.28, 2.89) |  | 1.08 (0.89, 1.32) | 1.8 (1.47, 2.19) | 1.65 (0.35, 2.9) |  | 0.35 (0.27, 0.43) | 0.53 (0.42, 0.66) | 1.34 (-0.08, 2.88) |
| LCNA |  | 0.28 (0.22, 0.34) | 0.52 (0.42, 0.63) | 2 (0.68, 3.39) |  | 0.27 (0.21, 0.33) | 0.52 (0.41, 0.64) | 2.11 (0.7, 3.59) |  | 0.29 (0.22, 0.36) | 0.52 (0.42, 0.64) | 1.88 (0.5, 3.44) |
| LCOT |  | 0.2 (0.16, 0.25) | 0.26 (0.21, 0.32) | 0.85 (-0.56, 2.24) |  | 0.19 (0.16, 0.24) | 0.26 (0.2, 0.33) | 1.01 (-0.59, 2.34) |  | 0.21 (0.16, 0.26) | 0.26 (0.21, 0.32) | 0.69 (-0.69, 2.24) |

Abbreviation: UI, Uncertainty interval; EAPC, Estimated annual percentage change; CI, Confidence interval; LCHB, Liver cancer by HBC; LCHC, Liver cancer by HCV; LCAL, Liver cancer by alcoholic use; LCNA; Liver cancer by nonalcoholic steatohepatitis (NASH); LCOT, Liver cancer by other causes

Table S13. Trends in human resources for health density (1990–2019), socio-demographic index (1990-2021) and universal health coverage (1990-2021) in the Western Pacific region by member state

| **Indicators/Countries** | **Years** | |  |
| --- | --- | --- | --- |
| **HRH (workers per 10,000 population)** | **1990** | **2019** | **EAPC, % (95% CI)** |
| ***Aged countries*** |  |  |  |
| **South Korea** | 19.94 (14.03, 27.53) | 30.15 (21.78, 40.67) | 1.44 (1.35, 1.53) |
| **Cook Islands** | 94.03 (67.6, 129.45) | 165.19 (123.07, 220.05) | 1.96 (1.85, 2.09) |
| **Japan** | 198.46 (148.15, 264.79) | 386.81 (314.84, 471.29) | 2.33 (2.01, 2.63) |
| **China** | 29.4 (21.14, 40.03) | 140.23 (103.12, 182.5) | 5.53 (5.37, 5.62) |
| **Australia** | 346.87 (285.72, 408.32) | 483.13 (383.87, 610.84) | 1.15 (1.02, 1.4) |
| **Singapore** | 106.51 (76.9, 140.42) | 366.23 (269.3, 490.45) | 4.42 (4.41, 4.35) |
| **New Zealand** | 312.68 (249.56, 385.97) | 471.55 (363.81, 594.96) | 1.43 (1.31, 1.5) |
| ***Aging countries*** |  |  |  |
| **Viet Nam** | 21.52 (15.73, 28.98) | 79.36 (57.5, 108.79) | 4.6 (4.57, 4.67) |
| **Palau** | 100.85 (71.64, 135.41) | 167.35 (120.28, 221.58) | 1.76 (1.71, 1.8) |
| **Northern Mariana Islands** | 155.43 (114.82, 205.13) | 198.27 (146.32, 260.32) | 0.84 (0.82, 0.84) |
| **Guam** | 189.36 (139.4, 250.16) | 215.37 (160.34, 287.12) | 0.48 (0.44, 0.48) |
| **American Samoa** | 83.1 (59.67, 110.33) | 114.69 (83.12, 153.18) | 1.14 (1.12, 1.15) |
| **Niue** | 65.52 (46.04, 88.12) | 118.76 (86.36, 159.94) | 2.08 (2.07, 2.19) |
| **Malaysia** | 54.91 (41.71, 70.56) | 147.26 (108.74, 192.97) | 3.46 (3.36, 3.53) |
| **Tokelau** | 35.48 (25.23, 47.31) | 87.55 (62.77, 116.74) | 3.16 (3.16, 3.19) |
| **Tuvalu** | 38.12 (27.77, 51.25) | 64.15 (46.2, 85.53) | 1.78 (1.77, 1.81) |
| ***Young countries*** |  |  |  |
| **Mongolia** | 53.56 (39.97, 69.81) | 127.94 (107.7, 150.99) | 3.05 (2.7, 3.48) |
| **Tonga** | 29.12 (21.23, 39) | 47.06 (33.9, 62.34) | 1.63 (1.63, 1.67) |
| **Brunei Darussalam** | 129.01 (94.06, 175.18) | 236.72 (172.62, 317.24) | 2.12 (2.07, 2.12) |
| **Cambodia** | 21.42 (17.19, 26.16) | 64.23 (52.72, 76.85) | 3.86 (3.79, 3.94) |
| **Vanuatu** | 17.71 (12.88, 23.55) | 36.32 (26.22, 49.33) | 2.51 (2.48, 2.58) |
| **Laos** | 18.81 (13.84, 25) | 51.65 (36.62, 70.12) | 3.54 (3.41, 3.62) |
| **Kiribati** | 19.49 (13.82, 26.45) | 52.45 (38.29, 71.27) | 3.48 (3.47, 3.58) |
| **Federated States of Micronesia** | 25.12 (18.24, 33.54) | 51.69 (36.92, 70.87) | 2.52 (2.46, 2.61) |
| **Nauru** | 51.14 (35.82, 68.35) | 81.22 (59.26, 106.86) | 1.61 (1.55, 1.75) |
| **Fiji** | 44.36 (31.79, 60.21) | 80.64 (57.56, 107) | 2.07 (2, 2.08) |
| **Samoa** | 23.81 (17.15, 32.44) | 36.3 (26.52, 49.66) | 1.48 (1.46, 1.51) |
| **Solomon Islands** | 11.46 (8.19, 15.7) | 42.03 (30.31, 55.68) | 4.58 (4.46, 4.62) |
| **Philippines** | 44.12 (35.21, 54.64) | 65.52 (53.93, 77.89) | 1.37 (1.23, 1.48) |
| **Marshall Islands** | 26.27 (19.06, 35.25) | 59.09 (42.92, 80.77) | 2.84 (2.83, 2.9) |
| **Papua New Guinea** | 15.86 (11.24, 21.99) | 24.24 (17.54, 33.38) | 1.47 (1.45, 1.55) |
| **SDI** | **1990** | **2021** | **Percetage change (%)** |
| ***Aged countries*** |  |  |  |
| **South Korea** | 0.69 | 0.89 | 28.99 |
| **Cook Islands** | 0.56 | 0.78 | 39.29 |
| **Japan** | 0.79 | 0.87 | 10.13 |
| **China** | 0.46 | 0.72 | 56.52 |
| **Australia** | 0.73 | 0.84 | 15.07 |
| **Singapore** | 0.69 | 0.86 | 24.64 |
| **New Zealand** | 0.75 | 0.85 | 13.33 |
| ***Aging countries*** |  |  |  |
| **Viet Nam** | 0.41 | 0.63 | 53.66 |
| **Palau** | 0.66 | 0.75 | 13.64 |
| **Northern Mariana Islands** | 0.71 | 0.77 | 8.45 |
| **Guam** | 0.68 | 0.8 | 17.65 |
| **American Samoa** | 0.61 | 0.72 | 18.03 |
| **Niue** | 0.59 | 0.73 | 23.73 |
| **Malaysia** | 0.55 | 0.74 | 34.55 |
| **Tokelau** | 0.52 | 0.69 | 32.69 |
| **Tuvalu** | 0.41 | 0.58 | 41.46 |
| ***Young countries*** |  |  |  |
| **Mongolia** | 0.47 | 0.62 | 31.91 |
| **Tonga** | 0.49 | 0.63 | 28.57 |
| **Brunei Darussalam** | 0.67 | 0.81 | 20.9 |
| **Cambodia** | 0.29 | 0.47 | 62.07 |
| **Vanuatu** | 0.35 | 0.47 | 34.29 |
| **Laos** | 0.26 | 0.49 | 88.46 |
| **Kiribati** | 0.41 | 0.53 | 29.27 |
| **Federated States of Micronesia** | 0.46 | 0.59 | 28.26 |
| **Nauru** | 0.54 | 0.63 | 16.67 |
| **Fiji** | 0.53 | 0.68 | 28.3 |
| **Samoa** | 0.49 | 0.59 | 20.41 |
| **Solomon Islands** | 0.3 | 0.43 | 43.33 |
| **Philippines** | 0.51 | 0.65 | 27.45 |
| **Marshall Islands** | 0.43 | 0.57 | 32.56 |
| **Papua New Guinea** | 0.31 | 0.42 | 35.48 |
| **UHC service coverage index** | **2000** | **2021** |  |
| ***Aged countries*** |  |  |  |
| **South Korea** | 73 | 89 | 21.92 |
| **Cook Islands** | 47 | 46 | -2.13 |
| **Japan** | 70 | 83 | 18.57 |
| **China** | 47 | 81 | 72.34 |
| **Australia** | 80 | 87 | 8.75 |
| **Singapore** | 64 | 89 | 39.06 |
| **New Zealand** | 75 | 85 | 13.33 |
| ***Aging countries*** |  |  |  |
| **Viet Nam** | 37 | 68 | 83.78 |
| **Palau** | 47 | 65 | 38.3 |
| **Northern Mariana Islands** | NA | NA | NA |
| **Guam** | NA | NA | NA |
| **American Samoa** | NA | 55 | NA |
| **Niue** | 42 | 44 | 4.76 |
| **Malaysia** | 52 | 76 | 46.15 |
| **Tokelau** | NA | NA | NA |
| **Tuvalu** | 37 | 52 | 40.54 |
| ***Young countries*** |  |  |  |
| **Mongolia** | 45 | 65 | 44.44 |
| **Tonga** | 43 | 57 | 32.56 |
| **Brunei Darussalam** | 49 | 78 | 59.18 |
| **Cambodia** | 24 | 58 | 141.67 |
| **Vanuatu** | 31 | 47 | 51.61 |
| **Laos** | 25 | 52 | 108 |
| **Kiribati** | 28 | 48 | 71.43 |
| **Federated States of Micronesia** | 36 | 48 | 33.33 |
| **Nauru** | 35 | 60 | 71.43 |
| **Fiji** | 42 | 58 | 38.1 |
| **Samoa** | 41 | 55 | 34.15 |
| **Solomon Islands** | 31 | 47 | 51.61 |
| **Philippines** | 36 | 58 | 61.11 |
| **Marshall Islands** | 42 | 59 | 40.48 |
| **Papua New Guinea** | 25 | 30 | 20 |

Abbreviation: HRH, Human resources for health; SDI, Socio-demographic index; UHC, Universal health coverage
